# Space groups and crystallographic symmetry: writing a multi-featured tutorial in a new style

**DOI:** 10.1107/S2056989021007039

**Published:** 2021-07-16

**Authors:** Bruce M. Foxman

**Affiliations:** a Department of Chemistry, MS015, Brandeis University, Waltham, MA 02454-9110, USA

**Keywords:** space groups, symmetry, crystallographic teaching

## Abstract

Knowledge of space groups and the implications of space group symmetry on the physical and chemical properties of solids are pivotal factors in all areas of crystalline solids. As Jerry Jasinski and I met to bring our ideas in teaching this subject to life, we both felt that ‘early and often’ – teaching the concepts with textual and visual reinforcement, is the key to providing a sound basis for students in this subject. The tutorial contains *>* 200 PowerPoint ‘slides’, in five modules, arranged by crystal class; a sixth module covers special topics. A ‘credits’ module gives the direct addresses of all embedded links. Space-group diagrams appear in Inter­national Tables format. The triclinic and monoclinic groups (2 + 13) are built from ‘scratch’, and are derived from the Hermann–Mauguin symbol. An additional section provides practice on many (but not all) of the ortho­rhom­bic groups in crystal class 222. Finally, a ‘Special Topics’ section on enanti­omorphous space groups features space groups *P*4_1_ and *P*4_3_. In the tutorial, lattice points build iteratively and inter­actively *via* keyclick, and the coordinates of points ‘pop up’ as the unit cell is filled. We trust that the elements of guidance, inquiry and occasional humor will make the learning process enjoyable.

## Abstract 2

Knowledge of space groups and the implications of space group symmetry on the physical and chemical properties of solids are pivotal factors in all areas of structural science. The tutorial contains *>* 200 PowerPoint ‘slides’, in five modules, arranged by crystal class; a sixth module covers special topics. A ‘credits’ module gives the direct addresses of all embedded links. In the tutorial, lattice points build iteratively and inter­actively with keyclick, and the coordinates of points ‘pop up’ as the unit cell is filled.

## Introduction   

Nearly two decades ago, the late Jerry Jasinski (Keene State College, New Hampshire, USA) and I conceived and wrote an extensive tutorial (now over 220 multi-part PowerPoint ‘slides’) and placed it on the Web. The number of *registered* downloads (registration is not required) numbers in the thousands. One rarely gets an opportunity to describe the conception, ideas, evolution, goals and progress of the preparation of a successful piece of teaching material, and I hope that this will encourage others to see that a different approach, using as simple a tool as PowerPoint, can provide a stimulating, enjoyable experience for students and teachers alike. My friend Lachlan Cranswick, called away at far too early a time, played an important role in stimulating the development of the tutorial. Jerry Jasinski spent time at Brandeis, where we worked closely together; he played a major role in the ideas, pedagogy, and the nuts and bolts of the production. With sadness yet with warm remembrances, I dedicate this contribution to Lachlan and Jerry, and provide a few anecdotes that illustrate their warmth and sincere helpfulness. In this paper I will describe how we conceived the tutorial, thought about many new and old aspects that needed to be there, and then developed it into an independent package that connects well with students, and has permanence, important issues that will be described below.

## First thoughts   

During Summer 2003, Professor Jerry Jasinski and an undergraduate student from Keene State College, Lisa Bennett, worked in my laboratory as part of the National Science Foundation Summer Research Program in Solid State Chemistry, Clemson University. The Clemson program first brought faculty and students to Clemson for a week and then these folks were ‘placed’ into various ‘host’ laboratories throughout the country. It was a great experience for all, and Jerry asked Professor Shiou-Jyh Hwu (Clemson) if he could return during Summer 2004. Fortunately, this turned out to be possible, and, in anti­cipation of requests of this type, Shiou-Jyh had set up the requirement that, in the second year, a faculty applicant must participate, along with her/his host, in a project involving education in solid-state chemistry. Further, the nature of that project would have to be pre-approved by Shiou-Jyh and his local committee at Clemson University. In retrospect, our initial plan was not highly ambitious: we proposed to assemble a course in X-ray structure determination using extant Web material. Our contribution would be to assemble the best materials, and then to link all, with commentary, on a single Web site.

Off we went in early June 2004, finding many nice things presented about unit cells and point-group symmetry. In 2004, we were at a standstill trying to find an extensive set of material on space groups that, from our perspective and experience, took advantage of modern presentation ideas and techniques, had (insofar as possible) a warm ‘lecture style’, and progressed through the groups in a logical, incremental fashion. The best one we found was part of the Bucknell University series *Crystallographic CourseWare* (Kastner, 1999 ▸; Kastner *et al.*, 2000 ▸). At the time we were considering our project, *Crystallographic CourseWare* was available on CD for purchase from JCESoftware; a free version is now available online. In 2004, we felt that an approach that offered a space-group tutorial without charge was a preferred option. Thus we took a right turn: instead of assembling a broad course, we decided to attempt a different stylistic tactic in the teaching of space-group symmetry. What vehicle would we use? While it might have been nice to develop a give and take, animated approach with an answer set (*e.g*., with Flash player, or even a tutorial developed with a high-level programming language), this was clearly beyond both our skill sets as well as the limitations of available time. Finally, we decided to test out the idea of making an inter­active tutorial with PowerPoint. On the down side in 2004, I thought of PowerPoint as a substitute option for no longer carrying a stack of transparencies to class or a meeting, and little more. At the bottom of the learning curve, we set out to see if (*a*) we could develop the artwork/set of crystallographic symbols necessary for the presentation, and (*b*) we could ‘build up’ space-group diagrams from only the symbol, one equivalent position at a time, as I had learned from postdoctoral fellow Dr Michael J. Bennett, a.k.a. SuperBennett, while a PhD student in Professor Al Cotton’s research group at MIT in the 1960s, and just as I had carried on the SuperBennett tradition in my lectures at Brandeis since 1975 or so. It soon became apparent that this was entirely possible, and in fact could be done in a very pretty, lively presentation as we shall discuss below. Given that the discussion will proceed in a publication document, we will not be showing the dynamic nature of the presentation in this paper, rather the ‘final’ slide in each case. If you have not seen the full tutorial, please download and run it on your PC (Jasinski & Foxman, 2007 ▸) or Mac (Jasinski *et al.*, 2015 ▸), while you read along. Each slide caption will allow you to locate that inter­active slide as you read. In the inter­ests of permanence, the files are also available as supporting information (files *symandsg.zip* and *symandsg_mac.zip*).

## Tutorial development   

**Figure 1:**
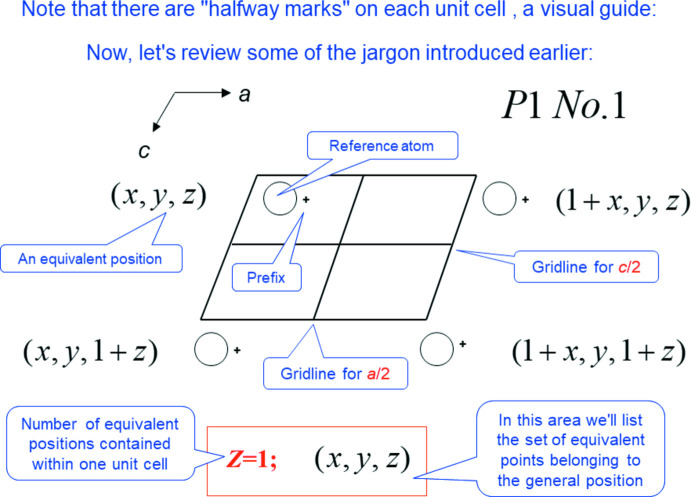
Slide 21, Chapter 1.

As we approached a final first draft of the tutorial, we decided to open with a large number of definitions, and a relatively complete background and/or numerous referrals to reference material. Then, spurred by our success in the symbol and tool development, we moved to the generation of space-group diagrams along the lines of the Inter­national Tables for X-ray Crystallography, Volume A (2006 ▸). The *P*1 diagram below (Fig. 1 ▸) takes eleven keyclicks to set up the visuals, and seven more to add in the jargon/definitions. Try it now in your downloaded copy.

**Figure 2:**
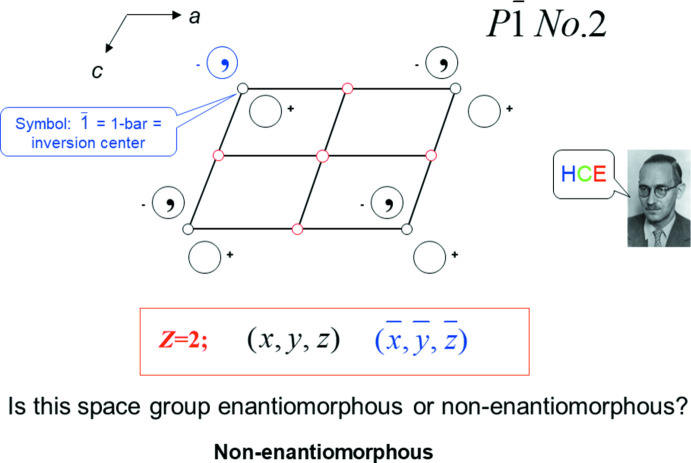
Slide 26, Chapter 1.

For space group *P* 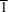, twenty-four steps build up the diagram (Fig. 2 ▸), culminating in a pause while the observer contemplates the answer to the question at the bottom of the page. The picture of Carl Hermann (Fig. 2 ▸; more about *pictures* below) pops up to remind the observer that they must think about **HCE** – **Height**, **Chirality** and the new symmetry **Elements** produced. In section 3.4 you will see how we set up **HCE**. The tutorial user has been prompted to answer the question at the next-to-last line, just before the answer will be revealed by an additional keyclick.

We then moved to Chapters 2, 3 and 4, involving the monoclinic space groups, divided into point groups 2, *m*, and 2/*m*, respectively. Finally, we prepared a fifth Chapter on the ortho­rhom­bic groups, but only those in point group 222. This all looked very nice to us, but there were a few things missing: (*a*) a way to have some give-and-take pedagogy, as best as could be done in PowerPoint (without that, it would just be another online book), and (*b*) valuable historical material, at that point a gaping hole in our developing draft of the tutorial.

### Combining presentation and history   

**Figure 3:**
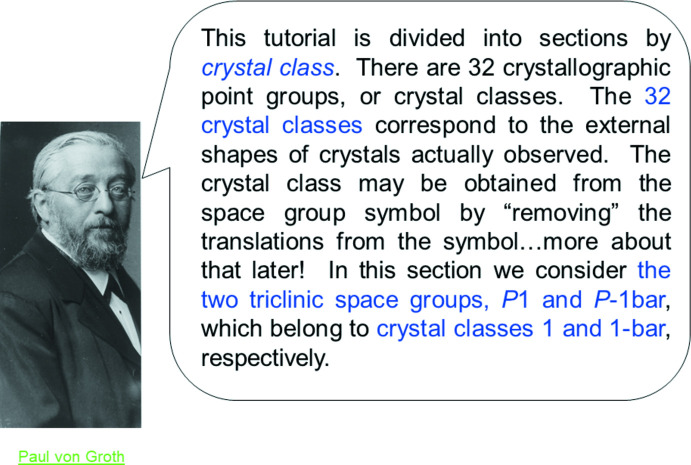
Slide 8, Chapter 1.

**Figure 4:**
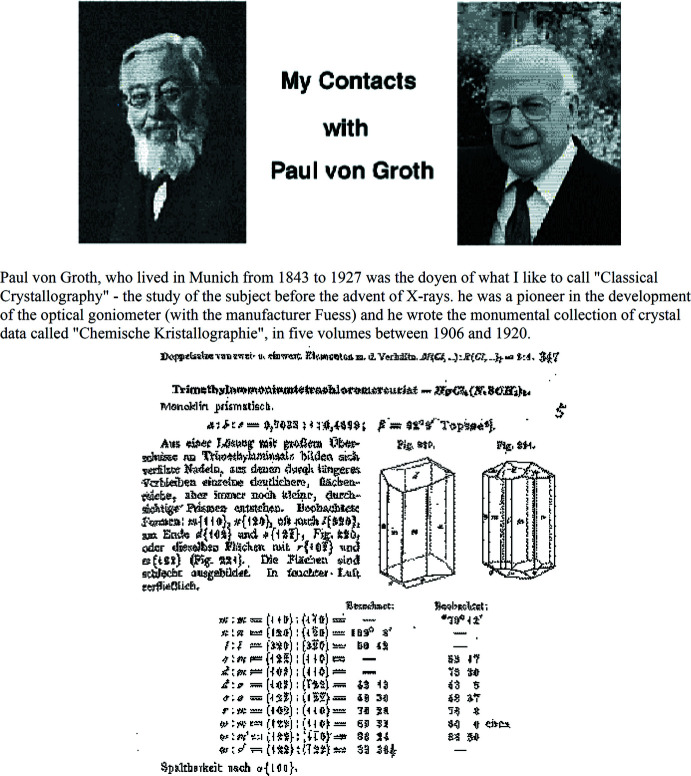
Linked page to Paul von Groth’s Historical Information.

Over a week or two, a few ways to add in that material became apparent. First, explanatory material could be accompanied by a pertinent graphic and name – and the name could be *linked* to a Web site reference. As an example, Fig. 3 ▸ shows a page that explains the layout of the tutorial. If the user clicks on von Groth’s name (in green), a link to the late C. Arnold Beevers’ recollections of von Groth (from the IUCr Web site) appears. An excerpt is shown in Fig. 4 ▸.

### Combining inquiry and history   

**Figure 5:**
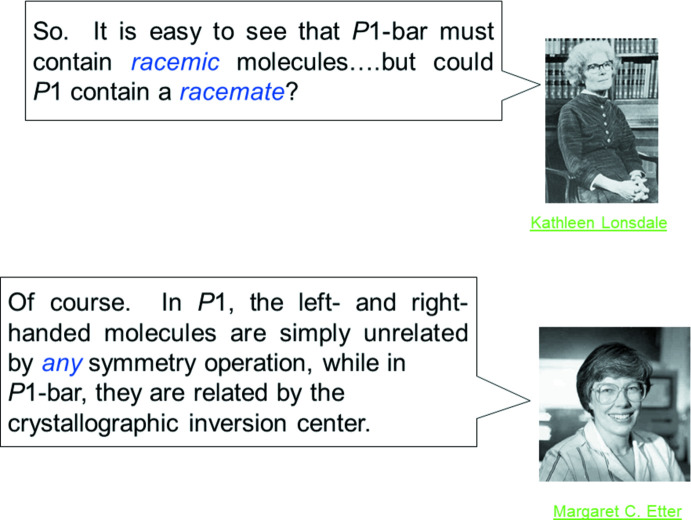
Slide 33, Chapter 1.

For illustrations of pedagogic points, we employed a question-and-answer (Q&A) page with an upper and lower portion. Two famous individuals appear on the screen; the upper person poses a question, and the user can think about it for a time. A keyclick brings the answer onto the screen. Both of the famous personages have links that lead to Web descriptions of their contributions. Fig. 5 ▸ shows a hypothetical dialogue between Kathleen Lonsdale and Peggy Etter. The Great Figures of Crystallography (hereafter, ‘GFCs’) included in the tutorial are all regrettably no longer with us. As we constructed the tutorial and incorporated the GFCs, we felt that it was important for students of the subject to see *images* of, and commentary on, these wonderful people. Not only are the photographs (some of which were very hard to find) valuable historical information, but also they provide an imprint and association for the user to see that these were real people.

### Combining inquiry, history, and professional direction   

**Figure 6:**
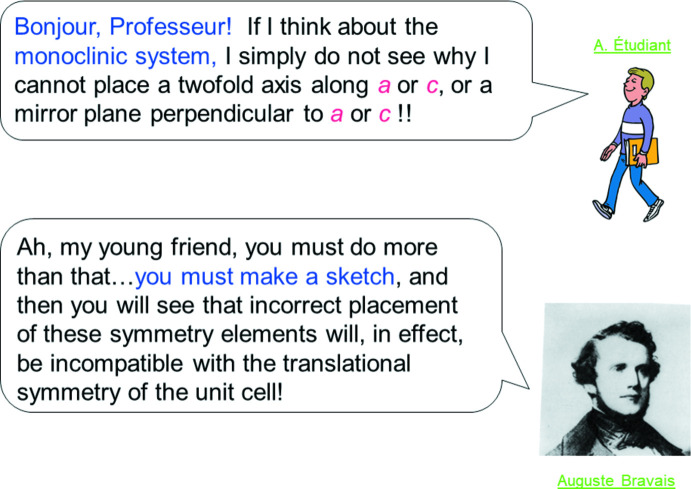
Slide 3, Chapter 2.

**Figure 7:**
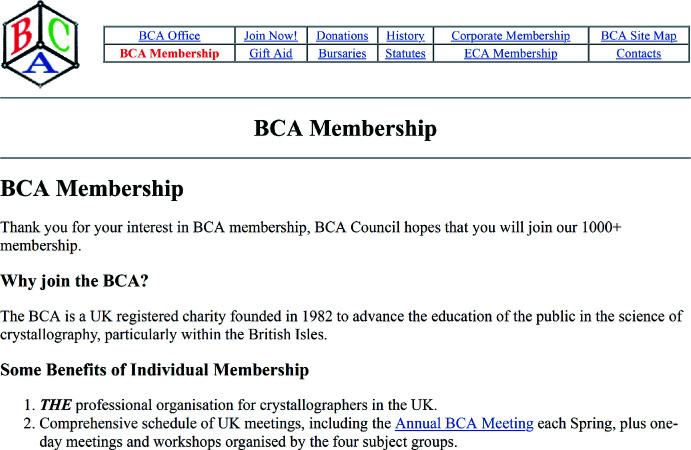
Weblink to Membership in the British Crystallographic Association.

Finally, we wanted to include ‘student images’ in some of the Q&A pages, either ‘alone’ or with one of the GFCs; we found some delightful graphics in our copy of *CorelDraw* 10. An example illustrating the latter approach is shown in Fig. 6 ▸. The ‘student’ is discussing monoclinic symmetry with Professeur Bravais, and after Bravais’ answer, the slides that follow in the tutorial illustrate axial placement in the monoclinic system. The reader of this paper might ask, ‘What happens if I click on A. Étudiant?’. We struggled with this for a time, then realized that there was a GREAT answer: Society Membership, in this particular case, the British Crystallographic Association (BCA)! An excerpt from that link is shown in Fig. 7 ▸. It turns out that this page has moved in the present day, but the idea is ageless, and a student could easily find the new site, and/or the appropriate Society in their home country, using search tools.

**Figure 8:**
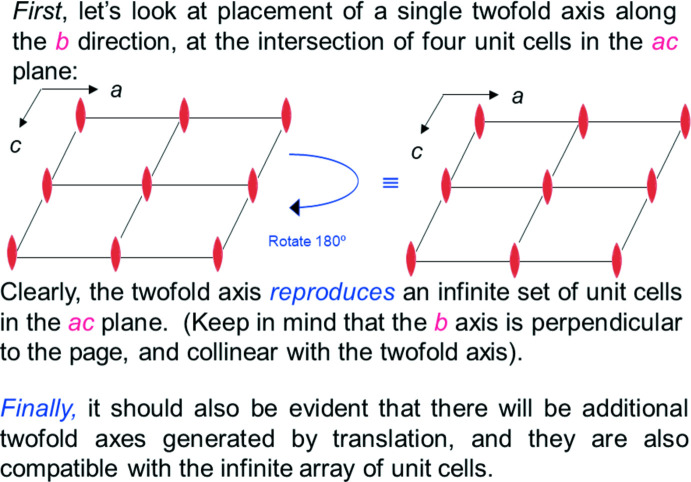
First of two slides illustrating placement of the 2 axis in the monoclinic system (slide 4, Chapter 2).

Let’s illustrate a follow-up to the subject of the question posed in Fig. 6 ▸. Figs. 8 ▸ and 9 ▸ show the monoclinic twofold symmetry applied to a lattice along the *b* axis and *a* axis, respectively, along with explanatory text. This type of illustration appears regularly in the tutorial, and is based on questions that students in our course in crystallography had over the 41 years it was taught at Brandeis University.

### Combining presentation, inquiry and humor   

**Figure 10:**
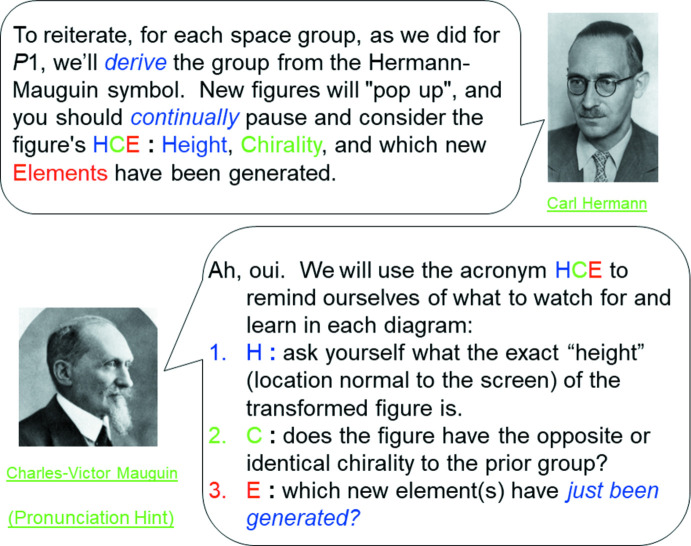
Slide 25, Chapter 1.

**Figure 11:**
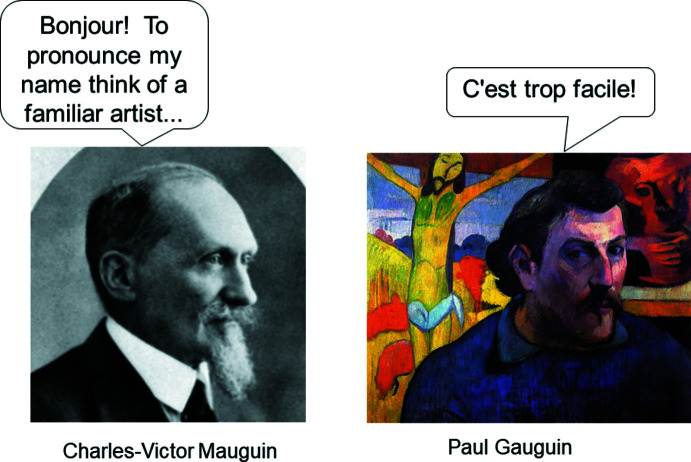
Link to pronunciation slide.

We added the amazing musical treatment of the Bravais lattices from Haverford College, in the style of a Tom Lehrer song, wishing there were more examples of such entertaining ‘Kristallmusik’ of this sort out there (try it now, http://ww3.haverford.edu/physics-astro/songs/bravais.htm or access *via* slide 7, Chapter 1)! Many of the ‘top and bottom’ question/answer pages have, we believe, a nice humorous twist, but our favorite is the pronunciation of Mauguin. This follows a link from slide 25, Chapter 1 (Fig. 10 ▸), where we explain that as a student generates and examines a space-group ‘build’, s/he must think about the new object’s **Height**, **Chirality** and any new symmetry **Elements** that appear. Note the ‘Pronunciation Hint’ in the lower left corner. When that is clicked, we see the answer develops per a set of keyclicks (Fig. 11 ▸).

### Producing a stable presentation   

In order to improve the ‘time-stability’ of the tutorial, we finally decided to embed the Web links in the distributed tutorial. Web addresses change all too frequently, and we were therefore often required to issue updates that contained only Web fixes, distracting both for us and for users. That created yet another problem, since after one embeds the material, the original Web addresses and often the source/authorship become rather muddled. Thus, we added a ‘Credits’ section (as Chapter 7) to the tutorial index that presents the citations to/locations of the *original* Web pages, some of which, of course, are no longer active, but the new locations – if needed and still extant – could be found by a simple search. Alternatively, the originals may be found using the Wayback Machine (https://archive.org/web/), but this should usually not be necessary.

## Final thoughts, conclusions and the future   

**Figure 12:**
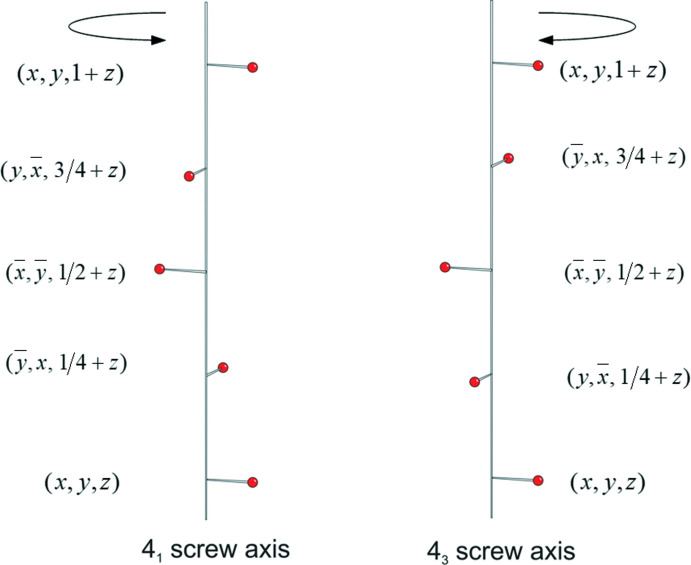
Slide 17, Chapter 4.

At this point, we then decided to add a sixth chapter on enanti­omorphous space groups *P*4_1_ and *P*4_3_, along with an explanation/comparison of right- and left-handed screw axes, a tricky subject for many students. Fig. 12 ▸ is built up in Chapter 6 over an extensive set of slides (4 through 38), an inter­active set building over a large number of keyclicks, and including the derivation of space-group diagrams for both *P*4_1_ and *P*4_3_.

Many more things remain as possibilities to be done: for example, more ortho­rhom­bic groups, more higher-symmetry groups, non-standard settings, particularly *P*2_1_/*n* and *I*2/*a*, and more*.* Other commitments have hindered our ability to add more chapters to the tutorial. Nonetheless, we believe it to be a suitable vehicle for most of the basic principles of space-group use. The tutorial was great fun to write, and the daily registrations still turning up, nearly 20 years since we began, are gratifying. We strongly hope that, if the reader has not yet looked at and downloaded our tutorial, this presentation will prompt a download from: https://peeps.unet.brandeis.edu/~foxman1/teaching/indexpr.html. Comments and suggestions have always been most welcome and highly appreciated. Indeed, offers to write a new Chapter will be even more warmly accepted!

## Remembering Lachlan Cranswick (1968–2010) and Jerry Jasinski (1940–2021)   

**Figure 13:**
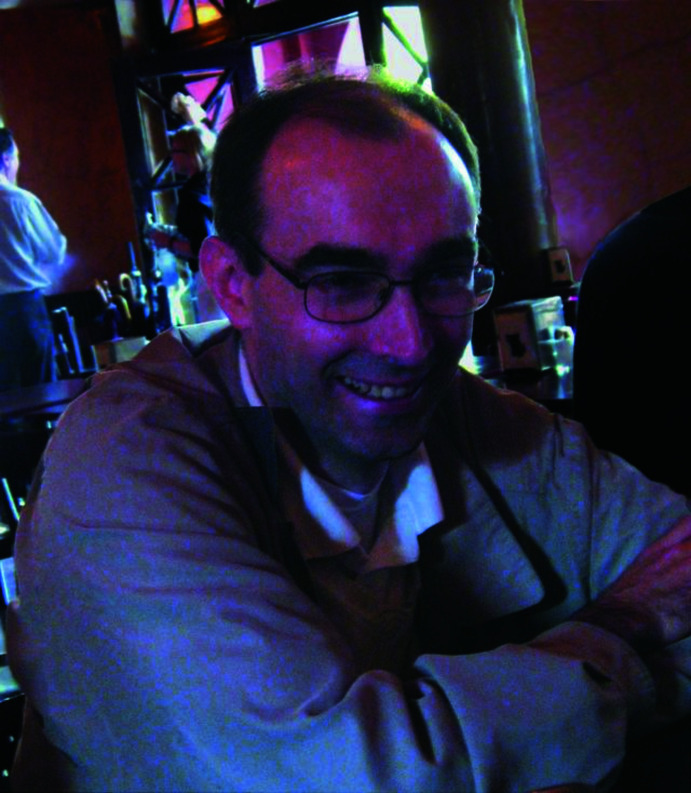
Lachlan Cranswick (1968–2010).

As we developed and then issued our first version of the tutorial, I sought the wisdom of my friends for comments, corrections, suggestions, and so on. One of the most important folks I contacted was Lachlan Cranswick (Fig. 13 ▸). In his short lifetime, Lachlan’s brilliance and generosity (Scarlett *et al.*, 2010 ▸) always illuminated the dark spots in my knowledge. We exchanged a number of emails about the tutorial. Over the years that I knew Lachlan, it was amazing: it did not matter what the difference in time zones happened to be, an answer came back ever so quickly. Later we were in the same time zone. Some of our email exchanges were lost during an ‘upgrade’ of the Brandeis email system. I remember them all as most useful, helpful and very stimulating. One that survived contains the following exchange, edited to improve the drama:

LACHLAN: Lots of people have made space group tutorials.

BRUCE: [gasp ⋯uh⋯gasp]

LACHLAN: But I’ve never seen one done as effectively as this.

BRUCE: [⋯phew⋯smile⋯]

LACHLAN: When are you going to do the rest of the 230 groups?

BRUCE: [more gasping⋯chuckle⋯I have my work cut out for me⋯.]

All of us who knew him still notice his absence; there are many days when I’d like to pick up the phone or send an email.

**Figure 14:**
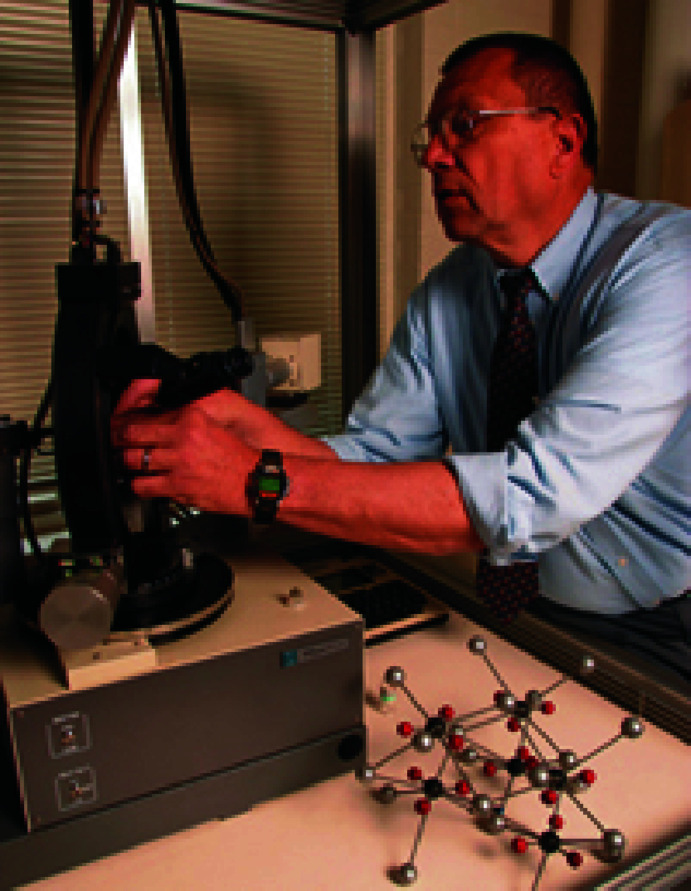
Jerry Jasinski (1940–2021).

Jerry Jasinski’s loss is truly overwhelming. Jerry (Fig. 14 ▸) is remembered as a great teacher, mentor, colleague, friend, and expert in X-ray crystallography. At his undergraduate institution, Keene State College, he published nearly 1,000 papers with collaborators from around the world (Wang, 2021 ▸). When we worked together, Jerry spent each day working enthusiastically and tirelessly to make the tutorial a unique teaching tool. He provided an endless succession of fine ‘space-group artwork’, solid teaching ideas, and great humor. Working with Jerry was a high point in my career. At Brandeis, Jerry’s desk was down the hall, about seven office doors away from mine. As we got new ideas for the tutorial, one of us would rush out of our door, and often we nearly collided with each other. Then followed a recounting of the new idea, and the best part: Jerry’s raucous laughter signaling his approval! At the same time as all this was going on, he rebuilt my garage door, seeing that I was incompetent in such tasks. That was in ’05, and it still works perfectly! I am certain that he is looking over my shoulder, smiling, as I write this story.

## Supplementary Material

Zipped tutorial version for PC. DOI: 10.1107/S2056989021007039/dj2032sup2.zip

Supporting information file. DOI: 10.1107/S2056989021007039/dj2032sup3.zip

## Figures and Tables

**Figure 9:**
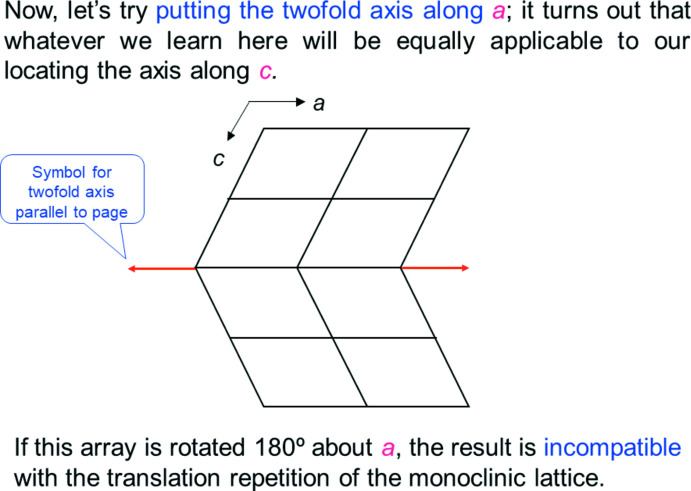
Second of two slides illustrating placement of the 2 axis in the monoclinic system (slide 4, Chapter 2).
