# Supplementary material for: Space groups and crystallographic symmetry: writing a multi-featured tutorial in a new style
Source: Acta Crystallogr E Crystallogr Commun. 2021 Jul 16;77(Pt 9):857–63. doi: 10.1107/S2056989021007039 (PMC8423017; doi:10.1107/S2056989021007039)
Supplement: Supplementary file 1 [file e-77-00857-sup2.zip › symandsg/Main/3DSPGRP.HTM]

Three-Dimensional Space Groups

# Three-Dimensional Space Groups

Steven Dutch, Natural and Applied Sciences, University
of Wisconsin - Green Bay  
First-time Visitors: Please visit Site Map and Disclaimer.
Use "Back" to return here.

---

## Introduction

Start by considering the point groups and lattice types in two dimensions:

| Symmetry | Lattice Type |
| --- | --- |
| 1 | Parallelogram |
| m | Rectangle |
| 2 | Parallelogram |
| 2m = mm | Rectangle, Rhombus |
| 3, 3m | Hexagonal |
| 4, 4m | Square |
| 6, 6m | Hexagonal |

By combining the permissible point groups with their possible lattices, we find 11 of
the 17 plane space groups. The remainder arise when we add *glides* to the patterns.
Not all point groups are compatible with all lattice types. Groups cannot have a lattice
with lower symmetry than that of the space group. A group can have a lattice with higher
symmetry, but nothing new results: putting a 1-fold motif into a square lattice still
results in a 1-fold space pattern, for example.

Similarly, in three dimensions we can combine the 14 Bravais lattices and the 32 point
groups as shown here:

| Crystal Class | Bravais Lattices | Point Groups |
| --- | --- | --- |
| Triclinic | P | 1, 1\* |
| Monoclinic | P, C | 2, m, 2/m |
| Orthorhombic | P, C, F, I | 222, mm2, 2/m 2/m 2/m |
| Trigonal | P, R | 3, 3\*, 32, 3m, 3\*2/m |
| Hexagonal | P | 6, 6\*, 6/m, 622, 6mm, 6\*m2, 6/m 2/m 2/m |
| Tetragonal | P, I | 4, 4\*, 4/m, 422, 4mm, 4\*2m, 4/m 2/m 2/m |
| Isometric | P, F, I | 23, 2/m3\* 432, 4\*3m, 4/m 3\* 2/m |

If we tally up the possible combinations, we get 66. Upon closer inspection, we
discover that a few other variations are possible. For example, we can have 3, 4, or
6-fold symmetry with mirror planes along the edges of the lattice or bisecting its angles.
There are a total of 73 space groups that arise from repeating a motif with one of the
point group symmetries according to the possible Bravais Lattices. These are called the *simple
space groups.*

In three dimensions, there are other possibilities. Not only can there be
glides, but
the orientation of the glide and its direction of translation can vary. In addition, there
is a combination of rotation and translation called a *screw axis*. A screw axis is
just what the name implies: an object rotates and translates along an axis in a helical
pattern. Glides and screw axes raise the total number of all space groups in three
dimensions to 230.

## Symbols

### Rotation Axes

### Mirror and Glide Planes

In all diagrams, the letter R is used as a motif, with
larger letters closer and smaller ones more distant. Outlined R's mean we
are viewing the back side of the motif. Overlapping solid and outlined R's are
used to indicate a motif and its reflection in a mirror plane in the plane of
the diagram.

|  |  |
| --- | --- |
|  | On the left in each diagram is the symbol and appearance of a mirror or glide plane seen edge-on. On the right is the symbol and appearance viewed perpendicular to the plane. Arrows show translation directions. |

**m** is a conventional mirror plane. Objects are reflected across the plane. Looking perpendicular to the plane we see the object and the reflection of its reverse side.

**a, b** are glides parallel to the unit cell edges. We see the object
alternating with its translated reflection.

**c** is a glide parallel to the third edge of the unit cell. Since all
the figures (except isometric classes) view down this direction, there is no view perpendicular to the plane. The
object and its reflection are translated along the line of sight, so we see the object,
then its reflection translated away from us (hence smaller). More distant translations are
hidden behind (beneath) the two images shown.

**n** is a diagonal glide, half a unit cell edge in each direction. In the
view along the plane, additional images would continue to step down and to the left, but
they are hidden behind neare images of the object.

**d** is like **n** in being a diagonal glide, but here the
step is one quarter unit cell edge in each direction. Viewing along the plane we see four
progressively more distant images of the object before the series in the neighboring unit
cell begins.

## The 230 Space Groups

### Trigonal and Monoclinic

Triclinic and Monoclinic (2 and m) Space Groups  
Monoclinic (2/m) Space Groups

### Orthorhombic

Orthorhombic (222) Space Groups  
Orthorhombic (mm) Space Groups  
Orthorhombic (2/m 2/m 2/m) Space Groups (P Lattices)  
Orthorhombic (2/m 2/m 2/m) Space Groups (C, I and F Lattices)

### Tetragonal

Tetragonal (4 and 4\*) Space Groups  
Tetragonal (4/m) Space Groups  
Tetragonal (422) Space Groups  
Tetragonal (4mm) Space Groups  
Tetragonal (4\*2m) Space Groups  
Tetragonal (4/m 2/m 2/m) Space Groups (P Lattices)  
Tetragonal (42/m 2/m 2/m) Space Groups (P Lattices)  
Tetragonal (4/m 2/m 2/m) Space Groups (I Lattices)

### Trigonal

Trigonal (3 and 3\*) Space Groups  
Trigonal (32) Space Groups  
Trigonal (3m) Space Groups  
Trigonal (3\*2m) Space Groups

### Hexagonal

Hexagonal (6) Space Groups  
Hexagonal (6\*, 6\*2m and 6/m) Space Groups  
Hexagonal (622) Space Groups  
Hexagonal (6mm) Space Groups  
Hexagonal (6/m 2/m 2/m) Space Groups

### Isometric

#### 233 (Tetartoidal) Space Groups

P23, F23, I23  
P213, I213

#### 2/m3\* (2m3 - Diploidal) Space Groups

Pm3\*, Pn3\*  
Fm3\*, Fd3\*, Im3\*  
Pa3\*, Ia3\*

#### 432 (Gyroidal) Space Groups

P432, P4232  
F432, F4132, I432  
P4332, P4132, I4132

#### 4\*3m (Tetrahedral) Space Groups

P4\*3m, F4\*3m, I4\*3m  
P4\*3n, F4\*3d, I4\*3d

#### 4/m 3\* 2/m (m3m - Hexoctahedral) Space Groups

Pm3m, Pn3n  
Pm3n, Pn3m  
Fm3m, Fm3c  
Fd3m, Fd3c  
Im3m, Ia3d

---

Return to Symmetry Index  
Return to Crustal Materials (Mineralogy-Petrology) Index

Return to Professor Dutch's Home Page

*Created 30 March 1999, Last Update 11 December 2001*

Not an official UW Green Bay site
