# Supplementary material for: Space groups and crystallographic symmetry: writing a multi-featured tutorial in a new style
Source: Acta Crystallogr E Crystallogr Commun. 2021 Jul 16;77(Pt 9):857–63. doi: 10.1107/S2056989021007039 (PMC8423017; doi:10.1107/S2056989021007039)
Supplement: Supplementary file 1 [file e-77-00857-sup2.zip › symandsg/Main/a26354.pdf]

J. Appl. Cryst. (1986). **19**, 205–207

Obituary

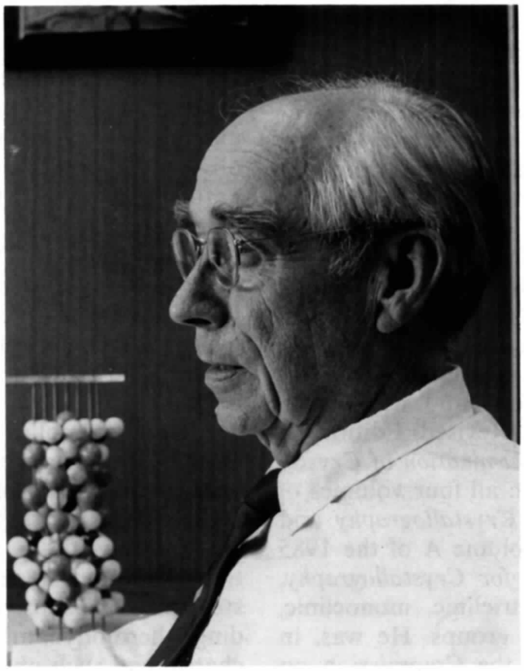

Martin Julian Buerger

1903–1986

M. J. Buerger, the name adorning more than 200 technical papers and one dozen texts and monographs, is without doubt the best known name of any US crystallographer. Is there an X-ray diffraction laboratory (worthy of the name) anywhere in the world that does not have his first book *X-ray Crystallography*? This book, as current today as on the day it appeared in 1942, exemplifies the characteristics distinguishing all Buerger's works: exhaustive completeness and utter thoroughness – enabling a reviewer of a subsequent book to observe that 'this is what we have come to expect of the author'.

Born in Detroit on 8 April 1903, Martin Buerger grew up in New York. He went to MIT to study mining engineering and to continue his graduate studies in mineralogy and ore deposition (PhD in Mineralogy, 1929). He stayed on as Assistant Professor of Mineralogy and Petrology and, following several promotions, was appointed in 1956 as second (after J. C. Slater) Institute Professor at MIT and first Director of its School of Advanced Studies. When forced into half-time retirement in 1968, Buerger accepted an appointment as University Professor of Geology at the University of Connecticut until he 'retired' in 1973 to continue his technical fecundity in offices provided by Harvard, the University of Connecticut and MIT. His editorial chores and other writings did not really cease until Tuesday morning, 25 February 1986, six months after participating in the presentation ceremonies of the first *M. J. Buerger Award* established by the American Crystallographic Association.

The growth and development of crystallography in the United States is intimately interwoven with the professional maturation of M. J. Buerger. When the American Society for X-ray and Electron Diffraction was established in 1941, Buerger was an active organizer and served as its third president (1943). Later, some of its members began to feel that preoccupation with structure determination at society meetings did not adequately represent the full topical range that crystallography encompasses and persuaded Buerger to become the founding President in 1945 of the Crystallographic Society ('of America' was added in 1947). When the Mineralogical Society of America realized what was happening to some of its membership, it sought to repair the damage by electing Buerger as its president in 1947. Finally, when the overlapping memberships of the ASXRED and CSA decided to amalgamate as the ACA, Buerger was once more one of the driving forces.

By mid-century, the significance of Buerger's technical contributions was gaining world-wide recognition. In 1951, the Geological Society of America

0021-8898/86/040205-03$01.50 © 1986 International Union of Crystallography

awarded him its Day Medal; in 1953, he was elected to membership in the US National Academy of Science; in 1958, he received the Roebling Medal of the MSA and an honorary doctorate from the University of Bern. Buerger was a member or corresponding member of the academies of science of Brazil, Torino, Lincei, Bavaria, Austria and Spain and he was honored and fêted in many other lands as well.

Martin Buerger was closely associated with the International Union of Crystallography from the beginning. He was a member of the Provisional International Crystallographic Committee chaired by P. P. Ewald from 1946 to 1948, and continued as a member of the IUCr Executive Committee from 1948 to 1951. He was also a member of the Commission on *International Tables* from its establishment in 1948 until 1981, having earlier taken an active interest in the 1935 edition. His corrections and notes were incorporated in the 1944 US Revised Edition of *International Tables for the Determination of Crystal Structures.* He was involved with all four volumes of *International Tables for X-ray Crystallography* and made many contributions to Volume A of the 1985 edition of *International Tables for Crystallography*, particularly the diagrams for triclinic, monoclinic, orthorhombic and cubic space groups. He was, in addition, an active member of the Commission on Crystallographic Apparatus from its establishment in 1948 until 1957.

It all began after Buerger, then a graduate student, attended the lectures that W. L. Bragg delivered at MIT in 1927. Buerger quickly realized that his efforts at understanding the chemical and physical properties of ore minerals could be advanced significantly by determining their crystal structures using X-ray diffraction. Lacking the requisite facilities and the financial resources to acquire them, he first used those available in a colleague's (B. E. Warren) laboratory and gradually began to assemble his own generators and diffraction cameras. In the process, he made numerous contributions ranging from the design of cylindrical powder and single-crystal cameras having radii equal to $360/n\pi$ centimeters, to the invention of the equi-inclination Weissenberg camera, the precession camera and the equi-inclination (single-crystal) diffractometer. It is noteworthy that, after reading a paper outlining the design of a camera for photographing the undistorted reciprocal lattice, Buerger preempted the authors by building the first functioning de Jong–Bouman camera. He also worked out all the required camera settings, Lorentz–polarization factors, *etc.*, and included them in a separate chapter of his first book.

During the early thirties, Buerger began his long list of crystal-structure determinations but always with an underlying reason for wanting to know the precise atomic arrangement. He often reiterated his belief that the crystal structure had to be known before the properties of a crystal could be understood fully and it was the properties that interested him most. With rare insight, Buerger analyzed the translation gliding in NaCl-type crystals in 1930 in terms that are fully acceptable today despite the increased sophistication afforded by dislocation theory. His continued interest in mineralogical problems led him to examine numerous crystals having varied histories. Based on these observations, Buerger was able to refute the growing notion that crystals had *mosaic* structures patterned after a synthetic (and often misinterpreted) model first proposed by C. G. Darwin to analyze the extinction effect in X-ray diffraction. Instead, Buerger proposed the *lineage theory* of crystal growth which, like some of his other phenomenological speculations on crystal growth, did much to assist the development of that field.

In another application of the growing knowledge of atomic arrays in crystals, Buerger began in 1936 to analyze the causes of polymorphism in crystals. His contributions to this field continued to the end of his life and include a comprehensive classification of transformation types, the concept of derivative structures and substructures, and various ideas regarding thermodynamic relations between structural change and such physical properties as specific heat.

In 1945 Buerger looked into the causes of twinning by crystals. The classification schemes that he proposed remain intact today. The genesis of twinning, like some of his speculations about gliding in ionic minerals, may appear simplistic some forty years later but, in their day, they were radical and challenging in underscoring the insights made possible by a knowledge of the crystal structure.

In the second half of this century, crystallographers began attempting the determination of ever more complicated crystal structures so that new ideas as well as better diffraction instruments were needed. Buerger first addressed this need in the forties by developing his *implication theory* to replace the trial-and-error methods then in common use. Too limited in applicability for widespread acceptance, this theory foreshadowed the utility of inequalities between measured intensities that proved useful in determining their phases. Buerger's subsequent efforts focused on the geometrical relations between the vector sets existing in Patterson space and, by 1951, he published the first generally valid method for determining crystal structures directly from the measured intensity magnitudes. In his inimitable style, Buerger considered all possible ways of 'superposing' Patterson maps, including some being explored independently in England at about the same time, and concluded that his *image-seeking* method or *minimum function* was superior because it eliminated spurious peaks most effectively. Alas, just as the virtues of the de Jong–Bouman

camera failed to be recognized by crystallographers caught up in converting their normal-beam Weissenberg cameras to equi-inclination, the minimum function turned out to be less well suited than alternative methods to the digital computers that were beginning to remove most of the drudgery inherent in crystal structure analyses. Nevertheless Buerger continued to analyze the vectorial relations in crystals, thereby providing many valuable insights. Most of these are recorded in *Vector Sets* and in his authoritative treatise on *Crystal-Structure Analysis*. It is indicative of the man's genius that, while co-authoring a monograph on *The Powder Method in X-ray Crystallography*, he realized the need to identify a reduced cell in an arbitrarily defined lattice and then proceeded to develop three different procedures (graphical, vectorial and algebraic) where one would have sufficed.

Doubtlessly, his most important legacy and one which he, himself, treasured most highly consists of the students and postgraduate associates who had the privilege of working in his laboratories and attending his lectures. A warm and friendly human being, Martin Buerger sought to inspire rather than direct those around him. His personal behavior provided just such an inspiration so that, not surprisingly, more than half his former students and associates have become professors, and, in turn, are assisting their students to continue in this tradition. Through his students, his books and his other publications, M. J. Buerger lives on.

LEONID V. AZÁROFF
Institute of Materials Science
University of Connecticut
