# Supplementary material for: Space groups and crystallographic symmetry: writing a multi-featured tutorial in a new style
Source: Acta Crystallogr E Crystallogr Commun. 2021 Jul 16;77(Pt 9):857–63. doi: 10.1107/S2056989021007039 (PMC8423017; doi:10.1107/S2056989021007039)
Supplement: Supplementary file 1 [file e-77-00857-sup2.zip › symandsg/Main/abs1247.html]

JCE 2000 (77) 1247 [Sep] Crystallographic CourseWare

|  |  |
| --- | --- |
|  |  |
|  | \| Subscriptions  \| Software Orders  \| Support  \| Contributors  \| Advertisers  \| |
|  | |

|  |  |  |  |  |  |  |  |  |  |  |  |  |  |  |  |  |  |  |  |  |  |  |  |  |  |  |  |  |  |  |  |  |  |  |  |  |  |  |  |  |  |  |  |  |  |  |  |  |  |  |  |  |  |  |  |  |  |  |  |  |  |  |  |  |  |  |  |  |  |  |  |  |  |  |  |  |  |  |  |  |  |  |  |  |  |  |
| --- | --- | --- | --- | --- | --- | --- | --- | --- | --- | --- | --- | --- | --- | --- | --- | --- | --- | --- | --- | --- | --- | --- | --- | --- | --- | --- | --- | --- | --- | --- | --- | --- | --- | --- | --- | --- | --- | --- | --- | --- | --- | --- | --- | --- | --- | --- | --- | --- | --- | --- | --- | --- | --- | --- | --- | --- | --- | --- | --- | --- | --- | --- | --- | --- | --- | --- | --- | --- | --- | --- | --- | --- | --- | --- | --- | --- | --- | --- | --- | --- | --- | --- | --- | --- | --- | --- |
| |  | | --- | | *JCE* Print | | |  |  | | --- | --- | |  | Current Issue   Previous Issues   Supplements   Search *JCE* Index | |      |  | | --- | | *JCE* Digital Library | | |  |  | | --- | --- | |  | DigiDemos   QBank   SymMath   WebWare | |      |  | | --- | | *JCE Software* | | |  |  | | --- | --- | |  | Latest Releases   Software & Video   Downloads   Support | |      |  | | --- | | Only@JCE Online | | |  |  | | --- | --- | |  | *JCE* Online Store   *JCE* HS CLIC   *JCE* Discussion Forums | | | |  |  | | --- | --- | |  | Biographical Snapshots   ChemEd Resource Shelf   Featured Molecules   Hal's Picks   Project Chemlab   Reviewed WWW Sites   "Web-Ed" Articles | |      |  | | --- | | About *JCE* | | |  |  | | --- | --- | |  | Features   Publications   Operations   Outreach   Contact Us | | |  | |  | | --- | | Home > JCE Print > Journal of Chemical Education > Issues >  2000  >  September  > | | |  |  |  |  |  |  |  |  |  |  |  | | --- | --- | --- | --- | --- | --- | --- | --- | --- | --- | --- | | |  | | --- | | Information • Textbooks • Media • Resources | | **JCE* Software* | | Crystallographic CourseWare | | **Margaret E. Kastner, Eric Vasbinder, Deborah Kowalcyzk, Sean Jackson, Joseph Giammalvo, James Braun, and Keith DiMarco**  Department of Chemistry, Bucknell University, Lewisburg, PA 17837    |  | | --- | |  | | | |  |  |  |  | | --- | --- | --- | --- | | |  |  |  | | --- | --- | --- | | **September 2000 Vol. 77 No. 9 p. 1247**   |  |  | | --- | --- | | |  | | --- | |  | |   Table of Contents  Supplements in This Issue    Previous Article    Next Article | | | | | |  | | --- | |  | |  | | Full Text (PDF) |  |  | | --- | | Full Text | | Crystallographic CourseWare is a series of modules for Mac OS and Windows designed to teach fundamental concepts in crystallography. The complete package of instructional programs and exercises is intended for use in an upper-division undergraduate or graduate special-topics course. Individual units can be used in general, inorganic, or physical chemistry courses.  **Contents**  Crystallographic CourseWare contains 12 modules.   1. **Crystal Growth** includes text and animation describing how to grow crystallographic-quality crystals. 2. **Symmetry**    provides definitions, illustrations, and animations of symmetry    elements and operations typically observed in triclinic, monoclinic, or    orthorhombic unit cells. 3. **More Symmetry** extends the information in Symmetry to include less common symmetry such as diamond glides, and 3*n* and 6*n* screws; includes a comparison of *S*6 and 3-bar symmetry operations. 4. **Unit Cells and Asymmetric Units** has definitions, illustrations, and animations including both plane group and space group lattices. 5. **Exercises in Plane Group Symmetry**    provides a simple drawing toolbox for students to locate and identify    the location of plane group symmetry elements, the unit cell, and    asymmetric unit for a simple motif illustrating each of the 17 plane    groups; additional exercises include motifs located at special    positions. Answers are available on-screen for all exercises, with    animations detailing how the answer is found in the pattern for some.    Two of the animations extend the instruction to introduce the standard    symbols used in the *International Tables for Crystallography* (*1*) and general positions. 6. **Exercises in Reading the *International Tables for Crystallography*** provides a simple drawing toolbox for students to build an understanding of the relationships between general positions (*x*, *y*, *z*), symmetry elements, and general position projections as presented in the *International Tables for Crystallography*. Some examples have answers included, others can be found in the *Brief Teaching Edition of the International Tables for Crystallography* (*2*), and the rest require access to the complete *Tables* and thus can be used as quiz materials. 7. **Reciprocal Space**    provides a step-by-step illustration of the relationship between real    and reciprocal lattices, with an introduction to systematic absences as    might be seen in precession photographs or computer-generated images of    the reciprocal lattice. 8. **Precession Photographs**    provides instruction with simple exercises for the indexing of    precession photographs and further instruction on how to identify    systematic absences and use that information to identify possible space    groups. 9. **Scattering Factors** contains interactive    programs to allow users to observe the change in scattering power as a    function of the angle of scattering, the radiation source used, the    atoms causing the scattering, and thermal motion. 10. **Thermal Parameters**     provides instruction to allow recognition of well- or poorly-behaved     molecules by looking at the thermal ellipsoid plots. The Windows     version also includes interactive programs for students to explore the     relationship between the algebraic and graphical representations of     ellipses. 11. **Fractional Crystal Coordinates** (*Windows only*)     provides interactive exercises to assist students in learning how the     fractional crystal coordinates relate to the position of molecules in     the unit cell, how the general positions correspond to the placement of     replicate molecules within the cell, and how the general position     projection diagrams as given in the *International Tables for Crystallography* (*1*) would change in appearance if the magnitude of *x*, *y*, and *z* were changed. 12. **Special Positions** (*Windows only*)     provides interactive exercises requiring the user to identify all of     the point group symmetry elements in a species, identify the point     group using a typical flow diagram, and identify the possible special     position locations of molecules using the symmetry projection diagrams     as provided in the *International Tables* (*2*).   **How to Use Crystallographic CourseWare**  Crystallographic CourseWare has been used in a student-centered (*3*) instructional setting, with students using the programs and the instructor available to help students as required; and for independent study, with the instructor meeting with the students between units to clarify material as needed. Individual units can be employed in other settings. For example, the instructional units on two-dimensional symmetry, unit cells, and asymmetric units and the related exercises can be used in a general chemistry course, particularly for engineering students who will subsequently be taking a materials science course. The unit on crystal growth can be effectively used by students doing synthetic research where one goal is the growth of crystallographic-quality crystals. The units on reciprocal space and precession photography could be used in physical chemistry courses. Although designed for individual or group student use, some units could be projected for use in lecture courses.  Well-prepared and motivated students can use Crystallographic CourseWare together with a standard text to make substantial progress learning crystallography in an independent-study course. The programs do not eliminate the need for the instructor, but they do allow the time spent one-on-one to be directed at clarification of issues rather than basic instruction. A syllabus used by the instructor for students doing a "half-course" or a "full-course" using a combination of these programs, a standard text, live demonstrations, and other exercises is provided in the documentation.  **Hardware and Software Requirements**  Hardware and software requirements for Crystallographic CourseWare are found in Table 2. Crystallographic CourseWare was created using Macromedia Director (*4*), QuickTime (*5*), Toolbook (*6*) (for the Windows version), and HyperCard (*7*) (for the Mac OS version).  **Table 2. Hardware and Software Required for Crystallographic CourseWare**  **Acknowledgments** Crystallographic CourseWare has been in production for ten years. Various parts of it have been class tested as described in the "How to Use" section above and the patience and comments of those students is appreciated. Also greatly appreciated are the comments of peer reviewers of the software, both formal and informal.  The HyperCard version and related QuickTime movies were produced with support from the Pew Charitable Trusts as administered through the Mid-Atlantic Cluster (1991-1992). The ToolBook version and cross-platform Macromedia Director units were produced with support by the Camille and Henry Dreyfus Foundation, Inc. (1997-1999).  **Literature Cited**   1. *International Tables for Crystallography: Volume A: Space Group Symmetry*; Hanh, T., Ed.; D. Reidel: Boston, 1983. 2. *International Tables for Crystallography, Brief Teaching Edition of Volume A, Space-Group Symmetry*, 3rd ed.; Hanh, T., Ed.; Dordrecht: Boston, 1993. 3. Kastner, M. E. *J. Appl. Crystallogr.* **1999**, *32*, 327-331. 4. Macromedia Director, version 6.5; Macromedia, Inc., San Francisco, CA; 1998. 5. QuickTime, version 3.0; Apple Computer: Cupertino, CA, 1998. 6. ToolBook II, Instructor, version 6.0; Asymetrix: Bellevue, WA, 1998. 7. HyperCard 2.3.5; Apple Computer: Cupertino, CA, 1998. | | | |  |  |  | | --- | --- | --- | | More Information | | | | Citation | Kastner, Margaret E.; Vasbinder, Eric; Kowalcyzk, Deborah; Jackson, Sean; Giammalvo, Joseph; Braun, James; DiMarco, Keith. *J. Chem. Educ.* **2000**  *77* 1247. | | | Keywords | Computer Assisted Instruction; Crystallography / Crystal Growth; Physical Chemistry | | | History | Created:   Last Updated: | August 29, 2000    June 22, 2005 | |  |  | | | | |  |  |  | | --- | --- | --- | |  | | | | << Previous Article | Table of Contents | Next Article >> | | |  | | Home > JCE Print > Journal of Chemical Education > Issues >  2000  >  September | |  | |  | | --- | | Subscriptions | |  | | Subscription Info   Order Forms |      |  | | --- | | JCE HS CLIC | | Our Secondary School editors work hard to distill all the *JCE* materials to produce a fraction of particular interest to high school teachers. We call it CLIC. | | JCE HS CLIC |      |  | | --- | | Contributions Welcome | | *JCE* welcomes your submission | | Contributors' Corner |      |  | | --- | | Advertisers | | In recent years we have worked hard to better match our advertisers with our readers. When shopping for chemistry education materials, visit our advertisers' WWW sites first. | | Recent Advertisers |      |  | | --- | | Be An Ambassador | | Take *JCE* along on your outreach missions. Copies of the *Journal*, guest access to JCE Online, our publications catalog, and more are available for your participants. | | Outreach with *JCE* | |


|  |  |  |
| --- | --- | --- |
|  | | |
| Comments to  jceonline@chem.wisc.edu | Copyright © Division of Chemical Education, Inc., American Chemical Society.  All rights reserved. |  |
