# Supplementary material for: Space groups and crystallographic symmetry: writing a multi-featured tutorial in a new style
Source: Acta Crystallogr E Crystallogr Commun. 2021 Jul 16;77(Pt 9):857–63. doi: 10.1107/S2056989021007039 (PMC8423017; doi:10.1107/S2056989021007039)
Supplement: Supplementary file 1 [file e-77-00857-sup2.zip › symandsg/Main/abso_files/sh0129bdy.htm]

(IUCr) Absolute structure and absolute configuration

## research papers

---

|  |  |
| --- | --- |
| **Volume 55**   **Part 5**    Pages 908-915    September 1999    Received 13 January 1999  Accepted 19 March 1999  © International Union of Crystallography 1999 | Absolute structure and absolute configuration **H. D. Flacka\* and G. Bernardinellia**  **a**Laboratoire de Cristallographie, University of Geneva, 24 quai Ernest Ansermet, CH-1211 Genève 4, Switzerland Correspondence e-mail: howard.flack@cryst.unige.ch  Fundamental notions concerning absolute structure and absolute configuration, and their determination from single-crystal diffraction measurements, are presented and reviewed. A glossary of terms with definitions useful in this field is provided. For absolute structure and its determination, the separate but interacting influences of the structure and the inversion-distinguishing power of an X-ray diffraction experiment with dispersive scatterers are examined. Important experimental and algorithmic details of the current methods used for absolute-structure determination are provided. Characterization of crystals for absolute-structure determination and of molecules for absolute-configuration determination are treated. Attention is given to the analysis of absolute structure and absolute configuration in twinned crystals.  **Keywords: absolute configuration; twinning.** |

### 1. Glossary of terms

|  |
| --- |
| - *Absolute configuration*.   The spatial arrangement of the atoms of a physically identified chiral   molecular entity (or group) and its stereochemical description (*e.g. R* or *S*, *P* or *M*, *D* or *L* *etc*.). - *Absolute structure*.   The spatial arrangement of the atoms of a physically identified   noncentrosymmetric crystal and its description by way of unit-cell   dimensions, space group and representative coordinates of all atoms. - *Chiral*. Having the property of chirality (Moss, 1996). - *Chirality*.   The geometric property of a rigid object (or spatial arrangement of   points or atoms) of being nonsuperposable on its mirror image; such an   object has no symmetry operations of the second kind (a mirror plane, *m*; a centre of inversion, 1; a roto-inversion axis, *N* ). If the object is superposable on its mirror image, the object is described as being achiral [Moss (1996), modified for H-M symbols]. - *Chirality sense*.   The property that distinguishes enantiomorphs. The specification of two   enantiomorphic forms by reference to an oriented space, *e.g.* of a screw, a right-threaded one or a left-threaded one. The expression *opposite chirality* is short for *opposite chirality sense* (Moss, 1996). - *Enantiomer*. One of a pair of molecular entities which are mirror images of each other and nonsuperposable (Moss, 1996). - *Enantiomerically pure/enantiopure*.   A sample in which all molecules have (within the limits of detection)   the same chirality sense. Use of homochiral as a synonym is strongly   discouraged (Moss, 1996). - *Enantiomorph*. One of a pair of chiral objects or models that are nonsuperposable mirror images of each other (Moss, 1996). - *Flack* (1983) *parameter*. The parameter *x* in the structure-amplitude equation    (Flack, 1983). - *Inversion twin*.   An inversion twin consists of centrosymmetrically related crystalline   domains. The symmetry operation relating domain structures in an   inversion twin is that of a centre of symmetry. - *Racemate*. An   equimolar mixture of a pair of enantiomers. It does not exhibit optical   activity. The chemical name or formula of a racemate is distinguished   from those of the enantiomers by the prefix (±) or *rac*- (or *racem*-) or by the symbols *RS* and *SR* (Moss, 1996). - *Racemic*. Pertaining to a racemate (Moss, 1996). - *Racemic compound*.   A crystalline racemate in which the two enantiomers are present in   equal amounts in a well defined arrangement within the lattice of a   homogeneous crystalline addition compound (Moss, 1996). - *Racemic conglomerate*.   An equimolar mechanical mixture of crystals, each one of which contains   only one of the two enantiomers present in a racemate. The process of   its formation on crystallization of a racemate is called *spontaneous resolution*, since pure or nearly pure enantiomers can often be obtained from the conglomerate by sorting (Moss, 1996). |

### 2. Introduction

> *...la dissolution laisse déposer, après quelques
> jours, des cristaux qui ont tous exactement les mêmes angles, le même
> aspect; et pourtant, à coup sûr, l'arrangement moléculaire dans les uns
> et les autres est tout à fait différent*. (Pasteur, 1848.)

*Absolute structure* is a crystallographer's term and applies to noncentrosymmetric crystal structures. *Absolute configuration* is a chemist's term and refers to *chiral* molecules. Note particularly that both the entity under consideration, *viz* crystal structure *versus* molecule, and the symmetry restrictions, *viz* noncentrosymmetric *versus*
lack of mirrors, centres of symmetry and roto-inversions, are
different. Both terms concern the complete specification of the spatial
arrangement of atoms with respect to inversion. Discussion and
justification of our choice of definition of these two terms will be
deferred until a later section. As the word *absolute*
appears as a qualifier, an impression is generated that a complete,
justified, invariant and transferable specification of the spatial
arrangement of the atoms is being made available. In practice, such
desirable conditions may not have been achieved.

Structure analysis and information technology have advanced greatly since other authors (Glazer & Stadnicka, 1989; Jones, 1984*a*,*b*, 1986*b*; Rogers, 1975, 1981) have warned about the misuse and misunderstanding of the terms *absolute structure* and *absolute configuration* and related concepts. The advent of the Crystallographic Information File, CIF (Hall *et al.*, 1991),
has created the requirement for the clear definition of data items
concerned with the reporting of absolute structure and absolute
configuration and permits their machine validation. Modern
single-crystal structure-analysis systems use the Flack parameter
(Flack, 1983; Bernardinelli & Flack, 1985, 1987)
to estimate absolute structure, a method which was in its infancy at
the time of the previous reports. Moreover, the availability of area
detectors and synchrotron-radiation sources offers improved
opportunities for data collection, which have made the determination of
absolute structure more widely applicable than previously.

The
current paper presents the fundamental notions associated with the
determination and reporting of absolute structure and absolute
configuration from crystal structure analysis. The topics treated have
been chosen on the basis of private communications with the authors and
of a comprehensive study of all papers in *Acta Crystallographica Section C*, Vol. 52 (1996). The latter will be presented in detail in a future paper (Flack & Bernardinelli, 1999).
The overall objective is to identify shortcomings in present practice
and to provide a basis for improvement. To this effect, we provide a
glossary of important terms in this field, have negotiated new or
improved definitions for CIF data names and, with the further paper,
provide a checklist in algorithmic form for use by publishers of
crystal-structure journals.

The terms in the glossary (§1)
related to absolute structure and absolute configuration have been
gathered together with the needs of the principal user groups in mind, *viz*
structure analysts and chemists. The terms chosen for inclusion are
those that have been used and adhered to in the present article and the
definitions are thought to be the clearest available corresponding to
current usage. Their source has been cited if not original to the
present paper. Of particular interest was *Basic Terminology of Stereochemistry*, *IUPAC Recommendations 1996* (Moss, 1996). No discussion is presented of terms not included in the list.

### 3. Absolute structure

#### 3.1. Inversion-distinguishing power

The central problem in absolute-structure determination is the
capacity to distinguish between an image of the crystal structure and
that of a centrosymmetrically related one. A successful structure
analysis on a crystal with a noncentrosymmetric structure will always
produce a list of atomic coordinates corresponding to a single
noncentrosymmetric spatial arrangement of atoms in the crystal, whether
or not the data are capable of distinguishing between this arrangement
and its inverse. Where the inversion-distinguishing power is low, the
result is in reality a choice between two centrosymmetrically related
images, although only one image of the structure is presented. One may
say that the structure determination is ambiguous. On the other hand,
where the inversion-distinguishing power is sufficiently high, one may
assert that the model and the crystal as mounted on the diffractometer
correspond one to another. The structure determination is potentially
absolute.

#### 3.2. Right-handed axes

As emphasized and discussed by Rogers (1975),
right-handed sets of axes must be used at every stage of an analysis of
absolute structure. Of particular danger for the structure analyst are
basis transformations performed to bring the unit cell into a standard
setting.

#### 3.3. Inversion twins

Crystals may be twinned by growth or phase transformation. Of
relevance to the study of absolute structure and absolute configuration
is the occurrence of inversion twins. Twinning by inversion is
characteristic of crystal structures displaying a noncentrosymmetric
space group. In an inversion twin, the crystal lattice (*i.e.*
the lattice translations after removing the atoms) is maintained
throughout the whole volume of the sample, but the atoms and molecules
take up either one spatial arrangement or the inverted one depending on
the position within the crystal. A visual model of an inversion twin,
applicable to chiral crystal structures, is to imagine the individual
components of a *racemic conglomerate*
being stuck together with their lattices being perfectly oriented. The
inversion-twinned crystal is an oriented solid-state mixture of
inverted structures. Inversion-twinned crystals do not form from an *enantiopure* sample of a substance.

### 4. Determination of absolute structure by X-ray diffraction with dispersive scatterers

We owe to Coster *et al.* (1930)
the first demonstration that X-ray diffraction using anomalous
scattering can distinguish a noncentrosymmetric crystal structure from
its inverted image. Previous to this experiment, the
inversion-distinguishing power of X-ray diffraction was considered to
be zero as expressed in Friedel's law. X-ray anomalous scattering is a
second-order effect, making its inversion-distinguishing power small
but nevertheless significant. By comparison, optical systems using
visible light and lenses have a very high inversion-distinguishing
power. The presence of significant dispersive scatterers in a
noncentrosymmetric crystal structure leads to the intensity
distribution in the X-ray diffraction pattern revealing the true point
symmetry of the structure (crystal class), rather than that of its
supergroup containing a centre of symmetry (Laue symmetry). Modern
technology is such that the observation of intensity differences
between Friedel opposites (*hkl* and *h* *k* *l*
) is now the rule rather than the exception. It follows that, with the
capacity to produce data displaying the symmetry of the crystal class,
it is now routinely possible to distinguish a noncentrosymmetric
structure from its inverse, thus opening the way to the determination
of absolute structure by X-ray diffraction from a single crystal.

An
advantage of distinguishing a noncentrosymmetric structure from its
inverse in the refinement of a crystal structure is the more realistic
modelling that it affords, with the consequent improvement in
statistical measures of fit. The capacity of a refinement to adapt to
an inverted model can be startling for a crystal structure in a space
group in which the origin cannot be fixed with respect to the symmetry
elements of the space group (point groups: 1, 2, *m*, *mm*2, 4, 4*mm*, 3, 3*m*, 6, 6*mm*). Under the conditions leading to a polar dispersion error (Ueki *et al.*, 1966; Cruickshank & McDonald, 1967),
displacement of the apparent position of the atoms along the free
directions will compensate for any mistake in the absolute structure or
anomalous-dispersion contribution. Thus the need to avoid systematic
errors, which may arise in the atomic positional coordinates and bond
lengths when a polar dispersion error occurs, turns realistic modelling
of the inversion possibilities from an advantage into a necessity. It
is also well established (Jones *et al.*, 1988) that an inverted structure model gives rise to false, although plausible, ghost atoms in an electron-density map.

The
main routine method for distinguishing a noncentrosymmetric structure
from its inverse when anomalous scattering is significant is use of the
Flack (1983) parameter. The most common alternative to this is Hamilton's (1965) *R*-factor-ratio test.

#### 4.1. Flack (1983) parameter

In essence, any noncentrosymmetric crystal is treated as being a
twin by inversion and the fractional contributions of the twin
components are considered as variable during the least-squares
refinement of the crystal structure. The underlying squared
structure-amplitude equation is

When *x* takes a value of zero, the atomic arrangement of the model and the crystal are identical; when *x*
takes a value of one, the atomic arrangement of the model and that of
the crystal are inverted one with respect to the other. For a twinned
crystal, *x* gives a measure of the
relative amounts of the structure and its inverse in the crystal. Full
details of the use and advantages of this approach have been given by
Flack (1983), Bernardinelli & Flack (1985, 1987) and Flack & Schwarzenbach (1988). The reciprocal of the standard uncertainty of *x* is a direct measure of the inversion-distinguishing power of the diffraction measurement, while *x*
itself is a way of indicating what has been distinguished. Under
conditions where a polar dispersion error may occur, one witnesses
large correlations between the Flack (1983) parameter and the atomic coordinates along the origin-free directions.

The implementation of the Flack parameter in the widely used refinement program *SHELXL*93/*SHELXL*97 (Sheldrick, 1993/1997) uses a special algorithm requiring care in its interpretation. Details will be given by Flack & Bernardinelli (1999).

#### 4.2. Hamilton's (1965) *R*-factor-ratio test

As applied to absolute-structure determination, Hamilton's (1965) test was subject to a very critical analysis by Rogers (1981), casting doubt on its fundamental validity in this case. This work gave rise to a flurry of activity (Jones, 1984*a*,*b*, 1986*a*,*b*; Jones & Meyer-Bäse, 1987; Flack, 1983; Bernardinelli & Flack, 1985, 1987; Glazer & Stadnicka, 1989) and the emergence of the Flack (1983) parameter. The basis of Hamilton's method is the comparison of the conventional or weighted *R* factors from two models of the structure, *i.e.* the refined model and its inverse. The study by Flack & Bernardinelli (1999)
of published structures shows that Hamilton's test is almost always
being applied incorrectly in current practice. It is worth mentioning
yet again (Flack, 1983; Bernardinelli & Flack, 1985) that the Hamilton test cannot take account of the effects of inversion twinning. *R*
values that are judged to be insignificantly different by Hamilton's
test may represent situations where either the inversion-distinguishing
power is too low or where the crystal is twinned by inversion.

#### 4.3. Enhancing inversion-distinguishing power

It is most instructive to examine the conditions controlling the
inversion-distinguishing power in an X-ray diffraction experiment with
dispersive scatterers. This will be studied by a simple model. Let the
nondispersive light atoms make a contribution **F***L*(**h**) to the structure factor **F**(**h**) of

obtained by summing over all light atoms in the unit cell. Let the
dispersive heavy atoms be all of the same kind and let their
contribution **F***H*(**h**) (obtained by summation over all heavy atoms in the unit cell) to **F**(**h**) be

Then

and the difference in intensity between Friedel opposites

The inversion-distinguishing power depends on the values of  for all reflection pairs in a data set and a rough measure is obtained from

<||> will increase as the values of  increase and it will be zero when  is systematically zero over all reflections. This occurs when (*a*) *L* = 0, *i.e.*
all atoms in the unit cell are dispersive but of the same kind as
occurs in the structure of a chemical element even if
noncentrosymmetric, (*b*) , *i.e.* there are no dispersive scatterers as occurs in the application of Friedel's law, and (*c*)  as occurs in centrosymmetric structures regardless of the choice of origin. The conditions that lead to a large value of <||> are evident. Both <*L*> and <*H*´´> must be as large as possible, *i.e.* there must be sizeable nondispersive and dispersive contributions in the unit cell. <*H*´´> may be increased by a change of X-ray wavelength. In general, the trigonometric term in <||> will take a nonzero value but it will tend to zero if *L* and *H*
are strongly correlated, as occurs if both the light- and the
heavy-atom substructures are centrosymmetric or close to it. On the
other hand, if the light-atom substructure is clearly
noncentrosymmetric, the heavy-atom substructure may be (pseudo-)
centrosymmetric or contain some other (pseudo-) symmetry operation of
the second kind. In other words, the inversion-distinguishing power of
an X-ray diffraction experiment is increased by the inclusion of
dispersive scatterers, there being very few restrictions on the
position of the dispersive atoms within the crystal structure. A simple
application of this effect is to cocrystallize the compound
investigated with a molecule such as CCl4.
In such a way, an achiral dispersive molecule may be used in the
determination of the absolute configuration of a chiral nondispersive
molecule.

#### 4.4. Intensity data

It is commonly admitted, albeit reluctantly, that in a satisfactory
raw intensity data set there should be at least one measurement from
each set of symmetry-equivalent reflections out to the resolution limit
chosen for the study. From this raw set, a reduced data set of unique
reflections is produced by averaging symmetry-equivalent reflections.
For the purposes of structure refinement and absolute-structure
determination of a noncentrosymmetric crystal structure, the correct
symmetry to use for the averaging process is that of the crystal class,
in which, of course, *h* *k* *l* is not symmetry-equivalent to *hkl*.
Use of a symmetry other than that of the crystal class, for example the
Laue symmetry, needs to be justified in each particular case. In
principle, there is an advantage to keeping *hkl* and *h* *k* *l*
separate, even if the absolute structure of the crystal is known at the
outset. To demonstrate this advantage, consider the half-sum, *S*, and the half-difference, *D*, of the squared model structure amplitudes dependent on *x*, the Flack (1983) parameter:

and

*S* is independent of *x* and thus contains only structural information corresponding to a 50% inversion-twin model. *D* may be systematically zero over a whole set of data, in which case *D* evidently contains no structural information. This occurs either when *x* = 1/2, an equivolume inversion twin, or when , a situation studied in the previous paragraph. With *D* not systematically zero, it is not possible to write *D* as a function of *x* and *S* only. Thus, from the functional form of *D*,
one sees that it contains both information on the distinguishability of
the structure from its inverse and structural information supplementary
to that contained in *S*. The information
content of crystal-class averaged data is higher or at least equal to
that of Laue-symmetry averaged data. An informal measure of the excess
structural information in crystal-class averaged data is afforded by
its capacity to phase reflections in single or multiple
anomalous-scattering techniques or to define the Flack (1983) parameter during refinement.

### 5. Determination of absolute structure by X-ray diffraction using an internal chiral reference

The presence in a crystal structure of enantiopure chiral molecules,
groups or chiral centres of known absolute configuration leads directly
to the determination of the absolute structure of the whole crystal by
making the image of the atomic arrangement correspond to that of the
known chiral molecule. The chiral molecules (or groups or centres) thus
act as an internal reference. It may be introduced as part of the
crystal by chemical reaction or cocrystallization using an enantiopure
sample of the reference substance. The internal-reference technique is
necessarily limited to chiral crystal structures due to the
enantiopurity of the reference substance. By using an internal chiral
reference, the determination of the absolute structure does not need
dispersive scatterers to be present. If present, the effects of
anomalous scattering must confirm the known absolute configuration of
the reference. It is important to stress that the correctness of
absolute-structure determination using an internal chiral reference
depends crucially on the knowledge of the enantiopurity of the
reference material and its indicated absolute configuration.

### 6. Determination of absolute configuration from absolute structure

Bijvoet (1949) and Peerdeman *et al*. (1951)
achieved the first determination of absolute configuration by X-ray
diffraction. In the terms used here, we would say that the absolute
structure of the crystal was first determined by single-crystal X-ray
diffraction with dispersive scatterers followed by the deduction of the
absolute configuration from this.

Not all valid determinations of absolute structure can necessarily lead to the assignment of an absolute configuration.

*Space-group restriction*:
the simplest restriction is one of space-group symmetry. If the space
group contains symmetry operations of the second kind, it must occur
that these operate either intramolecularly, forcing the individual
molecules to be achiral, or intermolecularly, forcing an arrangement of
pairs of opposite enantiomers. Thus, in the first case, the molecules
are achiral and in the second a racemate is present. It is only in
crystals displaying space groups containing exclusively symmetry
operations of the first kind (point groups: 1, 2, 222, 4, 422, 3, 32,
6, 622, 23, 432) that the determination of absolute configuration is
possible.

*Chiral molecular entity restriction*:
to comply with the definition of absolute configuration, one needs to
identify a chiral molecular entity and its spatial arrangement in the
crystal structure. For example, Bijvoet (1949) and Peerdeman *et al.* (1951)
established the absolute configuration of the tartrate anion (a chiral
molecule) but correctly made no claims to have performed so for the
sodium or rubidium cations, which are not molecules. The intramolecular
symmetry operations of the second kind forcing a molecule to be achiral
are either crystallographic (see *space-group restriction*)
or noncrystallographic. The spatial arrangement of a candidate molecule
for absolute-configuration determination must be examined for
noncrystallographic symmetry operations of the second kind. If any are
found, the molecule is achiral and its absolute configuration cannot be
determined.

*Solid-state enantiopurity restriction*:
one needs to verify that all occurrences in the crystal structure of
the chiral molecular entity are the same enantiomer. When the space
group contains no symmetry operations of the second kind (see *space-group restriction*)
and the asymmetric unit contains more than one occurrence of the chiral
molecule, the spatial arrangements of these molecules must be examined
to see if they are the same enantiomer. All occurrences of a chiral
molecule in a crystal structure must have the same chirality sense for
an absolute-configuration assignment to be valid.

### 7. Characterization of crystals and chiral molecules

For absolute-structure and absolute-configuration determinations to
have a practical value, their results need to be applicable to other
crystals or samples obtained from the bulk product. The sample used
needs to be physically characterized to permit the absolute-structure
or absolute-configuration determination to be reliably applicable to
other samples. In absolute-configuration determination, the chiral
molecules that constitute the bulk substance are the subject of
interest and it would seem important to characterize them in the form
that they are used by the chemist, most frequently, in solution. The
absolute configuration of a molecule as determined by crystal structure
analysis is that of the solid state. The spatial arrangement of the
atoms in a molecule in the solid state may not be the same as that in
solution.

#### 7.1. Characterization of crystals

Will every single crystal taken from the same batch or even from
different batches have the same absolute structure? For crystals formed
by the crystallization of an enantiopure substance, the crystals will
be chiral and all of the same enantiomorph. In this short section, on
the other hand, we will recall a few important cases in which
individual crystals, or the molecules in the crystals, are not
characteristic of the bulk substance. This list is not exhaustive. The
subject of *enantiomers*, *racemates* and resolutions is dealt with in detail by Jacques *et al.* (1994) and more briefly but more recently by Eliel & Wilen (1994).

A
racemic conglomerate may be formed by spontaneous resolution in the
crystallization of a racemate. Taking an arbitrary choice of crystal,
the enantiomorph will be arbitrary. When the crystallization system
contains an excess of one enantiomer, a mixture may be formed
containing an excess of the one enantiomorph corresponding to the
enantiomer in excess and an arbitrary choice of crystal may not be of
that enantiomorph that is in excess.

Crystals with a chiral
structure formed from achiral molecules in solution are a mechanical
mixture of the two enantiomorphs. This is not a racemic conglomerate
since the molecule is achiral. The proportion of the two enantiomorphs
in the mechanical mixture is not fixed, and the phase diagram of this
system and that of a racemate forming a racemic conglomerate are
essentially different. Taking an arbitrary choice of crystal, the
enantiomorph will be arbitrary.

In the preparation of crystals
containing an internal chiral reference, the starting solution contains
either the pure enantiomer *R* or the pure enantiomer *S* or perhaps a mixture of the two in unknown proportion. This solution is reacted or cocrystallized with another molecule *R*', known to be enantiomerically pure. *R*-*R*' and *S*-*R*'
are diastereoisomers and hence will be formed at different rates. The
equilibrium point of the two reactions will be different. The
solubility of *R*-*R*' and *S*-*R*' will be different. Under these conditions, it is easy to see that the proportion of crystals *R*-*R*' and crystals *S*-*R*' will not correspond to the proportion of the starting enantiomers *R* and *S* in solution. Only in the case of enantiomerically pure starting material will any crystal be characteristic of it.

#### 7.2. Characterization of chiral molecules

Classically, optical activity has been used as a fingerprint to
characterize chiral molecules, since the symmetry restrictions on
optical activity in solution are identical to those of enantiomers (the
same is not true for the crystalline state). However, measurement of
the optical rotation does not of itself establish the enantiopurity of
a substance. Calculation of optical activity is very tricky, even if
the molecular structure is known. Moreover, optical activity is
dependent on wavelength, concentration and solvent. For the synthetic
chemist, measurement of optical activity has taken a secondary role
behind modern physicochemical techniques, such as asymmetric
chromatography on enantioselective stationary phase, NMR, mass
spectroscopy *etc*. Once calibrated
correctly with a suitable mixture of enantiomers, these techniques are
capable of establishing the enantiopurity of a substance and even in
some cases of separating enantiomers.

### 8. Determination of absolute structure in twinned crystals

A twinned crystal may be viewed as a solid-state agglomerated
mixture of rotated and/or inverted copies of the untwinned crystal
structure. Each component in this mixture is specified by two
attributes.

|  |
| --- |
| - (i) The volume fraction *x**i* of the *i*th component in the macroscopic crystal. This value may be established during structure refinement. - (ii) The Euclidean isometry relating the   orientation of the component to that of the basic one. This   twin-symmetry operation may be established by arguments of symmetry   (Janovec, 1972; Flack, 1987) and is not unique. It comes from a group *G* of Euclidean isometries of which the crystal point group *P* is a subgroup, . The twin-symmetry operation is in fact a representative of a left coset in the decomposition of *G* with respect to *P*. |

#### 8.1. Rotation-only point groups

We will deal solely with the case in which *P*
is one of the 11 noncentrosymmetric point groups containing only
rotations (point groups: 1, 2, 222, 4, 422, 3, 32, 6, 622, 23, 432);
the analysis is simple and the results are of use in the determination
of absolute configuration. The symmetry operations of *P*,
being operations of the first kind, have a determinant of +1 in their
matrix representation. Thus, in the left coset decomposition of *G* with respect to *P*,
it is evident that all the symmetry operations in any one coset will
have the same determinant (either +1 or -1) since these are produced by
multiplying the symmetry operations of *P* on the left by one and the same symmetry operation drawn from *G*.
It results that for a rotation-only point group, although any
twin-symmetry operation is not unique, the determinant of equivalent
twin laws is invariant. Twin symmetry operations of determinant +1 will
produce a rotated-only image of the basic structure, whereas those with
determinant -1 will produce a rotated and inverted image. For the
purposes of the analysis of absolute structure, the total amount of
rotated-only structure, *x*+, may be deduced by summing the volume fractions corresponding to twin laws of determinant +1, , and that of rotated-and-inverted structure, *x*-, may be deduced by summing the volume fractions corresponding to twin laws of determinant -1, . *x*- is the equivalent of the Flack *x*
parameter for multiply twinned crystals. It must be emphasized that the
above analysis does not apply to the ten noncentrosymmetric point
groups containing symmetry operations of the second kind (point groups:
*m*, *mm*2, 4, 42*m*, 4*mm*, 3*m*, 6, 6*m*2, 6*mm*, 43*m*) nor to the 11 centrosymmetric point groups (point groups: 1, 2/*m*, *mmm*, 4/*m*, 4/*m**m**m*, 3, 3*m*, 6/*m*, 6/*m**m**m*, *m*3, *m*3*m*).

#### 8.2. Example 1 of Herbst-Irmer & Sheldrick (1998)

Consider example 1 of Herbst-Irmer & Sheldrick (1998), a twinned crystal of a compound containing a hydridochlorocarbonyltris(triphenylphosphine)osmium(II) ion in space group *P*31.
This space group contains only symmetry operations of determinant +1
and the above analysis may be applied. The structure was refined as a
four-component twin: `*k*2 = 0.064  (13) for matrix 010,100,00-1, *k*3 = 0.038  (17) for matrix -100,0-10,00-1 and *k*4 = 0.329  (13) for matrix 0-10,-100,001'. *k*1 may be obtained from the relationship *k*1 = 1 - *k*2 - *k*3 - *k*4 to give *k*1
= 0.569  (14) for matrix 100,010,001. The twin symmetry
operations are of determinant +1 for matrices 1 and 2, and -1 for
matrices 3 and 4. In the nomenclature of the current analysis, one has *x*1+ ( = *k*1) = 0.569 (14), *x*2+ ( = *k*2) =   0.064 (13), *x*1- ( = *k*3) = 0.038 (17) and *x*2- ( = *k*4) =   0.329 (13), giving *x*+ ( = *x*1+ + *x*2+) = 0.633 (17) and *x*- ( = *x*1- + *x*2-) = 0.367 (17). The standard uncertainty of *x*- (equivalent to the Flack *x*
parameter for this multiply twinned crystal) is low and one thus sees
that the inversion-distinguishing power of the measurements is very
good, as expected by Herbst-Irmer & Sheldrick (1998).
The experiment clearly shows that 63% of the crystalline sample
contains the structure determined by Herbst-Irmer & Sheldrick (1998) in space group *P*31 and 37% contains the inverted structure in space group *P*32.
From these measurements, it is clearly neither possible to establish
the absolute structure nor possible to establish the absolute
configuration for this compound, for which in any case the synthesis
was probably not stereospecific.

### 9. Defining absolute structure and absolute configuration

The definition of *absolute structure* as given in the glossary (§1) appears here for the first time in print, whereas that of *absolute configuration* is essentially the IUPAC definition (Moss, 1996)
with a small but most significant modification. The two definitions are
written in the same style in order to emphasize their similarities and
differences.

Our modification of the IUPAC definition of *absolute configuration* consists of the adjunction of the words *physically identified* as a qualifier to *chiral molecular entity*.
It is thought of importance to add these words as there is little use
of an absolute-configuration determination if the substance studied
cannot be reliably identified at a later stage. On this point, the
IUPAC (1996) definition has already been criticized by Eliel (Eliel
& Wilen, 1994) and Collet (1998) and it is instructive to note that previously Jacques *et al.* (1994)
insisted that the optical activity of the substance should be reported
for an absolute-configuration determination to be valid. As mentioned
above, other techniques are now also available. The expression *physically identified* has also been included in the definition of *absolute structure*, echoing the plea of Glazer & Stadnicka (1989)
that, as part of absolute-structure determination, the characterization
of the crystal itself by measurement of a physical, chemical or
morphological property be undertaken and reported. These authors also
provide a scheme for the consistent naming of the property.

The definition of *absolute configuration*
makes it clear that the term is to be applied only to chiral molecular
entities and not to whole crystals. This is in agreement with the
recommendations of Glazer & Stadnicka (1989)
`...that the term absolute configuration should be reserved only for
molecular species and not for crystal structures'. We concur with
Glazer & Stadnicka on this point.

Jones (1998) in his definition (Jones, 1984*a*) of the expression `determination of absolute structure', *viz*
“...it is often necessary to consider carefully exactly which
manifestation of noncentrosymmetry is being determined by the analysis
of anomalous scattering effects. It is unfortunate that there is no
general expression corresponding to `determination of absolute
configuration (or conformation) or of polar-axis direction or resolving
the ambiguity of enantiomorphic space-group pairs or of axis
directions'... I suggest the use of `determination of absolute
structure'...”, wished to provide a blanket term for the entity now
expressed by the Flack (1983) parameter. Several factors make us think that the time is now opportune to make much finer use of *absolute structure*.
Firstly, in the reporting of an analysis of a noncentrosymmetric
structure which does not seek to determine absolute structure or
absolute configuration, it is sufficient to state the value and
standard uncertainty obtained for the Flack parameter as proof that a
polar-dispersion error has not been made. In this case, no physical
interpretation of the Flack parameter is undertaken and no physical
characterization of the crystal or molecules is necessary. Use of
`determination of absolute structure' according to Jones may more
clearly be expressed by `refinement of the Flack (1983) parameter'. Secondly, for a property to be worthy of carrying the *absolute* qualifier, it must be adequately physically identified. Thirdly and lastly, it is natural for a crystal that a property *structure* should describe a spatial arrangement of atoms represented by a list of atomic coordinates. As defined, *absolute structure* takes on a clear and useful meaning, ensuring its continued use into the future.

### 10. Concluding remarks

> *On ne peut être trop prudent dans les
> conclusions à déduire de l'expérience, lorsque l'on a affaire à des
> substances quelquefois si semblables en apparence, et qui peuvent être
> au fond si différentes*. (Pasteur, 1848.)

In order to clarify the comprehension and presentation of absolute
structure, we have separated different but interacting concepts under
different names. Absolute structure describes the spatial arrangement
of atoms in a noncentrosymmetric crystal structure for which the
structure has been clearly distinguished from its inverse in a point.
This is in contrast to an ambiguous structure determination where the
results of the analysis are a choice between the two
centrosymmetrically related images. The inversion-distinguishing power
of a diffraction experiment measures its capacity to distinguish
between a crystal structure and its inverse. An experiment with high
inversion-distinguishing power has the capacity to lead to an
absolute-structure determination, whereas a low
inversion-distinguishing power permits only an ambiguous structure
determination. Inversion-distinguishing power may be increased in an
X-ray diffraction experiment either by the inclusion of dispersive
scatterers in the crystal structure, there being very few restrictions
on their position within the molecule or crystal structure, or by
suitably tuning the wavelength. A measure of the
inversion-distinguishing power of a diffraction experiment is afforded
by the reciprocal of the standard uncertainty of the Flack (1983) parameter.

The
underlying difficulty of the treatment of mixtures has appeared at
different points in this text. In contrast to pure, and in particular
enantiopure, substances, the crystals of which contain only molecules
of one single kind, impure substances, and in particular racemates and
samples with an enantiomeric excess, show a wide diversity in their
modes of crystallization and reactivity. It cannot be stressed
sufficiently that small amounts of impurity, and in particular the
opposite enantiomer as impurity in an `enantiopure' substance, may well
be the source of otherwise inexplicable results. Inversion twins and
multiply twinned crystals have been touched upon. These also are
mixtures of a special kind requiring careful treatment.

Caution
is always required in transferring an absolute-configuration assignment
to the bulk substance knowing that it has been derived from
measurements on only one single crystal and is characteristic of the
molecule in the solid state. It would seem a meagre precaution for the
publication of absolute-configuration determinations that proof of the
enantiopurity of the bulk sample should be provided. Furthermore,
measurement of the optical activity in solution continues to provide a
useful fingerprint and control. On the other hand, for those structure
determinations of which the primary objective is in the field of
structure-property relations, it is reasonable to expect that
absolute-structure determination will be accompanied by the measurement
of some chemical, physical or morphological property of the crystal.

### Acknowledgements

The authors wish to thank A. J. Blake, W.
Clegg, A. Collet, J. D. Dunitz, A. M. Glazer, Y. Grin, S. R. Hall,
P. G. Jones, J. Lacour, A. Linden, D. Schwarzenbach, A. L. Spek,
D. J. Watkin and the two referees for their invaluable comments on this
paper.

### References

Bernardinelli, G. & Flack, H. D. (1985). *Acta Cryst.* A**41**, 500-511.   
Bernardinelli, G. & Flack, H. D. (1987). *Acta Cryst.* A**43**, 75-78.   
Bijvoet, J. M. (1949). *Proc. K. Ned. Akad. Wet. Ser. B*, **52**, 313-314.   
Collet, A. (1998). Private communication.  
Coster, D. S., Knol, K. S. & Prins, J. A. (1930). *Z. Phys.* **63**, 345-369.   
Cruickshank, D. W. J. & McDonald, W. S. (1967). *Acta Cryst.* **23**, 9-11.    
Eliel, E. L. & Wilen, S. H. (1994). *Stereochemistry of Organic Compounds.* New York: John Wiley.  
Flack, H. D. (1983). *Acta Cryst.* A**39**, 876-881.    
Flack, H. D. (1987). *Acta Cryst.* A**43**, 564-568.   
Flack, H. D. & Bernardinelli, G. (1999). *Acta Cryst.* In preparation.  
Flack, H. D. & Schwarzenbach, D. (1988). *Acta Cryst.* A**44**, 499-506.   
Glazer, A. M. & Stadnicka, K. (1989). *Acta Cryst.* A**45**, 234-238.   
Hall, S. R., Allen, F. H. & Brown, I. D. (1991). *Acta Cryst.* A**47**, 655-685.   
Hamilton, W. C. (1965). *Acta Cryst.* **18**, 502-510.    
Herbst-Irmer, R. & Sheldrick, G. M. (1998). *Acta Cryst.* B**54**, 443-449.   
Jacques, J., Collet, A. & Wilen, S. H. (1994). *Enantiomers, Racemates and Resolutions.* Reissue with corrections. Malabar, FL: Krieger.  
Janovec, V. (1972). *Czech. J. Phys. B*, **22**, 974-994.   
Jones, P. G. (1984*a*). *Acta Cryst.* A**40**, 660-662.   
Jones, P. G. (1984*b*). *Acta Cryst.* A**40**, 663-668.   
Jones, P. G. (1986*a*). *Acta Cryst.* A**42**, 57.   
Jones, P. G. (1986*b*). *Acta Cryst.* C**42**, 924.   
Jones, P. G. (1998). Private communication  
Jones, P. G. & Meyer-Bäse, K. (1987). *Acta Cryst.* A**43**, 79-80.   
Jones, P. G., Schelbach, R., Schwarzmann, E. & Thöne, C. (1988). *Acta Cryst.* C**44**, 1196-1198.    
Moss, G. P. (1996). *Pure Appl. Chem.* **68**, 2193-2222. [*Basic Terminology of Stereochemistry*, *IUPAC Recommendations 1996*, available at http://www.Chem.qmw.ac.uk/iupac/stereo/.]   
Pasteur, L. (1848). *Ann. Chim. Phys.* **24**, 442-459.  
Peerdeman, A. F., van Bommel, A. J. & Bijvoet, J. M. (1951). *Proc. K. Ned. Akad. Wet. Ser. B*, **54**, 16-19.  
Rogers, D. (1975). *Anomalous Scattering*, edited by S. Ramaseshan & S. C. Abrahams, pp. 231-250. Copenhagen: Munksgaard.   
Rogers, D. (1981). *Acta Cryst.* A**37**, 734-741.   
Sheldrick, G. M. (1993/1997). *SHELXL*93/*SHELXL*97. *Program for the Refinement of Crystal Structures.* University of Göttingen, Germany.  
Ueki, T., Zalkin, A. & Templeton, D. H. (1966). *Acta Cryst.* **20**, 836-841.

---

*Acta Cryst* (1999). A**55**, 908-915   [ doi:10.1107/S0108767399004262 ]
