# Supplementary material for: Space groups and crystallographic symmetry: writing a multi-featured tutorial in a new style
Source: Acta Crystallogr E Crystallogr Commun. 2021 Jul 16;77(Pt 9):857–63. doi: 10.1107/S2056989021007039 (PMC8423017; doi:10.1107/S2056989021007039)
Supplement: Supplementary file 1 [file e-77-00857-sup2.zip › symandsg/Main/abso_files/sh0129hdr.htm]

(IUCr) Absolute structure and absolute configuration

|  |  |  |
| --- | --- | --- |
|  |  |  |
|  | | |

Go to section:Top1. Glossary of terms2. Introduction3. Absolute structure4. Determination of absolute structure by X-ray diffraction with dispersive scatterers5. Determination of absolute structure by X-ray diffraction using an internal chiral reference6. Determination of absolute configuration from absolute structure7. Characterization of crystals and chiral molecules8. Determination of absolute structure in twinned crystals9. Defining absolute structure and absolute configuration10. Concluding remarksReferences
