# Supplementary material for: Space groups and crystallographic symmetry: writing a multi-featured tutorial in a new style
Source: Acta Crystallogr E Crystallogr Commun. 2021 Jul 16;77(Pt 9):857–63. doi: 10.1107/S2056989021007039 (PMC8423017; doi:10.1107/S2056989021007039)
Supplement: Supplementary file 1 [file e-77-00857-sup2.zip › symandsg/Main/allspg.html]

Space Group Frequencies

Space Group Frequencies. Table of the percentage of
Organic/organometallic (C-H), Inorganic (M-M) and Protein (PDB)
structure space group frequencies in the Cambridge Crystallographic
Database(1997) [Allen, F.H., Kennard, O. Chemical Design Automation
News (1993) **8**, 31-37; Wilson A.J.C. (1988) A**44**, 715-724], the Inorganic Crystal Structure Data Base [Baur, W.H. & Kassner, D. Acta Cryst. (1992) B**48**,
356-369] and the Brookhaven Protein Data Base (6129 entries compiled by
Axel Brunger, Yale University, 1997) respectively. All results in
percentage of structures with the given space group in the given data
base.

Joseph H. Reibenspies, Ph.D. : mail inquires to j-reibenspies@tamu.edu

|  |  |  |  |  |
| --- | --- | --- | --- | --- |
| **#** | **Space Group** | **C-H%** | **M-M%** | **PDB%** |
| **1** | P1 | 0.97 | 0.4 | 2.3 |
| **2** | P-1 | 19.318 | 4.3 | - |
| **3** | P2 | 0.028 | 0.04 | 0.08 |
| **4** | P21 | 5.656 | 0.64 | 15.6 |
| **5** | C2 | 0.813 | 0.45 | 8.9 |
| **6** | Pm | 0.003 | 0.04 | - |
| **7** | Pc | 0.367 | 0.21 | - |
| **8** | Cm | 0.06 | 0.23 | - |
| **9** | Cc | 0.999 | 0.70 | - |
| **10** | P2/m | 0.025 | 0.2 | - |
| **11** | P21/m | 0.675 | 1.3 | - |
| **12** | C2/m | 0.515 | 3.4 | - |
| **13** | P2/c | 0.507 | 0.6 | - |
| **14** | P21/c | 35.177 | 8.1 | - |
| **15** | C2/c | 7.159 | 3.8 | - |
| **16** | P222 | 0.01 | 0.04 | 0 |
| **17** | P2221 | 0.022 | 0.05 | 0.03 |
| **18** | P21212 | 0.47 | 0.17 | 6.3 |
| **19** | P212121 | 9.013 | 1.1 | 23.9 |
| **20** | C2221 | 0.199 | 0.15 | 4.2 |
| **21** | C222 | 0.012 | 0.05 | 0.08 |
| **22** | F222 | 0.003 | 0.02 | 0.07 |
| **23** | I222 | 0.023 | 0.02 | 2.4 |
| **24** | I212121 | 0.006 | 0.005 | 0.07 |
| **25** | Pmm2 | 0.006 | 0.04 | - |
| **26** | Pmc21 | 0.026 | 0.16 | - |
| **27** | Pcc2 | 0.005 | 0 | - |
| **28** | Pma2 | 0.002 | 0.05 | - |
| **29** | Pca21 | 0.725 | 0.31 | - |
| **30** | Pnc2 | 0.02 | 0.02 | - |
| **31** | Pnm21 | 0.097 | 0.42 | - |
| **32** | Pba2 | 0.022 | 0.07 | - |
| **33** | Pna21 | 1.527 | 1.06 | - |
| **34** | Pnn2 | 0.036 | 0.07 | - |
| **35** | Cmm2 | 0.001 | 0.02 | - |
| **36** | Cmc21 | 0.166 | 0.7 | - |
| **37** | Ccc2 | 0.017 | 0.008 | - |
| **38** | Amm2 | 0.006 | 0.15 | - |
| **39** | Abm2 | 0.007 | 0.025 | - |
| **40** | Ama2 | 0.022 | 0.11 | - |
| **41** | Aba2 | 0.094 | 0.11 | - |
| **42** | Fmm2 | 0.014 | 0.03 | - |
| **43** | Fdd2 | 0.333 | 0.35 | - |
| **44** | Imm2 | 0.01 | 0.14 | - |
| **45** | Iba2 | 0.074 | 0.005 | - |
| **46** | Ima2 | 0.013 | 0.08 | - |
| **47** | Pmmm | 0.005 | 0.61 | - |
| **48** | Pnnn | 0.006 | 0.005 | - |
| **49** | Pccm | 0.002 | 0.005 | - |
| **50** | Pban | 0.007 | 0.03 | - |
| **51** | Pmma | 0.01 | 0.16 | - |
| **52** | Pnna | 0.079 | 0.14 | - |
| **53** | Pmna | 0.017 | 0.08 | - |
| **54** | Pcca | 0.041 | 0.08 | - |
| **55** | Pbam | 0.036 | 0.76 | - |
| **56** | Pccn | 0.354 | 0.14 | - |
| **57** | Pbcm | 0.139 | 0.35 | - |
| **58** | Pnnm | 0.083 | 0.78 | - |
| **59** | Pmmn | 0.05 | 0.34 | - |
| **60** | Pbcn | 0.897 | 0.76 | - |
| **61** | Pbca | 3.776 | 1.3 | - |
| **62** | Pnma | 1.537 | 3.2 | - |
| **63** | Cmcm | 0.133 | 1.85 | - |
| **64** | Cmca | 0.168 | 0.62 | - |
| **65** | Cmmm | 0.013 | 0.29 | - |
| **66** | Cccm | 0.013 | 0.13 | - |
| **67** | Cmma | 0.009 | 0.06 | - |
| **68** | Ccca | 0.038 | 0.08 | - |
| **69** | Fmmm | 0.011 | 0.14 | - |
| **70** | Fddd | 0.087 | 0.32 | - |
| **71** | Immm | 0.009 | 0.54 | - |
| **72** | Ibam | 0.053 | 0.28 | - |
| **73** | Ibca | 0.023 | 0.04 | - |
| **74** | Imma | 0.011 | 0.40 | - |
| **75** | P4 | 0.007 | 0.05 | 0.11 |
| **76** | P41 | 0.118 | 0.06 | 1.1 |
| **77** | P42 | 0.011 | 0.02 | 0.11 |
| **78** | P43 | 0.051 | 0.01 | 0.69 |
| **79** | I4 | 0.025 | 0.05 | 0.33 |
| **80** | I41 | 0.027 | 0.01 | 0.16 |
| **81** | P-4 | 0.027 | 0.03 | - |
| **82** | I-4 | 0.159 | 0.43 | - |
| **83** | P4/m | 0.01 | 0.05 | - |
| **84** | P42/m | 0.019 | 0.05 | - |
| **85** | P4/n | 0.101 | 0.17 | - |
| **86** | P42/n | 0.136 | 0.17 | - |
| **87** | I4/m | 0.061 | 0.42 | - |
| **88** | I41/a | 0.322 | 0.52 | - |
| **89** | P422 | 0.003 | 0.002 | 0.07 |
| **90** | P4212 | 0.009 | 0.01 | 0.34 |
| **91** | P4122 | 0.009 | 0.01 | 0.08 |
| **92** | P41212 | 0.264 | 0.25 | 3.1 |
| **93** | P4222 | 0.002 | 0 | 0.03 |
| **94** | P42212 | 0.023 | 0.04 | 1.0 |
| **95** | P4322 | 0.002 | 0.01 | 0.15 |
| **96** | P43212 | 0.127 | 0.08 | 5.20 |
| **97** | I422 | 0.006 | 0.03 | 0.51 |
| **98** | I4122 | 0.006 | 0.01 | 0.59 |
| **99** | P4mm | 0.001 | 0.25 | - |
| **100** | P4bm | 0.001 | 0.08 | - |
| **101** | P42cm | 0.002 | 0 | - |
| **102** | P42nm | 0.005 | 0.04 | - |
| **103** | P4cc | 0.001 | 0.02 | - |
| **104** | P4nc | 0.015 | 0 | - |
| **105** | P42mc | 0.001 | 0.01 | - |
| **106** | P42bc | 0.008 | 0.005 | - |
| **107** | I4mm | 0.004 | 0.08 | - |
| **108** | I4cm | 0.004 | 0.04 | - |
| **109** | I41md | 0.009 | 0.06 | - |
| **110** | I41cd | 0.039 | 0.04 | - |
| **111** | P-42m | 0.002 | 0.06 | - |
| **112** | P-42c | 0.003 | 0.02 | - |
| **113** | P-421m | 0.038 | 0.27 | - |
| **114** | P-421c | 0.143 | 0.12 | - |
| **115** | P-4m2 | 0.001 | 0.02 | - |
| **116** | P-4c2 | 0.002 | 0.02 | - |
| **117** | P-4b2 | 0.006 | 0.05 | - |
| **118** | P-4n2 | 0.026 | 0.05 | - |
| **119** | I-4m2 | 0.005 | 0.06 | - |
| **120** | I-4c2 | 0.013 | 0.04 | - |
| **121** | I-42m | 0.029 | 0.30 | - |
| **122** | I-42d | 0.055 | 0.66 | - |
| **123** | P4/mmm | 0.017 | 0.74 | - |
| **124** | P4/mcc | 0.02 | 0.05 | - |
| **125** | P4/nbm | 0.003 | 0.001 | - |
| **126** | P4/nnc | 0.022 | 0.002 | - |
| **127** | P4/mbm | 0.027 | 0.51 | - |
| **128** | P4/mnc | 0.013 | 0.24 | - |
| **129** | P4/nmm | 0.042 | 1.0 | - |
| **130** | P4/ncc | 0.041 | 0.14 | - |
| **131** | P42/mmc | 0.003 | 0.05 | - |
| **132** | P42/mcm | 0.002 | 0.004 | - |
| **133** | P42/nbc | 0.004 | 0.004 | - |
| **134** | P42/nnm | 0.009 | 0.05 | - |
| **135** | P42/mbc | 0.009 | 0.16 | - |
| **136** | P42/mnm | 0.025 | 0.80 | - |
| **137** | P42/mnc | 0.016 | 0.11 | - |
| **138** | P42/ncm | 0.009 | 0.04 | - |
| **139** | I4/mmm | 0.018 | 2.4 | - |
| **140** | I4/mcm | 0.006 | 0.98 | - |
| **141** | I41/amd | 0.021 | 0.96 | - |
| **142** | I41/acd | 0.042 | 0.16 | - |
| **143** | P3 | 0.034 | 0.10 | 0.10 |
| **144** | P31 | 0.081 | 0.08 | 0.24 |
| **145** | P32 | 0.061 | 0.01 | 0.59 |
| **146** | R3 | 0.148 | 0.25 | 1.27 |
| **147** | P-3 | 0.094 | 0.20 | - |
| **148** | R-3 | 0.485 | 1.4 | - |
| **149** | P312 | 0.001 | 0.04 | 0.02 |
| **150** | P321 | 0.006 | 0.22 | 0.67 |
| **151** | P3112 | 0.001 | 0.03 | 0.03 |
| **152** | P3121 | 0.111 | 0.42 | 3.7 |
| **153** | P3212 | 0.002 | 0.001 | 0.11 |
| **154** | P3221 | 0.053 | 0.08 | 7.33 |
| **155** | R32 | 0.043 | 0.19 | 1.19 |
| **156** | P3m1 | 0.002 | 0.20 | - |
| **157** | P31m | 0.003 | 0.09 | - |
| **158** | P3c1 | 0.011 | 0.02 | - |
| **159** | P31c | 0.023 | 0.13 | - |
| **160** | R3m | 0.039 | 0.64 | - |
| **161** | R3c | 0.136 | 0.28 | - |
| **162** | P-31m | 0.001 | 0.16 | - |
| **163** | P-31c | 0.032 | 0.14 | - |
| **164** | P-3m1 | 0.02 | 1.69 | - |
| **165** | P-3c1 | 0.047 | 0.25 | - |
| **166** | R-3m | 0.041 | 2.48 | - |
| **167** | R-3c | 0.111 | 1.32 | - |
| **168** | P6 | 0.004 | 0.002 | 1.06 |
| **169** | P61 | 0.061 | 0.05 | 1.31 |
| **170** | P65 | 0.043 | 0.02 | 0.64 |
| **171** | P62 | 0.007 | 0.001 | 0.03 |
| **172** | P64 | 0.004 | 0 | 0.05 |
| **173** | P63 | 0.075 | 0.54 | 0.47 |
| **174** | P-6 | 0.005 | 0.29 | - |
| **175** | P6/m | 0.003 | 0.05 | - |
| **176** | P63/m | 0.16 | 1.2 | - |
| **177** | P622 | 0.001 | 0.05 | 0.05 |
| **178** | P6122 | 0.022 | 0.004 | 2.12 |
| **179** | P6522 | 0.01 | 0 | 0.73 |
| **180** | P6222 | 0.005 | 0.19 | 0.34 |
| **181** | P6422 | 0.005 | 0.05 | 0.31 |
| **182** | P6322 | 0.008 | 0.10 | 0.59 |
| **183** | P6mm | 0.002 | 0 | - |
| **184** | P6cc | 0 | 0.001 | - |
| **185** | P63cm | 0.003 | 0.13 | - |
| **186** | P63mc | 0.029 | 0.83 | - |
| **187** | P-6m2 | 0 | 0.14 | - |
| **188** | P-6c2 | 0.002 | 0.08 | - |
| **189** | P-62m | 0.005 | 0.68 | - |
| **190** | P-62c | 0.015 | 0.11 | - |
| **191** | P6/mmm | 0.007 | 1.4 | - |
| **192** | P6/mcc | 0.009 | 0.19 | - |
| **193** | P63/mcm | 0.002 | 0.66 | - |
| **194** | P63/mmc | 0.026 | 3.91 | - |
| **195** | P23 | 0.003 | 0.05 | 0.02 |
| **196** | F23 | 0.005 | 0.11 | 0.02 |
| **197** | I23 | 0.01 | 0.12 | 0.33 |
| **198** | P213 | 0.069 | 0.47 | 0.95 |
| **199** | I213 | 0.002 | 0.13 | 0.39 |
| **200** | Pm-3 | 0.004 | 0.08 | - |
| **201** | Pn-3 | 0.005 | 0.11 | - |
| **202** | Fm-3 | 0.004 | 0.10 | - |
| **203** | Fd-3 | 0.008 | 0.10 | - |
| **204** | Im-3 | 0.007 | 0.29 | - |
| **205** | Pa-3 | 0.115 | 0.61 | - |
| **206** | Ia-3 | 0.007 | 0.12 | - |
| **207** | P432 | 0.001 | 0.002 | 0.02 |
| **208** | P4232 | 0 | 0.10 | 0.05 |
| **209** | F432 | 0.001 | 0.01 | 0.38 |
| **210** | F4132 | 0.003 | 0.01 | 0.13 |
| **211** | I432 | 0.001 | 0.05 | 0.18 |
| **212** | P4332 | 0.003 | 0.10 | 0.03 |
| **213** | P4132 | 0.006 | 0.05 | 0.07 |
| **214** | I4132 | 0 | 0.05 | 0.10 |
| **215** | P-43m | 0.011 | 0.25 | - |
| **216** | F-43m | 0.014 | 1.01 | - |
| **217** | I-43m | 0.028 | 0.41 | - |
| **218** | P-43n | 0.019 | 0.25 | - |
| **219** | F-43c | 0.011 | 0.07 | - |
| **220** | I-43d | 0.019 | 0.28 | - |
| **221** | Pm-3m | 0.012 | 1.58 | - |
| **222** | Pn-3n | 0.007 | 0.008 | - |
| **223** | Pm-3n | 0.006 | 0.15 | - |
| **224** | Pn-3m | 0.003 | 0.008 | - |
| **225** | Fm-3m | 0.033 | 4.4 | - |
| **226** | Fm-3c | 0.001 | 0.08 | - |
| **227** | Fd-3m | 0.019 | 3.0 | - |
| **228** | Fd-3c | 0.008 | 0.05 | - |
| **229** | Im-3m | 0.021 | 0.26 | - |
| **230** | Ia-3d | 0.002 | 0.72 | - |

 

 

 
