# Supplementary material for: Space groups and crystallographic symmetry: writing a multi-featured tutorial in a new style
Source: Acta Crystallogr E Crystallogr Commun. 2021 Jul 16;77(Pt 9):857–63. doi: 10.1107/S2056989021007039 (PMC8423017; doi:10.1107/S2056989021007039)
Supplement: Supplementary file 1 [file e-77-00857-sup2.zip › symandsg/Main/bcamember.htm]

BCA Membership

|  |  |  |  |  |  |  |  |  |  |  |  |  |  |
| --- | --- | --- | --- | --- | --- | --- | --- | --- | --- | --- | --- | --- | --- |
|  | |  |  |  |  |  |  | | --- | --- | --- | --- | --- | --- | | BCA Office | Join Now! | Donations | History | Corporate Membership | BCA Site Map | | **BCA Membership** | Gift Aid | Bursaries | Statutes | ECA Membership | Contacts | |

---

**BCA Membership**

---

**BCA Membership**

Thank you for your interest in BCA membership, BCA Council hopes that you will
join our 1000+ membership.

**Why join the BCA?**

The BCA is a UK registered charity founded in 1982 to advance the education of
the public in the science of crystallography,
particularly within the British Isles.

**Some Benefits of Individual Membership**

1. ***THE*** professional organisation for crystallographers in the UK.
2. Comprehensive schedule of UK meetings, including the Annual BCA Meeting
   each Spring, plus one-day meetings and workshops organised by
   the four subject groups.
3. Avoid non-member supplements for meetings organised under BCA auspices.
4. Regular listings of meetings of crystallographic interest,
   both in the UK and worldwide.
5. After 6 months of membership, students and postdocs can apply for
   an Arnold Beevers Bursary
   award for attendance at any relevant scientific meeting.
6. Receive a free copy of *Crystallography News* in
   A4 format every quarter;
   the BCA newsletter is full of crystallographic news and information, product
   advertisements, meeting schedules, job listings, etc.
7. Receive optional and occasional E-mail notifications of news items and
   meeting information.
8. *NEW* starting 2003!
   Receive a BCA Spring Meeting special edition of
   "Crystallography Reviews" each year.
9. Have your say in the development of crystallography, and nominate and
   vote for BCA Officers, Council Members and subject-group Committee Members,
   or even stand for office yourself!
10. Current subscription covers UK membership of the
    European
    Crystallographic Association and over half the UK subscription to the
    International Union of Crystallography
    (IUCr).

**So join the BCA *now*!**

Members names, addresses, and details of
their choice of BCA Groups and date of
payment of subscriptions are held in a computer database, to simplify our
membership records and for the mailing of *Crystallography News*.

Members can check details of their entry in the BCA database at any time
by contacting the BCA Administrative Office, or interactively at the
Annual Spring Meeting.

Members may update their details either on-line
or by contacting the BCA Administrative Office directly.

**Contacting the BCA**

There are three main contacts regarding BCA matters:

- BCA Administrative Office;
- Editor *Crystallography News*;
- BCA Webmaster.

---

|  |  |
| --- | --- |
| � Copyright.  British Crystallographic Association. |  |
