# Supplementary material for: Space groups and crystallographic symmetry: writing a multi-featured tutorial in a new style
Source: Acta Crystallogr E Crystallogr Commun. 2021 Jul 16;77(Pt 9):857–63. doi: 10.1107/S2056989021007039 (PMC8423017; doi:10.1107/S2056989021007039)
Supplement: Supplementary file 1 [file e-77-00857-sup2.zip › symandsg/Main/beev.html]

von Groth


# Historical Reminisences

  
Paul von Groth, who lived in Munich from 1843 to 1927 was the doyen of what
I like to call "Classical Crystallography" - the study of the subject before
the advent of X-rays. he was a pioneer in the development of the optical
goniometer (with the manufacturer Fuess) and he wrote the monumental
collection of crystal data called "Chemische Kristallographie", in five
volumes between 1906 and 1920.
*part of a page in the fourth edition with tables and diagrams, published in 1906.*   
It would seem that all known crystals are
described here, in sections such as "Sulphates of Monovalent metals". each
section has a critical review of the subject and for each substance there is
a detailed consideration of the crystal class (i.e.symmetry), the indices
of the various faces, the angles between them and the resultant axial
ratios.
This information forms an ideal introduction for the X-ray
diffraction work which has followed in subsequent years. the optical
properties are also discussed and the various refractive indices given,
usually to a four-figure accuracy, as are also the axial ratios. The whole
work could only have been accomplished by long years of devotion.
Furthermore von Groth was the founder and first editor of the international
journal **"Zeitschrift F�r Kristallographie"**,
begun in 1877 and still in regular publication.

My own work in crystallography began in the Physics department of the
University of Liverpool where no crystallography or mineralogy had been
taught. I was expected to solve the crystal structures of some salt
hydrates. Working later with Henry Lipson we relied heavily on the writings
of von Groth. We had at this time to construct our own X-ray tubes. We
pioneered the use of Fourier projections in structure determination and
supplied hundreds of "Beevers-Lipson" strips which expedited the
calculations considerably. These methods are of course now carried out by
digital computers at some millions of times the speed. In 1938 I came to the
University of Edinburgh as Dewar Research Fellow, and continued supplying
Fourier strips to the crystallographic world and continued with research and
teaching. I also took an interest in the history of both Chemistry and
Crystallography in Edinburgh. In looking through the contents of a somewhat
neglected attic in the Department I came across a rather soiled picture. It
was a photographic copy of an oil painting of von Groth painted by a local
artist in 1904, evidently to celebrate his 60th birthday. The legend
beneath the picture stated "Given by His Friends in Many Lands". Now we had
in Edinburgh at that time a famous professor of Chemistry, Alexander Crum
Brown, who had taught not only general Chemistry but also Crystallography.
he had taken von Groth's "Zeitschrift" from the first number and had a
collection of Fuess goniometers, crystals and crystal models in his museum.
Evidently Crum Brown had been a subscriber to his portrait. His great
interest in crystals is also shown by a model made by him in 1880 of the
structure of rock salt. the atoms in this model are made from balls of wool,
alternately red and blue, wound around the junctions of knitting needles to
give a correct representation of the well known structure.

I paid a disciple's visit to Munich in March 1995 and was shown the museum
there with its souvenirs of von Groth. They have there the original
portrait, but it has not been preserved as our copy in Edinburgh, which is
now framed in our Crum Brown museum. I am very happy to have established
this link between our two cities and two crystal enthusiast of a century
ago.

*C.Arnold Beevers  
Edinburgh June 1995*

**Editor's Note:**  
I am grateful to the Librarian and the Assistant Curator of Minerals in the
University Museum, Oxford, who kindly showed me some of their books by
Groth and helped me to find the illustrations for this article.The portrait
upper left on the previous page is from the 'Obituary Notice', by Sir Henry
A. Miers published in the ''Proceedings of the Royal Society' A Vol 119
1928. The portrait has no caption, just his signature. The diagram is part
of page 347 in the fourth edition of 'Chemische Kristallographie' published
in 1905, with 801 pages, 750 woodcuts (for the diagrams) and 3 colour
plates.Clearly it is much easier to make diagrams using computers in 1995!

We have since found out that the portrait was comissioned by his friends who
subscribed to give him a present of the portrait to commemorate the 25th
anniversary of the founding of "Zeitschrift F�r Kristallographie" which
happened to coincide with his 60th birthday.

We have also found that von Groth designed a set of wooden models for
teaching crystallography, which were manufactured by Krantz in Bonn. Ulrich
Burchard has written a book about them illustrated with drawings made by the
'Shape' program. The book, latest version of 'Shape' and the datafiles to
generate all the drawings are available for DM500.

Click here to return to BCA homepage
