# Supplementary material for: Space groups and crystallographic symmetry: writing a multi-featured tutorial in a new style
Source: Acta Crystallogr E Crystallogr Commun. 2021 Jul 16;77(Pt 9):857–63. doi: 10.1107/S2056989021007039 (PMC8423017; doi:10.1107/S2056989021007039)
Supplement: Supplementary file 1 [file e-77-00857-sup2.zip › symandsg/Main/bennett.htm]

(IUCr) Crystallography Journals Online - paper details

### Acta Crystallographica Section A

# Crystal Physics, Diffraction, Theoretical and General Crystallography

### Volume 31, Part 4 (July 1975)

---

## research papers

---

  
 

*Acta Cryst.* (1975). A**31**, 488-494    [ doi:10.1107/S0567739475001052 ]

### Applications of the King and Lipscomb expression for the X-ray scattering of a hindered rotor

### M. J. Bennett, W. L. Hutcheon and B. M. Foxman

|  |
| --- |
| bibliographic record in BIBTeX EndNote RefMan Refer Medline CIF SGML Plain Text  format |

---

|  |  |  |  |
| --- | --- | --- | --- |
| |  | | --- | | **Find reference:**  Acta Cryst. Acta Cryst. A Acta Cryst. B Acta Cryst. C Acta Cryst. D Acta Cryst. E Acta Cryst. F J. Appl. Cryst. J. Synchrotron Rad. **Volume** **Page** |  |  | | --- | | **Search:**  **From**   1948  1951  1961  1971  1981  1991  1996  1997  1998  1999  2000  2001  2002  2003  2004  2005  **to**   1950  1960  1970  1980  1990  1995  1996  1997  1998  1999  2000  2001  2002  2003  2004  2005  2006     Advanced search | |  |

Copyright © International Union of Crystallography
  
*IUCr Webmaster*
