# Supplementary material for: Space groups and crystallographic symmetry: writing a multi-featured tutorial in a new style
Source: Acta Crystallogr E Crystallogr Commun. 2021 Jul 16;77(Pt 9):857–63. doi: 10.1107/S2056989021007039 (PMC8423017; doi:10.1107/S2056989021007039)
Supplement: Supplementary file 1 [file e-77-00857-sup2.zip › symandsg/Main/bertaut.html]

(IUCr) Erwin Félix Lewy-Bertaut (1913-2003)

## crystallographers

---

|  |  |
| --- | --- |
| **Volume 37**   **Part 2**    Pages 349-350    April 2004    Received 25 February 2003  Accepted 25 February 2003  © International Union of Crystallography 2004 | Erwin Félix Lewy-Bertaut (1913-2003)   **Keywords: obituary.** |

Born in 1913 in Leobschütz (presently
in Poland but at the time in Germany), he was an undergraduate in law.
The political change in Germany in 1933 and the subsequent persecution
of the Jewish people, convinced him to emigrate to Paris. Finally, he
reached Bordeaux where he was a graduate student in chemistry. In
February 1939, just before the Second World War, he interrupted his PhD
thesis on colophany to join the French Army. After the French defeat,
he received a temporary identity card under the name Félix Bertaut. He
had a chemistry position in the `free' southern part of France where he
improved the resistance of bike brakes, made of cork, the bicycle being
the quasi-exclusive way of transportation at this time. To avoid police
controls, with the help of Alfred Kastler (Kastler, 1994), he joined the Laboratoire Central des Poudres where he learned how to use the *International Tables of Crystallography*. Following another police control he had to escape and was sent to work with Professor Louis Neel (Neel, 1970) in Grenoble, located at the time in the `free area' of France.

|  |  |
| --- | --- |
|  | **Figure 1**  Erwin Félix Lewy-Bertaut, crystallographer, CNRS honorary research director and member of the French Académie des Sciences, deceased in Grenoble on 6 November 2003. |

Professor Louis Neel was studying magnetism and the crystallography
notions of Félix Bertaut were very useful to this small research team
(most of them `emigrated' from France or elsewhere). Erwin Lewy
obtained a CNRS research fellowship at the CNRS under the name Félix
Bertaut and, under Neel's supervision, he contributed to the first
large CNRS laboratory outside Paris: the Laboratoire d'Electrostatique
et de Physique du Métal (LEPM) in Grenoble. With Jacques Mehring, he
constructed a crude X-ray apparatus. At the same time, Louis Weil
succeeded in synthesizing small-particle iron powders which would be
very good materials as permanent magnets. Hence, Félix Bertaut chose a
new thesis subject: *Study of size statistical distribution of iron grains by means of X-rays*.
His thesis also presented an industrial aim since these magnets were
used in bike dynamos. Furthermore, this grain-size distribution was
needed for Louis Neel's studies on magnetism. Félix Bertaut defended
his PhD thesis in February 1949 under the professorship of André
Guinier. The method developed by Bertaut (Bertaut, 1950, 1952) is always a reference in powder granulometry.

|  |  |
| --- | --- |
|  | **Figure 2**  Professor Erwin Félix Lewy-Bertaut with Professor André Guinier when he was appointed a full member of the French Academia of Sciences in 1979. |

Just after his thesis in 1949, his scientific destiny underwent a
substantial change of direction following a single-page publication by
C. G. Shull (Shull, 1994) and J. S. Smart who evidenced the `antiferromagnetic' order in MnO by neutron diffraction (Shull & Smart, 1949) and confirmed the theory proposed some 15 years before by Louis Neel (Neel, 1932, 1936).
Neel became enthusiastic and wanted to perform similar studies in
Grenoble. To improve his knowledge on this topic, Félix Bertaut visited
the United States, first in 1951, and then in 1953 with the support of
a Fulbright grant. In spite of the McCarthy atmosphere, with the help
of Ray Pepinsky he could visit the Brookhaven National Laboratory
centre and the neutron diffraction experiment of Lester Corliss and
Julius Hastings. With the French decision to install an atomic research
centre in Grenoble (CENG) under the direction of Louis Neel, Félix
Bertaut created the `Diffraction Neutronique' laboratory, headed by
himself from 1958 to 1976. During this time the LEPM increased in size
and was eventually split into several laboratories in 1971; the CNRS
Laboratoire de Cristallographie is one of them, also headed by Félix
Bertaut from 1971 to 1982. All these laboratories are located in a new
large area, a past military artillery `polygon', bought for a symbolic
price by Louis Neel. During this time, Félix Bertaut, Francis Forrat
and René Pauthenet became famous with their discovery of garnet
ferrites (Pauthenet & Blum, 1954; Bertaut & Forrat, 1956). Now, garnet ferrites are a key material for magnetic memories and for high-frequency electronics.

|  |  |
| --- | --- |
|  | **Figure 3**  Garnet ferrites also have the honour of being incrusted on the Félix Bertaut Academy's sword. |

As a scientific director, we addressed him as Monsieur Bertaut. His
research strategy was based on a parallel development of the synthesis
of new compounds and crystallography methods. His students had to
understand and use these new methods that he taught with enthusiasm and
the new materials they grew had to be not only theoretically
interesting but also industrially useful. Monsieur Bertaut did not
hesitate to initiate PhD works on innovative instrumental projects, in
spite of the risk incurred. Once given the thesis subject, he left
students quite free to carry out their research on their own. In fact,
his personality was sufficient to induce an innovative research
atmosphere. He had a lot of ideas ranging from realistic ones to
totally impossible others. In most of the laboratory meetings he was
the youngest in mind, although he was 25 years older than all the
others.

He was an eclectic man, learning latin, greek, french,
english, law, then chemistry and crystallography and, as mentioned by
Professor Andre Guinier (Guinier, 1969-1972),
`Félix Bertaut is a mathematician who does crystallography'. Félix
Bertaut has been concerned with different aspects in crystallography.
After his PhD work where he first discriminated the grain size itself
from the grain size distribution (Bertaut, 1950, 1952), he solved the structure of complex compounds like the pyrrhotite with a non-stoechiometric composition Fe1-*x*S. He developed the so-called *Structure factor algebra* (Bertaut, 1957, 1959*a*,*b*).
He contributed enormously to the development of neutron
crystallography. He extended the use of group theory in
crystallography, particularly to magnetic structures (Bertaut, 1968). When the IUCr decided to finalize the Symmetry Group International Tables, he belonged to the *ad hoc* committee and particularly contributed to the definition of magnetic groups (Bertaut & Wondrastschek, 1971).
He used the group theory to anticipate all the magnetic structures
compatible with the crystal symmetries. This `Bertaut method' was very
useful for complex structures and even more so with the lack of
computers. Of course his students had to apply this method in any case.
In parallel, he did not forget his chemistry background and in
his two laboratories chemistry syntheses continuously fed
crystallography studies, mainly for a better understanding of magnetism.

Félix
Bertaut and his laboratories became internationally renowned in
crystallography, in neutron diffraction and in magnetism. The first
International Conference on Neutron Scattering was held in Grenoble in
1963. It was at the banquet of this conference that the construction of
a European high-flux neutron reactor was first suggested by Neel in a
speech prepared by Félix Bertaut. Then, with the support of Louis Neel,
Félix Bertaut promoted this project and convinced his German
collaborators. He was certainly convincing, and this was a good idea at
the right time. The French and German peoples, under the leadership of
de Gaulle and Adenauer, were re-establishing new friendly relationships
at this time. Thus the high-flux neutron reactor was created. As
Grenoble was considered as a major place in magnetism with Neel and a
neutron diffraction pole with Bertaut, this institute was built in
Grenoble. Its name, Institut Laue-Langevin (ILL), nicely expresses the
association of crystallography and magnetism. Initially French-German,
the ILL became European and was a key partner for building the European
Synchrotron Facility in Grenoble. This synchrotron radiation source is
particularly used for crystallography and also for magnetic
nanostructure studies. Seeds scattered by Neel, Bertaut and their
colleagues are now springing up.

The scientific renown of Félix
Bertaut is international. He contributed largely to the IUCr committees
like the International Tables Commission and the Neutron Diffraction
Commission. He was the chair of the Charge, Spin and Momentum Density
Commission (1975-1978), the IUCr representative to the IUPAP Commission
on Solid State (1966-1972) and a Member of the IUCr Executive Committee
(1975-1981). He was the editor or a co-editor of numerous scientific
revues. From 1958 to 1982 he was a scientific advisor of various
institutes (CEA, CNRS, ILL and Max Planck Institut, Stuttgart). Awarded
several prizes and acknowledged as Professor Honoris Causa of various
Universities, he was appointed full member of the French Academia of
Sciences in 1979.

### References

Bertaut, E. F. (1950). *Acta Cryst.* **3**, 14-18.    
Bertaut, E. F. (1952). *Acta Cryst.* **5**, 117-121.    
Bertaut, E. F. (1953). *Acta Cryst.* **6**, 557-561.    
Bertaut, E. F. (1956). *Acta Cryst.* **9**, 769-770.    
Bertaut, E. F. (1957). *Acta Cryst.* **10**, 606-607.    
Bertaut, E. F. (1959*a*). *Acta Cryst.* **12**, 541-549.    
Bertaut, E. F. (1959*b*). *Acta Cryst.* **12**, 570-574.    
Bertaut, E. F. (1968). *Acta Cryst.* A**24**, 217-231.    
Bertaut, E. F. & Forrat, F. (1956). *C. R. Acad. Sci. (Paris)*, **242**, 382.   
Bertaut, E. F. & Wondrastschek, H. (1971). *Acta Cryst.* A**27**, 298-300.    
Guinier, A. (1969-1972). Past President of the IUCr.  
Kastler, A. (1994). Nobel Prize in Physics *for the discovery and development of optical methods for studying hertzian resonances in atoms*.  
Neel, L. (1932). *Ann. Phys. (Paris)*, **17**, 5.  
Neel, L. (1936). *Ann. Phys. (Paris)*, **5**, 232.   
Neel, L. (1970). Nobel Prize in Physics *for
fundamental work and discoveries concerning antiferromagnetism and
ferrimagnetism which have led to important applications in solid state
physics*.  
Pauthenet, R. & Blum, P. (1954). *C. R. Acad. Sci. (Paris)*, **239**, 33.   
Shull, C. G. (1994). Nobel Prize in Physics *for the development of the neutron diffraction technique*.  
Shull, C. G. & Smart, J. S. (1949). *Phys. Rev.* **76**, 1256.

---

*J. Appl. Cryst.* (2004). **37**, 349-350   [ doi:10.1107/S0021889804004376 ]
