# Supplementary material for: Space groups and crystallographic symmetry: writing a multi-featured tutorial in a new style
Source: Acta Crystallogr E Crystallogr Commun. 2021 Jul 16;77(Pt 9):857–63. doi: 10.1107/S2056989021007039 (PMC8423017; doi:10.1107/S2056989021007039)
Supplement: Supplementary file 1 [file e-77-00857-sup2.zip › symandsg/Main/bodyo-gif.html]

Draft


# Orthorhombic lattices

|  |
| --- |
| a1 ≠ a2 ≠ a3α = β = γ = 90o |

|  |  |
| --- | --- |
| iodine - simple orthorhombic | |
| Click to see animation | Click to see animation |

 

|  |  |
| --- | --- |
| Base centered orthorhombic | |
| Click to see animation | Click to see animation |

 

|  |  |
| --- | --- |
| Body centered orthorhombic | |
| Click on the picture to repeat animation  Go back | Click to see animation |

 

|  |  |
| --- | --- |
| Face centered orthorhombic | |
| Click to see animation | Click to see animation |
