# Supplementary material for: Space groups and crystallographic symmetry: writing a multi-featured tutorial in a new style
Source: Acta Crystallogr E Crystallogr Commun. 2021 Jul 16;77(Pt 9):857–63. doi: 10.1107/S2056989021007039 (PMC8423017; doi:10.1107/S2056989021007039)
Supplement: Supplementary file 1 [file e-77-00857-sup2.zip › symandsg/Main/boese.htm]

Prof. Roland Boese

**Home****][Group][Contact][Publications][Research][
Lectures][Equipment][?**

|  |  |  |
| --- | --- | --- |
|  |  | **Prof. Dr. Roland Boese****][Coworkers & Previous Coworkers** |
|  |  | ***Prof. Dr. Roland Boese*** |
|  |  | *FB 8 / Anorganische Chemie  Universit�t Essen  Universit�tsstr. 5  45117 ESSEN   **Tel.:** ++49 (0)201/183-2416  **Fax:** ++49 (0)201/183-2535   **Room:** S03 V02 F43   roland.boese@uni-essen.de* |
|  |  | **Roland Boese was born in 1945 in Lichtenfels/Main (South Germany) and raised in Bremen, studied chemistry at the University of Marburg and received his PhD in 1976 in Inorganic Chemistry. After a postdoc year at the same University in the programme 'structure and the chemical bond, he moved 1977 to the new founded University of Essen, built up the division for Structural Chemistry, did his 'Habilitation' in 1991 and is in the position of a Professor.**   ****As an X-ray Crystallographer he authored and coauthored more than** 570 papers and review articles**, on boron compounds, metalorganic complexes and highly strained hydrocarbons. His recent research interest is focused on crystal engineering, polymorphism and the understanding of solid state properties based on crystal packing. Crystallisation and inhibition of crystallisation mostly of clathrates and cocrystallisates of organic compounds (gas hydrates) are now in the center of various projects.****   ****With several crystallisation techniques, mainly for highly sensitive and/or low melting compounds, low temperature techniques and high resolution X-ray structure determinations he established fruitful and enjoyable cooperations with preparative chemists on an international basis.****   ****He is married, has two** son**s and he is a vegetarian. Since he recently started piloting with an ultralight aircraft again, he quit smoking but continues to collect all kind of drinks from all over the world for** his private bar**.**** |

|  |
| --- |
|  |
