# Supplementary material for: Space groups and crystallographic symmetry: writing a multi-featured tutorial in a new style
Source: Acta Crystallogr E Crystallogr Commun. 2021 Jul 16;77(Pt 9):857–63. doi: 10.1107/S2056989021007039 (PMC8423017; doi:10.1107/S2056989021007039)
Supplement: Supplementary file 1 [file e-77-00857-sup2.zip › symandsg/Main/bravais_aug.htm]

Auguste BRAVAIS (1811-1863)

# Portraits d'Ardèchois

# Auguste BRAVAIS 1811-1863, Physicien, astronome et minéralogiste

Auguste
Bravais fut un grand savant qui se distingua autant en mathématiques
que dans les sciences physiques, il est réputé notamment pour ses
travaux en cristallographie (les réseaux de Bravais).

Auguste
Bravais est né à Annonay le 23 août 1811. Son père François-Victor
(1764-1852) était médecin et botaniste passionné (on lui doit
l’introduction de la culture du Dalhia en France); sa mère est
Aurélie-Adélaïde Thomé (1774-1814). Il fit ses études au Collège
Stanislas à Paris puis intégra l'École Polytechnique en 1829.
Continuant à travailler les sciences naturelles et les mathématiques,
il fut reçu au Doctorat de Sciences à l'Université de Lyon en 1837

D'une très grande curiosité, on lui doit de nombreux mémoires : - niveau de la mer - Phénomènes crépusculaires - Mouvements propres du soleil - Aphélies
- Arc en ciel Blanc - Halos et phénomenes optiques qui les accompagnent
- Influence de la rotation de la terre sur le pendule conique ainsi que
plusieurs études botanniques en collaboration avec son frère Louis.

Il est Officier de marine à la fin de ses études.

### Mission avec la Marine

Grand
aventurier, il embarqua sur le "Finistère" en 1832, puis sur le
"Loiret" et coopéra à des travaux d'hydrographie le long des côtes
algériennes.

A la tête de 37 marins, le Lieutenant de
vaisseau Bravais enleva aux cavaliers d’Abdel Kader, deux de leurs
prisonniers, le Commandant et le Chirurgien du "Loiret". Pendant ses
voyages comme pendant ses congés, Auguste Bravais continuait à
travailler les mathématiques et les sciences naturelles.

Le
ministre de la marine attacha Auguste Bravais à la Commission
Scientifique du Nord et le désigna pour embarquer avec Charles Martins
sur la Corvette "La Recherche" sur laquelle il fit plusieurs voyages
d’étude, notamment au Spitzberg et en Laponie au secours de la Lilloise
en 1835 et 1836. Blessé à la jambe, il fut obligé d’hiverner en Laponie
où il compléta ses travaux sur les phénomènes crépusculaires, les
halos, les aurores boréales en 1838 et 1839. En 1841, il publie avec C.
Martins, un compte-rendu d'une étude ayant pour objet la fiabilité des
Baromètres. Il fut l'un des plus grands spécialistes des aurores
boréales.

### Expédition au Mont-Blanc

Monsieur
Villemain Ministre de l’instruction publique obtint du parlement en
1844 après une intervention d’Arago, en faveur d’Auguste Bravais,
d’organiser une expédition scientifique au Mont-Blanc pour y compléter
les observations de Saussure. Ses recherches furent consacrées à
l'optique des phénomenes atmosphériques notamment aux parélies et aux halos.
Auguste Bravais, Charles Martins et le Docteur Lepileur désignés à cet
effet, mais après plusieurs tentatives, passèrent plusieurs jours sur
la montagne dont une journée au sommet même du Mont-Blanc où ils firent
de nombreuses observations scientifiques, sur les lois de la pesanteur,
controlées à la station d'expériences de Chamonix par l’Abbé Camille
Bravais, frère d'Auguste.

### Enseignant

Il
fut nommé professeur de mathématiques appliquées à l'astronomie à la
Faculté des Sciences à Lyon à partir de 1840. Puis, il succéda à Lainé
à la chaire de physique de l'École Polytechnique entre 1845 et 1856,
date à laquelle il fut remplacé par Hureau de Sénarmont.

Co-fondateur
de la Société Météorologique, il est élu membre de l'Académie des
sciences en 1854, où il succéda à Roussin. Il fut élevé au grade
d'Officier de la légion d'Honneur.

Malade, il dût cesser toute activité à partir de 1856.

## Cristallographie

La
cristallographie est la science qui se consacre à l'étude des
substances cristallines à l'échelle atomique. L'arrangement spatial des
atomes dans la matière est étroitement lié à ses propriétés. L'état
cristallin est défini par un caractère périodique et ordonné à
l’échelle atomique ou moléculaire. Ce caractère périodique est appelé
la maille élémentaire. A. Bravais publia un mémoire traitant de
cristallographie en 1847.

### Réseaux de Bravais

Auguste
Bravais s'intéressa à l'étude des formes externes des cristaux et à
celle de leur structure interne et formula l'hypothèse de la structure
réticulaire des cristaux.

Il définit de façon rigoureuse,
en 1848, à partir des différentes combinaisons des éléments de symétrie
cristalline, 32 classes de symétrie, qui elles-mêmes se répartissent en
14 types de réseaux (il n'existe pas d'autre façon de disposer des
points dans l'espace, afin de réaliser un réseau ou une maille, de
manière à ne laisser aucun volume libre entre les réseaux). Les 14 réseaux de Bravais sont des expansions des 7 formes primitives de cristaux (les systèmes cristallins) .

C'est
à cette époque qu'il énonce sa théorie du réseau cristallin périodique
qui complète la théorie d'Haüy. Il définit un réseau comme étant un
modèle théorique de cristal, qui serait une structure infinie
constituée par la répétition, par la translation dans trois directions
de base, d'un motif élémentaire.

Bravais a calculé toutes
les circonstances du phénomène; au moyen de ses ingénieux appareils. Il
est également parvenu à donner la reproduction artificielle des
cercles, ainsi que celui des anthélies et de leurs arcs dans une
chambre obscure au moyen d'un prisme en glace qu'il faisait tourner
très rapidement en y projetant les rayons solaires.

L'hypothèse
émise par Bravais en 1849 de la structure réticulaire des cristaux fut
vérifiée en 1912 grâce à la diffraction des rayons X

### L'ombre des montagnes

Lorsqu'on se trouve placé au sommet d'une très haute montagne, l'ombre
que projette le Soleil, à son coucher, se dirige vers le ciel, et
produit quelquefois un magnifique phénomène, observé par Bravais et
Martins, dans une de leurs excursions au Mont-Blanc. Bravais en a donné
la description suivante :

"Le
soleil approchant de l'heure de son coucher, nous jetâmes les yeux du
côté opposé à l'astre, et nous aperçûmes, non sans quelque étonnement,
l'ombre du Mont-Blanc qui se dessinait sur les montagnes couvertes de
neige de la partie est de notre panorama. Elle s'éleva graduellement
dans l'atmosphère, où elle atteignit la hauteur d'un degré, restant
encore parfaitement visible.  
 L'air, au-dessus du cône d'ombre,
était teint de ce rose pourpre que l'on voit, dans les beaux couchers
de soleil, colorer les hautes cimes; le bord de cette teinte offrait
une zone plus intense, et cette bordure continue rehaussait l'éclat du
phénomène".

"Que
l'on imagine maintenant les montagnes de la grande vallée d'Aoste
projetant, elles aussi, à ce même moment, leur ombre dans l'atmosphère,
la partie inférieure sombre avec un peu de verdâtre, et au-dessus de
chacune de ces ombres la nappe rose purpurine avec la ceinture rose
foncée qui la séparait d'elles; que l'on ajoute à cela la rectitude du
contour des cônes d'ombre, principalement de leur arête supérieure, et
enfin les lois de la perspective faisant converger toutes ces lignes
l'une sur l'autre, vers le sommet même de l'ombre du Mont-Blanc,
c'est-à-dire au point du ciel où les ombres de nos corps devaient être
placées, et l'on n'aura encore qu'une idée incomplète de la richesse du
phénomène météorologique qui se déploya pour nous pendant quelques
instants. Il semblait qu'un être invisible était placé sur un trône
bordé, de feu, et que, à genoux, des anges aux ailes étincelantes
l'adoraient, tous inclinés vers lui. A la vue de tant de magnificence,
nos bras et ceux de nos guides restèrent inactifs, et des cris
d'enthousiasme s'échappèrent de nos poitrines. J'ai vu les belles
aurores boréales du Nord [sic] avec leurs couronnes zénithales aux
colonnes diaprées et mobiles, que nos plus beaux feux d'artifice ne
sauraient égaler par leurs effets; mais la vue de l'ombre du mont Blanc
me paraît plus grandiose encore".

### Formes symétriques des flocons

Dès
1623, après une chute de neige, Peiresc observe au microscope que les
flocons ont des formes toujours symétriques, comme une étoile à six
branches, avec des facettes hexagonales réfléchissantes. C'est le point
clé de l'explication des parhélies pressentie par Peiresc et Gassendi.
C'est seulement au XIXe siècle qu'Auguste Bravais,
professeur à l'École polytechnique, le démontrera. Les arcs-en-ciel
sont dus à des gouttelettes d'eau en suspension dans l'air, à faible
altitude. Les phénomènes de parhélies sont dus à la présence de
cristaux de glace ou de neige dans la haute atmosphère. Les
arcs-en-ciel sont des phénomènes de réfraction qui donnent des teintes
"irisées" avec de belles couleurs. Les parhélies sont obtenues par
réflexion sur les faces planes des cristaux de glace qui agissent comme
de petits miroirs. Ce sont des phénomènes "blancs". Les parhélies sont
des phénomènes très lumineux. Les faux soleils peuvent être aussi
lumineux que le vrai. Les parhélies sont très spectaculaires mais très
rares.

Auguste Bravais avait épousé en
1847, Antoinette Moulie de Paris, dont il eut un fils unique mort en
bas âge, douloureusement frappé par cette perte cruelle, il travailla
nuit et jour, tomba gravement malade et mourut à Versailles en 1863. Sa
veuve embrassa la vie religieuse au couvent des Clarisses de
Versailles, consacra sa fortune à cet ordre et y termina ses jours le
11 Février 1885 comme Vicaire de l’abbesse .

Auguste Bravais est décédé le 30 mars 1863, au Chesnay. Son buste figure en mairie d'Annonay.

Fragment de l'Éloge historique
d'Auguste Bravais, lu devant l'Académie des Sciences, dans sa séance
publique du 6 février 1865 par M. Elie de Beaumont , Secrétaire
perpétuel.

N.B.: Si vous avez des renseignements complémentaires sur la biographie de ce personnage merci de me les communiquer à Medarus

### Publications:

- Auguste Bravais, 1847, Mémoire sur les halos, Journal de l'école polytechnique, XXXIe cahier.

- Auguste Bravais : de la Laponie au Mont-Blanc

- Etudes Cristallographiques par A. Bravais, Lieutenant de vaisseau, Professeur à l'École Polytechnique.

- Accueil en Ardèche
- Accueil Medarus
- Commentaires

  

  


Mesure d'audience ROI frequentation par
