# Supplementary material for: Space groups and crystallographic symmetry: writing a multi-featured tutorial in a new style
Source: Acta Crystallogr E Crystallogr Commun. 2021 Jul 16;77(Pt 9):857–63. doi: 10.1107/S2056989021007039 (PMC8423017; doi:10.1107/S2056989021007039)
Supplement: Supplementary file 1 [file e-77-00857-sup2.zip › symandsg/Main/bravais_aug_files/bravais.htm]

403 Forbidden

# Forbidden

You don't have permission to access /Ardeche/07celebr/07celImg/bravais.gif
on this server.

Additionally, a 404 Not Found
error was encountered while trying to use an ErrorDocument to handle the request.

---

Apache/1.3.34 Server at www.medarus.org Port 80
