# Supplementary material for: Space groups and crystallographic symmetry: writing a multi-featured tutorial in a new style
Source: Acta Crystallogr E Crystallogr Commun. 2021 Jul 16;77(Pt 9):857–63. doi: 10.1107/S2056989021007039 (PMC8423017; doi:10.1107/S2056989021007039)
Supplement: Supplementary file 1 [file e-77-00857-sup2.zip › symandsg/Main/bravaisong.htm]

The Bravais Lattices Song

|  |
| --- |
| The Bravais Lattices Song  by Walter Fox Smith  Tune: "I Am the Very Model of a Modern Major General",   from *"The Pirates of Penzance"*, by William Gilbert & Arthur Sullivan  Lyrics: Web Page .. Word Format .. PDF    recording mp3 ------ Real Audio  Piano: Bruce Morrison .... Chorus: Marian McKenzie, Michael K. McCutchan, Faith H. McKenzie  RealAudio format is faster for modem downloads,  mp3 format is slightly higher audio quality.    Background courtesy of Free Backgrounds.com |
