# Supplementary material for: Space groups and crystallographic symmetry: writing a multi-featured tutorial in a new style
Source: Acta Crystallogr E Crystallogr Commun. 2021 Jul 16;77(Pt 9):857–63. doi: 10.1107/S2056989021007039 (PMC8423017; doi:10.1107/S2056989021007039)
Supplement: Supplementary file 1 [file e-77-00857-sup2.zip › symandsg/Main/Buerger.html]

M.J. Buerger Award

|  |  |  |  |  |  |  |  |  |  |  |  |  |  |  |  |  |  |  |  |  |  |  |  |  |  |  |  |  |  |  |  |  |  |  |  |  |  |  |  |  |  |  |  |  |  |  |  |  |  |  |  |  |  |  |  |  |  |  |  |  |  |  |  |  |  |  |  |  |  |  |  |  |  |  |  |  |  |  |  |  |  |  |  |  |  |  |  |  |  |  |  |  |  |  |  |  |  |  |  |  |  |  |  |  |  |  |  |  |  |  |  |  |  |  |  |  |  |  |  |  |  |  |  |  |  |  |  |  |  |  |  |  |  |  |  |  |  |  |  |  |  |  |  |  |  |  |  |  |  |  |  |  |  |  |  |  |  |  |  |  |  |  |  |  |  |  |  |  |  |  |  |  |  |  |  |  |  |  |  |  |  |  |  |  |  |  |  |  |  |  |  |  |  |  |  |  |  |  |  |  |  |  |  |  |  |  |  |  |  |  |  |  |  |  |  |  |  |  |  |  |  |  |  |  |  |  |  |  |  |  |  |  |  |  |  |  |
| --- | --- | --- | --- | --- | --- | --- | --- | --- | --- | --- | --- | --- | --- | --- | --- | --- | --- | --- | --- | --- | --- | --- | --- | --- | --- | --- | --- | --- | --- | --- | --- | --- | --- | --- | --- | --- | --- | --- | --- | --- | --- | --- | --- | --- | --- | --- | --- | --- | --- | --- | --- | --- | --- | --- | --- | --- | --- | --- | --- | --- | --- | --- | --- | --- | --- | --- | --- | --- | --- | --- | --- | --- | --- | --- | --- | --- | --- | --- | --- | --- | --- | --- | --- | --- | --- | --- | --- | --- | --- | --- | --- | --- | --- | --- | --- | --- | --- | --- | --- | --- | --- | --- | --- | --- | --- | --- | --- | --- | --- | --- | --- | --- | --- | --- | --- | --- | --- | --- | --- | --- | --- | --- | --- | --- | --- | --- | --- | --- | --- | --- | --- | --- | --- | --- | --- | --- | --- | --- | --- | --- | --- | --- | --- | --- | --- | --- | --- | --- | --- | --- | --- | --- | --- | --- | --- | --- | --- | --- | --- | --- | --- | --- | --- | --- | --- | --- | --- | --- | --- | --- | --- | --- | --- | --- | --- | --- | --- | --- | --- | --- | --- | --- | --- | --- | --- | --- | --- | --- | --- | --- | --- | --- | --- | --- | --- | --- | --- | --- | --- | --- | --- | --- | --- | --- | --- | --- | --- | --- | --- | --- | --- | --- | --- | --- | --- | --- | --- | --- | --- | --- | --- | --- | --- | --- | --- | --- | --- | --- | --- | --- | --- | --- | --- | --- | --- | --- |
| |  |  |  |  |  |  |  |  |  |  |  |  |  |  | | --- | --- | --- | --- | --- | --- | --- | --- | --- | --- | --- | --- | --- | --- | |  | | | | | | | | | | | | |  | | |  |  |  |  |  | | --- | --- | --- | --- | --- | |  |  |  |  |  | | | | | | | | | | | | | |  | |  |  | |  |  |  |  | | --- | --- | --- | --- | |  |  |  |  | |  | | |  | |  |  |  |  | |  |  | | | | | |  | M.J. Buerger Award | | | | |  | |  |  |  |  | | **Next Award   2006**       - September 2004  Call for Nominations - April 1, 2005     Closing date for submission nomination - July 2006           Presentation of Award at Annual Meeting | | |  | |  |  |  |  |  |  |  |  |  | |  | |  |  |  |  | | --- | --- | --- | --- | |  |  |  |  | |  | | |  | |  |  |  |  | |  |  | | | | | |  |  |  | |  |  |  |  |  |  |  |  |  | |  |  |  | Awards & Prizes  Quick Links  - Upcoming Awards & Deadlines  - Nomination Guidelines  - Awards LIst  - Poster Prizes  - Schedule  - Previous Winners | |  |  |  |  | |  |  |  |  |  |  | To recognize mature scientists who have made contributions of exceptional distinction in areas of interest to the ACA. There are no restrictions as to nationality, race, sex, religion, or membership in the ACA. Awarded triennially in memory of Martin J. Buerger, Institute Professor Emeritus of M.I.T. and University Professor Emeritus of the University of Connecticut, a mineralogist who made major contributions to many areas of crystallography. Established in 1983. The first award was made in 1985. A monetary award of $1,500, and up to $1,500 in travel expenses to accept award at Annual Meeting.  **Recipient of 2003 Award**  2003  **Jim Ibers** - *Less Difficult But Still Not Easy*  **Past Recipients** | | | | |  | |  |  |  |  |  |  |  |  |  | |  |  |  |  |  |  |  |  | Other Awards: | | | | |  | |  |  |  |  |  |  |  |  | - Warren Award | - Buerger Award | | - Etter Award | - Fankuchen Award |  | |  |  |  |  |  |  |  |  | - Patterson Award | - Wood Award | | - Trueblood Award | - Supper Award | | | |  |  | | --- | --- | |  |  | | | | |  |  |  |  | - Service Awards | | | | |  | |  |  |  |  |  |  |  |  |  | |  | | | | | | | | | | | | |  | |  |  |  |  |  |  |  |  |  |  |  |  |  |  | |
