# Supplementary material for: Space groups and crystallographic symmetry: writing a multi-featured tutorial in a new style
Source: Acta Crystallogr E Crystallogr Commun. 2021 Jul 16;77(Pt 9):857–63. doi: 10.1107/S2056989021007039 (PMC8423017; doi:10.1107/S2056989021007039)
Supplement: Supplementary file 1 [file e-77-00857-sup2.zip › symandsg/Main/CAB.html]

Cecil Arnold Beevers

## Obituary of Cecil Arnold Beevers (1908 -2001)

With the death of Dr C. Arnold Beevers, Reader Emeritus in Crystallography
at the University of Edinburgh on 16 January, 2001, we have said
farewell to a great man, who was scientist, teacher, inventor, humanitarian
and humorist in a rare combination. Arnold was born in Manchester on 27
May, 1908, but his family moved shortly thereafter to Liverpool, a city of
which he was always proud. His father was a master tradesman, whose work
included the installation of the glass roof of the Lever factories. He
obtained a B.Sc. in Physics from the University of Liverpool in 1929, and a
D.Sc. in 1933. While there, he was greatly influenced by Professor Lionel
Wilberforce, whose well-designed springs and clamps Arnold never tired
of demonstrating. After graduation, he was asked to set up work in the
relatively new subject of X-ray diffraction together with Henry Lipson, a
colleague and friend for many years. The two of them made frequent trips
(sometimes by bicycle) to the University of Manchester to get advice from
Lawrence Bragg, and Arnold eventually moved to a post there. After a short
appointment at Hull, Arnold was appointed in 1938 to the Dewar Fellowship in
Crystallography. This was a post offered jointly by the Departments of
Physics and Chemistry at the University of Edinburgh, and he was elected
Fellow of the Royal Society of Edinburgh later that year. And in Edinburgh
he remained, coming into his office in the Chemistry Department for the last
time less than two months before he died.

As a young adult, Arnold, like Kathleen Lonsdale, felt drawn to join the
Religious Society of Friends (Quakers) and his deep faith in and with that
body greatly influenced his approach to science and to life. He was
unswervingly committed to humanitarian causes, and in particular to world
peace, and he strongly disliked any form of superstition or other
obscurantism, and any sort of pomposity. His life was touched by tragedy,
especially the severe dementia which overtook his wife Marjorie in the late
1950's, and which grew ever worse until her death in 1992. Sadly, a
similar problem affected Yvonne, his second wife, who died in 1998. In
these circumstances, his never-failing sense of humour was all the more
remarkable.

Arnold's scientific contributions were many. From his early days, he is
most remembered now for the Beevers-Lipson strips, and the technique by
which they reduced the huge calculations of three-dimensional Fourier
summations to sums of manageable one-dimensional ones. These attractive
boxes of strips of card were produced in great quantity until the digital
computer became generally available in the late 1960's. The technique is
still used in many computer programs. Arnold's earliest structural
publication was a correct and beautiful reinterpretation of the structure of
beryllium sulfate tetrahydrate as interpenetrating tetrahedra of
Be(H2O)42+ and
SO42- ions. Previously, a remarkably imprecise
treatment had found that the sulfate ions were planar! Arnold rapidly
became involved in determinations using Fourier methods and the new
Patterson method; important examples were the alums, copper sulfate
pentahydrate, and particularly the so-called b-alumina, NaAl11O17, which he
published with the late Dr Marion Ross. Originally studied as a troublesome
impurity in Al2O3 production, it is now an important
solid-state ionic conductor, and two sites in the structure are known as
Beevers-Ross and anti-Beevers-Ross sites.

Arnold's arrival in Edinburgh was soon followed by the outbreak of the
Second World War. As a conscientious objector, he was sent to assist
Professor Norman Dott at the Western General Hospital in his work on
electroencephalography. Arnold threw himself enthusiastically into this
work, particularly as it threw light on the nature of sleep, and this
interest remained with him for the rest of his life.

After the war, Arnold joined the Department of Chemistry as a member of
staff, and built up an X-ray diffraction laboratory there, mainly with
equipment he had designed himself. His generators and cameras were
characteristically robust and precisely engineered. The arrival of Professor
E.L. Hirst and the growth of his carbohydrate group encouraged Arnold to
investigate the then daunting structures of sugars, and ones he studied
included glucose, and sucrose in the form of its sodium bromide adduct.

As a teacher, Arnold gave lectures of a highly individual nature, which have
remained in the memories of generations of students. As an experimenter, he
delighted in demonstrations, although they did not always behave for him.
One lecture ended uproariously as an explosion he had attempted to
demonstrate finally worked in the hands of the technician who was removing
the apparatus. Happily, no serious damage was done to the technician or
anyone else! Younger colleagues were surprised (to say the least) to see
Arnold showing the bones of his hand to visitors by using the direct beam
from the lab X-ray generators. Again, he always seemed to get away with it.
His crystallography lectures generally featured safer equipment, including
the collection of plasticene elephants he used to occupy the sites of a
lattice. He also had a large assortment of collecting boxes for Dr
Barnardo's Homes for this purpose, frequently pointing out that this was an
excellent charity and that the boxes had another useful function! He was
honoured one year by a song from the honours students, showing that they had
learned at least something from him:

*Twinkle, twinkle, little star  
How I wonder how you are?  
For you have five points, I see,  
And I know that cannot be.  
You must be point group 33  
A new system of symmetry!*

Possibly his most lasting impact as a teacher was his teaching of
chemistry for dental students over many years. In 1946, he published an
elegant interpretation of the structure of fluorapatite, the ideal bone
and teeth mineral, showing the function of the fluoride ions in holding
it together. Thereafter he became devoted to the cause of improving
dental health by adding fluoride to drinking water supplies which were
deficient in it. He often spoke at rallies on this subject, cheered on
by his students, and was once caught in a photograph by the Scotsman
with raised fist, in a most unpacific manner! When he retired in 1978,
the Edinburgh University Dental Society gave him a life membership of
which he was always very proud.

Arnold was for many years active in the Edinburgh Cripple Aid
Society. He was in much demand as a helper, particularly as Master of
Ceremonies at plays and concerts. An epileptic seizure affecting a
leading lady never fazed him, and he could immediately take over, often
getting the audience engaged in community singing. He displayed this
ability equally at international scientific meetings. Particularly
famous at home and abroad was his version of "My Bonnie lies over the
Ocean" in which the singers must stand up or sit down on each word
beginning with "b". Generally, by the third chorus, even a group of
scientists were coping with all the "bring backs"! At one international
school, he produced a song for the final dinner, which took place after
a particularly long and boring lecture. At the end of the song, Arnold
remarked brightly, as no one else would have dared, "I wrote that in
Professor H\*\*\*\*'s lecture this morning. Had he gone on any longer,
there would have been even more verses!"

The involvement with handicapped people was very important in
Arnold's last major scientific contribution, what are now known as
Beevers Miniature Models. Arnold moved away from structure
determination after 1960, as he was never really happy with the advent
of the digital computer; for the first time, he was being asked to use
equipment which he could not fully comprehend and design himself. He
sensed the need for accurate ball-and-spoke models on a scale much
smaller than that of the inch to �ngstrom models then available, and
developed precision drilling machines to enable the scale to be reduced
to 1 cm per �ngstrom. After some experimentation, he fixed on the 7mm
perspex spheres and 1mm steel rod now used, being determined that the
models must be both accurate and elegant. Typically, he published his
methods and would have no truck with the idea of a patent, which he saw
as unfairly denying public access to his ideas and methods. The
calculations required for drilling, he carried out manually, using a
Wulff net for his stereographic projections. From the beginning, much
of the work was carried out by handicapped workers, including at one
time Brian Wilson, the world's longest surviving patient on renal
dialysis (as listed in the Guinness Book of Records!). These workers
clearly took great pride in their work, which have gone all over the
world, to institutions ranging from a school in Port Moresby, New
Guinea, to I.B.M. Research. Over the years, the Models were taken over
by the University, and Arnold accepted that lesser mortals would have
to use the computer even for drilling calculations. He was actually
pleased to see himself eventually on a website!

The Beevers Miniature Models Unit continues, showing that he
was right that computer modelling would not replace completely the
elegant, permanent model in museums, teaching or research. Arnold is
survived by Lois and John, his daughter and son, by fourgrandchildren
and five great-grandchildren. Many former students and colleagues
world-wide have joined in sending them their sympathy and their happy
memories of a great and kind man.

**Robert and Sheila Gould**

Further information about Arnold Beevers

---

This file last updated  *19 Sept 2001*  
 BCA Education WebMaster
BCA@ISISE.RL.AC.UK


Click here to return to BCA homepage 
