# Supplementary material for: Space groups and crystallographic symmetry: writing a multi-featured tutorial in a new style
Source: Acta Crystallogr E Crystallogr Commun. 2021 Jul 16;77(Pt 9):857–63. doi: 10.1107/S2056989021007039 (PMC8423017; doi:10.1107/S2056989021007039)
Supplement: Supplementary file 1 [file e-77-00857-sup2.zip › symandsg/Main/cotton.htm]

Department of Chemistry at Texas A&M University - Frank A Cotton

|  |  |  |  |  |  |  |  |  |  |  |  |  |  |  |  |  |  |  |  |  |  |  |  |
| --- | --- | --- | --- | --- | --- | --- | --- | --- | --- | --- | --- | --- | --- | --- | --- | --- | --- | --- | --- | --- | --- | --- | --- |
| |  | | --- | | WELCOME | |  | | PEOPLE | |  | | RESEARCH | |  | | GRADUATE PROGRAM | |  | | UNDERGRADUATE PROGRAM | |  | | NSF-REU PROGRAM | |  | | SEMINARS and COLLOQUIA | |  | | INDUSTRIAL CONNECTIONS and RECRUITING | |  | | ADMINISTRATION | |  | | SAFETY | |  | | TEXAS A&M UNIVERSITY LIBRARIES and E-RESOURCES | |  | | CONTACT | |

|  |
| --- |
| Phonebook |
|  |
| Printable contact lists |
|  |
| Faculty |
|  |
| Graduate students |
|  |
| New graduate students |
|  |
| Staff |
|  |
| Research staff |
|  |
| Research associates |

|  |
| --- |
| Faculty by name |
|  |
| Faculty by research division |
|  |
| Faculty by research interest/area |
|  |
| Emeritus Faculty |

|  |
| --- |
| Analytical Chemistry |
|  |
| Biological Chemistry |
|  |
| Inorganic Chemistry |
|  |
| Organic Chemistry |
|  |
| Physical/Nuclear Chemistry |

|  |
| --- |
| Atmospheric Chemistry |
|  |
| Bioanalytical Chemistry |
|  |
| Bioinorganic Chemistry |
|  |
| Bioorganic Chemistry |
|  |
| Catalysis |
|  |
| Chemical Education |
|  |
| Chemistry of Interfaces |
|  |
| Chemistry of Materials |
|  |
| Molecular Structure |
|  |
| Nuclear Chemistry |
|  |
| Chemistry of Polymers |
|  |
| Spectrosocpy and Dynamics |
|  |
| Supramolecular Chemistry |
|  |
| Synthesis |
|  |
| Theoretical Chemistry |

|  |
| --- |
| Faculty |
|  |
| Research Centers |
|  |
| Research Facilities |
|  |
| Supporting Facilities |
|  |
| Faculty awards |
|  |
| Journals edited |
|  |
| Sterling C. Evans Library |

|  |
| --- |
| Faculty by name |
|  |
| Faculty by research division |
|  |
| Faculty by research interest/area |

|  |
| --- |
| Analytical Chemistry |
|  |
| Biological Chemistry |
|  |
| Inorganic Chemistry |
|  |
| Organic Chemistry |
|  |
| Physical/Nuclear Chemistry |

|  |
| --- |
| Atmospheric Chemistry |
|  |
| Bioanalytical Chemistry |
|  |
| Bioinorganic Chemistry |
|  |
| Bioorganic Chemistry |
|  |
| Catalysis |
|  |
| Chemical Education |
|  |
| Chemistry of Interfaces |
|  |
| Chemistry of Materials |
|  |
| Molecular Structure |
|  |
| Nuclear Chemistry |
|  |
| Chemistry of Polymers |
|  |
| Spectroscopy and Dynamics |
|  |
| Supramolecular Chemistry |
|  |
| Synthesis |
|  |
| Theoretical Chemistry |

|  |
| --- |
| Within department |
|  |
| Outside department |

|  |
| --- |
| Center for Chemical Characterization and Analysis |
|  |
| Center for Integrated Microchemical Systems |
|  |
| Elemental Analysis Laboratory |
|  |
| EPR Instrument Facility |
|  |
| Mass Spectrometry Facility |

|  |
| --- |
| Cyclotron Institute |
|  |
| Electron Microscopy and Imaging Center |
|  |
| Gene Technology Laboratory |
|  |
| Protein Chemistry Laboratory |

|  |
| --- |
| Laboratory for Molecular Simulation |
|  |
| NMR |
|  |
| X-ray diffraction |

|  |
| --- |
| Information Technology |
|  |
| Electronics shop |
|  |
| Glass shop |
|  |
| Machine shop |
|  |
| Available Technologies (TLO) |

|  |
| --- |
| Overview of Ph.D. Program |
|  |
| Information for prospective students |
|  |
| Information for current students |
|  |
| On-line Application |
|  |
| Orientation schedule (PDF) |
|  |
| Special programs and training grants |

|  |
| --- |
| Graduate handbook |
|  |
| Graduate catalog |
|  |
| Course schedules |
|  |
| Graduate course web sites |
|  |
| Graduate student seminar abstracts |
|  |
| Office of Graduate Studies, TAMU |
|  |
| Adult, Graduate & Off Campus Student Services |
|  |
| International Student Services, TAMU |

|  |
| --- |
| Information for undergraduate chemistry majors |
|  |
| Transfer Course Matrix |
|  |
| Undergraduate catalog |
|  |
| Course schedules |
|  |
| Undergraduate course web sites and syllabi |
|  |
| First year chemistry program |
|  |
| Orbitals newsletter |
|  |
| ACS Directory of Graduate Research |

|  |
| --- |
| General Information |
|  |
| Degrees Offered |
|  |
| Implementing Your Degree Plan |
|  |
| Tracks and Minors |
|  |
| Declaration of Minor in Chemistry |
|  |
| Undergraduate Research |
|  |
| Advice Before Registering for Classes |
|  |
| Stay Bonded |
|  |
| Frequently Asked Questions |

|  |
| --- |
| NSF-REU Interdisciplinary Chemistry Research Program |
|  |
| NSF-REU Nuclear Physics and Nuclear Chemistry Program |

|  |
| --- |
| Department seminar calendar |
|  |
| Frontiers series |
|  |
| Inorganic seminars |
|  |
| Organic seminars |
|  |
| IUCCP |

|  |
| --- |
| IUCCP |
|  |
| Graduate student resumes |
|  |
| Recruiting schedule |
|  |
| Texas A&M Career Center |

|  |
| --- |
| Office of the Department Head |
|  |
| Business Office |
|  |
| Department of Chemistry committees (PDF) |
|  |
| Department of Chemistry bylaws |

|  |
| --- |
| Emergency response plan |
|  |
| MSDS |
|  |
| Laboratory safety checklist |
|  |
| Department safety regulations |
|  |
| Environmental health and safety |

|  |
| --- |
| Sterling C. Evans Library |

|  |
| --- |
| Office of the Department Head |
|  |
| Graduate program office |
|  |
| Undergraduate program office |
|  |
| First year chemistry program |
|  |
| Business office |
|  |
| Texas A&M Chapter of American Chemical Society |
|  |
| Map and directions |
|  |
| Parking |
|  |
| Webmaster |

Home       Phonebook       Email       Directions
       Calendar       Sitemap      Search

|  |  |
| --- | --- |
| **Contact Information:**  Department of Chemistry  Texas A&M University  College Station, TX 77842  Phone: (979) 845-4432  Fax: (979) 845-9351  cotton@tamu.edu | **F. Albert Cotton**   W. T. Doherty - Welch Foundation Chair   Distinguished Professor  Ph. D., Harvard University  **Awards:**   - Director, Laboratory for Molecular Structure and Bonding - Member, National Academy of Sciences - National Medal of Science - Welch Award - Priestley Medal   **Areas of Interest:**   - Inorganic Chemistry   **Cotton Research Group** |
| ---  **Current Activities**  Our research program derives its impetus from the exploration of how transition metals form compounds with metal-metal bonds. This leads us to examine compounds in which metal atoms form bonds of various orders within certain structural frameworks. A fascinating thing about this approach to transition metal chemistry is that, in addition to frequently yielding results of the desired type, it often leads to unexpected results involving compounds different from the original targets. We deal with virtually the entire range of metallic elements and with a vast array of main group and organic species as ligands.    At the heart of most projects in the group is synthetic chemistry aimed at making new kinds of bonds or molecules; the synthetic work carries straight into an extensive use of X-ray crystallography to identify the products and reveal their important features. Normally, each worker in the group carries out X-ray studies; our facilities (diffractometers and computational capabilities) make this feasible and efficient. In addition, virtually the whole panoply of modern physical methods comes into play, including nuclear magnetic resonance, UV-visible, infrared, CD, EPR, magnetic susceptibility measurement and photoelectron spectroscopy. Theoretical analysis by molecular quantum mechanics at several levels of rigor, up to X-SCF-SW and H-F methods, are used. In the M-M multiple bond field, our current interests are in some of the less explored areas, namely compounds containing VV, NbNb, TaTa, CoCo, RuRu, Ru=Ru, OsOs, Ir-Ir and various heteronuclear species (e.g., MoW), although we still find interesting problems involving multiple bonds between pairs of Cr, Mo, W and Re atoms. In the area of metal atom clusters, we continue to pursue the triangulo clusters of Mo and W, such as Mo3O4n+, W3S4n+ and Mo3O2n+, and cuboidal species such as Mo4S4n+ and W4S4n+. We find new and better ways to make many of these clusters, as well as introduce many sorts of ligands not previously prominent in this class of compounds. We also have found that we can make discrete cluster species of Nb and Ta that correspond to the repeat units in previously known solid state materials in which there are infinite sheet structures.   ---  **Selected Publications** "Reactions of TiCl4 with Phosphines and Alkylating Reagents: an Organometallic Route to a Titanium(II) Cluster Compound," F. A. Cotton, C. A. Murillo and M. A. Petrukhina. **J. Organomet. Chem.** 573, 78 (1999).  "A Chain of Five Chromium(II) Atoms: A Desired Compound with an Undesired, Unsurprising, but Important Structure." F. A. Cotton, L. M. Daniels, T. Lu, C. A. Murillo and X. Wang. **J. Chem. Soc., Dalton Trans.** 517 (1999).  "Square and Triangular Arrays Based on Mo24+ and Rh24+ Units." F. A. Cotton, L. M. Daniels, C. Lin and C. A. Murillo. **J. Am. Chem. Soc.** 121, 4538 (1999).  "The Designed 'Self-assembly' of a Three-dimensional Molecule Containing Six Quadruply-bonded Mo24+ Units." F. A. Cotton, L. M. Daniels, C. Lin and C. A. Murillo. **Chem. Comm.** 841 (1999).  "First paddlewheel complex with a doubly-bonded Ir26+ core." F. A. Cotton, C. A. Murillo and D. J. Timmons. **Chem. Commun.** 1427 (1999). | |

Department of Chemistry,
Texas A&M University  
PO Box 30012, College Station, TX 77842-3012  
(t) 979-845-2011
(f) 979-845-4719

---

Privacy Statement | State
of Texas | State Wide Search | Texas
A&M University | Webmaster
