# Supplementary material for: Space groups and crystallographic symmetry: writing a multi-featured tutorial in a new style
Source: Acta Crystallogr E Crystallogr Commun. 2021 Jul 16;77(Pt 9):857–63. doi: 10.1107/S2056989021007039 (PMC8423017; doi:10.1107/S2056989021007039)
Supplement: Supplementary file 1 [file e-77-00857-sup2.zip › symandsg/Main/curtin2.htm]

David Y. Curtin and
Iain C. Paul  
  

1. "Loss of solvent from a molecular crystal solvate.
   Phenylazotribenzoylmethane hemietherate," McCullough, J. D.; Curtin, D.
   Y.; Miller, L. L.; Paul, I. C.; Pendergrass, D. B., Jr. *Mol. Cryst.
   Liq. Cryst.* **1970**, *11*, 407-412.
2. "Crystal structure of nitrosobenzene dimer. cis-Azobenzene
   dioxide," Dieterich, D. A.; Paul, I. C.; Curtin, D. Y. *J. Chem.
   Soc., Chem. Commun.* **1970**, 1710-1711.
3. "Reaction of ammonia gas with crystalline benzoic and related
   acids," Curtin, D. Y.; Miller, R. S.; Paul, I. C. *J. Am. Chem. Soc.*
   **1971**, *93*, 2784-2786.
4. "Crystal and molecular structures of diphenyl triketone
   sym-N-benzoylphenylhydrazone and diphenyl triketone
   sym-N-benzoyl-p-bromophenylhydrazone," Pendergrass, D. B., Jr.; Paul,
   I. C.; Curtin, D. Y. *J. Am. Chem. Soc.* **1972**, *94*,
   8730-8737.
5. "X-ray crystal structure and solid state rearrangement of
   phenylazotribenzoylmethane and the x-ray crystal structure of
   a-p-bromophenylazo-b-banzoyloxybenzalacetophenone," Pendergrass, D. B.,
   Jr.; Curtin, D. Y.; Paul, I. C. *J. Am. Chem. Soc.* **1972**,
   *94*, 8722-8730.
6. "Reaction of ammonia gas with crystalline substituted benzoic
   anhydrides," Miller, R. S.; Curtin, D. Y.; Paul, I. C.; Urbana *J.
   Am. Chem. Soc.* **1972**, *94*, 5117-5119.
7. "Beckmann-Chapman rearrangement in the solid state of oxime
   picryl ethers," McCullough, J. D., Jr.; Curtin, D. Y.; Paul, I. C. *J.
   Am. Chem. Soc.* **1972**, *94*, 874-882.
8. "Crystal structure of the syn- and
   anti-p-bromobenzophenone oxime O-picryl ethers," McCullough, J. D.,
   Jr.; Paul, I. C.; Curtin, D. Y. *J. Am. Chem. Soc.* **1972**,
   *94*, 883-889.
9. "X-ray crystal structures of the yellow and white forms of
   dimethyl 3,6-dichloro-2,5-dihydroxyterephthalate and a study of the
   conversion of the yellow form to the white form in the solid state,"
   Byrn, S. R.; Curtin, D. Y.; Paul, I. C. *J. Am. Chem. Soc.* **1972**,
   *94*, 890-898.
10. "Thermally induced organic reactions in the solid state," Paul,
    I. C.; Curtin, D. Y. *Acc. Chem. Res.* **1973**, *6*,
    217-225.
11. "Reactions of molecular crystals with gases. III. Relation
    of anisotropy to crystal structure in reactions of carboxylic acids and
    anhydrides with ammonia gas," Miller, R. S.; Curtin, D. Y.; Paul, I. C.
    *J. Am. Chem. Soc.* **1974**, *96*, 6340-6349.
12. "Structural studies on nitrosobenzene and 2-nitrosobenzoic
    acid. Crystal and molecular structures of cis-azobenzene dioxide and
    trans-2,2'-dicarboxyazobenzene dioxide," Dieterich, D. A.; Paul, I. C.;
    Curtin, D. Y. *J. Am. Chem. Soc.* **1974**, *96*,
    6372-6380.
13. "Reactions of molecular crystals with gases. II. X-ray
    structure of crystalline 4-chlorobenzoic acid and the anisotropy of its
    reaction with ammonia gas," Miller, R. S.; Paul, I. C.; Curtin, D. Y. *J.
    Am. Chem. Soc.* **1974**, *96*, 6334-6339.
14. "Reactions of molecular crystals with gases. I. Reactions
    of solid aromatic carboxylic acids and related compounds with ammonia
    and amines," Miller, R. S.; Curtin, D. Y.; Paul, I. C. *J. Am. Chem.
    Soc.* **1974**, *96*, 6329-6334.
15. "Use of solid-gas reactions to distinguish between left-
    and right-handed single crystals of an enantiomeric pair," Lin, C.-T.;
    Curtin, D. Y.; Paul, I. C. *J. Am. Chem. Soc.* **1974**, *96*,
    6199-6200.
16. "Anisotropic reaction with ammonia gas of a crystal of a
    carboxylic acid with linear hydrogen-bonded chains. Example of
    unitropic attack," Lin, C.-T.; Paul, I. C.; Curtin, D. Y. *J. Am.
    Chem. Soc.* **1974**, *96*, 3699-3701.
17. "Reactions of organic crystals with gases," Paul, I. C.; Curtin,
    D. Y. *Science* **1975**, *187*, 19-26.
18. "Correlation of the loss of solvent with the crystal
    structure of a 1:1 solvate. Indanetrione
    2-(N-p-tert-butylbenzoyl-N-phenylhydrazone) acetone solvate," Puckett,
    S. A.; Paul, I. C.; Curtin, D. Y. *J. Chem. Soc., Perkin Trans. 2*
    **1976**, 1873-1881.
19. "Correlation of chemical reactivity in the solid state with
    crystal structure," Paul, I. C.; Curtin, D. Y. *Jerusalem Symposia
    on Quantum Chemistry and Biochemistry* **1976**, *8*,
    307-327.
20. "A study of the crystalline environment of some
    dehydration reactions that take place in the solid state," Lewis, T.
    W.; Puckett, S. A.; Curtin, D. Y.; Paul, I. C. *Mol. Cryst. Liq.
    Cryst.* **1976**, *32*, 111-115.
21. "Dehydration of a carbinolhydrazine in the solid state.
    Correlation with crystal structure of the dehydration of
    2-hydroxy-2-(b-benzoyl-b-phenylhydrazyl)indan-1,3-dione," Puckett, S.
    A.; Paul, I. C.; Curtin, D. Y. *J. Am. Chem. Soc.* **1976**, *98*,
    787-792.
22. "Synthesis and interconversion by hydrogen exchange of isomeric
    quinhydrones," Desiraju, G. R.; Curtin, D. Y.; Paul, I. C. *J. Org.
    Chem.* **1977**, *42*, 4071-4075.
23. "The crystal and molecular structures of
    S-(+)-2,2-diphenylcyclopropanecarboxylic acid and of
    R-(+)-2,2-diphenyl-1-methylcyclopropanecarboxylic acid. A study of the
    environments of gas-solid reactions that exhibit anisotropic behavior,"
    Chiang, C. C.; Lin, C. T.; Wang, H. J.; Curtin, D. Y.; Paul, I. C. *J.
    Am. Chem. Soc.* **1977**, *99*, 6303-6308.
24. "Crystal growth by nonaqueous gel diffusion," Desiraju, G. R.;
    Curtin, D. Y.; Paul, I. C. *J. Am. Chem. Soc.* **1977**, *99*,
    6148.
25. "1,3-Benzoyl migration of
    2-benzoyl-2-phenylazoindan-1,3-dione. Molecular conformations and
    crystal structures of the rearrangement products indan-1,2,3-trione
    2-(N-benzoyl-N-phenylhydrazone) and indan-1,2,3-trione
    2-(N-bromobenzoyl-N-phenylhydrazone)," Puckett, S. A.; Greensley, M.
    K.; Paul, I. C.; Curtin, D. Y. *J. Chem. Soc., Perkin Trans. 2* **1977**,
    847-859.
26. "Conversion in the solid state of the yellow to the red
    form of 2-(4'-methoxyphenyl)-1,4-benzoquinone. X-ray crystal structures
    and anisotropy of the rearrangement," Desiraju, G. R.; Paul, I. C.;
    Curtin, D. Y. *J. Am. Chem. Soc.* **1977**, *99*,
    1594-1601.
27. "Color dimorphism of 14-hydroxymorphinone. X-ray analysis
    of two different crystalline modifications," Chiang, C. C.; DeCamp, W.
    H.; Curtin, D. Y.; Paul, I. C.; Shifrin, S.; Weiss, U. *J. Am. Chem.
    Soc.* **1978**, *100*, 6195-6201.
28. "[Crystal structure of] indan-1,2,3-trione
    2-(N-p-tert-butylbenzoyl-N-phenylhydrazone)," Booker, R. A.; Curtin, D.
    Y.; Paul, I. C. *Acta Crystallogr., Sect. B* **1978**, *B34*,
    2882-2885.
29. "1:1 Complex of indan-1,2,3-trione
    2-(N-benzoyl-N-phenylhydrazone) with indan-1,2,3-trione
    2-(N-phenylhydrazone)," McMillan, J. A.; Curtin, D. Y.; Paul, I. C. *Acta
    Crystallogr., Sect. B* **1978**, *B34*, 2878-2882.
30. "Picric acid: C6H3N3O7," Duesler, E. N.; Engelmann, J. H.;
    Curtin, D. Y.; Paul, I. C. *Cryst. Struct. Commun.* **1978**,
    *7*, 449-453.
31. "1-(a,a-Diphenylmethylene)-1,2-naphthoquinone," Duesler, E. N.;
    Lewis, T. W.; Curtin, D. Y.; Paul, I. C. *Acta Crystallogr., Sect. B*
    **1978**, *B34*, 985-988.
32. "Thermal, photochemical, and photonucleated thermal
    dehydration of p-hydroxytriarylmethanols in solids.
    (3,5-Dimethyl-4-hydroxyphenyl)diphenylmethanol and
    (3,5-dibromo-4-hydroxyphenyl)diphenylmethanol. X-ray crystal structures
    of (4-hydroxyphenyl)diphenylmethanol and its 3,5-dimethyl derivative,"
    Lewis, T. W.; Curtin, D. Y.; Paul, I. C. *J. Am. Chem. Soc.* **1979**,
    *101*, 5717-5725.
33. "Structural studies of 1:1 quinone-hydroquinone complexes,"
    Desiraju, G. R.; Curtin, D. Y.; Paul, I. C. *Mol. Cryst. Liq. Cryst.*
    **1979**, *52*, 563-566.
34. "Studies of thermal reactions in the solid state," Curtin,
    D. Y.; Paul, I. C.; Duesler, E. N.; Lewis, T. W.; Mann, B. J.; Shiau,
    W.-I. *Mol. Cryst. Liq. Cryst.* **1979**, *50*, 25-41.
35. "Solid-state resolution of binaphthyl: crystal and
    molecular structures of the chiral (A)1 form and racemic (B)1 form and
    the study of the rearrangement of single crystals. Requirements for
    development of hemihedral faces for enantiomer identification," Kress,
    R. B.; Duesler, E. N.; Etter, M. C.; Paul, I. C.; Curtin, D. Y. *J.
    Am. Chem. Soc.* **1980**, *102*, 7709-7714.
36. "Stoichiometric inclusion compounds of cyclotriveratrylene
    and cyclotricatechylene with small neutral molecules. X-ray crystal
    structure of cyclotricatechylene di-2-propanolate," Hyatt, J. A.;
    Duesler, E. N.; Curtin, D. Y.; Paul, I. C. *J. Org. Chem.* **1980**,
    *45*, 5074-5079.
37. "Investigation of crystalline naphthazarin B by carbon-13
    NMR spectroscopy using \"magic angle\" spinning techniques and by x-ray
    diffraction: evidence for a dynamic disordered structure," Shiau,
    W.-I.; Duesler, E. N.; Paul, I. C.; Curtin, D. Y.; Blann, W. G.; Fyfe,
    C. A. *J. Am. Chem. Soc.* **1980**, *102*, 4546-4548.
38. "Solid-state dehydration of
    (o-hydroxyaryl)diphenylmethanols. Crystal and molecular structures of
    [1-(2-hydroxyphenyl)]diphenylmethanol and of
    [1-(2-hydroxynaphthyl)]diphenylmethanol," Lewis, T. W.; Duesler, E. N.;
    Kress, R. B.; Curtin, D. Y.; Paul, I. C. *J. Am. Chem. Soc.* **1980**,
    *102*, 4659-4664.
39. "Experimental studies on the single crystal to single
    crystal transformation of 5-methyl-1-thia-5-azacyclooctane
    1-oxide(TACO) perchlorate. Evidence for an intermediate state,"
    Duesler, E. N.; Wiegers, K. E.; Curtin, D. Y.; Paul, I. C. *Mol.
    Cryst. Liq. Cryst.* **1980**, *59*, 289-298.
40. "Solid state phase transformation of a diacetylene by
    solvation. Crystal structure of a moderately reactive monomer form,"
    Patel, G. N.; Duesler, E. N.; Curtin, D. Y.; Paul, I. C. *J. Am.
    Chem. Soc.* **1980**, *102*, 461-466.
41. "2,6-Dimethyl-4-(a,a-diphenylmethylene)-1,4-benzoquinone (g
    form)," Duesler, E. N.; Lewis, T. W.; Curtin, D. Y.; Paul, I. C. *Acta
    Crystallogr., Sect. B* **1980**, *36*, 166-168.
42. "Molecular and crystal structures of the a and b forms of
    2,6-dimethyl-4-(a,a-diphenylmethylene)-1,4-benzoquinone(2,6-dimethylfuchsone):
    evidence for the thermal conversion of the racemic crystals (1a) to the
    chiral crystals (1b)," Lewis, T. W.; Paul, I. C.; Curtin, D. Y. *Acta
    Crystallogr., Sect. B* **1980**, *36*, 70-77.
43. "The heterogeneous thermal transformation of the orange
    diethyl N,N'-diphenyl-2,5-diaminoterephthalate-benzene complex to the
    red solvent-free compound. X-ray crystal structures of the two forms,"
    Mann, B. J.; Paul, I. C.; Curtin, D. Y. *J. Chem. Soc., Perkin
    Trans. 2* **1981**, 1583-1590.
44. "The heterogeneous thermal transformation of the yellow to
    the orange crystalline form of diethyl 2,5-diaminoterephthalate. X-ray
    crystal structures of the two forms," Mann, B. J.; Duesler, E. N.;
    Paul, I. C.; Curtin, D. Y. *J. Chem. Soc., Perkin Trans. 2* **1981**,
    1577-1582.
45. "Chemical consequences of the polar axis in organic solid-state
    chemistry," Curtin, D. Y.; Paul, I. C. *Chem. Rev.* **1981**,
    *81*, 525-541.
46. "Chemical consequences of a polar axis in a solid-gas
    reaction. Reaction of p-bromobenzoic anhydride crystals with ammonia
    gas. The absolute direction of a polar unitropic reaction and the
    relationship of absolute configuration with crystal morphology,"
    Duesler, E. N.; Kress, R. B.; Lin, C.-T.; Shiau, W.-I.; Paul, I. C.;
    Curtin, D. Y. *J. Am. Chem. Soc.* **1981**, *103*,
    875-879.
47. "Diethyl 2,5-bis[(o-aminophenyl)amino]terephthalate, C24H26N4O4,"
    Mann, B. J.; Wilson, R. B.; Curtin, D. Y.; Paul, I. C. *Cryst.
    Struct. Commun.* **1982**, *11*, 163-168.
48. "Deuterium nuclear quadrupole resonance spectra of
    nonlinear hydrogen bonds," Brown, T. L.; Butler, L. G.; Curtin, D. Y.;
    Hiyama, Y.; Paul, I. C.; Wilson, R. B. *J. Am. Chem. Soc.* **1982**,
    *104*, 1172-1177.
49. "Diacetylene monomers and polymers with chiral
    substituents: structure, solid-state polymerization, and properties,"
    Wilson, R. B.; Duesler, E. N.; Curtin, D. Y.; Paul, I. C.; Baughman, R.
    H.; Preziosi, A. F. *J. Am. Chem. Soc.* **1982**, *104*,
    509-516.
50. "Crystal structure and solid-state reactivity of
    4,4'-methylenediphenyl isocyanate (MDI)," Wilson, R. B.; Chen, Y. S.;
    Paul, I. C.; Curtin, D. Y. *J. Am. Chem. Soc.* **1983**, *105*,
    1672-1674.
51. "Solid state complexes of polymethylated quinones with
    polymethylated phenols. The crystal structure of the 2:1 complex of
    2,5-dimethyl-1,4-benzoquinone with 2,5-dimethyl-1,4-dihydroxybenzene,"
    Patil, A. O.; Wilson, S. R.; Curtin, D. Y.; Paul, I. C. *J. Chem.
    Soc., Perkin Trans. 2* **1984**, 1107-1110.
52. "Interconversion by hydrogen transfer of unsymmetrically
    substituted quinhydrones in the solid state. Crystal structure of the
    1:2 complex of 2,5-dimethylquinone with hydroquinone," Patil, A. O.;
    Curtin, D. Y.; Paul, I. C. *J. Am. Chem. Soc.* **1984**, *106*,
    4010-4015.
53. "Solid-state formation of quinhydrones from their
    components. Use of solid-solid reactions to prepare compounds not
    accessible from solution," Patil, A. O.; Curtin, D. Y.; Paul, I. C. *J.
    Am. Chem. Soc.* **1984**, *106*, 348-353.
54. "Polymorphism of naphthazarin and its relation to
    solid-state proton transfer. Neutron and x-ray diffraction studies on
    naphthazarin C," Herbstein, F. H.; Kapon, M.; Reisner, G. M.; Lehman,
    M. S.; Kress, R. B.; Wilson, R. B.; Shiau, W. I.; Duesler, E. N.; Paul,
    I. C.; Curtin, D. Y. *Proceedings of the Royal Society of London,
    Series A: Mathematical, Physical and Engineering Sciences* **1985**,
    *399*, 295-319.
55. "Effect of molecular symmetry and intermolecular
    halogen-halogen interactions on the crystal structures of
    halogen-substituted benzoic acids. X-ray crystal structure of
    m-iodobenzoic acid," Patil, A. A.; Curtin, D. Y.; Paul, I. C. *Isr.
    J. Chem.* **1985**, *25*, 320-326.
56. "CPMAS (cross-polarization magic angle spinning) carbon-13
    NMR spectra of quinones, hydroquinones, and their complexes. Use of CMR
    to follow a reaction in the solid state," Scheffer, J.; Wong, Y. F.;
    Patil, A. O.; Curtin, D. Y.; Paul, I. C. *J. Am. Chem. Soc.* **1985**,
    *107*, 4898-4904.
57. "Use of the pyroelectric effect to determine the absolute
    orientation of the polar axis in molecular crystals," Patil, A. A.;
    Curtin, D. Y.; Paul, I. C. *J. Am. Chem. Soc.* **1985**, *107*,
    726-727.
58. "Formation of crystalline complexes between polymethylated
    quinones and hydroquinones," Patil, A. O.; Curtin, D. Y.; Paul, I. C. *J.
    Chem. Soc., Perkin Trans. 2* **1986**, 1687-1692.
59. "Effect of methyl substitution on the crystal structures
    of complexes of quinones with hydroquinones. Crystal structures of the
    1:1 complex of 2,3,5,6-tetramethyl-1,4-benzoquinone with
    2,3,5,6-tetramethylbenzohydroquinone and of the 1:2 complex of
    2,3,5,6-tetramethylbenzoquinone with hydroquinone. Correlation of
    Fourier transform infrared spectra with structure," Pennington, W. T.;
    Patil, A. O.; Curtin, D. Y.; Paul, I. C. *J. Chem. Soc., Perkin
    Trans. 2* **1986**, 1693-1700.
60. "Recent studies on the formation and properties of
    quinhydrone complexes," Patil, A. O.; Pennington, W. T.; Desiraju, G.
    R.; Curtin, D. Y.; Paul, I. C. *Mol. Cryst. Liq. Cryst.* **1986**,
    *134*, 279-304.
61. "Solid-state reactivity of crystalline hydroquinones with
    quinone vapor; crystal structures of 2,5-dimethylhydroquinone and
    2,3,5,6-tetramethylhydroquinone," Pennington, W. T.; Patil, A. O.;
    Paul, I. C.; Curtin, D. Y. *J. Chem. Soc., Perkin Trans. 2* **1986**,
    557-563.
62. "Gas-solid reactions and polar crystals," Paul, I. C.; Curtin, D.
    Y. *Stud. Org. Chem. (Amsterdam)* **1987**, *32*,
    331-370.
63. "Reactions of crystalline (R)-(-)- and (S)-(+)-mandelic
    acid with amines. Crystal structure and dipole moment of (S)-mandelic
    acid. A method of determining absolute configuration of chiral
    crystals," Patil, A. O.; Pennington, W. T.; Paul, I. C.; Curtin, D. Y.;
    Dykstra, C. E. *J. Am. Chem. Soc.* **1987**, *109*,
    1529-1535.
64. "Crystal structures of D-(+)- and meso-hydrobenzoin.
    Absolute direction of the dipole moment of D- and L-hydrobenzoin in the
    crystal and correlation with crystal morphology, pyroelectric effect,
    and absolute configuration," Pennington, W. T.; Chakraborty, S.; Paul,
    I. C.; Curtin, D. Y. *J. Am. Chem. Soc.* **1988**, *110*,
    6498-6504.
65. "Structures and interconversion of polymorphs of
    2,3-dichloroquinizarin. Use of second harmonic generation to follow the
    change of a centrosymmetric to a polar structure," Hall, R. C.; Paul,
    I. C.; Curtin, D. Y. *J. Am. Chem. Soc.* **1988**, *110*,
    2848-2854.
66. "Crystal structure and direction of the polar axis of
    (-)-(1R)-pinonic acid b-oxime," Padmanabhan, K.; Paul, I. C.; Curtin,
    D. Y. *Acta Crystallogr., Sect. B* **1989**, *B45*,
    411-416.
67. "Carbon-13 NMR and x-ray structure determination of
    1-(arylazo)-2-naphthols. Intramolecular proton transfer between
    nitrogen and oxygen atoms in the solid state," Olivieri, A. C.; Wilson,
    R. B.; Paul, I. C.; Curtin, D. Y. *J. Am. Chem. Soc.* **1989**,
    *111*, 5525-5532.
68. "Carbon-13 CP-MAS study of the polymorphs of naphthazarin
    and of some methyl derivatives," Olivieri, A.; Paul, I. C.; Curtin, D.
    Y. *Magn. Reson. Chem.* **1990**, *28*, 119-123.
69. "Structure of N-iodosuccinimide," Padmanabhan, K.; Paul, I. C.;
    Curtin, D. Y. *Acta Crystallogr., Sect. C:* **1990**, *C46*,
    88-92.
70. "Configurational isomerism in crystalline forms of benzophenone
    anils," Matthews, J. H.; Paul, I. C.; Curtin, D. Y. *J. Chem. Soc.,
    Perkin Trans. 2* **1991**, 113-118.
71. "Structure of 3-benzoylbenzoic acid oxime," Paul, I. C.; Curtin,
    D. Y. *Acta Crystallogr., Sect. C:* **1992**, *C48*,
    2165-2167.
72. "Structure of p-acetylbenzoic acid oxime," Maurin, J. K.; Paul,
    I. C.; Curtin, D. Y. *Acta Crystallogr., Sect. C:* **1992**, *C48*,
    2163-2165.
73. "Structure of 3-benzoylbenzamide oxime," Maurin, J. K.; Paul, I.
    C.; Curtin, D. Y. *Acta Crystallogr., Sect. C:* **1992**, *C48*,
    1821-1823.
74. "Structure of (E)-4-benzoylbutyramide oxime," Maurin, J. K.;
    Paul, I. C.; Curtin, D. Y. *Acta Crystallogr., Sect. C:* **1992**,
    *C48*, 1819-1820.
75. "Hydrogen-bonded complex formation of oximes with
    carboxylic acids and with amides: (E)-acetophenone oxime-benzoic acid
    (1/1) and (E)-benzaldehyde oxime-benzamide (1/1)," Maurin, J. K.;
    Winnicka-Maurin, M.; Paul, I. C.; Curtin, D. Y. *Acta Crystallogr.,
    Sect. B* **1993**, *B49*, 90-96.
76. "Structures of 4-hydroxyimino-4-phenylbutanoic acid,
    C10H11NO3 (I), and 5-hydroxyimino-5-phenylpentanoic acid, C11H13NO3
    (II), at 223 K," Maurin, J. K.; Paul, I. C.; Curtin, D. Y. *Acta
    Crystallogr., Sect. C:* **1994**, *C50*, 78-81.

  
Last updated 03 March 2005  
