# Supplementary material for: Space groups and crystallographic symmetry: writing a multi-featured tutorial in a new style
Source: Acta Crystallogr E Crystallogr Commun. 2021 Jul 16;77(Pt 9):857–63. doi: 10.1107/S2056989021007039 (PMC8423017; doi:10.1107/S2056989021007039)
Supplement: Supplementary file 1 [file e-77-00857-sup2.zip › symandsg/Main/dharker.html]

David Harker, October 19, 1906 — February 27, 1991 | By Herbert A. Hauptman | Biographical Memoirs

|  |  |
| --- | --- |
| ***BIOGRAPHICAL MEMOIRS*** | National Academy of Sciences |

---


|  |  |
| --- | --- |
|  | David Harker  *October 19, 1906 — February 27, 1991*  By Herbert A. Hauptman |


THE FOCUS OF DAVE Harker's life, around which all his thoughts and actions revolved, was the science of
crystallography, which he dearly loved. To crystallography
he gave everything–his time, his energy, his total devotion.
So complete was his dedication to this science and so
fundamental and many faceted were his contributions that
he influenced forever the course of its development. To
this day, the Harker section and the Harker construction
play essential roles in the determination of the structures
of very large molecules. The Harker-Kasper inequalities
provided the inspiration for a new branch of X-ray
crystallography, the so-called direct methods of phase determination.

|  |
| --- |
| **PERSONAL HISTORY** |

Dave was born on October 19, 1906, into a scientific
and medical family. He grew up on the side of Mount
Tamalpais in Mill Valley near San Francisco within view of the bay
end of the Golden Gate Bridge. His father, George Asa
Harker, who died when Dave was five years old, was a medical
doctor from the University of California at Berkeley.
Dave's father introduced the concepts of shape, symmetry,
and structure into Dave's life. His earliest memories of his
father
are of him sitting on the front porch and making
plaster molds of his patients' feet, carefully hammering
copper into precise forms of arch support.

His mother, Harriette Butler Harker, graduated from
Vassar in 1898 and received her M.D. from the University of
California at Berkeley. She boasted that she was the first
woman in New Brunswick, New Jersey, to go to college, wear
trousers, ride a bike, and smoke a cigar. Dave's mother
personally took charge of his and his brother's education until
the fourth grade. His mother, together with faculty
members from Berkeley, taught classes at his high school in
exchange for free tuition.

In 1928 Dave graduated with honors in chemistry
from the University of California at Berkeley. His
undergraduate years had brought him into contact with distinguished
faculty that included Joel H. Hildebrand and Wendell
M. Latimer.

In 1930 Dave married Katherine De Savich, who, as
the daughter of the imperial prosecutor under the tzar,
fled Russia in 1917. Katherine later aided Dave in
translating scientific books into English. They also spent ten years
working on the translation of a Soviet physics journal for
crystallography, until her death in 1973. They had two
daughters, Tatiana Harker Yates and Liudmilla Harker.

Following the death of his first wife, Dave married
Deborah Maxwell in 1974. She died in 1997, six years after Dave's death.

|  |
| --- |
| **PROFESSIONAL HISTORY**  **ATMOSPHERIC NITROGEN CORP. (1930-33)** |

After graduation from Berkeley, Dave continued on as
a graduate student, but in 1930 he left to take a job as
laboratory technician at the research laboratory of the
Atmospheric Nitrogen Corp. in Solvay, N.Y. (near Syracuse). There
he weighed samples, made mixtures, and occasionally read
scientific
journals. In one of these he read a paper on
the crystal structure of sodium nitrate and its change as
the nitrate groups rotate at elevated temperatures. This
beautiful result so impressed him that he resolved to study
crystal structures in greater depth at some future time.

|  |
| --- |
| **CALTECH: THE HARKER SECTION (1933-36)** |

In 1933 (the depth of the Depression) Dave lost his
job. He returned to California with his wife and child,
borrowed some money from an old friend of his parents, and
entered the graduate school of the California Institute of
Technology. There, under the supervision of Linus Pauling, he
began to work on the determination of crystal structures
using the technique of X-ray diffraction. After some
preliminary studies of three or four simple structures, he
undertook the solution of his dissertation problem: to determine
the structures of the ruby silvers, proustite
(Ag3AsS3) and pyrargyrite
(Ag3SbS3), which are isomorphous (i.e., they
have the same structure).

Although only six parameters were needed to
describe these structures, the methods available at that time
(essentially clever trial and error) were totally inadequate.
Then, at one of the weekly seminars of Pauling's students, A.
L. Patterson's famous 1934 paper on the Patterson
function was presented. This function relates the experimentally
observable X-ray diffraction intensities with the totality of
interatomic vectors in the crystal. Owing to the large
number of interatomic vectors, interpreting the Patterson
function was, and still is, no easy task. A few nights after the
seminar, in Dave's words, he "awoke in the dark, sat up in bed,
and yelled, 'It's going to work.'" What he had seen was that
the relationships between symmetrically related atoms
would produce peaks in the Patterson function on certain
planes or along certain lines determined by the known
crystallographic
symmetries. These "Harker" peaks often lead
directly to the atomic position vectors and the crystal
structure, particularly in those cases when the Patterson
function itself is not readily interpretable. Thus was born
the famous Harker section, which effectively made the
Patterson function useful. In this way Dave quickly deduced the
structures of proustite and pyrargyrite and earned his Ph.D.
in 1936. The Harker section has withstood the test of
time and even today is indispensable for the determination
of macromolecular structures, particularly in those cases
where the structure contains a small number of heavy atoms,
when Patterson techniques are useful.

|  |
| --- |
| **JOHNS HOPKINS YEARS: THE DONNAY-HARKER LAW (1936-41)** |

Having become a physical chemist in 1936, Dave took
an academic job in chemistry at the Johns Hopkins
University, where he taught freshman chemistry, graduate courses
in crystal structure, crystal chemistry, and quantum
mechanics. He also inherited some X-ray diffraction equipment
left over by his predecessor M. L. Huggins.

Since in those days research money was in very
short supply, he and his students made their own equipment
from secondhand materials. In this way they set up a
continuously pumped X-ray tube and with its aid worked on
several crystal structure problems. Of these, the structures of
acetamide and hydrazinium difluoride were published.

During Dave's tenure at Johns Hopkins, Dorothy
Wrinch came to visit the university for about a year. She and
Irving Langmuir, who visited Johns Hopkins occasionally,
engaged in extended discussions concerning her theories of
protein structures. Dave was drawn into their conversations and
soon became interested in the problem of protein structure
determination. In addition, during this period, W. T.
Ashbury of Leeds gave a colloquium on the structures of
fibrous
proteins.
Dave, in his words, "became infected with the
protein structure virus, but for many years it lay dormant."

During those years, Dave met Professor J. D. H.
Donnay of Johns Hopkins and George Tunnell of the
Geophysical Laboratory in Washington, D.C. From these
prominent mineralogists Dave learned classical crystallography,
some mineralogy, and the significance and measurement of
crystal faces. It was Donnay's goal to correlate the internal
structure and external face development of crystals. The
earlier attempt to do this by Bravais resulted only in a rather
poor approximation. Donnay and Harker discovered that the
order of decreasing prominence of the faces of a crystal
was the same as the order of decreasing interplanar lattice
spacings, including the halvings, thirdings, and quarterings
due to the space group symmetries. This correlation, while
still not perfect, was an improvement over Bravais's earlier
attempt. It is known in mineralogical circles as the
Donnay-Harker law.

|  |
| --- |
| **GENERAL ELECTRIC: THE HARKER-KASPER INEQUALITIES (1941-50)** |

In 1941 Dave received an offer from W. D. Coolidge
to work in the famous research laboratory of the General
Electric Company and after some hesitation he accepted it. He
became a member of the metallurgy division at General
Electric and proceeded to learn properties of metals using
X-ray diffraction and other crystallographic methods.
Owing to the liberal policy of the General Electric research
laboratory in those days, Dave was not compelled to work
exclusively on metals, although he did publish several papers
on solid state reactions characteristic of them, including a
paper on grain shape and grain growth, another on
order-disorder reaction, and several others.

Although Dave is known primarily for his
contributions to X-ray crystallography, his metallurgical papers had a
considerable
impact on the physical metallurgical
community. One of these, in particular, was primarily concerned
with the microstructural subtleties associated with the
ordering reaction in the alloy AuCu in which there is a change
in unit cell from cubic to tetragonal. His theoretical
analysis of the complex microstructures, which are to be
expected as a means for the material to avoid long-range
internal stresses, was far ahead of its time and had
considerable influence on the research concerned with ordering
reactions in alloys.

During his years at General Electric, Dave also
developed an X-ray method for finding the orientation of quartz
fragments, so that oscillator plates could be cut from them.
In addition, he did several pieces of crystallographic work
for other divisions of the laboratory. He also started work
on the design of X-ray diffraction equipment with which
the diffracted intensity would be measured with a
Geiger-M�ller or other particle counter.

It was during Dave's tenure at General Electric that
he and his collaborator John S. Kasper produced their
paper on the inequalities among the crystal structure factors,
the famous Harker-Kasper inequalities. Because these
inequalities constitute the first contribution to the direct
methods of phase determination, which now (1997) has a
fifty-year history and which continues to be a subject of intense
interest, activity, and importance, it is appropriate to
describe in some detail the circumstances surrounding their
discovery. We are fortunate to have first-hand accounts by
the authors. First, Dave's account:

> One problem in particular fascinated us–the determination of the
> crystal structure of decaborane,
> B10H14. This turned out to be surprisingly
> difficult. It was borne in upon Dr. John S. Kasper and me that a
> structure which could not easily be guessed at approximately from known
> stereochemical principles, could not be solved by the traditional trial and
> error
> methods.
> Some twenty structures for the
> B10H14 molecule had been published, but none could be made to fit the X-ray diffraction data from
> the crystals.

One day John Kasper was sitting at his desk staring gloomily at a
lot of algebra he had been writing down. I looked over his shoulder and
said something like, "What on earth is that?" and he replied "Schwartz's
Inequality for a structure factor, but it doesn't seem to help." He then
kept on writing, while I looked on. I said, "Oh, well, let's expand those
squares of cosines into functions of double angles." So we did. Then it hit us
both, I think, at the same time. "Say! We can get the signs of some
structure factors from this!" Then we went madly to work, and in a couple of
weeks we had enough algebraic apparatus assembled "unitary" structure
factors, sum and difference inequalities, etc.–to be useful. Kasper applied
this schema to the decaborane data and came out with a preliminary
model which explained the diffracted intensities from one zone, and, after
another couple of months, the complete structure emerged. Thus was
born the subject of "sign determination" from intensities. This was in 1947.

At my request John Kasper sent me his account, with
a postscript by his wife Charlys:

> Here is my version of the origin of the sign-determining inequalities.
> First, I would like to give you some background information that may be
> of interest to you.

At the 1946 meeting of ASXRED (American Society for X-ray
and Electron Diffraction) at Lake George, N.Y., a method of attacking the
phase problem was presented by A. Booth, namely, the method of steepest
descent. While this did not turn out to be a viable method,
considerable discussion of the phase problem ensued. Nothing useful resulted,
however, and there was a consensus that nothing could be done about
obtaining phases and that it was a waste of time to think about it. Among the
minority were Dave Harker, Buerger, and Fankuchen, although no
convincing evidence could be given to justify the optimistic viewpoint. For Dave
and myself the phase problem was on our minds although we were quite
busy with other problems at G. E.

I became intrigued with the fact that the straightforward squaring
of a real structure factor, Fhkl (with cosine terms) contained, in part, the
sum of modified cosine squared terms. These latter could be rewritten, by
virtue of the relation 2 cos2A=1+cos 2A as components of
F2h,2k,2l. A relation then exists between
F2hkl and F2h,2k,2l, but also with the summation of
cross
terms.
I did not know what to do with the cross terms and so I put
the thing aside. Some days later (in 1947) it occurred to me that
Schwartz's inequality would deal only with the desirable summation of
cosine2 terms. Accordingly, one morning at work I wrote down the relationship
between F2hkl and
F2h,2k,2l resulting from the application of Schwartz's inequality.
No sooner had I written this down, when Dave walked in the office and
looked over my shoulder. "What is that?" Dave asked. "That is the result of
applying Schwartz's inequality to a structure factor," I replied. After
satisfying himself that what I had written was alright, Dave became quite excited
and remarked: "You can determine signs with that." "That's right," I replied.

I was unhappy, however, that the treatment so far was only for
the case of one kind of atom. Dave said that could be fixed, and in short
order he proposed using the unitary atomic structure factor, . This enabled treatment of more general situations.

For the next few weeks Dave was immersed in the applications
to various symmetries and space groups, and other ramifications, such as
sum and difference formulas. He also produced an elegant write up of
the work. I concentrated on its application to the Decaborane problem
which was uppermost in our minds.

I realize that my version is not exactly the same as one that Dave
has given, but I stand by it. We were in communication in 1989, with the
goal of achieving a version that was mutually agreeable, I regret deeply
that Dave's illness prevented the completion of that project.

From what you say I wonder if you have the autobiography which
was written in 1961, and which Dave sent to me in 1989. It is very
interesting reading to anyone who knew Dave. I have little to add to it. I would
mention what a good and influential teacher he was. I first knew Dave as
a teacher of freshman chemistry at Johns Hopkins. He revolutionized
the course with emphasis on basic principles. His approach was adopted
by students who subsequently taught chemistry. He only mentions his work
in metallurgy, but his contributions were fundamental in the areas of
grain growth and recrystallization and in order-disorder phenomena. I
would like to add that the single crystal orienter he developed was the first
such device for use with a counter.

I hope this is useful to you. I am not able to do many things
because I now am legally blind. That is why I am unable to attend the tribute
to
Dave.

I would appreciate a copy of the Biography when it is done.

Sincerely,

John S. Kasper

JSK:clk

P.S. I am typing this for John. I was working closely with both John
and Dave on the decaborane problem at the time and clearly recall the
sequence of events as John has described. I was also working in the
office while John was busy working with the relationship of Schwartz's
inequality and the structure factors to possibly help determine signs when Dave
arrived in the office and became very excited at the possibilities of its use.
It was an event one doesn't forget.

Charlys Lucht Kasper

It is appropriate to point out here the mathematical
basis of the Harker-Kasper inequalities since this is not
mentioned explicitly in their paper. This is simply the
non-negativity property of the electron density function, a fact
implicitly assumed in their analysis.

After a good deal of prodding on Dave's part, the
X-ray department of General Electric was finally persuaded
to build its first counter diffractometer for powder
patterns, although not before the North American Philips Co.
had already put a similar device on the market. Next, Dave
set about adapting it to single crystal work. By 1949 he
had built several models and had used them successfully,
mostly on metallurgical problems.

During his time at General Electric, Dave served as
president of the Society for X-ray and Electron Diffraction
(1946). He also headed the American delegation to the
London conference where the formation of the International
Union
of
Crystallography was proposed and later was
established, along with its adhering body in the United States, the
U.S. National Committee for Crystallography.

|  |
| --- |
| **BROOKLYN POLY YEARS: THE HARKER CONSTRUCTION AND RIBONUCLEASE (1950-59)** |

The next phase in Dave's career was triggered in the
fall of 1949 by Irving Langmuir, who asked him what he
would do with a million dollars. To this seemingly rhetorical
question Dave's offhand response was that he would take
ten years off and determine the structure of a protein. To
Dave's great surprise, within two weeks Langmuir came to his
office and announced that he could raise the money.
Dave suddenly realized that determining the structure of a
protein was what he had wanted to do for some time.
After months of interminable negotiations, the decision
finally was made to establish the Protein Structure Project at
the Polytechnic Institute of Brooklyn in July 1950. There
Dave and his team built a good single-crystal X-ray
diffractometer with counter detection of the diffracted beams. The
central device in this unit was a sort of theodolite arrangement
for orienting the crystal in any possible way. They called
this device a "Eulerian cradle," because the angular motions
it could give the crystal were Euler's angles. This
instrument was eminently successful, and led to the commercial
goniostat, which soon became increasingly popular. Much of the
success of this instrument was due to its careful design,
for which Thomas C. Furnas, Jr., was primarily responsible.

They chose ribonuclease as the protein on which to
work, because it could be had relatively pure at a reasonable
price, could be readily crystallized, and it had a quite small
molecular weight. Murray King crystallized this substance
in fourteen different modifications eventually. He also
invented the method of attaching heavy atoms to specific sites in
the
protein
crystals by "dyeing" the crystals with specially
synthesized dyes, the molecules of which contained heavy
atoms. Dave worked out the scheme of phase
determination for protein structure factors, which involved using the
intensities from three isomorphous crystalsone undyed,
the other two dyed with heavy atoms in different
arrangements, a scheme used by macromolecular crystallographers to
this day. It turned out that Professor Bijvoet of Utrecht
had found the same principle a few years earlier, but he had
not emphasized it in his papers. This scheme, since called
the method of multiple isomorphous replacement, led to
the first successful structure determinations of crystalline
proteinsthose of myoglobin by Sir John C. Kendrew and
of hemoglobin by Max F. Perutz, both of Cambridge
University. For this work they received the Nobel Prize in
chemistry for 1962.

|  |
| --- |
| **ROSWELL PARK CANCER INSTITUTE: THE STRUCTURE OF RIBONUCLEASE (1959-76)** |

From 1950 to 1959 Dave and his team worked at
the Brooklyn Polytechnic Institute on the crystal structure
problem presented by the protein ribonuclease. In 1959
Dave moved the whole project to the Roswell Park Cancer
Institute (then known as the Roswell Park Memorial
Institute), where he accepted the position of head of the
biophysics department. Due to the efforts of visiting
crystallographers, a number of critical problems were solved during the
Roswell Park years. M. V. King solved the problem of dyeing
the protein molecules in crystals and he prepared
ribonuclease in fourteen different crystal forms. F. H. C. Crick
discovered the strong temperature dependence of the
diffracted X rays from the protein crystals mounted in sealed
capillaries and showed how to control it. V. Luzzati showed
how the intensity statistics were related to the structure of
the
protein
crystals and why the standard statistical
methods could not be applied in these cases. A. Tulinsky worked
out the exact structure of beryllium basic acetate and made
it into a useful intensity standard. G. Kartha developed
new ways of using the diffraction data from
non-centrosymmetric crystals. A. de Vries showed how anomalous
dispersion effects could help in determining the structures of
crystalline proteins. J. Bello discovered new ways of labeling
ribonuclease crystals with heavy atoms. T. C. Furnas, Jr.,
built their counter diffractometer, aided by that artist in
instrument construction W. G. Weber.

The stage was set to begin to collect X-ray
crystallographic data from which the structure of ribonuclease could
be determined. This goal was finally reached in 1967 with
the determination of the crystal and molecular structure of
ribonuclease, the first protein structure to be determined
in the United States.

In the years following the determination of the
structure of ribonuclease, Dave was honored locally by three
major awards. In 1967 he received the Sigma Xi Award for
meritorious service to science from the State University of
New York at Buffalo. The *Buffalo Evening
News* awarded Dave its Outstanding Citizen Award in 1968, and the western
New York section of the American Chemical Society
awarded him the Schoellkopf Prize in 1969.

|  |
| --- |
| **THE FINAL YEARS: HAUPTMAN-WOODWARD MEDICAL RESEARCH INSTITUTE (1977-91)** |

In 1976 Dave retired from the Roswell Park Cancer
Institute, but he continued his crystallographic studies as a
research scientist emeritus at the Hauptman-Woodward
Institute (then known as the Medical Foundation of
Buffalo). He became interested in the more mathematical aspects
of crystallography, in particular the theory of colored
space
groups
and a description of several classes of infinite
polyhedra.

During the next fifteen years, Dave was honored
with several appointments and awards. The year 1977
marked Dave's election to the National Academy of Sciences
and the American Academy of Arts and Sciences. Two years
later, in 1979, he was nominated for a Nobel Prize. In 1980
the American Crystallographic Association honored him
with the prestigious Fankuchen Award in recognition of his
services to crystallography, in particular his research
accomplishments and his role as a teacher of crystallography.
In 1981 the State University of New York at Buffalo
awarded Dave an honorary degree of doctor of science in
recognition of his long and outstanding career in science, the
first such award by this university. In 1984 Dave received
the Gregory Aminoff Medal in Gold from the Royal
Swedish Academy of Sciences in recognition of his fundamental
contributions to the development of methods in X-ray
crystallography and for his determination of the molecular
structures of biologically important substances. On
Dave's eighty-second birthday, in 1988, the David Harker
Endowment Fund was established by an anonymous donor at
the Hauptman-Woodward Institute in Buffalo. The fund is
intended to support research and lectures in
crystallography. In 1989 Dave prepared a paper announcing his
discovery of four new types of polyhedra, which he named the
"tortuously corrugated two dimensionally infinite polyhedra."
This paper was published shortly before his death in the
January 1991 issue of *Proceedings of the National Academy of
Sciences*.

In conclusion, Dave was a warm and friendly man,
courteous and unpretentious, concerned to be helpful,
particularly to younger colleagues; and his teaching was
unsurpassed. He was reserved, almost shy, an
old-fashioned gentleman with old-fashioned values. He was one of
the
greatest
crystallographers of this century, but he was
never patronizing to others, young or old. He was kind and
gentle and, at the same time, a man of uncompromising
honesty and integrity. He was a tireless seeker of the truth,
wherever he could find it, and in this quest he succeeded as
few others have. On February 27, 1991, Dave died of
complications due to heart disease and pneumonia.

I WISH TO MAKE grateful acknowledgement to Ms. Tava Shanchuk
for her help in writing an initial draft of this biography; this was
of considerable assistance to me in the preparation of the final
manuscript.

|  |
| --- |
| **SELECTED BIBLIOGRAPHY** |

**1936**
:   The application of the three-dimensional Patterson method
    and the crystal structures of proustite,
    Ag3AsS3, and pyrargyrite,
    Ag3SbS3. *J. Chem.
    Phys.* 4:381-90.

**1937**
:   With J. D. H. Donnay. A new law of crystal morphology
    extending the law of Bravais. *Am. Mineral.* 22:446-47.

**1938**
:   With A. Kossiakoff. The calculation of the ionization constants
    of inorganic oxygen acids from their structures.
    *J. Am. Chem. Soc.* 60:2047.

**1940**
:   With J. D. H. Donnay. Nouvelles tables d'extinctions pour les
    230 groupes de recouvrements crystallographiques.
    *Nat. Can. (Que.)* 67:33-69

**1945**
:   With E. R. Parker. Grain shape and grain growth.
    *Tran. Am. Soc. Met.* 34:156.

**1948**
:   With J. S. Kasper. Phases of Fourier coefficients directly from
    crystal diffraction data. *Acta Crystallogr.*1:70-75.

**1950**
:   With J. S. Kasper and C. M. Lucht. The crystal structure of
    decaborane, B10H14. *Acta Crystallogr.*3:436-55.

**1951**
:   With D. MacLachlan, Jr. Finding the signs of the F's from the
    shifted Patterson product. *Proc. Natl. Acad. Sci. U. S.
    A.*
    37:846-49.

**1953**
:   The meaning of the average |F|2 for large values of the
    interplanar spacing. *Acta Crystallogr.* 6:731-36.

**1955**
:   With T. C. Furnas, Jr. Apparatus for measuring complete
    single-crystal X-ray diffraction data by means of a Geiger
    counter diffractometer. *Rev. Sci. Instrum.* 26:449-53.

**1956**
:   X-ray diffraction applied to crystalline proteins.
    *Adv. Biol. Med. Phys.* 4.
:   The determination of the phases of the structure factors of
    non-centrosymmetric crystals by the method of double
    isomorphous replacement. *Acta Crystallogr.* 9:1-9.
:   With others. Crystalline forms of bovine pancreatic
    ribonuclease: techniques of preparation, unit cells, and space groups.
    *Acta Crystallogr.* 9:460-65.

**1961**
:   With J. Bello and E. DeJarnette. X-ray investigation of
    reduced-reoxidized ribonuclease. *J. Biol.
    Chem.* 236:1358.

**1962**
:   With others. Crystalline forms of bovine pancreatic
    ribonuclease. Some new modifications. *Acta Crystallogr.*15:144-47.

**1967**
:   With G. Kartha and J. Bello. Tertiary structure of bovine
    pancreatic ribonuclease at 2� resolution.
    *Nature* 213:862-65.

**1972**
:   Meylin membrane structure as revealed by X-ray diffraction.
    *Biophys. J.* 12:1285-95.

**1976**
:   A table of the colored crystallographic and icosahedral point
    groups, including their chirality and diamorphism.
    *Acta Crystallogr. Sect. A*
    32:133-39.

**1978**
:   Colored lattices. *Proc. Natl. Acad. Sci. U. S. A.*75:5264-67.
:   The effect of rotational symmetry on colored lattices.
    *Proc. Natl. Acad. Sci. U. S. A.*  75:5751-54.

**1981**
:   The three-colored three-dimensional space-groups.
    *Acta Crystallogr. Sect. A* 37:286-92.

**1991**
:   Two-dimensionally infinite polyhedra with vertices related by
    symmetry operations. *Proc. Natl. Acad. Sci. U. S.
    A.* 88:585-87.


---

|  |  |
| --- | --- |
| *Biographical Memoirs* | National Academy of Sciences |
