# Supplementary material for: Space groups and crystallographic symmetry: writing a multi-featured tutorial in a new style
Source: Acta Crystallogr E Crystallogr Commun. 2021 Jul 16;77(Pt 9):857–63. doi: 10.1107/S2056989021007039 (PMC8423017; doi:10.1107/S2056989021007039)
Supplement: Supplementary file 1 [file e-77-00857-sup2.zip › symandsg/Main/dharker_files/fhat.htm]

The National Academies Press - 404 Error: File Not Found


|  |  |  |  |  |  |  |  |  |  |  |  |  |  |  |  |  |  |  |  |
| --- | --- | --- | --- | --- | --- | --- | --- | --- | --- | --- | --- | --- | --- | --- | --- | --- | --- | --- | --- |
| |  |  |  |  |  |  |  |  |  |  |  |  |  |  |  |  |  |  |  | | --- | --- | --- | --- | --- | --- | --- | --- | --- | --- | --- | --- | --- | --- | --- | --- | --- | --- | --- | | |  |  | | --- | --- | |  |  | | Read more than 3,600 books online FREE! More than 1000 PDFs now available for sale | | |  |  |  |  |  |  |  | | --- | --- | --- | --- | --- | --- | --- | | HOME | ABOUT NAP | CONTACT NAP | HELP | NEW RELEASES | ORDERING INFO | Items in cart [0] | | | | |  |  |  |  | | --- | --- | --- | --- | | TRY OUR SPECIAL **DISCOVERY ENGINE**: | |  |  |   Questions? Call 888-624-8373 | | | |
| |  |  |  |  |  |  |  |  |  |  |  |  |  |  |  |  |  |  |  |  |  |  |  |  |  |  |  |  |  |  |  |  |  | | --- | --- | --- | --- | --- | --- | --- | --- | --- | --- | --- | --- | --- | --- | --- | --- | --- | --- | --- | --- | --- | --- | --- | --- | --- | --- | --- | --- | --- | --- | --- | --- | --- | | |  | | --- | | Categories | | Agriculture | | Behavioral Science | | Biology | | Computer Science | | Chemistry | | Earth Science | | Education | | Energy | | Engineering | | Environmental Issues | | Food & Nutrition | | General Interest | | Industry & Economics | | International | | Marine/Maritime | | Math & Statistics | | Materials | | Medicine/Public Health | | Natural Resources | | Physical Sciences | | Public Policy | | Research Issues | | Space Science | | Science & Ethics | | Transportation | | Urban Development | | |  | | --- | |  | | We apologize for the inconvenience, but we are unable to locate the content you requested. | | **Suggestions:**   » Check the spelling in the URL. Extra spaces or characters will cause errors.    » Try our search engine (above) to find similar information. Use a title or key     phrase related to the desired publication.    » Use the categories on the left to browse recent reports.    » Visit the National Academies Press homepage.   » Contact customer service to ask for assistance.  If you would like to purchase a book, please call our Customer Service Department toll-free at (888) 624-8373. We are open from 8:30 a.m. to 5:00 p.m. (eastern standard time), Monday through Friday, except holidays. We offer toll-free service from within the United States, including Alaska and Hawaii, and from Canada. | |  | |

  
  


|  |  |  |
| --- | --- | --- |
| [ Top of Page ] [ Home ] [ Contact Us ] [ Help ]  [ The National Academies Home ] | | |
| Copyright © 2006. National Academy of Sciences. All rights reserved. 500 Fifth St. N.W., Washington, D.C. 20001.  Terms of Use and Privacy Statement | | |
