# Supplementary material for: Space groups and crystallographic symmetry: writing a multi-featured tutorial in a new style
Source: Acta Crystallogr E Crystallogr Commun. 2021 Jul 16;77(Pt 9):857–63. doi: 10.1107/S2056989021007039 (PMC8423017; doi:10.1107/S2056989021007039)
Supplement: Supplementary file 1 [file e-77-00857-sup2.zip › symandsg/Main/ecamembership.htm]

European Crystallographic Association - Membership

European

Crystallographic

Association

# Membership

|  |
| --- |
| General lnformation  Membership Information  National (Countries)  Individual (Persons)  Affiliate (Corporate)  Benefits for individual members  Registration  Election of IM Councillors |

  
  

|  |
| --- |
|  |

# ECA membership

---

The ECA acknowledges three types of membership:

- National Membership (Countries)
- Affiliate [Corporate] Membership (Companies)
- Individual Membership (Persons)

Note that Europe in the ECA statutes is more then just Europe in the geographical meaning! See the
List of National Members

The ECA whishes to promote Individual Membership relative to the present situation where
National Membership dominates

Why IM-ship should be promoted?

This is an enthusiastic call for you to become an Individual Member of the ECA !

Why should you ??

What are your personal benefits?

If you are an Individual Member: thank you for joining your  colleagues
in making crystallography in Europe, neighbouring countries, countries
around the Mediterranian, and South Africa a powerful living
science.

If you are NOT YET an Individual Member, you can register now. Please, do so.

**Do not forget to register in the World Directory of Crystallographers - it's free!**
