# Supplementary material for: Space groups and crystallographic symmetry: writing a multi-featured tutorial in a new style
Source: Acta Crystallogr E Crystallogr Commun. 2021 Jul 16;77(Pt 9):857–63. doi: 10.1107/S2056989021007039 (PMC8423017; doi:10.1107/S2056989021007039)
Supplement: Supplementary file 1 [file e-77-00857-sup2.zip › symandsg/Main/elem.htm]

Elementary Crystallography - The MIT Press

|  |  |
| --- | --- |
|  | HOME | YOUR PROFILE | TO ORDER | CONTACT US | FAQ |
|  | |
|  | |  |  |  | | --- | --- | --- | |  |  |  | |

|  |  |  |  |  |  |  |  |  |  |  |  |  |  |  |  |  |  |  |  |  |  |
| --- | --- | --- | --- | --- | --- | --- | --- | --- | --- | --- | --- | --- | --- | --- | --- | --- | --- | --- | --- | --- | --- |
| |  | | --- | | May 1978 (PAPER)   ISBN-10:  0-262-52048-6 ISBN-13:  978-0-262-52048-5 **Out Of Print** | |  | Elementary Crystallography  **An Introduction to the Fundamental Geometric Features of Crystals**  Martin Buerger       Of Related Interest:   |  | | --- | |  |  |  |  | | --- | --- | | The Closed World Paul N. Edwards Paper / August 1997 | Information Technologies and International Development Quarterly (Fall, Winter, Spring, Summer) |   **See Other Titles In:**   |  | | --- | |  |  |  |  |  |  |  | | --- | --- | --- | --- | --- | | |  |  | | --- | --- | |  | | | > | Science, Technology & Society | | | |  | | --- | |  | | Title Author Keyword ISBN/ISSN | | Advanced Search |     |  |  |  |  |  | | --- | --- | --- | --- | --- | | |  |  | | --- | --- | |  | Join an E-mail Alert List | |  | E-Mail This Link | | |

|  |  |  |  |  |  |
| --- | --- | --- | --- | --- | --- |
|  | | | | | |
|  | | | | | |
| |  |  | | --- | --- | | ABOUT THE PRESS | RSS FEEDS | TERMS OF USE | PRIVACY POLICY | COPYRIGHT � 2006 | | | | | | |
