# Supplementary figures and images for: Space groups and crystallographic symmetry: writing a multi-featured tutorial in a new style (part 2 of 2)
Source: Acta Crystallogr E Crystallogr Commun. 2021 Jul 16;77(Pt 9):857–63. doi: 10.1107/S2056989021007039 (PMC8423017; doi:10.1107/S2056989021007039)

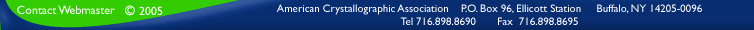

Supplement: Supplementary file 1 [file e-77-00857-sup2.zip › symandsg/Main/Buerger_files/suppage_footer.gif]

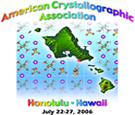

Supplement: Supplementary file 1 [file e-77-00857-sup2.zip › symandsg/Main/Buerger_files/testWEB.jpg]

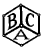

Supplement: Supplementary file 1 [file e-77-00857-sup2.zip › symandsg/Main/CAB_files/bca_min.gif]

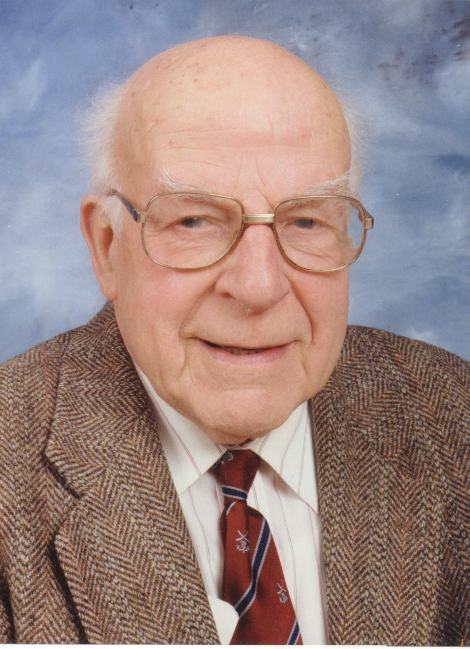

Supplement: Supplementary file 1 [file e-77-00857-sup2.zip › symandsg/Main/CAB_files/CAB.jpg]

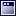

Supplement: Supplementary file 1 [file e-77-00857-sup2.zip › symandsg/Main/cotton_files/calendar2.gif]

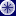

Supplement: Supplementary file 1 [file e-77-00857-sup2.zip › symandsg/Main/cotton_files/compass.gif]

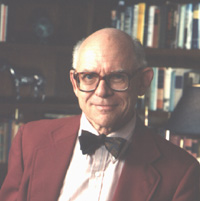

Supplement: Supplementary file 1 [file e-77-00857-sup2.zip › symandsg/Main/cotton_files/cotton.jpg]

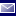

Supplement: Supplementary file 1 [file e-77-00857-sup2.zip › symandsg/Main/cotton_files/envelope.gif]

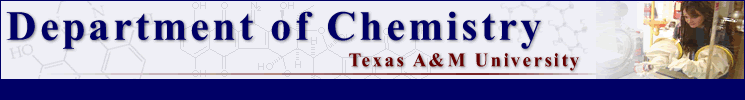

Supplement: Supplementary file 1 [file e-77-00857-sup2.zip › symandsg/Main/cotton_files/header.gif]

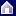

Supplement: Supplementary file 1 [file e-77-00857-sup2.zip › symandsg/Main/cotton_files/home.gif]

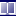

Supplement: Supplementary file 1 [file e-77-00857-sup2.zip › symandsg/Main/cotton_files/phonebook.gif]

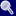

Supplement: Supplementary file 1 [file e-77-00857-sup2.zip › symandsg/Main/cotton_files/search.gif]

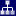

Supplement: Supplementary file 1 [file e-77-00857-sup2.zip › symandsg/Main/cotton_files/sitemap.gif]

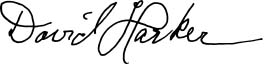

Supplement: Supplementary file 1 [file e-77-00857-sup2.zip › symandsg/Main/dharker_files/dharker.JPG]

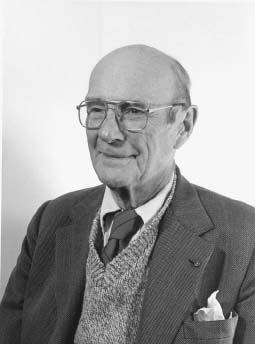

Supplement: Supplementary file 1 [file e-77-00857-sup2.zip › symandsg/Main/dharker_files/dharker_002.JPG]

Supplement: Supplementary file 1 [file e-77-00857-sup2.zip › symandsg/Main/dharker_files/webbug.gif]

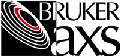

Supplement: Supplementary file 1 [file e-77-00857-sup2.zip › symandsg/Main/ecamembership_files/bruker.gif]

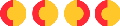

Supplement: Supplementary file 1 [file e-77-00857-sup2.zip › symandsg/Main/ecamembership_files/ccdc.gif]

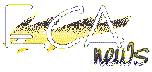

Supplement: Supplementary file 1 [file e-77-00857-sup2.zip › symandsg/Main/ecamembership_files/ecanews_bg.gif]

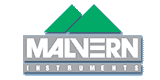

Supplement: Supplementary file 1 [file e-77-00857-sup2.zip › symandsg/Main/ecamembership_files/malvern.gif]

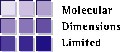

Supplement: Supplementary file 1 [file e-77-00857-sup2.zip › symandsg/Main/ecamembership_files/mdllogo.jpg]

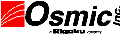

Supplement: Supplementary file 1 [file e-77-00857-sup2.zip › symandsg/Main/ecamembership_files/osmic.gif]

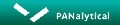

Supplement: Supplementary file 1 [file e-77-00857-sup2.zip › symandsg/Main/ecamembership_files/pan.jpg]

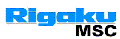

Supplement: Supplementary file 1 [file e-77-00857-sup2.zip › symandsg/Main/ecamembership_files/rigaku.gif]

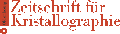

Supplement: Supplementary file 1 [file e-77-00857-sup2.zip › symandsg/Main/ecamembership_files/zkrist.gif]

Supplement: Supplementary file 1 [file e-77-00857-sup2.zip › symandsg/Main/elem_files/black_line.gif]

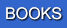

Supplement: Supplementary file 1 [file e-77-00857-sup2.zip › symandsg/Main/elem_files/books.gif]

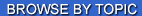

Supplement: Supplementary file 1 [file e-77-00857-sup2.zip › symandsg/Main/elem_files/browse.gif]

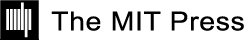

Supplement: Supplementary file 1 [file e-77-00857-sup2.zip › symandsg/Main/elem_files/colophon_arial.gif]

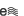

Supplement: Supplementary file 1 [file e-77-00857-sup2.zip › symandsg/Main/elem_files/email_alert.gif]
