# Supplementary figures and images for: Space groups and crystallographic symmetry: writing a multi-featured tutorial in a new style (part 1 of 2)
Source: Acta Crystallogr E Crystallogr Commun. 2021 Jul 16;77(Pt 9):857–63. doi: 10.1107/S2056989021007039 (PMC8423017; doi:10.1107/S2056989021007039)

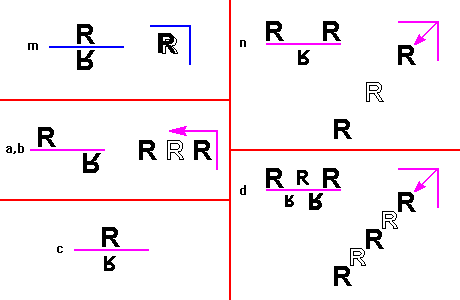

Supplement: Supplementary file 1 [file e-77-00857-sup2.zip › symandsg/Main/3DSPGRP_files/glidesym.gif]

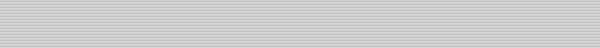

Supplement: Supplementary file 1 [file e-77-00857-sup2.zip › symandsg/Main/abs1247_files/bkgnd.gif]

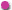

Supplement: Supplementary file 1 [file e-77-00857-sup2.zip › symandsg/Main/abs1247_files/bullet_dot.gif]

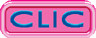

Supplement: Supplementary file 1 [file e-77-00857-sup2.zip › symandsg/Main/abs1247_files/clic_logo96.gif]

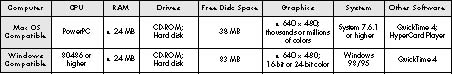

Supplement: Supplementary file 1 [file e-77-00857-sup2.zip › symandsg/Main/abs1247_files/JCE2000p1247tbl.jpg]

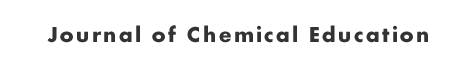

Supplement: Supplementary file 1 [file e-77-00857-sup2.zip › symandsg/Main/abs1247_files/JCEBanner.gif]

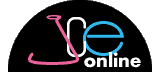

Supplement: Supplementary file 1 [file e-77-00857-sup2.zip › symandsg/Main/abs1247_files/JCELogo.gif]

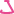

Supplement: Supplementary file 1 [file e-77-00857-sup2.zip › symandsg/Main/abs1247_files/jFlask.gif]

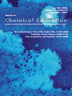

Supplement: Supplementary file 1 [file e-77-00857-sup2.zip › symandsg/Main/abs1247_files/Sep.jpg]

Supplement: Supplementary file 1 [file e-77-00857-sup2.zip › symandsg/Main/abs1247_files/spacer.gif]

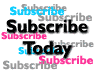

Supplement: Supplementary file 1 [file e-77-00857-sup2.zip › symandsg/Main/abs1247_files/Subscribe.gif]

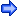

Supplement: Supplementary file 1 [file e-77-00857-sup2.zip › symandsg/Main/abso_files/sh0129bdy_data/bluearr.gif]

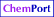

Supplement: Supplementary file 1 [file e-77-00857-sup2.zip › symandsg/Main/abso_files/sh0129bdy_data/chemportborder.gif]

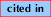

Supplement: Supplementary file 1 [file e-77-00857-sup2.zip › symandsg/Main/abso_files/sh0129bdy_data/citedinborder.gif]

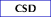

Supplement: Supplementary file 1 [file e-77-00857-sup2.zip › symandsg/Main/abso_files/sh0129bdy_data/csdborder.gif]

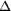

Supplement: Supplementary file 1 [file e-77-00857-sup2.zip › symandsg/Main/abso_files/sh0129bdy_data/Delta_rmgif.gif]

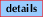

Supplement: Supplementary file 1 [file e-77-00857-sup2.zip › symandsg/Main/abso_files/sh0129bdy_data/details.gif]

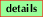

Supplement: Supplementary file 1 [file e-77-00857-sup2.zip › symandsg/Main/abso_files/sh0129bdy_data/details_002.gif]

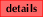

Supplement: Supplementary file 1 [file e-77-00857-sup2.zip › symandsg/Main/abso_files/sh0129bdy_data/details_003.gif]

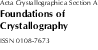

Supplement: Supplementary file 1 [file e-77-00857-sup2.zip › symandsg/Main/abso_files/sh0129bdy_data/journallogo.gif]

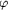

Supplement: Supplementary file 1 [file e-77-00857-sup2.zip › symandsg/Main/abso_files/sh0129bdy_data/phiv_rmgif.gif]

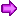

Supplement: Supplementary file 1 [file e-77-00857-sup2.zip › symandsg/Main/abso_files/sh0129bdy_data/pinkarr.gif]

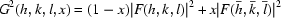

Supplement: Supplementary file 1 [file e-77-00857-sup2.zip › symandsg/Main/abso_files/sh0129bdy_data/sh0129fd1.gif]

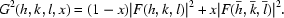

Supplement: Supplementary file 1 [file e-77-00857-sup2.zip › symandsg/Main/abso_files/sh0129bdy_data/sh0129fd2.gif]

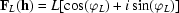

Supplement: Supplementary file 1 [file e-77-00857-sup2.zip › symandsg/Main/abso_files/sh0129bdy_data/sh0129fd3.gif]

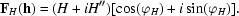

Supplement: Supplementary file 1 [file e-77-00857-sup2.zip › symandsg/Main/abso_files/sh0129bdy_data/sh0129fd4.gif]

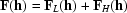

Supplement: Supplementary file 1 [file e-77-00857-sup2.zip › symandsg/Main/abso_files/sh0129bdy_data/sh0129fd5.gif]

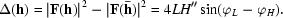

Supplement: Supplementary file 1 [file e-77-00857-sup2.zip › symandsg/Main/abso_files/sh0129bdy_data/sh0129fd6.gif]

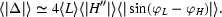

Supplement: Supplementary file 1 [file e-77-00857-sup2.zip › symandsg/Main/abso_files/sh0129bdy_data/sh0129fd7.gif]

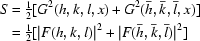

Supplement: Supplementary file 1 [file e-77-00857-sup2.zip › symandsg/Main/abso_files/sh0129bdy_data/sh0129fd8.gif]

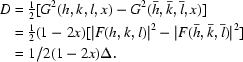

Supplement: Supplementary file 1 [file e-77-00857-sup2.zip › symandsg/Main/abso_files/sh0129bdy_data/sh0129fd9.gif]

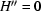

Supplement: Supplementary file 1 [file e-77-00857-sup2.zip › symandsg/Main/abso_files/sh0129bdy_data/sh0129fi2.gif]

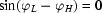

Supplement: Supplementary file 1 [file e-77-00857-sup2.zip › symandsg/Main/abso_files/sh0129bdy_data/sh0129fi3.gif]

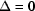

Supplement: Supplementary file 1 [file e-77-00857-sup2.zip › symandsg/Main/abso_files/sh0129bdy_data/sh0129fi5.gif]

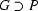

Supplement: Supplementary file 1 [file e-77-00857-sup2.zip › symandsg/Main/abso_files/sh0129bdy_data/sh0129fi6.gif]

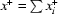

Supplement: Supplementary file 1 [file e-77-00857-sup2.zip › symandsg/Main/abso_files/sh0129bdy_data/sh0129fi7.gif]

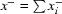

Supplement: Supplementary file 1 [file e-77-00857-sup2.zip › symandsg/Main/abso_files/sh0129bdy_data/sh0129fi8.gif]

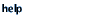

Supplement: Supplementary file 1 [file e-77-00857-sup2.zip › symandsg/Main/abso_files/sh0129hdr_data/help.gif]

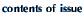

Supplement: Supplementary file 1 [file e-77-00857-sup2.zip › symandsg/Main/abso_files/sh0129hdr_data/issuecontents.gif]

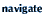

Supplement: Supplementary file 1 [file e-77-00857-sup2.zip › symandsg/Main/abso_files/sh0129hdr_data/navigate.gif]

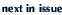

Supplement: Supplementary file 1 [file e-77-00857-sup2.zip › symandsg/Main/abso_files/sh0129hdr_data/nextinissue.gif]

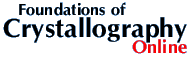

Supplement: Supplementary file 1 [file e-77-00857-sup2.zip › symandsg/Main/abso_files/sh0129hdr_data/onlinelogo.gif]

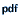

Supplement: Supplementary file 1 [file e-77-00857-sup2.zip › symandsg/Main/abso_files/sh0129hdr_data/pdf.gif]

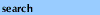

Supplement: Supplementary file 1 [file e-77-00857-sup2.zip › symandsg/Main/abso_files/sh0129hdr_data/search.gif]

Supplement: Supplementary file 1 [file e-77-00857-sup2.zip › symandsg/Main/abso_files/sh0129hdr_data/spacer.gif]

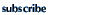

Supplement: Supplementary file 1 [file e-77-00857-sup2.zip › symandsg/Main/abso_files/sh0129hdr_data/subscribe.gif]

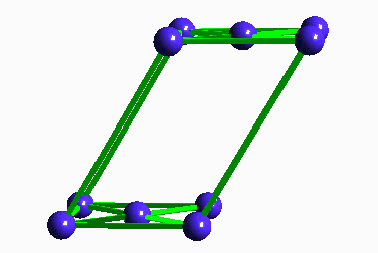

Supplement: Supplementary file 1 [file e-77-00857-sup2.zip › symandsg/Main/basemono-gif_files/basemono.gif]

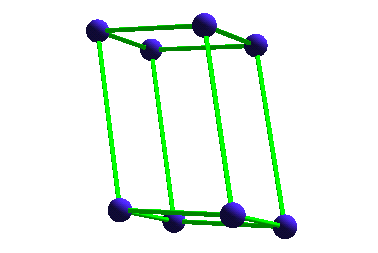

Supplement: Supplementary file 1 [file e-77-00857-sup2.zip › symandsg/Main/basemono-gif_files/monoc.png]

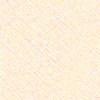

Supplement: Supplementary file 1 [file e-77-00857-sup2.zip › symandsg/Main/basemono-gif_files/paper.jpg]

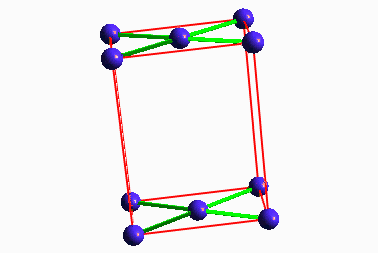

Supplement: Supplementary file 1 [file e-77-00857-sup2.zip › symandsg/Main/baseo-gif_files/baseo.gif]

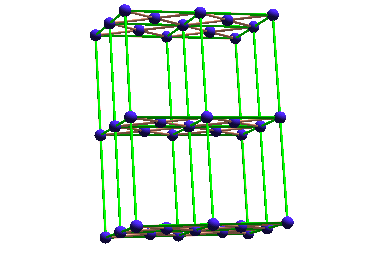

Supplement: Supplementary file 1 [file e-77-00857-sup2.zip › symandsg/Main/baseo-gif_files/baseobig.png]

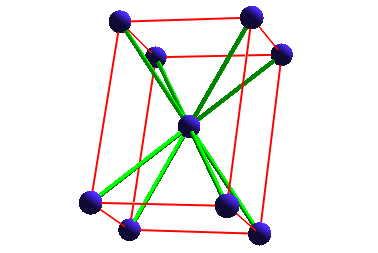

Supplement: Supplementary file 1 [file e-77-00857-sup2.zip › symandsg/Main/baseo-gif_files/bodyo.png]

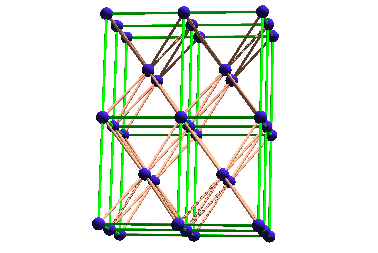

Supplement: Supplementary file 1 [file e-77-00857-sup2.zip › symandsg/Main/baseo-gif_files/bodyobig.png]

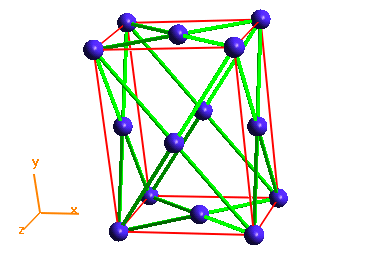

Supplement: Supplementary file 1 [file e-77-00857-sup2.zip › symandsg/Main/baseo-gif_files/faceo.png]

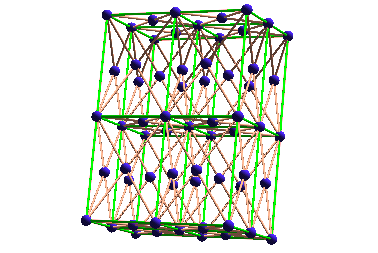

Supplement: Supplementary file 1 [file e-77-00857-sup2.zip › symandsg/Main/baseo-gif_files/faceobig.png]

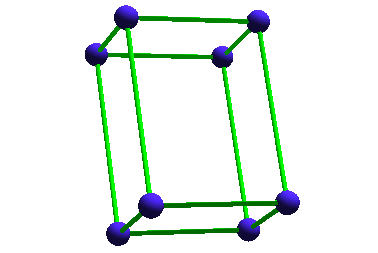

Supplement: Supplementary file 1 [file e-77-00857-sup2.zip › symandsg/Main/baseo-gif_files/i.png]

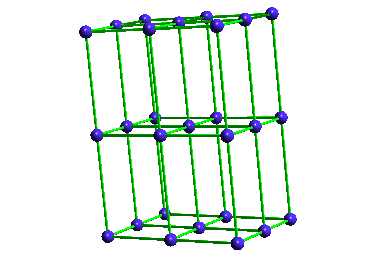

Supplement: Supplementary file 1 [file e-77-00857-sup2.zip › symandsg/Main/baseo-gif_files/ibig.png]

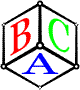

Supplement: Supplementary file 1 [file e-77-00857-sup2.zip › symandsg/Main/bcamember_files/bcalogo3.gif]

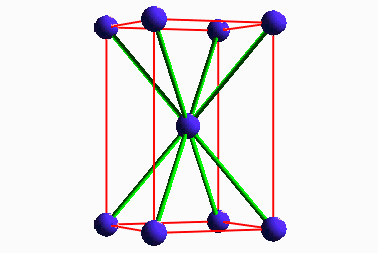

Supplement: Supplementary file 1 [file e-77-00857-sup2.zip › symandsg/Main/bct-gif_files/bct.gif]

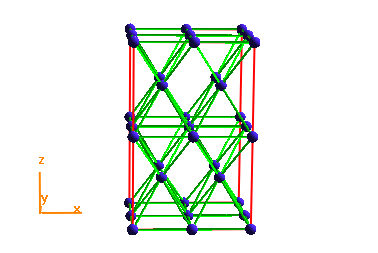

Supplement: Supplementary file 1 [file e-77-00857-sup2.zip › symandsg/Main/bct-gif_files/bctbig.png]

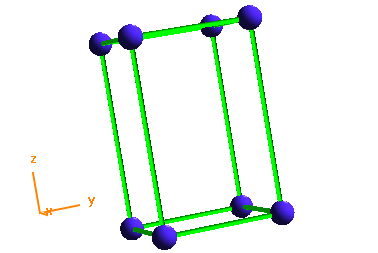

Supplement: Supplementary file 1 [file e-77-00857-sup2.zip › symandsg/Main/bct-gif_files/pa.png]

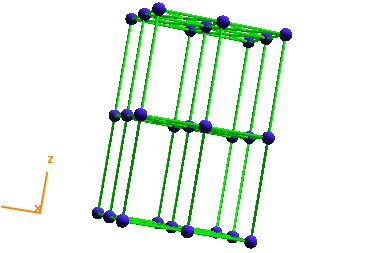

Supplement: Supplementary file 1 [file e-77-00857-sup2.zip › symandsg/Main/bct-gif_files/pabig.png]

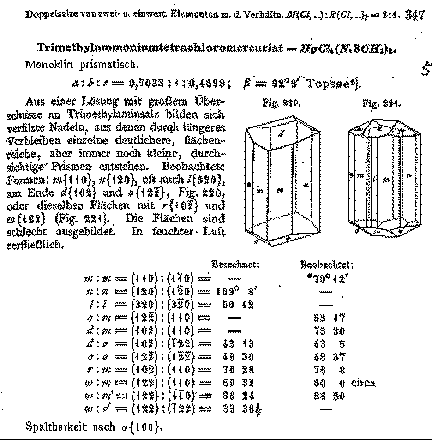

Supplement: Supplementary file 1 [file e-77-00857-sup2.zip › symandsg/Main/beev_files/Groth3.gif]

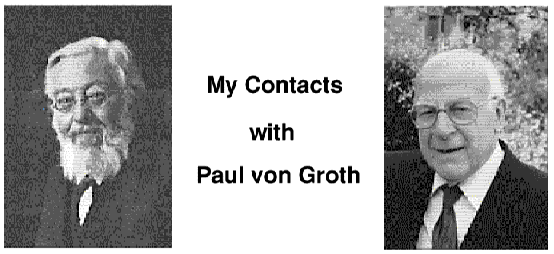

Supplement: Supplementary file 1 [file e-77-00857-sup2.zip › symandsg/Main/beev_files/PvGAB.gif]

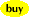

Supplement: Supplementary file 1 [file e-77-00857-sup2.zip › symandsg/Main/bennett_files/buy.gif]

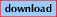

Supplement: Supplementary file 1 [file e-77-00857-sup2.zip › symandsg/Main/bennett_files/downloadborder.gif]

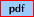

Supplement: Supplementary file 1 [file e-77-00857-sup2.zip › symandsg/Main/bennett_files/pdfborder.gif]

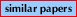

Supplement: Supplementary file 1 [file e-77-00857-sup2.zip › symandsg/Main/bennett_files/similarpapersborder.gif]

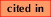

Supplement: Supplementary file 1 [file e-77-00857-sup2.zip › symandsg/Main/bertaut_files/citedinborder.gif]

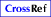

Supplement: Supplementary file 1 [file e-77-00857-sup2.zip › symandsg/Main/bertaut_files/crossrefborder.gif]

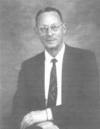

Supplement: Supplementary file 1 [file e-77-00857-sup2.zip › symandsg/Main/bertaut_files/es0336fig1thm.gif]

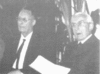

Supplement: Supplementary file 1 [file e-77-00857-sup2.zip › symandsg/Main/bertaut_files/es0336fig2thm.gif]

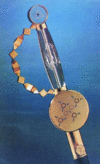

Supplement: Supplementary file 1 [file e-77-00857-sup2.zip › symandsg/Main/bertaut_files/es0336fig3thm.gif]

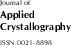

Supplement: Supplementary file 1 [file e-77-00857-sup2.zip › symandsg/Main/bertaut_files/journallogo.gif]

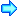

Supplement: Supplementary file 1 [file e-77-00857-sup2.zip › symandsg/Main/bertaut_files/turqarr.gif]

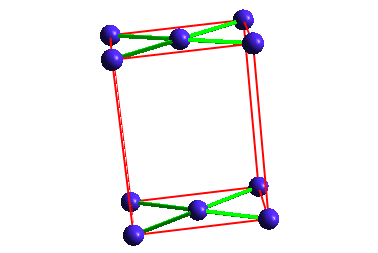

Supplement: Supplementary file 1 [file e-77-00857-sup2.zip › symandsg/Main/bodyo-gif_files/baseo.png]

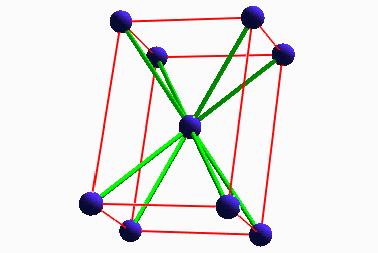

Supplement: Supplementary file 1 [file e-77-00857-sup2.zip › symandsg/Main/bodyo-gif_files/bodyo.gif]

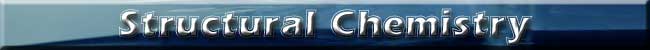

Supplement: Supplementary file 1 [file e-77-00857-sup2.zip › symandsg/Main/boese_files/Banner14en.jpg]

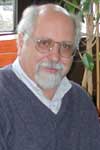

Supplement: Supplementary file 1 [file e-77-00857-sup2.zip › symandsg/Main/boese_files/boese.jpg]

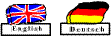

Supplement: Supplementary file 1 [file e-77-00857-sup2.zip › symandsg/Main/boese_files/deen.gif]

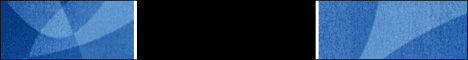

Supplement: Supplementary file 1 [file e-77-00857-sup2.zip › symandsg/Main/bravais_aug_files/637091.gif]

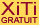

Supplement: Supplementary file 1 [file e-77-00857-sup2.zip › symandsg/Main/bravais_aug_files/hit.gif]

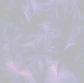

Supplement: Supplementary file 1 [file e-77-00857-sup2.zip › symandsg/Main/bravaisong_files/bImage42.gif]

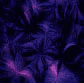

Supplement: Supplementary file 1 [file e-77-00857-sup2.zip › symandsg/Main/bravaisong_files/Image42.gif]

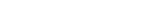

Supplement: Supplementary file 1 [file e-77-00857-sup2.zip › symandsg/Main/Buerger_files/2005ACAMeetinglogo_r1_c1.gif]

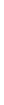

Supplement: Supplementary file 1 [file e-77-00857-sup2.zip › symandsg/Main/Buerger_files/2005ACAMeetinglogo_r2_c1.gif]

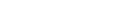

Supplement: Supplementary file 1 [file e-77-00857-sup2.zip › symandsg/Main/Buerger_files/2005ACAMeetinglogo_r3_c2.gif]

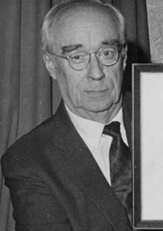

Supplement: Supplementary file 1 [file e-77-00857-sup2.zip › symandsg/Main/Buerger_files/Buerger.gif]

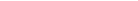

Supplement: Supplementary file 1 [file e-77-00857-sup2.zip › symandsg/Main/Buerger_files/homebutton1_r1_c1.gif]

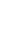

Supplement: Supplementary file 1 [file e-77-00857-sup2.zip › symandsg/Main/Buerger_files/homebutton1_r2_c1.gif]

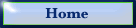

Supplement: Supplementary file 1 [file e-77-00857-sup2.zip › symandsg/Main/Buerger_files/homebutton1_r2_c2.gif]

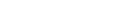

Supplement: Supplementary file 1 [file e-77-00857-sup2.zip › symandsg/Main/Buerger_files/homebutton1_r3_c2.gif]

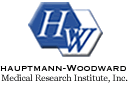

Supplement: Supplementary file 1 [file e-77-00857-sup2.zip › symandsg/Main/Buerger_files/hwilogo.gif]

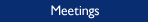

Supplement: Supplementary file 1 [file e-77-00857-sup2.zip › symandsg/Main/Buerger_files/meetings1.gif]

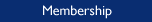

Supplement: Supplementary file 1 [file e-77-00857-sup2.zip › symandsg/Main/Buerger_files/membership1.gif]

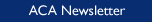

Supplement: Supplementary file 1 [file e-77-00857-sup2.zip › symandsg/Main/Buerger_files/newsletter1.gif]

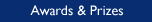

Supplement: Supplementary file 1 [file e-77-00857-sup2.zip › symandsg/Main/Buerger_files/prizes1.gif]

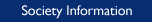

Supplement: Supplementary file 1 [file e-77-00857-sup2.zip › symandsg/Main/Buerger_files/societyinfo1.gif]

Supplement: Supplementary file 1 [file e-77-00857-sup2.zip › symandsg/Main/Buerger_files/spacer.gif]

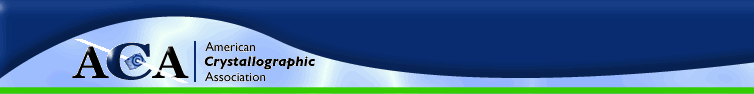

Supplement: Supplementary file 1 [file e-77-00857-sup2.zip › symandsg/Main/Buerger_files/subpage_header.gif]
