# Supplementary material for: Space groups and crystallographic symmetry: writing a multi-featured tutorial in a new style
Source: Acta Crystallogr E Crystallogr Commun. 2021 Jul 16;77(Pt 9):857–63. doi: 10.1107/S2056989021007039 (PMC8423017; doi:10.1107/S2056989021007039)
Supplement: Supplementary file 1 [file e-77-00857-sup2.zip › symandsg/Main/etter.pdf]

Chemistry of MATERIALS

VOLUME 6, NUMBER 8 AUGUST 1994

© Copyright 1994 by the American Chemical Society

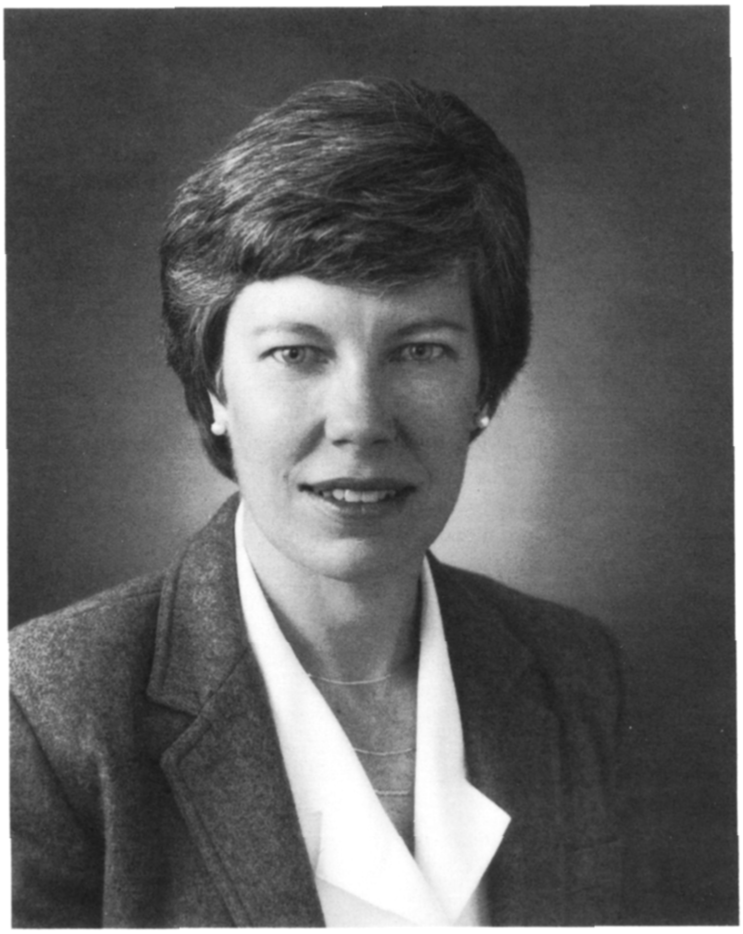

Margaret Cairns (Peggy) Etter
1943–1992

Margaret Cairns (Peggy) Etter was an inspirational force and a leader in the rapidly emerging field of solid-state organic chemistry. For those of us who knew Peggy, the cover of this issue and the quote that goes with it are especially moving, because they signify the excitement and passion that she brought to her chemistry and to the field of solid-state organic chemistry as a whole. This special issue of *Chemistry of Materials* honors her life and the contributions that she made to her field and to the lives of those who knew her. The overwhelming response to the call for papers for this issue gives only a small indication of Peggy's central role in the field of solid-state chemistry and of the number of people who counted her as a friend and a colleague.

0897-4756/94/2806-1087$04.50/0 © 1994 American Chemical Society

Peggy Etter was born and raised in Greenville, Delaware, close to Wilmington, where her father worked as a chemist at the DuPont Co. She did her undergraduate work at Cornell University and at the University of Pennsylvania, and after a short time at DuPont went on to finish her Master's degree at the University of Delaware with Harold Kwart in 1971. Peggy attended the University of Minnesota as a National Science Foundation predoctoral fellow and obtained her Ph.D. and conducted postdoctoral research with Jack Gougoutas on the solid-state chemistry and crystallography of polyvalent iodine compounds. This work resulted in several publications, including three independent ones, in which Peggy describes the clever use of X-ray diffraction and spectroscopic techniques to probe the topotaxy and twinning processes in the reactions that relate the peroxide and benzoxiodole structures and their hydrolysis products. In these early papers, Peggy revealed her long-standing interest in the relationships between crystal structures of related compounds and polymorphs and in the "rules" for packing in organic solids.

After one year as an assistant professor at Augsburg College in Minneapolis, Peggy moved to the Central Research Laboratories at 3-M Co. in St. Paul. Here she worked as a crystallographer, solid-state scientist, and polymer chemist. Peggy collaborated with a number of workers at 3-M and in academia, including Allen Siedle on chemistry and solid-state rearrangements of organometallic complexes, John Gladysz and Charles Strouse on transition-metal complexes, and Jacques Vicens on solid-state hydrolysis and packing patterns in acetylanthranyl. Her work with Illinois chemists Kress, Duesler, Paul, and Curtin showed that the solid-state resolution of racemic binaphthyl occurred primarily through a solid–vapor–solid process. She also worked with Joel Bernstein, Ruth Kress, and Derek Cash on cyanine–oxonol mixed dyes. During this time, Peggy studied a number of solid-state hydrogen-bond rearrangements and started to develop rules for hydrogen-bonding patterns in organic solids. For acids and amides, she found that crystals will typically form in such a way that the maximum number of hydrogen-bond acceptor sites will be involved in hydrogen bonding.

In 1984, Peggy moved back to the University of Minnesota for a second postdoctoral appointment in solid-state NMR spectroscopy with Robert Bryant. Within a year, she was appointed to the faculty as an assistant professor in the Department of Chemistry. For the next eight years, much of Peggy's work was devoted to understanding and predicting the properties of crystalline organic compounds, and in particular hydrogen-bonded systems. She was spectacularly successful in this work, which touched on a wide variety of prominent themes in solid-state organic chemistry, including the nature of crystal growth, polymorphism, clathrate formation and phase transitions in solids. At Minnesota, she continued to collaborate with other scientists, including crystallographers Gleason and Britton and NMR spectroscopists Bronniman, Frye, and Regitze and Robert Vold.

When Peggy started her academic career at the University of Minnesota, a prevailing attitude was that hydrogen bonding in organic solids was too complicated to understand and that exceptions far outweighed any rules that one might develop for understanding packing patterns in these crystalline solids. Her determination to establish sets of rules for hydrogen-bonding preferences and her success at that endeavor helped to transform the field of crystal engineering and molecular recognition in solids. Throughout her work, she combined X-ray diffraction, solid-state NMR, and crystal growth techniques with an intuition about crystal packing that few others have displayed. Following earlier work by Curtin and Paul, Peggy discovered that a wide variety of cocrystals could be formed simply by grinding two different molecular solids together. By combining solid-phase synthesis with measurements of second harmonic generation, she was able to survey a large number of compounds and identify cocrystals that were good candidates for NLO materials and further crystallographic study. Some of this work was featured as an invited communication in the inaugural issue of *Chemistry of Materials* in 1989.

Peggy had a superb talent to see the forest through the trees, and was extremely adept at disentangling the complex web of hydrogen bonds that hold organic solids together. In an important article in *Accounts of Chemical Research*, she outlined an extensive set of rules for hydrogen-bonding preferences, with emphasis on systems containing nitrogen, including nitroanilines, imides, diarylureas, nucleotide bases, and cocrystals of carboxylic acids with 2-aminopyrimidines. "Etter's Rules" for hydrogen bonding are now used widely by practitioners in the field, and the graph set theory of hydrogen bonding developed in her laboratory provides a framework for classifying hydrogen-bond networks into well-defined patterns that can be recognized, assigned, and compared.

To put it in her own words, Peggy was "on a roll" when the news of her kidney cancer first arrived in the spring of 1991. Almost two-thirds of her papers were published in the last five years of her life, and virtually everything was working in the laboratory. Her group had grown to twelve students, and her work was well-funded by several agencies. To Peggy and all of those who knew her, the first news of her cancer was devastating, because of its late stage of development and because the prognosis was so poor from the start. Nevertheless, during her last months, Peggy continued to embrace life and to attend to the needs of people around her. She did everything she could to put her own house in order, making sure that each of her students had secure funding for the duration of his or her Ph.D. career and that each of them had a coadvisor who could continue in her place in the event that she could no longer advise them. She continued to teach and to inspire and to devote her time and effort to her students, family and colleagues. On Thanksgiving Day in 1991, Minnesota Public Radio paid a special tribute to Peggy, who, in the midst of all her pain and turmoil, gave so much of herself to others.

Peggy Etter will be remembered not only as a superb scientist but also as a genuinely kind and helpful person who went out of her way to welcome others into her field. She played a particularly important role as a mentor to young scientists and participated in several projects that helped women and minorities in science. In 1979, she was awarded the WCCO Radio Good Neighbor Award for her contributions to high school science programs for minority students through the STEP program at 3-M.

Since her death in June of 1992, Peggy's colleagues have memorialized her in several ways. Through the contributions of her many friends, an endowed lectureship in solid-state chemistry and materials science has been established at the University of Minnesota. During the past two years, there have been four conferences that have held sessions

that have honored Peggy and her contributions to chemistry. These include the national meeting of the American Crystallographic Association (Pittsburgh, PA, August, 1992), the Gordon Conference on Physical Organic Chemistry (Holderness School, June, 1993), the Eleventh International Conference on the Chemistry of the Organic Solid State (Jerusalem, July 1993) and the Midwest Solid State Organic Chemistry Conference (Lincoln, NE, June, 1992).

Mark D. Hollingsworth
Department of Chemistry
Indiana University

Peggy Etter is survived by her husband Bill Etter, a son, Michael Latimore, a daughter, Karen Etter, her parents, Theodore and Margaret Cairns, brothers John and Richard Cairns, and a sister, Elizabeth Reveal. Our hope is that this issue of *Chemistry of Materials* will serve as a prominent tribute to her and that it will give her family and friends a permanent record of her inspiring role in her field and of the deep appreciation that her colleagues had for her.

Michael D. Ward
Department of Chemical Engineering and Materials Science
University of Minnesota
