# Supplementary material for: Space groups and crystallographic symmetry: writing a multi-featured tutorial in a new style
Source: Acta Crystallogr E Crystallogr Commun. 2021 Jul 16;77(Pt 9):857–63. doi: 10.1107/S2056989021007039 (PMC8423017; doi:10.1107/S2056989021007039)
Supplement: Supplementary file 1 [file e-77-00857-sup2.zip › symandsg/Main/ewald.pdf]

Acta Cryst. (1986). **A42**, 1–5

Obituary

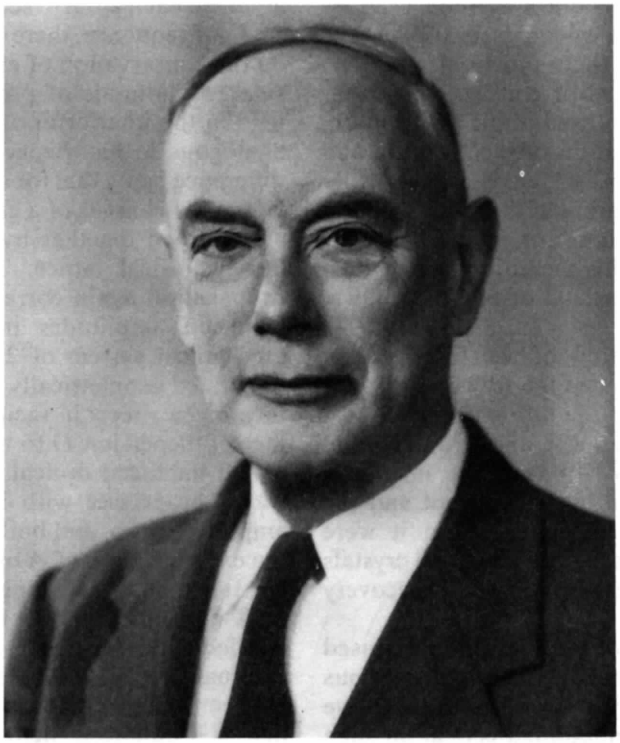

1950

Paul Peter Ewald

23 January 1888–22 August 1985

Paul Ewald, the last surviving founder of X-ray crystallography, died on 22 August 1985 at his home in Ithaca, New York. In March 1958, on the occasion of his election to the Royal Society of London, he prepared a curriculum vitae in the form of an 'obituary'. This 'obituary', which he gave to some of his friends and members of his family and which follows, discusses the problem he worked on all his life and the progress he made toward its solution.

As obituary notices go, the one on our friend P. P. Ewald is not difficult to write. Some physicists are very versatile, and their papers extend over a wide range of subjects which a single reviewer can hardly cover. Others are attracted to some problem at an early stage of their development and, if it deserves its name, it may occupy them throughout their life, leaving little chance for other production. This does not necessarily imply that their interest is confined to just that problem – it may be quite wide, even including other parts of science and arts than physics – but their own productivity in other fields appears to be postponed indefinitely until their essential problem has found an adequate solution. To all intents and purposes they remain dilettantes in general (*i.e.* knowing something of, and enjoying all science) and become experts only in their own special field. This was Ewald's type. As a lad of 15 or 16, reading Koenigsberger's biography of Helmholtz, he became interested in the problem of using the properties of light in order to find the ultimate atomic structure of matter. After trying his hand at the study of chemistry and mathematics, he was drawn to physics by the clarity and beauty of Sommerfeld's courses and finally asked his teacher for a thesis subject. When, as the last and least recommended subject on a long list offered by Sommerfeld, he found 'to discuss whether a periodic anisotropic spatial araangement of resonators leads to the laws of double-refraction', he unerringly chose this topic for his work, thus implementing a long forgotten but deeply rooted resolve.

Sommerfeld's mastery in providing a mathematically well formulated problem enabled Ewald to find a strict and affirmative answer. In the course of the research two optical fields had to be calculated. The first, the total optical field, is the sum of the fields of the spherical wavelets issuing from *all* the resonators. In modern terminology, a convenient sum is found as the Fourier transform of the sum of spherical wavelets. The second is the 'field of excitation', *i.e.* the total field minus that contribution which comes

from a particular one of the resonators. The correct subtraction of this one contribution from the total field presented some difficulties which were solved only after P. Debye pointed out a method used by Riemann in a similar case which is based on the transformation property of theta functions.

Much of Ewald's later work consists of further applications of the methods used in his dissertation. The method for the calculation of electrostatic and other potentials in crystals, which is now usually adopted, follows his elaboration of the theta function method and his interpretation, in physical terms, of the mechanism by which this method produces two quickly convergent series instead of one that slowly converges.

The Fourier transformation of the total optical field, on the other hand, opened the way for an easily visualized representation of the diffraction effects of X-rays when traversing a 'periodic, anisotropic spatial arrangement of resonators'. In fact, the now universally accepted reciprocal lattice concept and the construction of a 'sphere of reflexion' in it were developed by Ewald in the case of orthogonal crystals within a few hours after hearing of Laue's discovery of X-ray diffraction.

The mathematical rigor of the crystal model used in the thesis brought out the fact that in the previous theories of dispersion the role of the 'incident' wave was rather hazy. For the unbounded crystal no such wave is required, or even possible, and yet the laws for the phase velocity of the optical field are fully determined. In fact, the state of the unbounded crystal containing an optical wave is comparable to a single proper vibration of a mechanical system. If the crystal is cut by a plane and the half above the plane is removed, the lower half-crystal can be acted upon by an incident wave and it will assume a state comparable to the forced vibration of a mechanical model. In such a half-crystal, the incident wave (of unwanted direction and velocity) is out of order although, by the principle of superposition of fields, it would be underlying the pattern of spherical wavelets from the resonators. The conclusion is that, by limiting the resonators to a half-crystal, an extra field is created inside the crystal which serves to annihilate the incident wave. Simultaneously, there is created a field outside the crystal which is the reflected wave. Ewald showed that this leads to the Fresnel formulae for the amplitudes of the refracted and reflected waves without the need for boundary conditions in the usual sense. The same conclusion, although in a more abstract form, was arrived at by C. W. Oseen nearly simultaneously and the theorem that the surface of the body shields its interior from the incident wave is sometimes referred to as the *Ewald-Oseen'scher Auslöschungssatz.*

This deepened understanding of the phenomena of dispersion, refraction and reflection helped Ewald considerably with the much more complicated case of X-ray propagation in a crystal. The then-accepted theory of Laue was a purely kinematical theory which assumed each atom to scatter only the 'incident' wave. As a consequence, there was an obvious infringement on the conservation of energy. Ewald again first considered the mode of propagation of an X-ray wave field in the interior of an unbounded crystal. This is analogous to his theory of dispersion for light, the difference being that for a given frequency the 'optical field' now consists of a group of n plane waves which are coupled together by the sphere of reflection in the reciprocal lattice. The condition of balanced propagation again corresponds to the condition for balanced amplitudes in a proper vibration of a mechanical system of $2n$ degrees of freedom. It is illustrated geometrically by the construction of a surface of $2n$ sheets in reciprocal space, called the surface of dispersion D to which a point A representing a well balanced optical field is bound. Each of the waves progresses with its own phase velocity and amplitude ratio, and both these quantities depend on the exact location of A on any one of the sheets of D.

After thus establishing the laws of propagation of an X-optical field in the interior of the crystal, Ewald applied this knowledge to relate the internal to the external field in the case of a half-crystal. The link between the two fields is the condition that the internal, self-balanced field be not disturbed by the penetration of an incident wave to the interior. This gives a determination of the detectable amplitudes of the diffracted waves which is in keeping with energy considerations.

The dynamical theory of X-ray diffraction was conceived and carried out by Ewald while he was a medical X-ray technician in the German army from 1915 to 1918 on the front near Dwinsk that was then quiet. It seemed a long way to any experimental verification at the time, but the development of the large number of concepts required for carrying through the original idea to a successful end contained its own satisfaction. Experimental verification came earlier than expected after the war was over, in the deviations from Bragg's law discovered by the Swedish spectroscopists (Stenström *et al.*) and by Bergen Davis and von Nardroff's measurements. The concepts developed for the case of X-rays were later taken over by H. Bethe for the discussion of electron diffraction and electron motion in metals where, owing to the stronger interaction, the kinematical theory is but a poor approximation.

There are two cases in which Ewald's theory gives a complete answer: if there is only one point-scatterer per unit cell, any number of component plane waves in the optical field can be treated; and if there is any number of point-scatterers distributed throughout the cell (or a continuous electron density distribution) only two coupled waves can be strictly solved. Com-

ing back to this subject in several later papers, Ewald tried to extend the treatment to the general case, having in mind the construction of a theory embracing both long optical and short X-ray fields. This has been only partially successful.

Whereas Ewald's merits as an original thinker undoubtedly are confined to the field of crystal optics (in a wide sense), he has directed much of his efforts to the service of others. In his academic profession he has tended the development of a number of outstanding physicists who were his assistants or doctorands, among them E. Fues, F. London, H. Bethe, H. Hönl, C. Hermann, M. Renninger, U. Dehlinger and A. Kochendörfer. A large number of his former students have taken up research or are in an academic career. Apart from this professional activity, he has done much for the consolidation and development of the New Crystallography with which he was connected from its inception. It was on the insistence of P. Groth that, in 1923, he joined the new Board of Editors of the *Zeitschrift für Kristallographie*, the journal founded by Groth in 1877 and edited by him single-handedly up to his 79th year. Under the chief editorship of P. Niggli, the journal severed its close connection with mineralogy and, fulfilling the old desire of its founder, became the first journal of crystallography in 1920. Ewald, well-known internationally through his book *Kristalle und Röntgenstrahlen* (1923), received and edited a large fraction of the papers on X-ray crystal analysis. His connection with *Z. Krist.* lasted from 1924 (Vol. 60) to 1939 (Vol. 101) when it came to an end through the war.

The ever increasing spate of crystal structure determinations made it appear desirable to collect the results and apply a certain amount of criticism to the published determinations. This led to a collection, the *Strukturbericht*, which appeared as an extension volume of the *Zeitschrift*. The appearance of the first volume, covering the year 1913–28, was delayed until 1931 because Ewald and C. Hermann could not cope any quicker with the new determinations. It was utterly unexpected that this modestly planned collection amounted to over 800 pages of text. The plan of the report required much redrawing of structures and recalculation. As a first attempt of this kind of collection it was a success, and it remains a standard source of information on early crystal analysis. Ewald has not taken part in the preparation of later volumes except in a consulting fashion. The successor to the series of *Strukturbericht* is the present volumes of *Structure Reports.*

Ewald's book *Kristalle und Röntgenstrahlen* was sold out quickly, but a second edition was delayed by his writing an article for the *Handbuch der Physik* which covered the same subject matter more succinctly. It appeared in 1927 (Vol. 24), and was brought up to date in a second edition in 1933 (now Vol. 23/2). Unfortunately these articles, which would have served very well as a second and third edition of the original book, were not released by the publisher for publication or even translation outside the unwieldy *Handbuch* volume.

The planning of a second edition brought up the following problem. By 1929 at least half a dozen books on X-ray crystal analysis had appeared and in most of them, in order to facilitate their use, elaborate tables of point groups, space groups, coordinates of equivalent positions, *etc.* and also X-ray wavelengths, absorption coefficients and the like were given. Worse, some of the tables contained many errors and few of them were alike in using the same conventions regarding setting or choice of origin. Any future author whose book was to be of practical help in structure determination would have to waste space and effort on another such set of tables. It was bearing this in mind that Ewald, strongly seconded by Bernal, convinced Sir William Bragg at a meeting in the Royal Institution in 1929 that *International Tables for the Determination of Crystal Structures* should be prepared for which subsidies would be sought from various Academies, Governments or Foundations. The plans for these *Tables* were discussed at a conference in Zürich in 1930 in Niggli's Institute which brought together practically all previous authors of such textbooks or tables. Ewald and Bernal had undertaken the preparation of this conference and Ewald chaired the meeting which lasted the greater part of a week. By the end of the meeting general agreement had been reached on the contents of the two-volume work, the nomenclature and setting questions, the style of figures for the representation of the space groups, the contents of Vol. 2 - physical and mathematical tables - and all other points including the designation of C. Hermann as Editor and Bornträger as publisher. The book appeared in 1935 and soon became the standard reference for all those engaged in crystal structure analysis.

Some of the funds for continuing the *Strukturbericht* were granted by the International Union of Pure and Applied Physics (IUPAP) of which Sir William Bragg was then President. The early history of this Union is a record of inactivity caused by the slowness of converting the scientific Unions from an instrument of the Allied General Staff during World War I to unpolitical scientific organizations. Ewald greatly appreciated the kindness with which his request for funds was treated in the 1930's and he became much interested in the Scientific Unions. It was natural that he agreed to become Secretary General of IUPAP when asked by the President, Manne Siegbahn, at the end of World War II to re-establish IUPAP, which had very nearly lost even the limited existence it enjoyed before the war. Ewald took eagerly to this task and arranged for a General Assembly in Paris on 3 and 4 January 1947. As a result, IUPAP was put in working condition, the National Committees were

reformed, and further work was left in the hands of an able Secretary General, Professor P. Fleury.

The reason for Ewald withdrawing from further active participation in the affairs of IUPAP (he remained Vice-President for some time) lay in his involvement with the establishment of a separate International Union of Crystallography. In an address to the X-ray Group of the Institute of Physics given in Oxford, 31 March 1944, he pleaded strongly for the establishment of such a Union and stated its tasks. From that time on, Ewald together with R. C. Evans were the main promoters of this idea and, after a preparatory meeting of crystallographers from many countries had been held in London under the auspices and chairmanship of Sir Lawrence Bragg in 1946, Ewald and Evans worked out draft statutes for the new Union. At its first General Assembly in Harvard University, July 1948, the statutes were accepted and the new International Union of Crystallography was formally established at the business meeting under Ewald's chairmanship; Sir Lawrence Bragg was elected President and M. von Laue Honorary President.

At the 1946 meeting in London, Ewald was asked to organize an international journal of crystallography to replace the *Zeitschrift für Kristallographie* which had succumbed to the war and for which there was very little hope of revival. He was to act as Editor in Chief with a British, an American and a French co-editor at his side (R. C. Evans, I. Frankuchen and J. Wyart). The present name, *Acta Crystallographica*, was proposed by A. V. Shubnikov, who was designated Russian co-editor but who retired from the board of editors before the first three issues appeared in 1948. Owing to its unique position as the organ of publication for a rapidly advancing and expanding field of science, *Acta Crystallographica* developed rapidly into one of the important international scientific journals. It slowly but steadily increased its distribution in physical, chemical, metallurgical, mineralogical and biological laboratories throughout the world. The revived *Zeitschrift für Kristallographie* and a new Russian journal, *Kristallographiya*, are now publishing in the same field. It is with a sense of gratitude and obligation toward his old teacher Groth that Ewald has continued to edit crystallographic research.

In his academic profession, Ewald enjoyed the experience of teaching mathematical physics for 18 years in Germany (University of Munich and Technische Hochschule in Stuttgart), for 10 years in Britain (The Queen's University, Belfast) and for 9 years in the USA (Polytechnic Institute of Brooklyn, New York). His students liked and respected him. In the critical period before Hitler took over in Germany, he volunteered to be Rector of the Technische Hochschule and, although he could not stem the rising tide of Nazism, he managed to avoid the troubles which Rectors encountered at other Universities. He resigned the rectorate after a law was promulgated by which all Jewish professors were dismissed. The Nazi student body expressed their qualified regret in a letter at parting from a rector whose views they could not share but whose fairness and personality they otherwise respected! Before handing back his office, Ewald had seen to it that he was succeeded by a non-Nazi and, since this was successful a second time, it preserved the Technische Hochschule at Stuttgart for three more years from Nazi governance. When this finally took place Ewald, on returning in 1936 from his first visit to the United States, soon broke off his relations with the Technische Hochschule and was pensioned. The University of Cambridge offered him a Research Grant and he emigrated to England in October 1937, bringing his family there in April 1938. He was appointed in April 1939 as Lecturer in Mathematical Physics in Belfast and the family followed him in August. In spite of the war and the deportation of his youngest son to Australia in the Dunkirk period, the Ewalds (including his mother, the painter) felt very happy in Belfast owing to the great kindness of their colleagues, neighbors and friends. Ewald's mother died in 1948, aged 88, and since retirement would have brought his connection with Queen's University to an end a little later, he accepted an offer to become Head of the Physics Department and Professor of Physics at the Polytechnic Institute of Brooklyn. The warm-hearted acceptance by his American colleagues, the challenge of developing the Physics Department from a service-oriented department to one doing research in its own right, and the central situation of New York for international connections made the nine years of Ewald's activity at the Polytechnic Institute very happy ones.

Ewald has served on many committees both in England and the USA. He was President of the American Crystallographic Association in 1952. As an officer of IUCr, he was a member of the USA National Committee for Crystallography from 1950 until 1966.

The first academic distinction Ewald received was to be made Corresponding Member of the Göttingen Academy in 1936, which was doubly appreciated because of the date of his election. He became in succession a Fellow of the American Academy of Arts and Science in 1955, Dr. h. c. of the Technische Hochschule, Stuttgart in 1954, Membre d'honneur, Société Française de minéralogie et de cristallographie in 1955, Fellow of the Royal Society of London in 1958, and Ehrenmitglied der Deutschen Mineralogischen Gesellschaft, also in 1958.

Ewald strove to be remembered by his juniors and students as a friend and not as a boss (even if he did not always achieve this), by his friends as reliable and honest, and by his fellow scientists as a faithful servant to science. *RIP.*

Ewald was only 73 years old when he wrote this notice in 1959. He continued to work on his 'thesis problem' until he was in his nineties. He edited and wrote major portions of *Fifty Years of X-ray Diffraction* (1962) and several additional publications. A previously unpublished paper written when he was 88 years old will appear in the forthcoming Ewald Memorial issue of *Acta Crystallographica.*

In recent years his dynamical theory has been applied by workers in the field to X-ray interferometry, to crystal topography, to the experimental solution of the X-ray phase problem and to a wide range of synchrotron X-radiation applications.

He continued as Editor of *Acta Crystallographica* until 1960. He was president of IUCr from 1960 to 1963, he was awarded an honorary doctorate by the University of Paris in 1958, by Adelphi University in 1966 and by the University of Munich in 1967. He was made a Fellow of the Akademie Deutscher Naturforscher (Leopoldina) in 1966.

The January 1968 issue of *Acta Crystallographica* was dedicated to him on the occasion of his 80th birthday. That issue contains one of his major scientific publications and a three page article on 'Personal Reminiscences' which includes many interesting biographical items. More than 250 scientists from four continents gathered at the University of Oklahoma on 23 March 1979 to honor Professor Ewald in his 90th year with a day-long *Dynamical Diffraction Symposium.* The first paper was presented by Professor Ewald. He received standing ovations before his talk and after its completion.

On his 90th birthday he was given the Max Planck medal of the German Physical Society. (At a recent memorial meeting, H. A. Bethe recounted that Ewald's election by previous medalists was unanimous, unprecedented in the history of the Society.) The following year, he was awarded the first Gregori Aminoff Medal of the Royal Swedish Academy of Sciences in 1979, a prize given annually for outstanding contributions to crystallography.

Ewald is remembered with affection and esteem as a 'faithful servant of science' as well as for his personal qualities, his friendliness, his scrupulous honesty, his wide-ranging interest in ideas and people, and his wit and unfailing humor.

J. J. Dropkin
B. Post
Polytechnic Institute of New York
