# Supplementary material for: Space groups and crystallographic symmetry: writing a multi-featured tutorial in a new style
Source: Acta Crystallogr E Crystallogr Commun. 2021 Jul 16;77(Pt 9):857–63. doi: 10.1107/S2056989021007039 (PMC8423017; doi:10.1107/S2056989021007039)
Supplement: Supplementary file 1 [file e-77-00857-sup2.zip › symandsg/Main/frame_patt1.html]

6. The Patterson function


<body>
To access this tutorial without frames, open one window on each of these
links (middle or right mouse button):
<ul>
<li><a href="app\_patt1.html">Structure factor applet</a>
<li><a href="tut\_patt1.html">Tutorial script</a>
</ul>
</body>
