# Supplementary material for: Space groups and crystallographic symmetry: writing a multi-featured tutorial in a new style
Source: Acta Crystallogr E Crystallogr Commun. 2021 Jul 16;77(Pt 9):857–63. doi: 10.1107/S2056989021007039 (PMC8423017; doi:10.1107/S2056989021007039)
Supplement: Supplementary file 1 [file e-77-00857-sup2.zip › symandsg/Main/frame_patt1_files/app_patt1.htm]

Structure factor tutorial

|  |  |
| --- | --- |
|  | Introduction 1. Miller Indices.  2. Structure factors (1).  3. Structure factors (2).  4. Symmetry (1).  5. Symmetry (2).  6. Pattersons (1).  7. Pattersons (2).  8. Direct Methods.  Tutorial Script  by  Kevin Cowtan  email |
