# Supplementary material for: Space groups and crystallographic symmetry: writing a multi-featured tutorial in a new style
Source: Acta Crystallogr E Crystallogr Commun. 2021 Jul 16;77(Pt 9):857–63. doi: 10.1107/S2056989021007039 (PMC8423017; doi:10.1107/S2056989021007039)
Supplement: Supplementary file 1 [file e-77-00857-sup2.zip › symandsg/Main/frame_patt1_files/tut_patt1.htm]

Structure factor tutorial

# 6. Patterson (1)

In this section we will look at the Patterson function, which was used
to solve many early crystal structures and is still important in the
location of heavy atoms.

The biggest problem in X-ray crystallography is our inability to
measure phases directly. A single diffraction experiment only yields
diffraction intensities, from which we can calculate the structure
factor magnitudes, **|F(h)|**, but not their phases.

What happens if we ignore the phases and calculate a Fourier transform
just using the information we have? For example, we could set all the
phases to zero. It is convenient also to square the structure factor
magnitudes.

The resulting map is called the Patterson function, **P(x)**. It may be expressed by the Fourier transform as follows:

| P(x) | = | 1/V_h_\|F(h)\|^2^exp( -2i h.x) |
| --- | --- | --- |
|  | = | 1/V_h_F(h)F(-h)exp( -2i h.x) |

from the Friedel relationship.

The convolution theorem states that the Fourier transform of a product
is equal to the convolution of the Fourier tranforms of the original
functions. Since the Fourier transform of **|F(h)|** is **(x)**, and the Fourier transform of
**|F(-h)|** is **(-x)**, **P(x)**
must be the convolution of **(x)** and
**(-x)**:

| P(x) | = | _y_(y)(x+y) dy |
| --- | --- | --- |

Therefore **P(x)** must be large anywhere that both **(y)** is large and **(x+y)** is large for any **y**, in other words,
whenever **x** is an interatomic vector.

In the window above there are two copies of the Structure Factor
applet (scroll down to see the second). The first shows the Patterson
resulting from the two marked atoms, and the second shows the electron
density itself.

The Patterson shows a big origin peak. This corresponds to the vector
from every atom in the structure to itself (in this case two
atoms). There are also peaks above and to the right, and below and to
the left, representing the vectors from the first to the second atom
and from the second to the first atom. Clearly the Patterson must
always be centrosymmetric.

Check some of the structure factors to confirm that the fourier
coefficients of the Patterson are equal to the squares of the
structure factor magnitudes, and that all the phases are zero.

---

Up Introduction
