# Supplementary material for: Space groups and crystallographic symmetry: writing a multi-featured tutorial in a new style
Source: Acta Crystallogr E Crystallogr Commun. 2021 Jul 16;77(Pt 9):857–63. doi: 10.1107/S2056989021007039 (PMC8423017; doi:10.1107/S2056989021007039)
Supplement: Supplementary file 1 [file e-77-00857-sup2.zip › symandsg/Main/Franklin,_Rosalind.html]

CWP at physics.UCLA.edu // Franklin


|  |  |
| --- | --- |
| **Welcome to CWP at UCLA** |  |

**86 Eminent Physicists** 

**Search the Archive**

**Fascinating Documents**

**Annotated
Photo
Gallery**

**In
Her Own Words**

**Some Physics History**

**500+ Books and Articles**

**Project Staff**

**Field Editors**

**Project Support**

**Photo Credits**

**Copyright Notice**

#### Crystallography

 

|

|  |

|  |  |  |
| --- | --- | --- |
| Contributions  ---  Publications  ---  Honors  --- | Rosalind Franklin   1920-1958 | Jobs/Positions  ---  Education  ---  Additional Information  --- |

> As a scientist Miss Franklin was distinguished by extreme clarity
> and perfection in everything she undertook. Her photographs are
> among the most beautiful X-ray photographs of any substance ever
> taken. Their excellence was the fruit of extreme care in preparation
> and mounting of the specimens as well as in the taking
> of the photographs.
>
> -- J. D. Bernal [1958 N]

### Some Important Contributions:

Best known for her work on DNA, not only the
excellent X-ray diffraction photographs which she
obtained by painstaking and systematic work, but also her insight
into what they implied.

> Rosalind Franklin made crucial contributions to the solution of the structure
> of DNA. She discovered the B form, recognized that two states of the DNA
> molecule existed and defined conditions for the transition. From
> early on, she realized that any correct model must have the phosphate groups on the outside of the molecule.
> She laid the basis for the quantitative study of the diffraction patterns, and after the formation of the
> Watson - Crick model she demonstrated that a double helix was consistent with the X-ray patterns of both
> the A and B forms.
> -- Sir Aaron Klug
> [1968 N]

Her colleague Maurice Wilkins, without obtaining her permission, made
available to Watson and Crick her then unpublished X-ray diffraction
pattern of the B form of DNA , which was crucial evidence for the
helical structure. In his account of this discovery, Watson wrote

> "The instant I saw the 
> picture 
> my mouth fell open and my pulse began to race.... the black cross of
> reflections which dominated the picture could arise only from a helical
> structure... mere inspection of the X-ray picture gave several of the
> vital helical parameters." [dh1980jw]

Work on tobacco mosaic virus:

> "Using the method of isomorphous replacement, she
> showed that the virus particle was not solid, as had
> been previously thought, but actually a hollow tube. ...
> [Her work] showed that the ribonucleic acid was not to be
> found in the central cavity but embedded in the protein."
> -- J. D. Bernal [1958 N]

After her untimely death, her unpublished hypothesis that TMV RNA is a single-strand helix was confirmed. [dsb1981ro] [1958 N]

Early work on carbons:

"In a series of beautifully executed researches, she discovered
the fundamental distinction between carbons that turned into graphite
on heating and those that did not, and further related this difference
to the chemical constitution of the molecules from which the chars were
made." -- J. D. Bernal
[1958 N]

### Some Important Publications:

"Molecular Configuration in Sodium Thymonucleate," *Nature* 171: 740 (1953), with R.G. Gosling.

"The Structure of Sodium Thymonucleate Fibres:
I. The Influence of Water Content," *Acta Crystallographica* 6: 673 (1953), with R.G. Gosling.

"Evidence for a 2-chain Helix in the Crystalline
Structure of Sodium Deoxyribonucleate," *Nature* 172: 156 (1953), with R.G. Gosling.

"Tobacco Mosaic Virus: An Application of the Method
of Isomorphous Replacement to the Determination of the Helical
Parameters and Radial Density Distribution," *Acta Crystallographica* 11: 213 (1958).

"The Structure of Viruses as Determined by X-ray Diffraction," *Plant Pathology: Problems and Progress, 1908-1958*,
C.S. Holton, et al. (eds.), University of Wisconsin Press 1959, with
D.L.D. Caspar and A. Klug. This was published posthumously, and is
introduced with A
tribute to Dr.Franklin
by W.M. Stanley.

"The Interpretation of Diffuse X-ray Diagrams of Carbon," *Acta Crystallographica* 3: 107 (1950).

"Crystallite Growth in Graphitizing and Nongraphitizing Carbons," *Proceedings of the Royal Society* A209: 196 (1951).

### Honors

1951-58 Turner-Newall Research Fellowship

The
Nobel Prize for Physiology and Medicine was awarded to Watson, Crick,
and Wilkins in 1962 for their DNA work. Owing to her untimely death in
1958, the award could not have gone to Franklin. The view
has been expressed that her contribution
to the discovery was comparable to those who did receive the award. [26 SBM]

### Jobs/Positions

1942-1946 Assistant Research Officer, British Coal Utilization Research Association (CURA)   
1947-1950 Cherucheur, Laboratoire Central des Services
Chimiques de l'�tat, Paris   
1951-1953 Turner-Newall Research Fellow, King's College, University of London  
1953-1958 Turner-Newall Research Fellow, Birkbeck College, University of London

### Education

B.A. Newnham College, Cambridge University 1941  
Ph.D. Cambridge University 1945

### References consulted

[dm1996cds], [5A11 DSB], [n1974fc], [pp1959rf], [wos1990mt], [26 SBM], [rfd1975as], [1968 N], [1958 N], [dh1980jw]

**Biographies of
Rosalind Franklin and some further accounts of her role in the discovery of the structure of DNA :**

Anne Piper ***Light on a Dark Lady***

Ann Sayre, ***Rosalind Franklin and DNA,*** W.W. Norton & Co., New York 1975.

James Watson,  *The Double Helix: A Personal Account of the Discovery of the Structure of DNA,*  Atheneum, New York 1968.
The Norton Critical Edition of this book (W. W. Norton & Co. 1980) has included
some reviews, commentaries, and original papers.

Horace Freeland Judson, "Annals of Science: the Legend of Rosalind Franklin," *Science Digest* 94: 56 (January 1986).

Aaron Klug, "Rosalind Franklin and the Discovery of the Structure of DNA," *Nature* 219: 808 (1968).

Max Perutz, Letter to the Editor, *Science* 164: 1537 (1969).

### Additional Information/Comments

Some of Franklin's important contributions to the discovery of the
structure of DNA were posthumously reported by Francis Crick and
James Watson. For example, Watson reports that they had to abandon
their early three chain
model of DNA after she pointed out that
the phosphates in the molecule were likely hydrated and on the outside.[dh1980jw]

Frances Crick wrote that

> "Rosalind Franklin was only
> two steps away from the solution [of the structure of DNA]. She needed to realize that the two [sugar-phosphate]
> chains must run in opposite directions and that the bases, in their correct tautomeric form, were paired together."
> [n1974fc]

The historian of science Robert Olby described Franklin as

> " a deft experimentalist, keenly observant and with an immense capacity
> for taking pains. As a result she was able with difficult material to
> achieve a remarkable standard of resolution in her X-ray diagrams.
> Although a bold experimentalist, she was critical of speculation,
> favoring an inductive approach which proved very successful in her work
> on coals and TMV but which allowed others to get ahead of her in her
> work on DNA." [5A11 DSB]

At the request of The Royal Society (London) Franklin mounted an exhibit at the World's Fair in Brussels (1958) on
the structure of DNA and
the structure of small viruses.

Franklin enjoyed French culture and French conversation; Ann Sayre reports that "she was credited by her
[French]
colleagues with speaking the best French any of them had ever heard in a foreign mouth." [rfd1975as]

---

#### Submitted by:

Martha Keyes  
<`secwp@physics.ucla.edu`>

#### Field Editor: Professor Kenneth Trueblood, UCLA

<`knt@chem.ucla.edu`>

 Copyright � CWP and Regents of the University of California 1997

***To cite this citation:***  
" Franklin, Rosalind." *CWP*   
< http://www.physics.ucla.edu/~cwp>

---

**BACK TO THE TOP**


---


Last revision 5/16/97, 4/27/01(Piper article linked) nb
