# Supplementary material for: Space groups and crystallographic symmetry: writing a multi-featured tutorial in a new style
Source: Acta Crystallogr E Crystallogr Commun. 2021 Jul 16;77(Pt 9):857–63. doi: 10.1107/S2056989021007039 (PMC8423017; doi:10.1107/S2056989021007039)
Supplement: Supplementary file 1 [file e-77-00857-sup2.zip › symandsg/Main/gfriedel.html]

GEORGES FRIEDEL (1865-1933)

## Georges FRIEDEL (1865-1933)

- Biographie de Georges FRIEDEL, par F. Grandjean (1934)
- L'oeuvre de Georges FRIEDEL en minéralogie et cristallographie, par R. Hocart (1939)
- G. FRIEDEL et l'imprimerie Berger-Levrault
- G. FRIEDEL sur le site de Jussieu (Alain Soyer)

Georges Friedel, né à Mulhouse en 1865, décédé à Strasbourg en 1933, était le fils de Charles Friedel
et de sa première épouse née Emilie Koechlin (1837-1871). Il épousa en
1888 Mlle Hélène Berger-Levrault, fille d'Oscar Berger-Levrault et
soeur d'Alfred Berger-Levrault, d'une famille d'imprimeurs originaire
d'Alsace et installée à Nancy depuis 1871. Il eut plusieurs enfants :
Marie (qui soigna ultérieurement son père atteint d'emphysème de 1930 à
sa mort), Jeanne (épouse de Joseph Roederer, aciériste), Marguerite
(épouse de Jules Louis Crussard, 1876-1959, X 1895, corps des mines), Charles (1893-1970, PDG de la société BERGER-LEVRAULT), Edmond, Emilie (épouse de Jean Brustlein, industriel).
  
Jules Louis Crussard et Marguerite Friedel eurent eux-mêmes 3 fils
polytechniciens : Jean (1911-1986 ; X 1930), François (1911-1935 ; X
1930) et Charles (né en 1916 ; X 1935 corps des mines, docteur d'Etat
en mathématiques qui occupa lui-même des fonctions importantes à
l'IRSID, Péchiney, Ciments Lafarge).

---


  
Georges Friedel, élève de Polytechnique  
*(C) Collections
Ecole polytechnique* 


---


### Biographie de Georges Friedel par F. GRANDJEAN

*Publiée dans le Bulletin de la Société Française de Minéralogie, 1935*

Le 11 décembre 1933 est mort à Strasbourg, après une très longue
maladie qui lui interdisait depuis plusieurs années toute activité
physique, mais lui laissa jusqu'au bout sa merveilleuse lucidité et le
même intérêt passionné pour sa science, le grand cristallographe
Georges Friedel. Il était membre de la Société Française de Minéralogie
depuis 1891 et il ne cessa de publier presque chaque année dans notre
bulletin les travaux si personnels que nous avons tous admirés. C'est
ainsi qu'il tenait une grande place parmi nous, bien qu'il n'ait
presque jamais assisté à nos séances, car il fut toute sa vie,
volontairement, un provincial. Sa perte est cruellement ressentie par
tous ceux qui l'ont connu, par notre société, et surtout, car elle
dépasse de beaucoup le cercle de ses amis et de ses collègues, par la
Science minéralogique dont il fut et restera un des maîtres les plus
éminents.

Sa famille comptait déjà des savants célèbres. Duvernoy,
professeur au Collège de France et au Muséum, successeur de Cuvier,
était le grand-père maternel de Charles Friedel,
c'est-à-dire un arrière-grand-père de Georges. Le grand chimiste et
excellent cristallographe Charles Friedel, père de Georges, était
membre de l'Institut, professeur de minéralogie, puis de chimie
organique à la Faculté des Sciences. Il était aussi conservateur des
collections de minéralogie à l'École supérieure des Mines et il eut
toujours à ce titre, dans cette École, un petit laboratoire, et même,
jusqu'en 1880, son appartement. Georges Friedel, bien que né à
Mulhouse, habita donc l'École des Mines pendant toute son enfance et
jusqu'à 15 ans.

Il est vraisemblable que ce furent ces fortes attaches avec
l'École qui fixèrent l'orientation de ses études. Il fut admis à
l'Ecole Polytechnique en 1885 et en sortit en 1887 pour entrer à
l'École des Mines comme élève-ingénieur. Mallard
fut son professeur de minéralogie. A cette époque les élèves-ingénieurs
restaient trois ans à l'École, ce qui leur laissait quelques loisirs et
leur permettait de faire des études personnelles. Georges Friedel en
protita pour étudier sérieusement les minéraux, pour lesquels il avait
un goût naturel très vif. Mallard eut certainement alors une très
grande influence sur sa formation, mais son père, conservateur des
collections, le conseillait aussi. Il l'associait même à ses travaux.
Plusieurs synthèses ont été faites à cette époque, par Charles et
Georges Friedel pendant que Georges était élève. L'heureux bénéficiaire
de cette remarquable direction scientifique devait faire grand honneur
à ses maîtres.

Nommé ingénieur ordinaire des Mines en 1891, puis, chargé du
sous-arrondissement de Moulins, Georges Friedel attendit la première
vacance à l'École des Mines de Saint-Etienne pour y entrer comme
professeur, selon l'usage des jeunes ingénieurs des Mines qui veulent
se consacrer à la science. Il fut d'abord chargé des cours d'analyse
minérale et de métallurgie du fer (1893), puis des cours de physique,
de minéralogie, de géologie et de conférences sur les applications de
l'électricité à l'exploitation des mines (1894). Il continua jusqu'en
avril 1894 à assurer le service de Moulins en même temps qu'il faisait
ses leçons à Saint-Ëtienne. Ce fut certainement pour Friedel une
période de grand travail car il fut aussi consciencieux comme ingénieur
qu'il le fut comme professeur et plus tard comme directeur.
Nous avons même de lui, pendant cette période à demi technique, une
note sur " les systèmes de fermeture des recettes des puits de mines
dans la région de Commentry " qu'il est assez curieux de rencontrer
parmi les travaux d'un cristallographe, d'autant plus que Friedel avait
déjà publié, seul ou en collaboration avec son père, plusieurs études
de minéraux.

En 1899, Friedel ne professait plus que la minéralogie et la géologie. En 1907, il était nommé directeur de l'École des Mines de Saint-Étienne
et il ne gardait plus, dès 1908, que le seul cours de minéralogie.
Ensuite vint la guerre. Le retour des provinces perdues, avec ce qu'il
apportait de réconfort et de promesse à un homme de souche alsacienne,
devait réussir à arracher Friedel à cette École de Saint-Étienne où il
avait passé tant d'années, toujours dévoué à son service, et qu'il
avait plusieurs fois refusé de quitter pour une chaire à l'École des
Mines de Paris. Certes Friedel ne pouvait résister à l'appel de la
petite patrie, qu'il n'avait connue qu'occupée et dont il avait été
banni pour dix ans, et aussi à la joie de revoir libre sa propriété de
famille à Graffenstaden, aux environs de Strasbourg, où il s'était plu
à passer bien souvent une partie de ses vacances. Friedel posa donc sa
candidature à l'Université de Strasbourg.

En 1919, il y fut nommé directeur de l'Institut des Sciences
Géologiques et chargé des leçons de minéralogie et de cristallographie.
Il professa jusqu'en 1930, où il prit sa retraite. La maladie ne lui
permit pas, pendant ses dernières années, de faire ses cours et il dut
se faire suppléer par son fils Edmond.
Bientôt même elle s'aggrava et il lui fut impossible de quitter la
chambre. Sa femme était morte en 1920. Ce fut sa fille Marie qui
assista son père en toutes choses avec un admirable dévouement et vécut
avec lui jusqu'à la fin.

A cette vie de travail ne manquèrent pas les honneurs, bien que
Friedel ne les ait jamais recherchés. Il était membre correspondant de
l'Académie des Sciences depuis 1917 [élu en remplacement de M.
Vasseur], inspecteur général des Mines, collaborateur principal à la
Carte géologique de France. L'Académie des Sciences lui avait décerné
le prix Delesse
en 1905 et le prix Joseph Labbé en 1917. L'Université de Lausanne
l'avait nommé docteur Honoris Causa. Il était professeur honoraire à
l'Université de Strasbourg, officier de la Légion d'honneur,

L'oeuvre scientifique de Georges Friedel est considérable et
très inégalement partagée entre de nombreux sujets. Deux d'entre eux,
c'est-à-dire les macles, avec la loi de Bravais, et les « stases »
mésomorphes de la matière sont de beaucoup les plus importants. Après
eux vient la croissance et la décroissance des cristaux. Friedel a
étudié aussi les zéolithes, le diamant, le quartz, des cristaux de
synthèse, la mélanophlogite, la boléite et ses congénères, le sel gemme
difforme des marnes salifères et beaucoup d'autres minéraux. La
radioscopie des cristaux lui doit des remarques importantes. Il fut
aussi géologue et pétrographe en compagnie de Pierre Termier et ses
observations contribuèrent à la découverte des premières mylonites dans
notre pays.

Ses premiers travaux furent des synthèses qu'il fit dans le
tube d'acier à garniture de platine qui avait déjà servi à Ch. Friedel
et à E. Sarasin. On sait qu'il suffit de chauffer dans ce tube, vers
500°, en présence d'un excès d'eau, c'est-à-dire à de fortes pressions,
toutes sortes de substances minérales pour avoir de belles
cristallisations. Beaucoup de silicates naturels ont été reproduits de
cette manière. En collaboration avec son père, Georges Friedel réussit
à préparer la néphéline, la leucite non mimétique en cristaux simples
quadratiques, la sodalite, une hydronoséane, la cancrinite, des
feldspaths. Plusieurs de ces synthèses étaient nouvelles. A partir de
1891 Georges Friedel continua seul. Il fabriqua du corindon, du
diaspore, de la brucite, de l'analcime. Il obtint aussi des cristaux
qui n'existent pas dans la nature, comme ce très sensible
chloroaluminate de calcium (1897) qui se macle à tout propos, quand on
le touche ou quand on change sa température, et qui se démacle
au-dessus de 36^o^
quand on a dépassé son point de transformation paramorphique ; ou
encore comme cet aluminate de calcium (1903) qu'il a cité plusieurs
fois à l'appui de sa théorie des macles, à cause de ses beaux
assemblages par pseudomériédrie cubique laissant voir avec une
particulière évidence la symétrie simplement orthorhombique du cristal,
ou enfin comme ce silicate de lithium Si O3 Li2 qui est isomorphe de la
phénacite Si O4 Be2 et peut même cristalliser avec elle en donnant un
mixte, malgré la discordance des formules.

Ce remarquable tube à synthèses, je l'ai vu en 1909, à l'Ecole
des Mines de Saint-Etienne où je venais d'être nommé professeur.
Friedel était désireux de me faire participer à des études
minéralogiques. Il ressortit donc son tube où nous mîmes au hasard,
pour commencer, du pyroxène et une solution alcaline. Le tube nous
donna facilement d'abord quelques minéraux, notamment de la mésotype et
une chlorite très dispersive, ce qui était un bon début ; mais c'était
aussi la fin. Après les premiers essais le tube ne voulut plus rien
savoir. A chaque expérience nouvelle, quand nous déboulonnions, avec
beaucoup de curiosité et même un peu d'émotion, le disque de fermeture,
c'était pour être désappointés. Aucune réaction n'avait eu lieu parce
que l'eau était partie dès 1e commencement du chauffage par quelque
fissure imperceptible. Après quelques réparations qui ne réussirent
point il fallut abandonner l'instrument.

D'autres essais furent entrepris avec un tube semblable à
garniture de cuivre (celui qui avait servi à G. Friedel en 1891) mais
ils ne furent pas très heureux. Le cuivre est fortement attaqué, même
par les liqueurs alcalines, et on le trouve en inclusions dans les
cristaux de synthèse. Nous avons donc abandonné, avec regret, ce beau
champ de travail. Friedel devait le reprendre un peu plus tard, en
1912, pour la dernière fois. C'était pour obtenir la néphéline
potassique et montrer que l'excès de silice des néphélines naturelles
ne vient pas de leur mélange avec un silicate potassique plus siliceux.

Kntre 1896 et 1899 c'est la période des zéolithes. On
connaissait déjà, par les travaux de Damour et de Mallard, le singulier
comportement de l'eau zéolithique. Cette eau peut être chassée, puis
reprise, plusieurs fois de suite, et le cristal n'a pas perdu sa
cohésion ni sa transparence. Friedel établit pour l'analcime, la
chabasie et la mésotype des courbes de déshydratation dans une
atmosphère à tension de vapeur d'eau connue, il en déduisit la
véritable loi de l'équilibre. La tension de dissociation ne dépend pas
seulement de la température, mais aussi de la quantité d'eau qui reste
encore dans la zéolithe. Un cristal est toujours homogène, même s'il
est partiellement déshydraté. Il n'y a pas de combinaison ni de
proportion définie entre le silicate anhydre et l'eau.

Ce caractère additionnel de l'eau zéolithique, Friedel le rend
plus manifeste en nous montrant que beaucoup d'autres substances, comme
le gaz ammoniac, le gaz carbonique, l'hydrogène sulfuré, l'hydrogène,
l'alcool éthylique, peuvent la remplacer. Friedel a fait beaucoup de
mesures de ces absorptions. La chabasie absorbe 325 fois son volume de
gaz ammoniac et jusqu'à 2,7 % de son poids d'air. Tous les dosages
d'eau par perte de poids sont donc faux pour les zéolithes à moins que
l'on ait tenu compte de l'air absorbé dans l'exsiccateur, ou que l'on
ait chauffé jusqu'à fusion pour détruire « l'éponge » absorbante.

Les zéolithes sonl d'ailleurs loin de se comporter toutes de la
même manière. L'analcime est remarquable par son inertie au-dessous de
100° ; la mésotype par une sorte d'hystérésis de la déshydratation ; la
chabasie déshydratée par la vigueur avec laquelle elle absorbe. Dans ce
domaine Friedel a donc ajouté beaucoup à nos connaissances. L'eau
zéolithique n'est d'ailleurs pas spéciale aux zéolithes ni même aux
silicates. On la relrouve dans le chloroaluminate de calcium à macles
mécaniques réalisé en 1897.

Friedel fut peut-être le seul cristallographe qui ait vraiment
étudié la loi de Bravais. En général la loi fut condamnée ou négligée,
soit à cause de ses exceptions très évidentes, soit parce qu'elle a été
présentée par Bravais comme le résultat de spéculations sur les actions
mutuelles des molécules et que ces spéculations sont inacceptables
aujourd'hui. Friedel sépare donc la loi de celle base théorique,
inutile et fausse. Il l'accepte comme une loi d'observation possible,
précisant la loi d'Haüy, et il la confronte avec les faits.

S'il existe un réseau qui la vérifie, ce sera le réseau de Bravais,
peut-être identique au réseau-période et peut-être différent, mais
exprimant toujours une propriété remarquable de la matière cristallisée
puisqu'il suffira de connaître ce réseau pour en déduire les
importances relatives des faces, c'est-à-dire le faciès du cristal. Si
le réseau de Bravais existe il faut choisir les paramètres d'après lui,
afin que ces paramètres nous donnent le plus possible de renseignements
sur les formes. La loi de Bravais, considérée comme une loi
d'observation, nous fournit donc un critérium pour le choix de la forme
primitive et des paramètres. C'est même le seul critérium car la loi
d'Haüy est insuffisante. Comme les cristallographes n'ont tenu compte,
en général, que de la simplicité des indices, les réseaux qu'ils ont
choisis ne sont pas toujours conformes à la loi de Bravais. S'ils ne le
sont pas, on devra leur substituer un réseau conforme, multiple ou
sous-multiple du premier.

Tel est le point de vue dont Friedel est parti dès 1904. pour
recalculer des réseaux, classer leurs plans réticulaires par ordre de
densités décroissantes et comparer la liste obtenue à celle des
clivages et des formes. Il a fait dans cette voie un travail
considérable, notamment dans ses « Groupements cristallins » (1904) et
ses « Etudes sur la loi de Bravais » (1907). Dans les résultats il y a
beaucoup de bonnes vérifications tandis que d'autres, moins nombreuses,
ne sont qu'assez bonnes, ou médiocres, ou même mauvaises. Mais de
l'ensemble se dégage nettement que l'influence de la densité
réticulaire, sans être exclusive, est prépondérante. Il faut signaler
plus particulièrement, à ce sujet, les calculs de Friedel sur les
séries de faces de mêmes indices qui devraient être d'égales
importances si la simplicité des indices était seule en jeu, tandis
qu'au contraire leurs densités réticulaires les différencient en
proportion, ou du moins à peu près en proportion, de leur
développement. Il faut signaler aussi la curieuse loi des indices
moyens, établissant une relation de proportionnalité entre les
paramètres et les sommes des valeurs absolues des indices, de sorte
qu'elle permet de calculer les paramètres, et même les angles des axes,
si l'on connaît beaucoup d'indices. C'est une conséquence de la loi de
Bravais comme l'a démontré A. Liénard (1908). Je me rappelle avoir
demandé à Friedel comment il avait pu penser à une relation de ce genre
: « Quand on a manipulé plusieurs milliers de plans réticulaires avec
la constante préoccupation de voir comment varient leurs indices et
leurs densités à chaque changement des axes et des paramètres, c'est
une intuition toute naturelle », me répondit-il. Il y a beaucoup
d'exemples, dans l'histoire des sciences, de ce genre d'intuition a
posteriori, après de longs et patients calculs.

Plus tard, après les magnifiques résultats de l'étude
radiologique, Friedel revint sur la loi de Bravais. Ce fut pour
constater que le réseau de Bravais est différent du réseau-période
contrairement à ce qu'il avait admis comme très probable, mais non pas
comme certain, dans ses premiers travaux. Mais Friedel maintient, et je
crois que c'est très justement, que le réseau de Bravais reste le
meilleur pour le choix de la forme primitive et des paramètres, la
connaissance des structures nous laissant l'espoir de connaître un jour
sa signification véritable. Nous pourrons alors corriger l'énoncé
actuel, encore fruste et trop simple, de la loi de Bravais ; mais cette
loi restera fondamentale pour la cristallographie.

En même temps qu'il réhabilitait la loi de Bravais Friedel
entreprenait l'étude générale des macles. Il est même assez probable
que ce fut par les macles que Friedel commença ; mais pour que l'idée
qu'il dut s'en faire rapidement ait un sens précis et vérifiable il
fallait qu'il fût possible de distinguer, indépendamment du phénomène
de macle, le réseau simple d'avec ses multiples. C'est à quoi répondait
la seule loi de Bravais puisque l'on ne connaissait pas encore la
méthode radiographique.

Ce fut en 1904 que Friedel énonça sa loi générale sur le
prolongement ou le quasi-prolongement du réseau-période, à travers un
édifice maclé. Cette loi des macles est si simple, elle est si
naturellement la suite des interprétations que Bravais et Mallard
avaient déjà données pour les macles par mériédrie et par
pseudosymélrie, que l'on ne comprend pas bien pourquoi ces grands
esprits ne la conçurent pas dans toute sa généralité ; car il suffisait
d'assimiler les mailles multiples, pourvu qu'elles ne soient pas trop
grandes, à la maille simple et de leur attribuer les mêmes propriétés ;
et cette assimilation paraît bien naturelle puisque nous ne sommes pas
toujours capables de distinguer sûrement la maille la plus simple et
les autres. On pouvait encore être conduit à la loi des macles d'une
tout autre manière : en généralisant quelques observations d'Haüy sur
la staurotide et l'amphibole ; mais il semble bien que ces observations
si remarquablement intuitives du Père de la cristallographie aient
passé complètement inaperçues jusqu'en 1905 où Friedel attira
l'attention sur leur importance.

Friedel définit donc ses 4 classes de macles, les deux
dernières, celles par mériédrie et par pseudomériédrie réticulaires
étant nouvelles. Si la loi générale est vraie cela veut dire qu'une
macle quelconque peut être définie par une opération de symétrie de
réseau, c'est-à-dire, soit par une rotation de 180°, 120°, 90° ou 60°
autour d'une rangée importante, soit par une symétrie relativement à un
plan réticulaire dense, soit par une symétrie relativement à un point.
Les « Groupements cristallins » et beaucoup d'autres publications de
Friedel nous font bien voir en effet que toutes les macles peuvent se
définir ainsi. A l'exception d'un très petit nombre de cas douteux et
d'autres qui devinrent plus tard les macles monopériodiques, les macles
aberrantes n'existent pas. Friedel les a étudiées une à une. Il suffit
de changer leur définition pour les faire entrer dans le cadre de la
théorie générale, car une macle peut se définir de bien des manières.
Friedel nous donne à cette occasion la marche à suivre pour reconnaître
toutes les définitions possibles d'une macle, ce qui permet de choisir
celle qui est le plus exactement d'accord avec les bonnes mesures. Il
est remarquable que cette meilleure défmition corresponde à une
symétrie de réseau, ou bien, si plusieurs d'entre elles sont également
conformes aux mesures, qu'il y en ait toujours une qui corresponde à
une symétrie de réseau. On peut trouver un type de ce genre de travail
dans l'étude de 1922 sur la staurotide.

L'intervention nécessaire, dans de nombreux cas, de plusieurs
réseaux multiples pour expliquer les diverses macles d'un même minéral
est spécialement mise en lumière. C'est un point auquel Friedel tenait
beaucoup, parce qu'il rend impossible d'expliquer simultanément ces
macles par la pseudosymétrie du réseau simple, ou d'une « particule
cristallographique », ou plus généralement d'une figure quelconque. Les
pseudosymétries que semblent avoir tant de réseaux n'appartiennent
réellement qu'à certains de leurs multiples. Elles ne sont apparentes
que par les macles qu'elles rendent possibles. On peut même calculer
les probabilités d'existence des diverses macles et les résultats de ce
calcul sont assez bien confirmés par l'observation. Friedel explique
ainsi, entre autres choses, pourquoi tant de cristaux présentent des
macles à 60° malgré que leurs réseaux simples n'aient rien de
pseudosénaire.

Quant à la surface d'accolement, il est clair qu'elle peut être
quelconque dans les macles par mérièdrie et par mérièdrie réticulaire.
Mais dans les deux autres classes de macles elle est déterminée par le
quasi-prolongemeni du réseau simple ou multiple. On démontre sans peine
que, s'il y a un axe binaire de macle, l'accolement doit se faire
suivant la section rhombique. Friedel découvre donc la raison d'être de
cette direction non réticulaire, déjà connue dans les feldspaths et
quelques autres minéraux, mais dont aucune explication correcte n'avait
été donnée.

Cette belle théorie s'étend d'elle-même à l'orientation
mutuelle d'espèces différentes, à la syncristallisation, à
l'isomorphisme, au paramorphisme. Il suffît qu'il y ait une concordance
approchée, en forme et en dimensions, entre les périodes, ou entre des
multiples simples des périodes. Il suffit même, pour certains cas
d'orientation, comme l'a montré Royer, que la concordance existe dans
la surface d'accolement. On pouvait prévoir par ces derniers
phénomènes, si remarquables, que la théorie des macles allait être
attirée vers une nouvelle généralisation.

Ce que fut cette généralisation, nous le savons par le dernier
et beau travail de Friedel, écrit en juin 1933, six mois avant sa mort
et paru dans notre bulletin. Il y est question de la macle de Zinnwald
que J. Drugman venait de retrouver en abondance dans les quartz
bipyramidés des Cornouailles. La macle de Zinnwald se refuse à entrer
dans le cadre ordinaire de la théorie. Il faut faire intervenir, pour
l'expliquer, un principe nouveau qui est la possibilité d'orientation
entre deux cristaux préexistants d'un même minéral lorsqu'ils se
touchent pendant leur croissance alors que l'un d'eux, au moins, est
libre et de petite taille (Gaubert, Drugman, Schaskolsky et
Schubnikow). Des exemples très peu nombreux, mais incontestables,
montrent qu'il suffit pour cela qu'une rangée, c'est-à-dire une période
à une dimension, soit commune.

Friedel ne pouvait qu'être satisfait de ce genre d'exception à
sa théorie puisqu'il la confirme en étendant son principe de
concordance à des cas nouveaux. Presque toutes les macles connues sont
tripériodiques, c'est-à-dire qu'elles ont 3 périodes linéaires dans 3
directions de l'espace. Il existe aussi, probablement, des macles
dipériodiques mais elle n'ont pas encore été rencontrées. Les macles
monopériodiques sont celle de Zinnwald pour le quartz et la macle de
l'alun réalisée par M. Schaskolsky et A. Schubnikow.

Nous commençâmes en 1909, Friedel et moi, à observer les
liquides anisotropes de 0. Lehmann. Ils étaient alors très généralement
désignés sous le nom de « cristaux liquides » et Lehmann avait décrit
abondamment leurs propriétés ; mais de ses descriptions, mêlées à
toutes sortes de considérations étrangères, ne se dégageait aucune idée
précise, du moins pour nous. Nos premières observations se rapportèrent
aux « flüssige Kristalle » qui sont aujourd'hui les corps nématiques.
Nous décrivîmes leurs « plages à noyaux » et aussi leurs fils qui ne
sont pas des impuretés comme Lehmann le croyait alors ; mais dans ce
premier travail nous ne réussîmes pas à interprêter justement les
singuliers phénomènes que nous observions. Comment admettre qu'un
liquide qui semble agité fortement est néanmoins capable de s'orienter
sur ses pellicules superficielles et de garder à tout instant, en
chaque point, malgré son agitation, un axe optique invariable? Cela
nous semblait impossible. Bien que nous ayons fait connaître aux
minéralogistes (je le crois du moins), à l'occasion de ce travail, la
représentation de Poincaré, nous n'avions pas songé à construire le «
le cône de Mauguin » pour expliquer les plages tordues. Ce fut donc
Mauguin qui établit la structure hélicoïdale du liquide entre les deux
pellicules superficielles dont il raccorde les orientations.

L'année suivante nous découvrîmes les surprenantes coniques
focales dans les « fliessende Kristalle » c'est-à-dire dans les corps
smectiques. Dans le domaine focal l'axe optique est orienté partout
suivant une droite qui va d'une ellipse à une branche d'hyperbole, les
deux coniques étant liées l'une à l'autre par la relation de focalité.
L'ellipse et l'hyperbole sont des lignes de discontinuité en chaque
point desquelles il y a une infinité d'axes optiques formant un cône de
révolution. Les domaines focaux, qui ne se compénètrent jamais, se
groupent de diverses manières ; ils peuvent avoir leurs ellipses
tangentes entre elles et leurs hyperboles concourantes ; ils peuvent
raccorder des plages homogènes ; ils peuvent se fragmenter et
s'excentrer dans des espaces de faible épaisseur à surface externe un
peu convexe. L'observation de certains mélanges dans lesquels la
cristallisation conserve la trace de la structure à coniques conduit à
penser que les molécules très allongées des corps smectiques sont
disposées dans le domaine focal suivant des couches d'égale épaisseur,
toutes incurvées suivant une même famille de cyclides de Dupin
parallèles dont les focales sont l'ellipse et l'hyperbole. A
l'extérieur des domaines focaux, car ceux-ci ne peuvent occuper tout
l'espace, les surfaces perpendiculaires en chaque point à l'axe optique
ne sont plus des cyclides de Dupin mais d'autres surfaces qui les
prolongent.

A la fin de 1911 ma nomination à Paris interrompit brusquement
notre travail. J'ai conservé de cette collaboration trop brève, où j'ai
peu donné et beaucoup reçu, un souvenir très reconnaissant à celui qui
fut mon initiateur aux recherches personnelles. Je n'ai jamais retrouvé
dans la suite, lorsque j'ai pu reprendre seul, à de longs intervalles,
ces travaux de ma jeunesse, le plaisir que j'eus alors, aux côtés de
Georges Friedel, à Saint-Étienne, dans le très, modeste laboratoire de
l'ancienne Ecole des Mines.

Ce fut avec L. Royer, en 1921, que Friedel se remit aux liquides
anisotropes. Leur nombre avait beaucoup augmenté, grâce aux chimistes
allemands, de sorte que l'on pouvait mieux voir leurs caractères
généraux. Friedel fit donc une révision de tous ceux qu'il put se
procurer et il s'en occupa jusqu'à la fin de sa vie, tantôt avec Royer,
tantôt seul, tantôt avec son fils Edmond. Ces corps très singuliers,
Lehmann en diminuait sans raison l'importance en les appelant cristaux
liquides. Ce ne sont pas des cristaux, ni toujours des liquides, mais
deux nouveaux arrangements de la matière, que Friedel appelle des
stases. Elles sont aussi importantes et aussi dignes d'étude que les
deux autres stases qui sont connues depuis beaucoup plus longtemps, la
stase cristallisée et la stase amorphe. Les deux stases nouvelles sont
appelées mésomorphes. Ce sont la smectique où une direction unique de
surfaces, toujours perpendiculaires à l'axe optique, jouit en chaque
point de propriétés vectorielles discontinues, et la nématique où rien
n'est discontinu en un point quelconque, mais qui diffère de la stase
amorphe par l'anisotropie spontanée. Les 4 stases de la matière se
suivent toujours dans le même ordre, quand la température s'élève,
d'abord la cristalline puis la smectique, puis la nématique, enfin
l'amorphe. Les 4 stases sont séparées par des discontinuités absolues.

Pour les corps smectiques Friedel assimile les gradins des
gouttes planes aux pellicules de savon étudiées par J. Perrin et P. V.
Wells. Leurs molécules sont distribuées au hasard mais en couches
parallèles d'épaisseur uniforme, sur des surfaces équidistantes bien
définies. Une de leurs directions, probablement leur allongement, est
perpendiculaire à ces surfaces. Cette direction privilégiée, c'est
l'axe optique, qui est toujours unique et de plus grand indice. Les
coniques focales sont les seules discontinuités de la structure.
Friedel suggère l'emploi des rayons X pour établir définitivement la
distribution spatiale par couches équidistantes et pour mesurer
l'équidistance. Il eut le bonheur de voir réaliser cette belle
vérification par son fils en 1923, avec de Broglie, et de la voir
confirmer dans un autre travail d'Edmond Friedel en 1925.

Pour les corps nématiques Friedel distingue nettement les
nématiques proprement dits, de signe positif, qui sont nos anciens
liquides à fils ou à noyaux, et les corps cholestériques, de signe
apparemment négatif. L'absence de diffraction des rayons X montre bien
que la distribution spatiale des molécules est quelconque, dans l'un
comme dans l'autre groupe. Le grand mérite de Friedel et de Royer est
ici d'avoir reconnu le véritable caractère des corps cholestériques en
les rapprochant étroitement des corps nématiques proprement dits,
malgré des différences considérables qui semblaient les apparenter
beaucoup plus, au premier abord, aux corps smectiques ; car les corps
cholestériques montrent aussi des plans équidistants et ces plans
peuvent aussi s'incurver en cyclides de Dupin, lesquelles sont alors
accompagnées, nécessairement, comme dans les corps smectiques, de
coniques focales.

On sait que les corps cholestériques, dans la structure à
plans, ont des pouvoirs rotatoires énormes et qu'ils réfléchissent des
couleurs magnifiques. On sait aussi que ces couleurs, dites
épipoliques, sont toujours polarisées circulairement, l'une des
vibrations circulaires paraissant se réfléchir sélectivement sur les
plans tandis que l'autre n'est pas affectée. Friedel et Royer ont
montré que la vibration circulaire qui est réfléchie n'a pas changé de
sens, ce qui est contraire aux lois habituelles de la réflexion, et que
sa longueur d'onde paraît être identique à la longueur d'onde
d'inversion de Stumpf.

En mélangeant d'autre part, en proportion convenable, du
cyanobenzalaminocinnamate d'amyle (dextre) et du benzoate de
cholestérine (senestre) Friedel et Royer ont réussi à observer à une
température TETA un corps cholestérique qui n'est ni dextre ni
sénestre. Ce corps cholestérique dépourvu de pouvoir rotatoire est
également dépourvu de plans équidistants et de couleurs épipoliques, de
sorte qu'il est identique à un corps nématique proprement dit, de signe
positif. En faisant varier la température, de part et d'autre de TETA,
le liquide devenait droit ou gauche et toutes les propriétés de la
structure à plans réapparaissaient. Ainsi dans cette observation
capitale le passage du type nématique proprement dit au type
cholestérique est continu. Il paraît se faire par torsion autour d'une
normale à l'axe optique positif du liquide. Cette torsion spontanée
détermine le pouvoir rotatoire et l'uniaxie négative apparente. Les
corps cholestériques sont des corps nématiques qui sont tordus à cause
d'une dissymetrie holoaxe de leur molécule. La torsion est en général
très grande. Elle est accompagnée, on ne sait pourquoi ni comment, de
discontinuités équidistantes qui jouent le même rôle que les plans
moléculaires des corps smectiques et qui entraînent les mêmes textures
; mais ces discontinuités, dans les corps cholestériques, sont très
largement espacées, par exemple de 2.000 à 10.000 Â ou davantage tandis
qu'elles sont de quelques dizaines d'Â dans les corps smectiques,
c'est-à-dire unimoléculaires, très probablement.

La torsion spontanée des corps cholestériques n'est qu'une
hypothèse, mais elle explique bien des choses. Friedel a soin de nous
faire remarquer qu'elle ne rend pas compte de tout. Nous ne voyons pas,
en particulier, pourquoi les couleurs épipoliques et le pouvoir
rotatoire disparaissent entièrement dans la texture à coniques.

A ces remarquables observations il faudrait en ajouter beaucoup
d'autres. Par exemple, pour un mélange de corps nématique sans pouvoir
rotatoire avec une petite quantités de corps cholestérique rotatoire,
la surprenante augmentation du pouvoir rotatoire quand la quantité
diminue ; ou l'apparition de propriétés cholestériques dans des
mélanges de corps nématiques proprement dits avec de la colophane, qui
n'est pas mésomorphe, mais qui est douée de pouvoir rotatoire
moléculaire.

Ainsi nous avons maintenant, grâce aux travaux de Friedel et de
ses collaborateurs, une vue d'ensemble sur les corps mésomorphes, et
une classification de la matière en 4 stases très distinctes et très
bien caractérisées par leurs structures. Friedel insiste beaucoup pour
que l'on différencie profondément ces stases, toujours séparées par des
discontinuités absolues, et les 3 états de la matière, le solide, le
liquide et le gazeux, qui peuvent passer de l'un à l'autre par voie
continue. Il faut donc distinguer l'amorphisation d'avec la fusion. Le
quartz, par exemple, s'amorphise à 1.200° par transformation
discontinue, en perdant brusquement sa structure réticulaire. Il ne
devient liquide qu'à une température plus élevée et mal définie,
graduellement, sans aucun changement nouveau de structure.

La croissance et la décroissance des cristaux est un des sujets
importants de l'oeuvre de Friedel. Un mémoire de 1913 attire
l'attention, à la suite de A. Berthoud, sur l'insuffisance de la
théorie de P. Curie. Rien n'autorise à admettre qu'un cristal solide, à
une température donnée et en présence de sa solution saturée puisse
être dissous dans certaines directions et accru dans d'autres de
manière à garder un volume constant, jusqu'à ce qu'une certaine forme
soit réalisée. Les prémisses de Curie ne sont pas acceptables. Quant à
la loi de Wulff sur les vitesses normales de croissance, si on
l'applique à un cristal de forme quelconque, comme le fait Wulff, on
est en contradiction avec l'hypothèse de Curie dont la loi de Wulff est
cependant déduite. Mais la contradiction n'existe pas pour un liquide.
En faisant le calcul dans ce dernier cas, Friedel arrive à ce résultat
que le liquide doit prendre, s'il est entouré par un fluide isotrope,
la forme définie par la loi de Wulff. Si donc ses tensions capillaires
variaient d'une manière discontinue avec la direction il serait limité
par des faces planes comme un cristal. Les « cristaux liquides » ne
montrant rien de semblable, contrairement à l'affirmation de Lehmann,
il faut en conclure que les tensions capillaires y sont vectorielles
continues (sauf peut-être pour une seule direction de plan, en chaque
point, dans le cas des corps smectiques) et que ce ne sont pas des
cristaux. Plus tard, en 1924-1926, Friedel abandonne complètement la
considération théorique des constantes capillaires pour ne plus
s'occuper que des vitesses de croissance et de décroissance lesquelles
ont le grand avantage d'être accessibles à l'observation. Il reprend
une formule de Berthoud sur ces vitesses et il généralise et
perfectionne une théorie de la diffusion due à Berthoud et à Valeton.
On peut ainsi prévoir et justifier les surfaces courbes de croissance.
Il n'y a aucune dissymétrie physique entre la croissance et la
décroissance des cristaux, mais seulement une dissymétrie géométrique
apparemment très accentuée, mais disparaissant presque si l'on compare,
comme on le doit, la croissance en forme convexe à la décroissance en
forme concave. Friedel a fait beaucoup d'observations sur les cristaux
négatifs obtenus en agrandissant par dissolution une cavité de forme
quelconque creusée au préalable dans un cristal. Il montre que ces
cavités se limitent par les mêmes faces lentes qu'un cristal convexe en
voie d'accroissement. La théorie permet de prévoir aussi « l'effet
d'angle » qui introduit une dissymétrie entre la croissance convexe et
la décroissance concave et surtout entre la croissance concave et la
décroissance convexe. Friedel a appliqué très heureusement cet effet
d'angle et plus généralement les règles de la croissance et de la
décroissance des cristaux aux figures de croissance et de corrosion. Il
aborde même une théorie plus générale tenant compte de la chaleur de
cristallisation.

Bien qu'il n'ait pas établi lui-même des radiogrammes, Friedel
s'est beaucoup intéressé à la diffraction des rayons X et nous avons de
lui plusieurs travaux théoriques sur cette question. L'une de ses
remarques essentielles est relative à la longueur moyenne d'un train
d'onde. Il faut faire intervenir dans les calculs cette longueur
moyenne qui n'est pas connue, mais qui est certainement très petite, au
lieu d'admettre, ce qui ne peut être vrai, que toute la partie du
cristal intéressée par le rayonnement est capable d'interférence
régulière. On explique ainsi les lois empiriques de Bragg sur
l'intensité des rayons diffractés. La série dite normale en l/sin^2^
THETA ne donne les intensités des ordres successifs que dans le
dispositif de Bragg ou pour une partie des plans réflecteurs dans la
méthode du cristal tournant. On a tort de l'appliquer à d'autres
dispositifs.

Friedel n'a pas accepté l'interprétation ordinaire pour les
radiogrammes des cristaux mixtes. De ce que ces cristaux donnent des
radiogrammes aussi simples que ceux des corps purs et correspondant à
des paramètres bien définis calculables par une loi de moyenne, ne
résulte pas qu'il y ait dans un cristal mixte un réseau parfaitement
périodique à paramètres constants. C'est le phénomène optique de
diffraction qui réalise la période moyenne, et non pas le cristal
mixte. Friedel applique cette conclusion aux édifices pseudoparamorphes
et il en montre l'importance. Pour le quartz, par exemple, la structure
à laquelle sont arrivés les auteurs est probablement inexacte. On sait
d'ailleurs qu'elle s'accorde mal avec les intensités. Il en est
probablement ainsi pour tous les cristaux mimétiques lorsque les
orientalions coexistantes sont très finement mélangées. La diffraction
des rayons X devient alors impuissante à nous révéler la vraie
distribution des atomes. Il faudrait, pour progresser dans cette voie,
savoir mesurer plus exactement les intensités et savoir aussi les
calculer plus sûrement en fonction de la structure.

Friedel n'a pas recherché les espèces nouvelles. Je crois même
qu'il n'en a découvert aucune qui le soit véritablement, à l'exception
de quelques cristaux de synthèse. Il a beaucoup observé cependant, mais
surtout pour vérifier sa théorie des macles ou la loi de Bravais. Nous
le voyons par exemple, dans sa belle étude sur la boléite et ses
congénères, élucider parfaitement les relations complexes entre ces 3
minéraux quadratiques. La boléite seule se macle. C'est « le plus
magnifique exemple de mimétisme cubique dû à une macle et sans rapport
aucun avec la pseudosymétrie cubique », car son réseau octaédral a une
maille exceptionnellement allongée. Les groupements trirectangulaires
de cumengéite, qui seraient des exceptions à la loi des macles s'ils
existaient seuls, ont toujours un noyau de boléite maclée et de
pseudoboléite sur lequel ils sont régulièrement orientés.

D'autres études ne se rattachent pas directement aux grands
sujets théoriques. Friedel a décrit la singulière mélanophlogite dont
il avait recueilli de beaux cristaux pendant un voyage en Sicile ;
l'anthophyllite de Saint-Germain-l'Herm où il remarque un clivage
cylindrique ou linéaire qu'il compare aux clivages fibreux et vitreux
du gypse ; les cristaux difformes de sel gemme que l'on trouve dans les
marnes salifères du gisement potassique d'Alsace. Il s'est occupé
beaucoup du diamant, d'abord pour en décrire les formes et discuter la
question de l'anti-hémièdrie, puis pour montrer, par des observations
très minutieuses, que la biréfringence que possèdent tous les diamants
n'est pas due au mimétisme, contrairement à ce qu'en pensait Mallard,
mais à des tensions intérieures. Cette biréfringence, très
irrégulièrement répartie, suit exactement les mêmes lois que dans les
corps isotropes qui sont devenus biréfringents par déformation ;
c'est-à-dire qu'elle se manifeste par deux sortes de points fixes
dépourvus de biréfringence autour de chacun desquels les directions
principales sont disposées d'une manière constante et particulière. La
distribution serrée des points fixes prouve que la déformation du
diamant a été extrêmement brusque de sorte qu'il n'en faut pas chercher
la cause dans les conditions extérieures mais au contraire dans le
diamant lui-même. Friedel est conduit de cette manière à supposer
qu'elle accompagne une transformation paramorphique à haute
température, puis à réaliser cette transformation, en collaboration
avec G. Ribaud, grâce à un four électrique perfectionné permettant
d'atteindre en quelques minutes une température de 2000°. Il faut aller
très vite afin que le diamant ne se transforme pas entièrement en
graphite pendant la durée d'un essai. Le changement brusque se fait à
1885° sous la pression atmosphérique. Au-dessus, le diamant BETA est
cubique, peut-être antihémièdre. Au-dessous, le diamant ALPHA est
également cubique, mais holoèdre. Tous les diamants ont traversé le
point de transformation.

Friedel conçoit donc la genèse du diamant à très grande
profondeur, dans le noyau métallique terrestre, sous la iorme BETA,
puis son refroidissement par ascension vers la surface jusqu'à une
température inférieure au point de transformation paramorphique et
assez basse pour que le diamant ALPHA ne puisse plus subir la
graphitisation ni l'action oxydante des silicates qui l'enroberont par
la suite.

Par l'exposé que je viens de faire, bien qu'il soit très
imparfait, on peut juger de l'activité du savant et de l'importance de
son oeuvre, mais on s'en ferait une idée encore incomplète, car Friedel
ne fut pas exclusivement cristallographe ou minéralogiste. Il préféra
certainement son laboratoire et c'est là qu'il fit presque tous ses
travaux ; mais il ne dédaigna pas d'étudier sur le terrain, de sorte
qu'il fut aussi pétrographe et géologue. Dans ce domaine comme dans les
autres, bien qu'il ne fut le sien qu'assez peu souvent et par occasion,
Friedel montra ses grandes qualités d'observateur. En 1897 il décrivit
avec détail les sources minérales de Pougues qu'il avait eues dans son
service à Moulins. Ensuite vint le granite de Firminy (1902) qui devait
avoir une grande fortune géologique. Friedel y parle d'une roche
singulière qu'il prend d'abord pour une arkose, tapissant au Sud Ouest
le fond du bassin houiller de Saint-Étienne, et reposant sur les
micaschistes. Mais en 1906, en collaboration avec Pierre Termier, la
vraie nature de la roche est reconnue : ce n'est pas une arkose, c'est
un granite écrasé complètement, une mylonite; elle témoigne d'un
charriage qui a précédé le dépôt du houiller ; avec le granite de
Firminy elle est le reste d'une nappe dont on retrouve d'autres
lambeaux, recouvrant d'autres nappes plus profondes.

On sait que cette question des nappes antestéphaniennes se
développa dans la suite et que plusieurs géologues y collaborèrent.
Friedel a fait pour sa part, dans la région de Saint-Étienne, un grand
nombre de courses géologiques et j'ai eu le plaisir de l'accompagner
souvent. Il y était tout à fait naturaliste, attentif au détail comme à
l'ensemble, et saisissant avec une clarté qui m'émerveillait, dans
cette difficile question, les rapports des très nombreuses roches
cristallines et les variations de leurs faciès.

Nous avons encore de Friedel, avec Pierre Termier, une étude
sur le bassin houiller du Gard et sur les klippes d'urgonien de la
plaine d'Alès. Depuis Marcel Bertrand c'est une terre classique des
tectoniciens. L'étude détaillée des klippes confirme bien qu'ils sont
des restes d'une ancienne nappe urgonienne, étendue par charriage sur
l'oligocène. Le bassin houiller du Gard, avec ses chevauchements
compliqués de terrains plus jeunes par des terrains plus anciens, doit
bien sa structure à un charriage d'âge alpin allant du Sud-Est au
Nord-Ouest et se heurtant au grand massif cristallin du Plateau
Central.

Il faut encore ajouter, au travail du savant, celui du
professeur et du directeur. Friedel s'intéressait beaucoup à
l'enseignement. En 1905, en compagnie de deux de ses collègues, MM. A.
Liénard et R. Etienne, il visita plusieurs écoles d'ingénieurs à
l'étranger afin de les comparer à l'Ecole des Mines de Saint-Étienne et
de rechercher des améliorations pour celle-ci. L'organisation des cours
et leurs programmes ont toujours tenu beaucoup de place dans les
préoccupations de Friedel. En 1931, il intervint dans une discussion
publiée dans « l'Enseignement scientifique » sur le régime des concours
et des examens. Il a rédigé ses leçons à plusieurs reprises. Nous avons
trois éditions de son cours à l'École des Mines de Saint-Ëtienne, avant
les admirables « Leçons de cristallographie professées à la Faculté des
Sciences de Strasbourg » (1926). Combien la lecture de ces « Leçons »
si pleines et si personnelles fait regretter l'absence du 2e volume !
Friedel avait l'intention de l'écrire mais la maladie l'en empêcha.
Friedel fut avant tout un esprit clair, un logicien rigoureux, ennemi
de toute confusion, surtout de celle qui se dissimule derrière des mots
imprécis ou des définitions incomplètes. Il eut une prédilection pour
les principes fondamentaux de la cristallographie. Constamment il y
revint, comme aussi sur beaucoup d'autres propriétés générales, afin
d'en dégager plus nettement, mais en partant de la seule observation,
les vrais caractères. Il est bon, dit-il quelque part, de retourner les
questions de toutes les manières. C'est bien ce qu'il a fait pour la
définition du cristal, la loi d'Haüy, celle de Bravais, l'homogénéité,
l'hypothèse réticulaire. Il a précisé toutes ces notions beaucoup mieux
qu'on ne l'avait fait avant lui. En les précisant il rencontra
certaines propriétés intéressantes, comme celles des axes ternaires
irrationnels qui sont compatibles avec la loi d'Haùy mais qui ne le
sont pas avec la structure réticulaire. Il eut toujours le souci de
délimiter strictement les hypothèses et de rejeter toutes celles qui ne
sont pas indispensables. La molécule cristallographique, par exemple,
est une hypothèse qui est non seulement inutile, mais fâcheuse, car
elle empêcha Mallard, entre autres choses, de généraliser ses
macles par pseudosymétrie. Pour les molécules qui se groupent en un
cristal, Friedel veut que l'on ne fasse à leur sujet aucune hypothèse.
C'est déjà en faire une, dit-il en 1904, «que d'admettre qu'elles y
restent distinctes, de façon que dans un cristal Na Cl par exemple un
atome Na et l'atome Cl correspondant forment un petit groupe bien
sépare des autres, et qu'il n'y a aucun doute quant à l'attribution de
tel atome de Na à tel atome de Cl plutôt qu'au voisin ». Le mieux est
donc d'appeler d'un mot nouveau, le motif, ce remplissage de la maille
afin de traduire exactement la seule propriété qu'on lui connaisse, qui
est de pouvoir remplir périodiquement tout l'espace par des
translations de grandeurs fixes.

Le choix des mots, surtout de ceux qui doivent exprimer les
idées fondamentales, avait donc pour Friedel une très grande
importance. Personne ne fut convaincu plus que lui du rôle malfaisant
des expressions mal choisies ou à double sens. Aussi s'attache-t-il
partout dans son oeuvre à bien définir. Il distingue les textures et
les structures, les stases et les états de la matière. La netteté du
langage lui est nécessaire. C'est pour cela que ses publications sont
si faciles à lire. Elles ne laissent jamais aucune indécision dans
l'esprit.

A la rigueur et à la clarté Friedel ajouta bien d'autres
mérites. II fut très cohérent et très complet, capable, ce qui est
exceptionnel, d'envisager simultanément toutes les faces d'un problème
et tous les faits qui s'y rattachent; car il ne fut pas un spécialiste.
Il embrassa vraiment toute la cristallographie. Il en explora lui-même
tous les grands domaines expérimentalement et par la pensée, jusqu'à ce
qu'il pût s'en faire une opinion personnelle. Très ouvert aux nouvelles
méthodes, c'est-à-dire à l'étude de la diffraction des rayons X, il ne
pensa jamais qu'elles aient fait perdre leur importance aux anciennes
observations. Friedel était surpris de voir avec quelle facilité
certains cristallographes, et surtout les physiciens qui n'envisagent
souvent les cristaux qu'au seul point de vue radiographique, ayant
adopté avec enthousiasme les nouvelles méthodes, ce qui est très
justifié, en arrivent à tenir les autres propriétés pour
négligeables.Pour la boléite, par exemple, il est singulier que des
contradicteurs, concluant faussement à la symétrie cubique de ce
minéral, ne tiennent plus compte de son clivage octaédrique, si net
cependant et capable de donner le paramètre avec autant de précision
que les meilleurs radiogrammes.

Il faut qu'une théorie nouvelle s'accorde exactement avec les
faits anciens. Si l'accord n'est pas bon et s'il s'agit, bien entendu,
de faits certains et vérifiés, il faut le dire et proclamer
l'imperfection de la théorie. Supprimer les faits contraires ou
contester leur importance n'est pas scientifique, car les théories
évoluent et se transforment profondément, mais les faits demeurent. Le
seul intérêt d'une théorie physique est d'établir un lien entre des
faits observables. Friedel aimait les théories et les idées générales,
il fut théoricien autant qu'observateur, mais il n'avait confiance que
dans les faits.

Georges Friedel l'ut un savant très original, une forte
personnalité. Vivant retiré dans sa famille, à Saint-Etienne ou à
Strasbourg, en relation avec quelques amis excellents et peu nombreux,
fuyant les honneurs, il fut aussi indépendant qu'il est possible de
l'être. Aussi exprima-t-il toujours sa pensée très simplement, sans
aucun détour, parfois même avec un peu de rudesse. Il avait le goût de
la controverse et la tenait pour féconde. Son esprit vigoureux
n'admettait pas qu'on fût neutre. C'est qu'il aimait passionnément sa
science. A travers les hommes il ne vit jamais qu'elle et ses progrès
magnifiques, car il vécut une grande époque de la cristallographie. Que
de découvertes, dans des directions si différentes, depuis l'année où
il s'asseyait sur les bancs de l'Ecole des Mines, écoutant Mallard !
Combien la science des cristaux est aujourd'hui plus riche et plus
solidement bâtie ! Lorsque Friedel, dans sa chambre de Strasbourg, le
corps malade et douloureux, mais la pensée aussi ardente qu'au temps de
sa jeunesse et aussi attentive aux progrès de la cristallographie
qu'elle le fut jamais, contemplait ce bel édifice, il ne pouvait pas ne
pas voir de quelle importance y était son oeuvre personnelle. Ce fut sa
très noble récompense, la meilleure que puisse avoir le savant.

F. GRANDJEAN.
