# Supplementary material for: Space groups and crystallographic symmetry: writing a multi-featured tutorial in a new style
Source: Acta Crystallogr E Crystallogr Commun. 2021 Jul 16;77(Pt 9):857–63. doi: 10.1107/S2056989021007039 (PMC8423017; doi:10.1107/S2056989021007039)
Supplement: Supplementary file 1 [file e-77-00857-sup2.zip › symandsg/Main/guinier.html]

(IUCr) André Guinier (1911-2000)

<body>
Because your browser cannot handle the frames used to structure this article, you must <a href="es0293bdy.html">CLICK HERE</a>
</body>
