# Supplementary material for: Space groups and crystallographic symmetry: writing a multi-featured tutorial in a new style
Source: Acta Crystallogr E Crystallogr Commun. 2021 Jul 16;77(Pt 9):857–63. doi: 10.1107/S2056989021007039 (PMC8423017; doi:10.1107/S2056989021007039)
Supplement: Supplementary file 1 [file e-77-00857-sup2.zip › symandsg/Main/guinier_files/es0293bdy.htm]

(IUCr) André Guinier (1911-2000)

## obituaries

---

|  |  |
| --- | --- |
| **Volume 57**   **Part 1**    Pages 1-3    January 2001     © International Union of Crystallography 2001 | André Guinier (1911-2000)  **Marianne Lambert^a^^\*^**  ^**a**^Laboratoire de Physique des Solides, Bâtiment 510, Université Paris-Sud, 91405 Orsay, France Correspondence e-mail: lambert@lps.u-psud.fr   **Keywords: obituary.** |

André
Guinier died on 3 July 2000, one month short of his 89th birthday. He
had an exceptionally long and brilliant career as a solid-state
physicist and crystallographer; passionately fond of scientific
research, he still attended scientific meetings and laboratory seminars
until a year before his death.

|  |  |
| --- | --- |
|  | **Figure 1** |

When a young man, he was a very gifted student and succeeded in
entering the illustrious Ecole Normale Supérieure (ENS). He graduated
in 1934 and became Agrégé Préparateur in the Physics Laboratory of the
ENS in 1935. Such a privileged position gave him the opportunity to
begin research work, with a free choice both of the scientific field
and of his thesis supervisor. He chose the respected crystallographer
Charles Mauguin. André Guinier presented his thesis in 1939 [*La
Diffraction des Rayons X aux Très Faibles Angles: Applications à
l'Etude des Phénomènes Ultra-microscopiques. Ann. Phys. (Paris)*, (1939), **12**
161-236] and obtained a position at the Conservatoire National des Arts
et Métiers (CNAM), where he became deputy-director of the Testing
Laboratory (Laboratoire d'Essais) in 1944. The CNAM is situated in a
historic building in the centre of Paris; André Guinier nevertheless
managed to create a Research Laboratory there where he received his
first students. In 1949, he became a Professor at the University of
Paris (the Sorbonne). He was then giving two types of course in
parallel: basic physics at the University, and X-rays and the structure
of metals at the CNAM. From very early on, his inventive and
stimulating research work attracted foreign researchers and
post-doctoral students to his group.

Ten years later, a
University Scientific Centre was created at Orsay, outside Paris, as an
extension of Paris University. André Guinier was the first Dean of
Orsay and spent much energy and dynamism to successfully build up this
new campus, which rapidly became one of the world's major scientific
centres. He transferred his Research Laboratory to Orsay, and together
with Jacques Friedel and Raimond Castaing founded the Solid State
Physics Laboratory (Laboratoire de Physique des Solides, LPS). When
this university laboratory later became associated to the CNRS (Centre
National de la Recherche Scientifique), André Guinier was its first
Director.

Professor at Orsay University and Member of the French
Academy of Sciences, André Guinier was a very eminent scientist: his
work is known and praised all over the world and he has been awarded
numerous prizes from foreign as well as French institutions. Related to
both his deep understanding of order and disorder phenomena in solids
and his faculty to plan and carry out the vital experiment, his
contributions were fundamental in a number of fields concerning
condensed matter. Most of his work concerned the use of X-ray
scattering to investigate and define the extent of order in solids: he
pointed out the significance of diffuse scattering arising outside the
Bragg selective diffraction spots and the importance of `local order'
to explain some physical properties of materials. A variety of
experiments were used by him, particularly small-angle scattering and
wide-angle diffuse scattering, which, together with their analysis, led
to the development of what has come to be known as the `Guinier school'
of X-ray scattering.

In the case of small-angle scattering, the
diffuse scattering is located close to the direct beam and is given by
particles with an electron density different from that of the
surrounding medium. André Guinier's very early observations made in
1938 on the Al-Cu alloy led to the discovery of the Guinier-Preston
zones, which are specific to pre-precipitation phenomena in alloys, of
major importance for hardening in alloys. The spatial extent of the
diffuse scattering is directly connected to the size of the particles
(the smaller the particles, the larger the extent) and the approximate
relationship for the intensity variation is known as the Guinier law.
Such kinds of studies were performed later on by André Guinier and his
students for many different systems: metallurgical solid solutions,
defects and radiation damage in crystals, biological solutions,
polymers *etc*.

The study of
wide-angle diffuse scattering corresponds to a more difficult situation
since it involves, in principle, the whole reciprocal-space
description. In the simplest cases, it is relatively easy to explain
experimental observations by some particular distribution of atoms in
the material: position correlation of atoms in disordered solid
solutions, displacement correlations of atomic motions close to phase
transitions. In fact, owing to the progress of instrumentation and
analysis resources, more complex systems are now investigated:
low-dimensional organic and inorganic conductors, fullerene compounds *etc*.
The careful modeling of the intricate diffraction patterns gives
information on the microscopic phenomena at play, the dimensionality
and strength of local interactions. In most crystalline materials, the
global intensity of diffuse scattering is much lower than that of the
diffraction spots related to the mean lattice. This is not the case for
liquid-crystalline materials: taking into account both kinds of
scattering, the new investigative tools, built up for the description
of structural fluctuations related to phase transitions, are basic for
the understanding of these peculiar `structures'.

A natural
progression takes one from the study of metallic solid solutions to
amorphous metals. At the beginning of the 70's, new metallic alloys
were prepared at Caltech by very rapid quenching of the melt: this
technique was then introduced in the Orsay laboratory, opening up new
studies of metallic metastable phases. It was the beginning of a new
field of structural investigations on glasses, liquids, and amorphous
semiconductors, where André Guinier played a central role.

It
always remained a great pleasure for André Guinier to learn about and
discuss every novel result discovered in the laboratory. Many of us
have benefited from his enlightening advice and he will be very much
missed.

André Guinier was always closely concerned with
instrumentation problems; most of the work described above would not
have been achieved without specific devices involving a well defined
monochromatic beam of high intensity. This developed into the well
known Guinier camera, which is used at the present time in X-ray
laboratories worldwide. In a slightly different field, he played an
inciting role in the conception of the electron microprobe (EPM), which
Raimond Castaing developed into an outstanding scientific instrument.

André
Guinier's teaching was on a par with his research activity. He liked to
be as simple as possible in his physics lectures for undergraduates as
well as when developing new concepts for his research students. His
perspicacity, associated with his wide range of competence, explains
the worldwide interest aroused by the publication of his books, the
best known of which is certainly *Théorie et Technique de la Radiocristallographie*,
a major achievement with French editions in 1956 and 1965, which was
translated into English, Japanese, Russian and Chinese. After his
retirement, he also spent a considerable part of his time in writing
scientific books for a wide audience.

During his exceptionally
long scientific career, André Guinier assumed national and
international responsibilities: he was President of the French Society
of Mineralogy and Crystallography (1960), President of the French
Society of Physics (1962), Member of the Executive Committee of the
International Union of Crystallography (IUCr) (1954-1960), Chair of the
Commission on Crystallographic Teaching of the IUCr (1966-1969),
President of the IUCr (1969-1972) and Immediate Past President of the
IUCr (1972-1975).

---

*Acta Cryst* (2001). A**57**, 1-3   [ doi:10.1107/S0108767300015440 ]
