# Supplementary material for: Space groups and crystallographic symmetry: writing a multi-featured tutorial in a new style
Source: Acta Crystallogr E Crystallogr Commun. 2021 Jul 16;77(Pt 9):857–63. doi: 10.1107/S2056989021007039 (PMC8423017; doi:10.1107/S2056989021007039)
Supplement: Supplementary file 1 [file e-77-00857-sup2.zip › symandsg/Main/guinier_files/es0293hdr.htm]

(IUCr) André Guinier (1911-2000)

|  |  |  |
| --- | --- | --- |
|  |  |  |
|  | | |

Go to section:TopReferences
