# Supplementary material for: Space groups and crystallographic symmetry: writing a multi-featured tutorial in a new style
Source: Acta Crystallogr E Crystallogr Commun. 2021 Jul 16;77(Pt 9):857–63. doi: 10.1107/S2056989021007039 (PMC8423017; doi:10.1107/S2056989021007039)
Supplement: Supplementary file 1 [file e-77-00857-sup2.zip › symandsg/Main/hauyii.htm]

Life and Work Hauy

|  |  |  |  |  |  |  |  |
| --- | --- | --- | --- | --- | --- | --- | --- |
| **Home** | **AmMin** | **GMR** | **RiMG** | **Collectors Corner** | **Directory** | **Short Courses** | **Contacts** |
| --- | | | | | | | |

---

*Volume 3, pages 60-89,
1918*


THE LIFE AND WORK OF HAÜY

GEORGE F. KUNZ

*Chairman, Haüy Celebration Committee*

    
If a keen and observant mineralogist had not accidentally dropped a
group of calcite crystals belonging to a French
collector in such a manner that one crystal was broken; and if this
crystal had not been presented to the mineralogist
who broke it; and if he had not possessed the wonderful gift of
thoughtful and conscientious observation that is
characteristic of the people of France-even tho he was but the son of a
weaver (indeed, this weaver probably possessed
the same qualities); and if he had not interested himself in botany and
had not casually listened to a lecture of the
great Daubenton, professor of mineralogy and curator at the Muséum
d'Histoire Naturelle at Paris; then modern
crystallography would not have been founded on the scientific basis on
which it now rests.

    
It is because of this event that we are here assembled, at the 175th
anniversary of the birth of this mineralogist, to
celebrate and do honor to him, and to the great institution with which
he was later connected, and to urge, as an added
inspiration to our young men, the importance of the study of
mineralogy, especially of crystallography, as this would be
of the greatest aid to the nation in times of war and in times of
peace. So greatly has Haüy's work been appreciated
that at this time of stress no difficulty was found in obtaining the
cooperation of eminent scientists in nearly forty
institutions in the United States and Canada, who have selected as
their honorary chairman the present incumbent of the
chair of mineralogy in the Muséum d'Histoire Naturelle, Professor
Alfred Lacroix, who not only has so ably contributed
to the science of mineralogy, but has been further honored with being
called to the secretaryship of the Académie des
Sciences, a post once occupied by the famous Georges Cuvier, who wrote
the great eulogy of Abbé Haüy.

    
The life of Abbé Haüy, the founder of the science of crystallography,
furnishes us with one of the many instances in
which a great scientist has developed out of the simplest surroundings,
and without having enjoyed any very exceptional
advantages.

PLATE 6

PORTRAIT OF Haüy, in the possession of Prof. Alfred Lacroix, who occupies the chair of mineralogy in the Muséum
d'Histoire Naturelle, formerly filled by Haüy, from 1802 to 1822.

    
René-Just Haüy, the son of a poor weaver, Just Haüy, and of his wife
Magdeleine Candelot, was born in the town of St. Just, Dept. Oise,
France, on February 28,
1743.^1^ In his
childhood his religious bent found expression in frequent attendance at the simple services of the village church,
whither he may perhaps have been attracted partly by his natural love of music. His assiduity was remarked by the prior
of an abbey of Premonstrants near the village who, after having taken occasion to talk with him several times, became
impressed with the liveliness of his intelligence, and had him given some lessons by certain of the monks. Believing
that he would prove worthy of any assistance that might be afforded him to secure better educational advantages than he
could hope for in his native village, the prior suggested to the boy's mother that she take him for a while to Paris,
where, by means of letters of recommendation, Haüy could secure entrance to some educational institution, so as to
develop his abilities.

    
The mother's means were very scant, barely sufficient to enable her to
subsist for a few months in the great city, but
her love for her son and her ambition for his success induced her to
take the risk. At first, however, the boy could
find no better position in Paris than that of chorister in a small
church of the St. Antoine quarter, not an uncongenial
occupation, it is true, for a lover of church music, but still one that
offered no prospect of mental culture. However,
the boy's patient and willing acceptance of this place was before long
rewarded. One of the good prior's letters
influenced a Parisian friend to secure for Haüy a scholarship in the
College of Navarre, where his devotion to his
studies and his general good conduct won for him the favor of those in
charge of the institution, so that he was
accorded the position of usher; and subsequently, in 1764, before he
had attained his twenty-first year, an appointment
as regent (master) of the fourth class. So deceptive are physical
indications that when he was named for this position
he heard one of his colleagues remark, "There is a young man who will
not live out the year." At the end of a
few years he was appointed regent of the second class in an associated
educational establishment, the Cardinal Lemoine
College.^2^

PLATE 7

Portrait of René Just Haüy. Drawn by Ambroise Tardieu (1788-1841)

    
While still in the Collège de Navarre, Haüy was attracted to the study
of physics, and followed a course of lectures
delivered there by Brisson. To these he listened with such lively
interest, and watched the experiments performed by the
lecturer so closely, that he was able to repeat many of them for his
own instruction. However, altho he was thus laying
a foundation for his later work, his knowledge of natural history was
still very slight. His first earnest effort in
this direction was due to his friendly relations with a fellow
instructor, the Abbé Charles Lhomond (1727-1794), who
composed a number of elementary educational books for the use of young
people. With him Haüy frequently took long walks
into the suburbs of Paris, during which Lhomond, an. enthusiastic
botanist, busied himself with collecting specimens of
plant life. The young master soon began to feel regret that he could
not intelligently share in the interest of his
friend, and this moved him to take up the study by himself, saying
nothing to Lhomond about it until he should have
attained some degree of proficiency. A good opportunity presented
itself during one of his vacations, which he passed in
his native village, for he found there a monk who knew something of
botany. Haüy profited by this chance so well that
on his return to Paris he was able to give Lhomond a pleasant surprise
by proving, on their next walking tour, that his
own knowledge of botany was now almost on a level with that of his
friend. Indeed, he published in the Mémoires of the
Académie for 1785 (p. 210) a paper on the "Manière de faire des
herbiers." In the preparation of his own
herbarium he employed a special process for preserving the colors of
flowers.

    
Thus, little by little, Haüy was moving forward along the path that was
eventually to lead him to the discovery which
has made him famous. It was but natural that his botanical studies
should render him a frequent visitor to the Jardin du
Roi, now the Jardin des Plantes, the establishment of which dates back
to 1626.^3^ On one of these visits he was led,
almost by chance, to assist at a lecture on mineralogy by Daubenton. His study of physics in general had been sufficiently thoro to enable
him to grasp the general principles of mineralogical science, and his quick perceptions were impressed with the
difference between the systematic classification of plant forms, and the comparative lack of system in the arrangement
of mineral forms. He soon realized that this must be due to the fact that while it was comparatively easy to trace the
relationship of the groups of plants by the constancy of even the most complicated forms, the classification of minerals
presented much greater difficulty, since in many cases the external forms of those of identical chemical composition
were widely diverse.

    
The train of thought suggested by these reflections was given a
definite direction by what we may call a happy chance,
altho the real value of such a fortuitous happening strictly depends
upon the preparation of the mind to seize upon its
real significance. Haüy himself relates this circumstance as follows in
his "Traité":^4^

    
"The observation I have just noted is that which has served to develop my ideas on the structure of crystals. It
presented itself in the case of a crystal that the citizen Defrance^5^ was kind enough to give me just after it had broken
off from a group this enlightened amateur was showing me, and which formed part of his mineralogical collection. The
prism had a single fracture along one of the edges of the base, by which it had been attached to the rest of the group.
Instead of placing it in the collection I was then forming, I tried to divide it in other directions, and I succeeded,
after several attempts, in extracting its rhomboid nucleus. This at once surprised me, and gave me the hope that I could
advance beyond this first step."

    
In his eulogy of Haüy, delivered before the Academy of Sciences June 2,
1823, the great Cuvier repeats this story with
the slight variation that Haüy "by a fortunate awkwardness" had let the
group fall so that the crystal broke
off. It is a rather curious circumstance that some six or seven years
before this time a young Swedish chemist named
Gahn, later professor at Abo, Finland, in breaking a pyramidal calcite
crystal had also remarked its rhomboid nucleus.
He communicated his observation to his master Bergmann, of Upsala, but
the latter failed to realize its significance and, instead of carrying out experimental research on other crystals, lost himself in a maze of hypotheses.

    
Haüy, on the contrary, immediately proceeded to utilize this chance
observation. On his return to his room he took a
piece of spar of hexahedral form and, after breaking it carefully and
skillfully, found the fragments of a rhomboid; the
same proved true of lenticular spar. Pursuing his researches, he
experimented with a great variety of crystals and found
that the principle constantly held good. The variation, in most cases,
of the exterior form from the primal form was due
to the wearing away of superposed layers, either at the angles or along
the edges, and to a special and peculiar
arrangement of the elementary molecules, subordinated to the same
structural laws.

    In view of the
fact that Romé de l'Isle's investigations and partial results in the domain of crystallography were so
soon eclipsed by those of Haüy, we may perhaps forgive him for the satirical turn' of certain of his criticisms of his
rival. Thus Cuvier tells us that he is said to have found pleasure in calling Haüy a "cristalloclaste"
because he was a breaker of crystals, just as in the Eastern. Empire those who shattered the church images they
considered as lures to idolatry were called "iconoclasts."^6^

    
Haüy now felt sufficient confidence in the value of his observations
and the deductions he had made from them to speak
of his results to Daubenton, and the latter communicated them to
Laplace, who advised Haüy to present them to the
Academy of Sciences. It was not an easy matter to overcome his
disinclination to do so, not from any want of confidence
in the truth of his discovery but from an inborn lack of
self-assurance. Finally, on January 10, 1781, he read before
the Academy his first memoir, in which he treated of the
crystallization of garnets and calcareous spars. In accordance
with the rules of the Academy a communication made by a non-member was
only published according to a report made by one
or more members. In the present case this duty devolved upon Daubenton
and Bezout, but their report, published in the
following month, showed that they had failed to grasp the full
importance of Haüy's communication. On January 22,
however, the latter read a second memoir in which he confined himself
strictly to the calcites. This time, in a report by the same members,
the publication
of which was delayed until the following December, the scope of the
discovery was satisfactorily presented, and justice
was done to its importance.

    
As an acknowledgment of the great service Haüy had rendered to the
science of mineralogy, the Academy was eager to
include him among its members, but there was at this time no vacancy
either in the section of physics or in that of
mineralogy. However, he was elected as  *adjoint* in the class of botany, a vacancy caused by the promotion of the botanist
Jussieu to the rank of *associé*. When it became necessary for him to appear as a candidate, a difficulty arose about
his dress, since he was exceedingly loath to lay aside the strictly ecclesiastical garb prescribed by the old rules of
the church, and it required the authoritative opinion of a doctor of the Sorbonne to remove his scruples. The date of
his election was February 12, 1783, and five years later he became *associé* in the class of natural history and
mineralogy.^7^

    
An unofficial recognition of Haüy's merit, one scarcely less impressive
than his speedy election to the Academy, was
the request made by such masters of science as Lagrange, Laplace,
Fourcroy, Berthollet, and De Morveau, that the
diffident master of the second class in the Cardinal Lemoine College
should deliver a special course of lectures for
them on his new theory. Indeed, this theory was so bound up with his
personality, he was so exclusively the fountainhead
whence all exact knowledge of it must flow, that the master minds in
other fields were for the time being obliged to
content themselves with the part of learners.

    
With the lapse of twenty years from the beginning of Haüy's service in
the university, he became entitled to receive a
pension as emeritus. This he hastened to ask for, as by adding it to
the income from a small benefice, he would be
provided with what was strictly necessary; he continued to lodge in the
Cardinal Lemoine College. It remained, however,
requisite that this small sum should be assured to him. Unfortunately,
political events were about to dispose of things
otherwise. One of the radical measures adopted by the Constituent
Assembly was a law requiring the clergy to take. an
oath of adhesion to the new form of government, under penalty of being
deprived of their emoluments and of their places. Haüy's sensitive
conscience opposed a bar to his taking this oath. He therefore became
subject to the penalty imposed upon those who refused, and as a result
he found himself stripped of his little income.

PLATE 9.

PORTRAITS OF HAÜY, in his uniform as member of the Académie des Sciences.

    
This punishment, however, fell far short of satisfying the
anti-clerical partisans of the Revolution, and by another
decree passed a few days after the assault on the Tuileries, August 10,
1792, and the imprisonment of the royal family,
it was ordered that all priests who had failed to take the oath should
be imprisoned. Haüy, in his retired life, solely
devoted to scientific study, had but a rather vague sense of the rapid
political changes transpiring in Paris, and he
was therefore much startled when one day a party of rough men violently
entered his modest retreat in the college. Their
first question was whether he had any firearms with him. Haüy, who had
quickly regained his wonted composure, answered,
"I have none but this," at the same time drawing a spark from an
electric machine. His quiet bearing and this
half-humorous response served to quiet the men a little, but they soon
began a hunt for compromising material of any
kind. They seized his papers, covered with algebraic formulas, which
they may have suspected to be mysterious and
treasonable ciphers, and what was much more trying for him, tumbled
about his precious minerals, his sole and only
treasure. The search being completed, they bore him away to the nearby
seminary of St. Formin, which had been turned
into a prison. Here he found himself shut up with all the recalcitrant
priests and masters of this quarter of Paris.

    
The change from his simple room in the college to a similar one in the
seminary seemed a matter of comparative
indifference, and the presence of many of his friends still further
tranquilized him. He was also accorded the favor of
having the drawers filled with his precious crystals brought to him,
and was able to put them again into some sort of
order. The very real risk from the passions of the bloodthirsty mob he
was far from realizing. Fortunately he had
friends outside who were better aware of the dangers of his position.
Among them was his colleague of a later time,
Geoffroy St. Hilaire, who left no stone unturned to secure the
liberation of his friend. His appeal and their own
affection and respect for Haüy induced members of the Academy and
functionaries of the Jardin du Roi to subject
themselves to the humiliation and rebuffs they were sure to encounter
in asking any favor of the ferocious men then in control of Paris. There was, however, so little possible excuse for persecuting
one so harmless as Haüy that their efforts were at last crowned with success, and an order of release was granted.
Armed with this, Geoffroy St. Hilaire lost no time in hastening to the St. Formin seminary. On his arrival there, rather
late in the day, he found Haüy perfectly unconcerned and quite disinclined to leave that same evening. When the morrow
came it was still hard to persuade him of the imminent peril in which he was, and to induce him to leave the place. How
important haste was in this matter was soon made evident by the dreadful prison massacres that took place a few days
later, on September 2, 1792, when eighty priests who had shared his captivity at the St. Formin were done to death by
the brutal mob.

    
This was the only serious danger to which Haüy was subjected during the
Revolution, and his experience of the active
side of the great political convulsion was limited to a single
appearance at the review of the battalion to which he had
been assigned. His obvious physical unfitness for military service
procured him an immediate discharge. He was called on
November 9, 1794, to the professorship of physics at the École Normale;
on April 17, 1795, the Commission of Weights
and Measures appointed him as secretary, and in the same year he was
made Curator of the École des Mines. Here it was
that he composed his great *Traité de Minéralogie*, originally published serially in the  *Journal des Mines*, and later
issued, in 1801, in four octavo volumes. This work was immediately recognized as the most important contribution yet
offered by France to the science of mineralogy, one that placed the country in the very forefront of the European
nations in this science. In it the author makes crystallography the basis of his determination of mineral species,
relegating the data derived from chemical analysis to second rank. The mathematical precision with which the
crystallographic facts could be presented provided an absolutely exact basis for mineralogy which it had lacked before
that time, as the correctness of the crystallographic determinations could always be proved by rigid geometrical tests.

     
On Daubenton's death in. 1800 Haüy seemed clearly indicated as his
legitimate successor in the position of professor of
mineralogy at the Muséum, but he himself earnestly solicited that Dolomieu should be chosen, altho the latter was at this time
confined in a Sicilian prison, whence he was only able to communicate with his friends by means of a few lines written
with lampblack on slivers of wood, even this poor resource being obtained by bribing his jailor. He was nevertheless
elected to the professorship, but only occupied the post for a brief time after his return from captivity, for his
health had been undermined by his sufferings. He died in 1801, and in the following year Haüy was chosen to succeed
him.

PLATE 10.

D'APRES J. BOILLY

PORTRAIT OF HAÜY, engraved from drawing by J. Boilly.

    
Haüy's entrance into this wider sphere of activity gave new life to the
study of mineralogy in the Muséum, for the
great reputation he had already acquired attracted students from all
parts of Europe. His hearers were not only
impressed by the lucidity of his expositions, but were also charmed by
the unaffected simplicity and kindliness of his
manner.

    
When public worship was restored in France during the Consulate, Haüy
was given the title of honorary canon of Notre
Dame, and on the institution of the Légion d'Honneur he was made
chevalier in the new order. From the constitution of the
Institute in 1795, he formed part of it, with the other members of the
Academy of Sciences. Napoleon, the First Consul,
who, as is well known, fully realized the great importance of the
higher education for the upbuilding of a nation's fame
and prosperity, commissioned Haüy to write a treatise on physics to be
used in the  *lycées* of France. On learning that
he was to be given but six months' time for the completion of a work of this scope, the scientist hesitated to undertake
it, but was at last persuaded to do so. He took up the task so zealously that at the expiration of four months he was
able to offer the two volumes of his "Traité de Physique" to the First Consul.

    
While exiled on the island of Elba in 1814, Napoleon occupied some of his enforced leisure in reading this treatise, and
on his triumphant return to Paris in the spring of 1815, lie promoted Haüy to the rank of officer in the Légion
d'Honneur,^8^ a grade that was taken from him in the Restoration which followed soon.

    
When the allied sovereigns assembled in Paris on Napoleon's downfall
Haüy's modest lodging was visited by the King
of Prussia, the father of William I of Germany, by Alexander I of
Russia, and by Archduke John of Austria. The Austrian
archdukes are said to have tried to induce him to sell his collection by the tempting offer of 600,000 francs for it. However, he declared that he had resolved to bequeath it to France. His
generous intention was left unfulfilled, and some years after his death his heirs were induced to sell it in England,
where it remained until the overthrow of Louis Philip, in 1848; it was then in the possession of the Duke of Buckingham,
and was bought from him for £325 or 8,125 francs by the Second Republic, and placed in the Muséum d'Histoire Naturelle,
its natural resting place.

    
In spite of the high reputation he had gained as a scientist, Haüy
suffered from straitened circumstances in the
closing years of his life. With barely enough to provide for his own
absolute needs, he was obliged to receive and care
for. his brother Valentin, who, after founding the "Institution des
Jeunes Aveugles" in Paris, traveled to
Germany and Russia and founded there similar institutions for the
blind. From these wanderings he returned with broken
health and without money, and thus imposed an added burden upon
René-Just Haüy. Valentin died a few days before his
illustrious brother, on March 19, 1822. However, the latter's simple
habits and almost ascetic mode of living made him
less sensitive to the inconveniences resulting from his lack of means
than would have been the case with many others;
indeed, he is said to have even been able to give a little aid now and
then to those poorer than himself.

    
Altho never robust, he must have had an essentially sound constitution,
for he had attained the age of seventy-nine
years when he passed away on June 3, 1822. His death was the result of
an accident, altho the fatal outcome was
undoubtedly due to his physical condition. He fell, while walking
across his room, and broke his thigh-bone. An abscess
formed at the point of fracture and an acute fever which supervened put
an end to his life in a few days. M. Brongniart,
who had been his assistant in teaching for several years, was called to
fill his place as professor in the Muséum.

      Saint-Just-en-Chaussée (dept. Oise), for it was on
this day that a monument was dedicated there to the brothers Haüy, the scientist René-Just and the philanthropist
Valentin. The title the latter possesses to the grateful remembrance of all who are interested in what makes life more
bearable for those afflicted with blindness must not be forgotten in our admiration for the more famous discoverer of crystallography. Valentin Haüy's methods for enabling those born blind to share the
power of reading with their more favored brothers and sisters merit a place alongside of the methods initiated by the
celebrated Abbé de 'l'Épée to give the deaf-mutes the power to communicate with those enjoying the powers of hearing
and speech.

PLATE 11.

PORTRAITS OF THE BROTHERS HAÜY (RENE JUST AND VALENTIN) drawn by J. Boilly, engraved by Alphonse Boilly.

     One of the most illustrious continuers of Haüy's work, the mineralogist and geologist François
Mallard^9^ (1833-1894);
has paid him the following just tribute.^10^

    
"The science of crystallography was entirely created by Haüy's genius,
and his successors have had little to do
except to perfect the details of his work. No other branch of human
knowledge is in the same degree the work of one man
alone."

    
It is but natural, in view of the surprising revelations as to the
structural relationship of minerals due to Haüy's
discoveries and researches, that mineralogy, properly so called, should
have been for a time thrust into the background.
However, during the first quarter of the nineteenth century the
development of mineralogical chemistry greatly changed
the aspect of thing in this respect, and at the time of Haüy's death in
1822, a reaction of the mineralogical chemists
against the exclusive pretensions of the crystallographers had already
taken place.

   The most
illustrious predecessor of Haüy in the study of crystal forms was
unquestionably Romé de l'Isle (1736-1790),
who in 1772 published his  *Essai de Cristallographie*, in which he foresaw the importance of crystalline symmetry and
announced the constance of crystal faces. The views expressed here were presented in a fuller form in a second edition
issued in 1783, in three volumes, and bearing the title  *Cristallographie*. To this book Linnaeus gives the following high
praise: "Among the works produced in this century on mineralogy, it is certain that your  *Cristallographie* takes the
first place. It testifies to your penetrating intellect, to the immense number of observations you have made, to your
wide reading, and, what is rare indeed, to your kindly attitude toward myself."

    In this work Romé de
l'Isle embodied the results he had been able to obtain by the help of Carangeot's recently
contrived goniometer, for accurately measuring the angles of crystals; this instrument having been made from Carangeot's drawings
by the mechanician Vinçard. There can be no doubt that crystallography owed much to the mechanical aid the goniometer
afforded; indeed, it has frequently been the case that important discoveries in pure science have depended upon the
invention of new mechanical apparatus. In 1809, Dr. Wollaston (1766-1829) invented a perfected instrument, the reflection
goniometer, those of the Carangeot type subsequently being known as contact goniometers. For the proper measurement of
small crystals the reflection goniometer is needed, but for the larger ones the more easily and quickly operated contact
goniometer has been found useful.

   
The credit of publishing the first accurate observations on a type of
crystal has been given to Nicholas Steno, a Danish,
physician, who later became Bishop of Titupolis. In a work published in
1669,^11^ he treated of the characteristics of
quartz crystals, carefully noting that whatever may be their apparent diversity of form the angles between similar pairs
of faces are always the same. The theory of the upbuilding of a crystal about a definite and constant nucleus is also
enunciated by him. The first book devoted to crystallography as a separate branch of mineralogy was the  *Prodromus
Crystallographiae* of M. A. Capeller. This appeared in Lucerne in 1723, nearly fifty years before the publication of De
l'Isle's  *Essai*.

    
Of the immediate predecessors or contemporaries of Romé de l'Isle one
of the most noted was the Swedish physicist
Torbern Olaf Bergmann, whose essay on the crystal forms of calcspar was
printed in 1773 in the Acta of the Royal Society
of Sciences in Upsala. Had he followed out the indications supplied by
his observations he might have occupied a much
higher place than he does in the history of crystallography. A year
later, in 1774, Abraham Gottlob Werner (1750-1817)
in his writing  *Von den äuserlichen Kennzeichen der Fossilien* treated of the various forms of crystals, and undertook
a partial classification of them.

     Of the special merits of Haüy's discovery and theory, Von Kobell has written as follows:^12^

    
"The laws governing crystallization as presented by Haüy not only
indicated the form derivable from a given
nucleus, but at the same time those which could not be derived
therefrom, and showed that the dimensions given by
calculation constituted the accurate definition of those approximately
determinable by the goniometer. One of the most
important results of Haüy's researches was the discovery of the law of
symmetry, according to which when one form of
crystallization is modified by its combination with other forms, all
the similar parts, the edges, angles and faces, are
always modified at the same time and in the same way. Haüy called the
goniometer a kind of geometrical magnifier, which
enabled the observer to perceive excessively slight differences, which
could not be perceived ocularly. To him is due
the credit of recognizing that the so-called Spanish chrysolite was
apatite, long before Vauquelin reached the same
result thru chemical analysis. He also recognized before Klaproth that
the mineral from Norway called vesuvianite was in
reality zircon. His crystallography first brought the beryl and the
emerald into the same species, and showed that
euclase constituted a peculiar species.

     We have already alluded to the  *Traité de Physique* written in 1803 at the instance of Napoleon Bonaparte, when First
Consul. In his introduction to this work Haüy remarks that nothing was better calculated to stimulate his zeal than the
hope of realizing the views of the "Hero of France," who wished to provide for the students enrolled in the
higher schools a grade of instruction calculated to develop their judgment by the acquisition of a thoroly systematized
learning, which would assure their success in whatever functions they might later be called upon to fulfill. In this
connection, he insists upon the fact that any exposition becomes vague when it is strictly confined to its general
outlines, and that details may fairly be called the touchstones of theories, for they either guarantee their value or
reveal their weakness.

     In 1809, eight years after the publication of his  *Traité de Minéralogie*, wherein he states the essential principles of
his discovery of the laws governing crystallization, Haüy issued a work entitled  *Tableau Comparatif des Résultats de
la Cristallographie, relativement d la Classification des Minéraux*.^l3^ He herein states that his principal object in
publishing the book is his wish to give to those who had followed his course of lectures a full tabulation of his mineralogical methods, revised in
accordance with the discoveries and observations with which science had been enriched since the publication of his  *Traité
de Minéralogie*. New minerals had been discovered by travellers, and a more thoro study of those already classified had
shown many of them to be related to species with which no connection had been suspected.^14^

    
One of the peculiar merits of crystallography, in his opinion, was that
it made use of what might not unjustly be termed
a "palpable means" of verification, and that it depended upon the kind
of anatomy of which crystals are
susceptible, as well as upon modifications of a structure which, to a
certain extent in the case of minerals, is what
the organism is in regard to beings endowed with life. Thus mineralogy
was made to share with zoology and botany the
merit of making a direct appeal to the eye.^15^

    
That with all his single-minded devotion to the cause of pure science
Haüy was not without a quick sense of the value
of applying scientific knowledge to useful ends, is shown in one of his
latest works,  *Traité des Caractéres
Physiques des Pierres Précieuses* .^16^
In his introduction, to emphasize the chemical importance of gem-stones, he
alludes to the fact that thru the analysis of the jacinth and the beryl two new elements had been discovered, Klaproth
having recognized zirconium in the former, while the presence of another new element, glucinum, had been found by
Vauquelin in beryl. In the preparation of this treatise Haüy asserts that his aim is to present clearly the physical
characteristics observable in precious stones that are cut as gems, and to arrange the data in the form of rules that
may be used for the determination of these gems. He believes that such rules will prove of great value to the gemcutters
as well as to the dealers in gems, since they enable them to verify impressions received thru the eye.

    
However, the chief object of the work is to help those forming
collections of gem-stones, as among the collectors of
these objects, which, as Haüy writes, "form part of what we look upon as our riches," there was but little positive knowledge of     
them. Of this he says:

    
"The idea that what has been offered them as an Oriental ruby is really
one of those eagerly-sought stones which
occupy the first rank after the diamond, is a satisfaction that they
only enjoy on the bare word of the dealer. I have
therefore thought that they would gladly assure themselves, by decisive
tests, of the authenticity of an object which
had cost them a sum proportionate to their appreciation of it, and
learn whether the name under which they had acquired
it was really that to which its characteristics entitled it."

    
It is interesting to learn that the completion of this treatise was
hastened by the impression a perusal of the
unfinished manuscript produced upon the great English banker and
gem-collector, Henry Philip Hope, while he was on a
visit to Paris. Haüy adds that as a testimony of his appreciation, Hope
generously donated to the scientist's
collection a number of rare specimens which were lacking, their place
being but poorly supplied by analogous material
not possessing all their characteristics. Hope also took occasion to
supply himself with the mechanical instruments
necessary to prove experimentally the existence of the several
properties of the gem-stones.

     One of the earliest records of Haüy's crystallographic investigations is the  *Mémoire* on the structure of feldspar
crystals, read by him before the Académie Royale des Sciences, June 26, 1784. In it he notes that M. Demarest, a member
of the Académie, had brought back from his travels, in 1770, specimens of two very interesting varieties of feldspar,
one of which he had found among the lavas of Auvergne and the other in the province of Limousin. He deposited in the
Cabinet du Roi an especially fine specimen of the latter variety, which was a six-faced prism. The first variety was
also of prismatic form, but with ten faces. Haüy describes these crystals exhaustively in his  *Mémoire*, treating them
as secondary forms of feldspar. Combined with the examples described by Padre Pini, professor of natural history in
Milan, in a paper published in 1779, Haüy found that they constituted one of the most complete series of crystal forms
that had yet been observed. He adds:

    
"Besides the fact that feldspar crystals already merit the attention of
naturalists for the reason that they are
forms of one of the most interesting substances in the mineral kingdom,
I have also been incited to communicate my observations to the Académie, on account of the new proofs they appear to furnish
me of the principles I enunciated in the outline of a theory regarding crystals, published toward the end of the year
1783. I have not yet seen feldspar in its simple primitive form, freed from all accidental faces. This form would be
that of a quadrilateral prism."

The characteristic letters of Haüy here added give an excellent idea of his quality of mind, of the grace of his style
and of his urbanity. The longer one is translated from a facsimile reproduction in  *Isographie des Hommes Célèbres*
(Paris, 1843), the material for which was assembled by T. Delarue.

PARIS, September 8, 1807

Sir: It would be difficult for me to recall a visit which has given me greater cause to feel flattered at having
received it, than that of Mr. Nass. It combined the advantage of becoming acquainted with a very distinguished savant
and the lively satisfaction of receiving from you a very precious gift accompanied with an exceedingly kind letter. Your
crystals of arsenical cobalt offer a variety lacking in my collection, of which they now form one of the finest
ornaments. I finished, a few days ago, my public course of lectures on mineralogy, and I hasten to fulfill the promise I
made you to send you an article for the second volume of your excellent manual. It is an epitome of what is new in my
lectures relative to the science of which they treated. I add it to my letter, begging you, Sir, to make all the changes
and modifications you may judge proper. I speak of myself in the third person, because I have thought it might perhaps
be better that the article should be considered to have been communicated to you by one of those who assisted at my
course. I could have written at greater length, had I not feared to exceed the limits. I will add here, for yourself
alone, a résumé of the observations I have made on a letter written anonymously to M. Berthollet, and which you have
doubtless read in the  *Journal de Physique*, July, 1807, vol. VI. The writer seems to have failed to grasp
the method I employ in the determination of mineral species. It is not only in the results of mechanical division, or of
cleavage, that I make it consist, but also in the relations established by calculation between the dimensions of the
primitive form indicated by the operations in question. Thus the cymophane, the apophillite (icbthyophthalmite), the
peridot, the mésotype, etc., having been mechanically divided, give as the primitive form a rectangular parallelopiped.
On the other hand the phosphate of lime, the sulfate of copper, etc., give hexagonal prisms, such as are determined by
the laws of decrescence which produce their secondary forms, varying from one species to another; and it is above all
this variation that, in my method, serves to trace the lines of demarcation which sharply separate the bodies I take as
types of species. A fortiori, the same theory indicates the distinction of species whose primitive forms differ by the
respective disposition of their faces. There results from this, in regard to each one of the species in question, a
particular system of crystallization to which it is impossible, without violating nature, to bring the varieties
comprised in another species. All the subordinate divisions that can be conceived in the sense of diagonals of primitive
forms, or in other directions (and in this connection the anonymous writer establishes rules by supposing a multitude of
cleavages I have never seen), all these divisions, I repeat, will not become recognizable from the divisions proven to
be unrecognizable by calculation. The writer's objections come a little late, since my system is complete from beginning
to end, and I can demonstrate to anyone the application of my principles to each one of the species which compose it. I
have believed that I ought to communicate these observations to my students, several of whom have already made them on
their own account. However, I have never made a printed reply to any objections which have been raised against me; when
they seemed to me to be well founded, I accepted the correction. I recall that a famous savant, having composed an
answer to an attack directed against him, read it to Daubenton, saying to him; "Is this not victorious?"
"Without doubt," replied Daubenton, "but you are going to have a war on your hands, and what victory is
comparable to peace?" Daubenton was my master in mineralogy; I have also taken him as my master in morality. Will you pardon me, Sir, for having entered into all these details with
you? I beg you at least to look upon them as a witness of how highly I prize your esteem.

    
Accept the expression of my lively gratitude and that of the great
respect with which I have the honor to be, Sir,

Your Very Humble and Very Obedient Servant, HAÜY

    
If you see fit, Sir, to make any changes in my article, I ask
indulgence as to what I write of the arsenical cobalt. I
would be flattered to be able to send you some small specimens from
among my duplicates; but I should wish to know those
which you lack, especially of the minerals of France.

PLATE 12.

LETTER OF HAÜY TO MONSIEUR MARRON, AND NOTE To DR. COGSWELL; reduced.

PARIS, Sept. 4, 1813

     *Sir*: The pleasure caused me by the reading of the verses, as elegant as they are harmonious, which you have the goodness
to address to me, proves to me that the study of mineralogy has not made me forget the beauties of Virgil and of Horace,
which I have passed twenty years of my life in cultivating. The picture that you draw of the results of my work in
Natural History, testifies on your part to that indulgence which is the usual associate of great merit. But I would be
doing myself a wrong if I should disavow the good opinion you seem to me, Sir, to have formed of me in the matter of
moral qualities, above all at a time when I feel myself so powerfully moved by gratitude. Accept the homage of that
which I have so justly dedicated to you, and permit me to join with it the assurance of the very distinguished regard
with which I have the honor to be

Sir

Your Very Humble and Very Obedient Servant

HAÜY

To Monsieur Marron.

     A copy of one of Haüy's writings, now in the New York Public Library, an offprint of his memoir
 *Des surfaces vibrantes,*
bears on the title leaf an autograph presentation by the author to "Monsieur Kogswell." This was Dr. Joseph G.
Cogswell, the first Superintendent of the Astor Library, who visited Paris in 1819, during his three years' residence in
Europe.^17^

I have the honor, my dear sir, of sending you a synopsis for the *Journal
Encyclopédique*, with a few words for the authors of this  publication. I beg you to be so kind as to enclose these two
papers in an envelope and to send them to the gentlemen in a question, with a copy of the work. Would you also be so kind as 
to announce it in the *Gazette*; and on the public posters. I charge
myself with the  *Journal de Pa*ris, the  *Mercure*, the  *Année Littéraire*, the
 *Journal de Monsieur*, the  *Annonces* of the Abbé de Fonsenai [?], the
 *Journal des Savans*, and the law journal of M. Buchoz. The
binder whom I saw this morning has promised me, for Sunday, my eighty
copies, from which I shall make my
presentations.

I have the honor of being, sir, 

With the highest regard, 

Your very humble and 

Obedient servant  HAÜY

December 9th

(Collection Berard)

Plate 13

 LETTER of HAÜY, dated December 9, announcing that he sends a notice of his latest work for the " Journal
Encyclopedique." To M. Barrois, Libraire.

     Among the many titles and honors bestowed upon Haüy, the following is a list of the principal ones:

    
Member of the Institut National (Académie des Sciences); Honorary Canon
of the Eglise Métropolitain de Paris (Notre
Dame); Professor of Mineralogy at the Museum d'Histoire Naturelle;
Professor of Mineralogy in the Faculty of Sciences
of the Université Royale; Member of the Académie Royale des Sciences of
Berlin and of the Société des Scrutateurs de
la Nature (Naturforscher) in the same place; Member of the Imperial
Academy of Sciences of St. Petersburg; of the Royal
Academies of Sciences of Lisbon and of Munich; of the Imperial
University of Vilna; of the Geological Society of London;
of the Mineralogical Society of Jena; of the Société Italienne des
Sciences; of the Société des Sciences de Haarlem,
etc. Officer of the Legion d'Honneur and Chevalier of the Order of St.
Michael, of Bavaria.

BIBLIOGRAPHY OF HAÜY'S WRITINGS.

Essai d'un théorie sur la structure des cristaux, Paris, 1784.

Exposition raisonnée de la théorie de l'électricité et du magnétisme, d'après les principes d'AEpinus, Paris,
1787.

De la structure considérée comme caractère distinctif des minéraux, Paris, 1793.

Exposition de la théorie de la structure des cristaux, Paris, 1793.

Extrait d'un traité élémentaire de minéralogie, Paris, 1797.

Traité de minéralogie, 4 vols., Paris, 1801; 2d ed. in 1822.

Traité élémentaire de physique, 2 vols., Paris, 1803; 2d ed. in 1806; 3d in 1821.

Tableau comparatif des résultats de la cristallographie, et de l'analyse chimique relativement à la classification des
minéraux, Paris, 1809.

Traité des pierres précieuses, Paris, 1817.

Traité de cristallographie, 2 vols., Paris, 1822.

Sur l'alumine fluatée (*Journ. Phys*., 49, 1799).

Sur l'électricité de l'apatite (*Journ. Min*., 2, 1796).

Sur les services rendus a là science minéralogique de Romé de l'Isle (*Journ. Min.*, 4, 1796).

Sur la forme des stalactites (*Journ. Min*., 4, 1796).

Distribution et nomenclature des substances dont l'étude est du ressort de la géologie (*Journ. Min*., 6, 1797).

Sur la mesure des angles des cristaux (*Ann. Min*. [1], 3, 1818).

Sur le pyroxène analogique (*Ann. Min.* [1], 4, 1819).

Sur la structure des cristaux de mercure sulfuré (*Ann chim. phys.*, 8, 1818).

Sur l'électricité des minéraux (*Ann. chim. phys*., 8, 1818).

[The following are not in Royal Society list.]

Obs. sur les schôrl (*Mém. de la Académie royal des science*, 1784).

Sur la structure des cristaux de feldspath (*Mem. Par*., 1785).

Sur la structure de diverses cristaux metalliques (*Mem. Par*., 1785). 

Sur la structure du cristal de roche (*Mem.
Par.*,
1786).

 Sur la structure des cristaux de schôrl (*Mem. Par*., 1787). 

Mémoire oû l'on expose les problèmes relatifs à
la structure des cristaux  *(Mem. Par*., 1788). 

Sur la double réfraction du spath d'islande (*Mem.
Par.*, 1788). 

Sur la manière
de ramener à la théorie du parallélipipède celle de toutes les autres formes primitives des cristaux (*Mem. Par.*,
1789).

 Sur la structure des cristaux de grenat (*Journ. phys.*, 19, 1782).. . 

Sur la structure des spaths calcaires
(*Journ. phys*., 20, 1782). 

Sur le spath adamantine (*Journ. Phys*., 30, 1787). 

De l'électricité du spath boracique
(*Journ. Phys*., 38, 1791). 

Sur les aimants naturels (*Journ. phys*., 45, 1794).

BIOGRAPHIES OF HAÜY

Cuvier, Georges, baron. Éloge historique de M. Haüy, prononcé dans la séance publique de l'Académie royale des
sciences, le 2 juin 1823. (*Académie royale des sciences de l'Institut de France*, 8, pp. cxliv-clxxviii, Paris, 1829).

----- Biographical notice of Haüy. (*Am. J. Sci*. [1], 8, 362-371. 1824). Translated and abridged by John Griscom.

----- Memoir of Haüy. Translated for the Smithsonian Institution by C. A. Alexander. (*Smiths. Inst. Ann. Rept.*, 1860,
376-392. 1861).

----- Éloge historique de René-Just Haüy. (In his:  *Éloges historiques*, 247-283, Paris [1860]).

Delafosse et Durozoir. L'Abbé Réné-Just Haüy. (In:  *Biographie universelle, ancienne et moderne*. Supplement, 66,
551-563, Paris, 1839).

PORTRAITS OF HAÜY

Biographie nouvelle des contemporains. Paris, 1823. 9, pl. opp. p. 74. Bust port., with star of Legion of Honor. In
outline.

Cap, Le Museum d'Histoire Naturelle. Paris, 1854. Pl. Opp. p. 71. Head, facing left. From medal. Engr. by A. Feart.

Lucas, Tableau methodique des espèces minérales. Part 1. Paris, 1806. Frontispiece. Facing left and partly front.
Portrayed in the act of measuring the angle of a crystal of calcite. "Traduit du dessin de F. Massard."

Leonhard, Taschenbuch für die gesammte Mineralogie. 3. Frankfurt am Main, 1809. Frontispiece. Evidently copied from the
frontispiece in Lucas, without the crystal.

Société Montyon et Franklin. Portraits et histoire des hommes utiles. Paris,

1833-41. In v. 2, pl. 47.[Not seen; not in New York Publ. Libr.]

NOTES

1 At the date of Haüy's birth his native town formed part of the old province Ile de France, the department of the
Oise not having yet been constituted.

2 These two colleges formed part of the complex constituting the Université. The Collège de Navarre dated from 1304,
when is was founded by Jeanne de Navarre, wife of Philippe le
Bel; among its pupils had been the Due d'Anjou (Henri III), Henri de Navarre (Henry IV),
Armand Duplessis (later the famous Cardinal Richelieu) and Bossuet. The Collège du Cardinal Lemoine was of even earlier
foundation, since it was established in 1297 by Cardinal Jean Lemoine.

3 The first idea of the Jardin du Roi has been attributed to the physician of Marie de'Medici, Jean Rolin, who in his
travels had visited the recently established botanical gardens in Germany and Italy.

4  *Traité de Minéralogie*, vol. I, pp. 23, 24, 1801.

5 Defrance du Croisset, a financier (mâitre de comptes), who owned fine collections of shells and minerals, which he
was always ready to show to savants.

6 Baron Georges Cuvier, "Éloges historiques," Paris, in "Éloge historique de René-Just Haüy," p.
261, 1860.

7 In plate 9, as well as in the frontispiece, Haüy is shown wearing the jacket of the Academy.

8 In the frontispiece Haüy is shown wearing the medal indicating this rank.

9 Author of  *Traité de cristallographie géometrique et physique*, Paris, 1879-1884; 2 vole. and atlas.

10 Cited in  *La Science Française*, Exposition Universelle de San Francisco, vol. 1, p. 170.

11 "De solido intra solidum naturaliter contento," Florence, 1669; Engl transl. 1761; see also  *American
Mineralogist*, 2, 33, 1917.

12 Franz von Kobell,  *Geschichte der Mineralogie von 1650-1860*, pp. 188, 191, 1864.

13 Paris, 1809; 312 pp.; 4 plates.

14  *Tableau Comparatif*, introduction, p. 1.

15  *Op. cit.*, p. xvii.

16 Paris, 1817; xvi, xxii and 253 pp., 3 plates.

17  *Bull. N. Y. Public Library*, 22 (3), p. 204, March, 1918.

---

Copyright © 1918
- 2004
Mineralogical Society of America. All
rights reserved
