# Supplementary material for: Space groups and crystallographic symmetry: writing a multi-featured tutorial in a new style
Source: Acta Crystallogr E Crystallogr Commun. 2021 Jul 16;77(Pt 9):857–63. doi: 10.1107/S2056989021007039 (PMC8423017; doi:10.1107/S2056989021007039)
Supplement: Supplementary file 1 [file e-77-00857-sup2.zip › symandsg/Main/hauyvii.htm]

Hauy, father of crystallography

|  |  |  |  |  |  |  |  |
| --- | --- | --- | --- | --- | --- | --- | --- |
| **Home** | **AmMin** | **GMR** | **RiMG** | **Collectors Corner** | **Directory** | **Short Courses** | **Contacts** |
| --- | | | | | | | |

---

*Volume 3, pages 131-132,
1918*

HAÜY, THE "FATHER OF CRYSTALLOGRAPHY"

FRANK D. ADAMS 

*McGill University*

    
IT is very fitting that the student in any branch of knowledge, and
particularly in Natural Science, should pause, on
occasion, to glance back at the history of the development of his
subject. Nothing can be more instructive than to trace
anew the progress that has been made thruout the centuries, from the
very crudest beginnings, a progress as sure and
irresistible as the oncoming tide, now gradual, and now, like the
onrush of a seventh wave, going forward more rapidly,
borne on by the stimulus of some exceptionally brilliant mind. To
re-study the problems which have from time to time
presented themselves, and to observe with what ingenuity they have been
attacked and overcome must needs afford the
greatest possible incentive and encouragement to the present-day
student in dealing with the problems which still await
solution.

    
Crystallography forms no exception to this generality, and altho
comparatively few years have elapsed since its firm
establishment as an exact science, the page of its history is
emblazoned with the names of scores of eminent men, from
the times of Nicolaus Steno and of Romé de l'Isle, who laid the first
foundation, up to the present-day.
Unquestionably, however, the first solid pillar in the structure of the
science was set up by the Abbé Haüy, professor
of the humanities at the University of Paris, during the last decades
of the eighteenth century. He it was who, by
careful observation and research, followed by clear deductive
reasoning, first brought order out of chaos and raised the
study of crystals to the dignity of a science. In his own words; "*un coup d'oeil peu attentif, jété sur les
cristaux, les fit appeler d'abord de purs* JEUX DE LA NATURE,  *ce qui n'étoit qu'une manière plus élégante de faire
l'aveu de son ignorance. Un examen réfléchi nous y découvre des lois d'arrangement, à l'aide desquelles le calcul
représente et enchâine l'un à l'autre les résultats observés; lois si variables et en même temps si précises et
si régulières; ordinairement très simples, sans rien perdre de leur fécondité!*  " The fact that the theory of
crystal structure elaborated by Haüy, and based on his discourses of these laws of symmetry, of rational intercepts,
and of constancy of crystalline form, does not differ very materially, in its essential points, from the views
now prevailing, is a remarkable tribute to his genius, and will forever render the name of Haüy famous as the
"Father of Crystallography." This proud title is most appropriate and has been bestowed upon Haüy with
reason. If any support were needed, it is only necessary to recall the testimony of Henry James Brooke, in his
"Familiar Introduction to Crystallography," published in 1823 a few years after Haüy's death, to the effect
that "The Abbey Haüy's works on crystallography are the only ones in which a truly scientific exposition of the
theory of crystals is to be found." His work has afforded the key wherewith it has been possible for his successors
to unlock many of the secrets of crystal structure, and the great strides which the science has made during the past
century have all had, as their starting point, the discoveries and theories of Haüy. It is especially fitting that now,
on the one hundred and seventy-fifth anniversary of his birth, crystallographers thruout the world should unite in
paying homage to the memory of this distinguished scientist, and should be reminded afresh of the extent to which the
science of crystallography is indebted to his brilliant pioneer work.

---

Copyright © 1918
- 2004
Mineralogical Society of America. All
rights reserved
