# Supplementary material for: Space groups and crystallographic symmetry: writing a multi-featured tutorial in a new style
Source: Acta Crystallogr E Crystallogr Commun. 2021 Jul 16;77(Pt 9):857–63. doi: 10.1107/S2056989021007039 (PMC8423017; doi:10.1107/S2056989021007039)
Supplement: Supplementary file 1 [file e-77-00857-sup2.zip › symandsg/Main/Hermann.htm]

Carl Hermann

# Carl Hermann (17.6.1898 – 12.9.1961)

Die Universität
verliert in Carl Hermann einen Forscher von internationalem Rang und einen
Lehrer, der durchdrungen war von der Aufgabe, sein umfassendes Wissen und mehr
noch die von ihm selbst erarbeiteten Erkenntnisse an seine Schüler
weiterzugeben. Carl Hermann entstammt sowohl väterlicher- wie
mütterlicherseits bis in die Reformationszeit zurückreichenden alten
Pfarrerfamilien. Er wurde am 17. 6. 1898 in Wesermünde geboren. Seine akademische
Ausbildung prägte die große Forscherpersönlichkeit des Physikers Max Born, bei
dem er 1923 in Göttingen promovierte, ungefähr gleichzeitig und in vielfachem
Studienkontakt mit Werner Heisenberg. Das Göttingen jener Jahre gewährte in
jeder Hinsicht die befruchtende Atmosphäre, die geeignet war, in einem
angehenden Mathematiker Begeisterung für die theoretische Physik zu wecken.
Entscheidend für die Weiterentwicklung des jungen Wissenschaftlers wurden die
Assistentenjahre in Stuttgart bei P. P. Ewald, von 1925 bis 1935. Sie brachten
die erste nähere Berührung mit der nach der Laue'schen Entdeckung der
Röntgenstrahl-Interferenzen in mächtigem Aufschwung befindlichen
Kristallstrukturforschung. In Stuttgart habilitierte er sich im Jahr 1931, und
dort entstand die nach ganz neuen Gesichtspunkten hergeleitete Systematik,
„Genealogie“ und Nomenklatur für die „Raumgruppen“, die 230 verschiedenen
Möglichkeiten für periodische Symmetrieanordnung von Bauelementen im Raum.
Diese Nomenklatur wird für alle Zeit mit seinem Namen verknüpft sein und ist
jetzt bei den Strukturforschern der ganzen Welt in ausschließlichem Gebrauch.
Eine weitere Frucht der Stuttgarter Jahre ist der zusammen mit Ewald initiierte
„Strukturbericht“, eine heute zu einem unentbehrlichen Hilfsmittel aller sich
mit dem festen Körper befassenden Wissenschaft gewordene, inzwischen zu einem
vielbändigen Nachschlagewerk angewachsene Darstellung und Beschreibung aller
erforschten Strukturen.

Das „Dritte
Reich“ machte 1935 dem weiteren Verbleib Carl Hermanns an der Hochschule ein
Ende, da sich das kompromißlose Wesen des Verstorbenen der in zunehmendem Maß
den Hochschullehrern abgeforderten, „politischen Betätigung“ und dem
politischen „Bekenntnis“ versagen mußte. Er nahm - wohl als erster
„Theoretiker“ der Industrie - eine Stellung als Physiker bei der
IG-Farbenindustrie Ludwigshafen an. Dort entstand eine seinen Na­men wiederum
in alle Welt tragende Veröffentlichungsreihe, zusammen mit Brill, Grimm und
Peters, über den Feinbau aller kristallinen Materie und damit über die verschiedenen
Arten und Möglichkeiten chemischer Bindung. Doch auch dort sollte ihn der
Terror erreichen, ausgelöst allerdings durch ihn selbst, durch zwei seiner
hervorstechendsten Eigenschaften, seine unbegrenzte Hilfsbereitschaft gegenüber
allen Menschen und seine gelassene Furchtlosigkeit für die eigene Person:
Nachdem er zahllosen jüdischen Mitbürgern Schutz vor der Deportation gewährt
und vielen zur Flucht verholfen hatte, wurde er verhaftet und zu langjähriger
Freiheitsstrafe verurteilt. Befreiung und Rehabilitierung brachte erst das
Kriegsende.

Nach kurzem
Wirken als Dozent an der TH Darmstadt erreichte ihn 1947 die Berufung unserer
Universität auf den neugeschaffenen Lehrstuhl für Kristallographie, den er bis
zuletzt innehatte. In dem zugleich eingerichteten Kristallographischen Institut
reifte unter seiner Leitung eine Reihe von weiteren vielbeachteten
Untersuchungsreihen. Im theoretischen Bereich drang das phänomenale räumliche
Vorstellungsvermögen des Verstorbenen hinaus über den vertrauten dreidimensionalen
Raum zu den Symmetrie-Möglichkeiten in Räumen aller Dimensionen.

Carl Hermann war
ein Mensch von ganz seltener, absoluter Lauterkeit der Gesinnung, von einer
kein Opfer scheuenden Güte für jedermann. Seit Jahren gehörte er dem
Disziplinargericht der Universität an, und stets war er es, der für alles
Versagen der Betroffenen verstehende und entschuldigende Worte, sowie Gründe
für eine Milderung der Urteile suchte.

Alle
Liebhabereien, die Carl Hermann betrieb, trugen einen Zug von Genialität und
erweckten oft den Eindruck, als könnten sie, wenn bevorzugt, ohne weiteres zur
Haupt-Berufung werden. So war er sprachlich und philologisch hochbegabt,
lernte z. B. spielend innerhalb kürzester Zeiten jeweils eine ihn gerade
reizende neue Sprache bis zur Konversationsreife.

Seinem humanitären Denken
und seiner religiösen Überzeugung, die sich im Dritten Reich so glänzend
bewährt hatten, blieb er auch nach dem Zusammenbruch treu: Bei den Quäkern und
in der christlichen Friedensbewegung war er stets aktiv tätig. In ständigem
Kontakt mit ihren führenden Männern, gleichgültig welcher theologischen
Richtung, war ein großer Teil seines Denkens dem Ringen um die Erhaltung des
Weltfriedens gewidmet.

Die Welt ist um
einen bedeutenden Gelehrten und wahrhaft guten Menschen ärmer. Kollegen,
Schülern, Freunden und Bekannten wird sein Andenken in hohen Ehren bleiben.

*Nachruf des Rektors der Philipps-Universität, Marburg, 18.9.1961*

  

**Prof. Carl Hermann**, director of the Crystallographic Institute at the
University of Marburg
died unexpectedly in his sleep on September 12 at the age of sixty-three. He
was a pupil of Max Born and a fellow research student with Werner Heisenberg,
at Göttingen. Later he became a lecturer at Stuttgart, where he and Paul Ewald
initiated the *Strukturberichte* which (with its successor *Structure
Reports*) gives a detailed abstract of every known crystal
structure determination, and which is a valued work of reference in every
modern scientific library. He also developed, with Prof. C. Mauguin, a systematic
nomenclature (now known as the Hermann-Mauguin notation) for the 230 space
groups, from which the symmetry can be derived at a glance; and he travelled
widely in the early 1930's, gathering material for the
two volumes of the *Internationale Tabellen zur Bestimmung von
Kristallstrukturen.*

Hermann and his wife Eva were guests
in my home at the time when the National Socialist party doubled its strength
in the General Election of June 1932, which later brought Hitler to
power; and I can well remember their dismay, for they realized even then what
would follow. Later, Carl Hermann refused to conform to the political
conditions imposed on academic staff and he took a position as physicist at the
I.G. Farbenwerke at Ludwigshafen, where he continued crystallographic research
of high quality on the nature of chemical bonds and on symmetry in *n*-dimensional
space. He and his wife were imprisoned throughout much of the Second
World War
for their increasing activities in hiding and helping innumerable Jews
to escape
from the terrors of Nazi persecution. Only his eminence as a scientist
and the intervention of his friends saved him from the carrying out of
a death sentence; but these years left their
mark on him.

In 1947 he was appointed to the
new chair of crystallography in the University of Marburg and he threw himself
with great energy into the teaching of the subject and into the building-up of
an active research school. But he also gave much of his time to the work of the
Society of Friends, of which he and his wife were members, and to efforts to
promote international understanding. His presence at con­ferences was the more
valued because of his great gift for languages; if a new language interested
him, he learnt it.

The statement
put out by the Rector and Senate of the University of Marburg says of him:
"Carl Hermann was a man of absolute integrity, and of kindness to others
which had no limit. For many years he was a member of the Disciplinary Court of
the University and it was always he who tried to understand and to excuse the
defendant . . .".

He is not likely to be forgotten, for he has left many friends.

*Nachruf von
Kathleen Lonsdale in Nature **192**, 1961*

  

|  |  |  |
| --- | --- | --- |
| *Mathematische Kristallographie in Marburg* | *Elke Koch* | *Werner Fischer* |

*Stand: Mai 2001*
