# Supplementary material for: Space groups and crystallographic symmetry: writing a multi-featured tutorial in a new style
Source: Acta Crystallogr E Crystallogr Commun. 2021 Jul 16;77(Pt 9):857–63. doi: 10.1107/S2056989021007039 (PMC8423017; doi:10.1107/S2056989021007039)
Supplement: Supplementary file 1 [file e-77-00857-sup2.zip › symandsg/Main/his.schomaker.html]

(IUCr-Crystallographers Online) Verner Schomaker 1914-1997

# Crystallographers Online

---


## Verner Schomaker 1914-1997

 Verner Schomaker, possessor of one of the most critical and
wide-ranging scientific intellects of our time, died in Pasadena,
California, on March 30, 1997, of pancreatic cancer.
What follows is a very personal account. Many of those who worked with
or were closely exposed to Verner might have written something similar;
I am confident that the general flavor would have been the same.

Every scientific question seemed to interest Verner, and anyone with a knotty problem was
welcome at his always-open door. And his time was always yours -- until he, at least,
understood in some depth what you were asking, and preferably you did too. The answer
did not, of course, always come in one session -- even though the sessions could last
for many hours, past meal times and past other appointments that you forgot about because
you were so engrossed. His memory was prodigious, and when he encountered a problem
he 'worried it', like a dog with a bone. He might not have all the insight he wanted
when the question was first raised, or even during the next few days or weeks -- but he
wouldn't forget. You might encounter him some years later, and he'd say: "I've been
thinking about what you said, and ....".

He was at once friendly, open, uncommonly generous, and extremely bright.
He was, to those who were privileged to work with him or otherwise benefit from his
insights, simply without peer as a one-on-one teacher. In the 40's and '50's, many
who worked with him felt complimented when he would say "How can you be so god-damned
stupid?", since we realized that he expected us to understand and that, frustrated
though he might be with our slowness, he would not give up until we understood, or
left. In his later years, he learned patience and mellowed somewhat, and those who
couldn't follow an abstruse line of reasoning he was explaining might be asked "What
do I have to do, say it louder?" But never, and I do mean **never**, was there any
animosity involved in what might seem to some to be harsh remarks. Nor did Verner's
own ego ever intrude. He was selfless, far more so than almost anyone imaginable with
his level of intellect and accomplishment. He was interested in getting things **right**,
not in who got the credit, and was never afraid to admit his errors and his own
limitations, although he overestimated them (as he, generously, did the abilities of
some of his collaborators).

He is best known for his contributions in electron and X-ray diffraction.
He thought that his most important contribution had been in the early days of electron
diffraction, for development of techniques for the visual interpretation of the
scattering of electrons by gas molecules. None of the structures reported from his
productive group had later to be revised, when sector methods gave greater resolution
and precision. But he published in many other fields as well -- one of his final
papers (with J. Waser) was on the "Global Thermodynamics of Systems That Include
Stressed Solids", and he was saddened that he could not interest any colleague in
studying it intently enough to discuss it with him meaningfully. At least one of his
papers became a "Citation Classic" in the Science Citation Index. However, his total
publication list probably didn't reach 200 papers, because he was a perfectionist when
writing a paper, and because he was so readily distracted by the intriguing problems
presented by those who sought him out. His generous spirit, his penetrating intellect,
his breadth of interests and curiosity, and his selflessness, led almost everyone
within his orbit to use him as a consultant. There is little doubt that if there
were a "Science Advisor Acknowledgment Index", he would have ranked at or very near the
top. It has been estimated that during the 1940s and '50s, at least one third, and
perhaps as many as one half, of the papers published by the "Gates and Crellin
Laboratories" (the Caltech Division of Chemistry) concluded with a phrase such as
"We are grateful to Professor Verner Schomaker for helpful discussions", or
"The valuable insights provided by our colleague Verner Schomaker helped to make this
work possible". And these papers covered the gamut of work in the Division, not just
in diffraction, but in quantum mechanics, immunochemistry, nmr, spectroscopy,
thermodynamics, inorganic and organic chemistry, ... In those years, the reference
"V. Schomaker, unpublished" was so common (in others' papers especially) that it could
be recognized by the astute as what Patterson meant by "Vernished" in his famous spoof
of the Acta Crystallographica telegraphic style\*.

A native of Nebraska, where he grew up on a farm, he earned a BS from that
state's University in 1934 and a MS in 1935. He then moved to Pasadena, where Pauling
quickly recognized his uncommon qualities. After receiving a PhD in 1938, he went up
the academic ladder in Chemistry at Caltech (taking time out for war-time research from
1942 to 1945). He received the ACS Award in Pure Chemistry in 1949, and served as ACA
President in 1961-62. In 1958 he left academic work to join the Union Carbide Research
Institute (just north of New York City), where he spent seven years -- but when it became
apparent that the initial promise of something modelled on the Bell Labs or what was
then the Shell Development Laboratory was never going to materialize, he joined the
faculty of the Department of Chemistry at the University of Washington in Seattle,
serving initially for five years as Chair, during an important time for faculty growth.
He became Professor Emeritus in 1984. After his retirement, he was also a Faculty
Associate at Caltech, dividing his time about equally between Pasadena and Seattle.

He is survived by his wife Judy, his sisters Helen and Mariana, his sons David,
Eric, and Peter, and eight grandchildren. His family has requested that donations in
his memory be made to the Verner Schomaker Memorial Fund, c/o California Institute of
Technology, Office of Donor Relations, Mail Code 105-40, Pasadena, CA 91125.
The fund will be used to support student research.

```
					Kenneth Trueblood
```

\* The complete text of this 21-line paper by "A. L. Pon" is
reproduced in "Patterson and Pattersons", edited by Jenny Glusker,
Betty Patterson, and Miriam Rossi, Oxford University Press (1987), p.
618

---

25th July 1997 - Crystallographers Online - **Copyright � International Union of Crystallography**
