# Supplementary material for: Space groups and crystallographic symmetry: writing a multi-featured tutorial in a new style
Source: Acta Crystallogr E Crystallogr Commun. 2021 Jul 16;77(Pt 9):857–63. doi: 10.1107/S2056989021007039 (PMC8423017; doi:10.1107/S2056989021007039)
Supplement: Supplementary file 1 [file e-77-00857-sup2.zip › symandsg/Main/Hodgkin.html]

CWP at physics.UCLA.edu // Hodgkin

|  |  |
| --- | --- |
| **Welcome to CWP at UCLA** |  |

**86 Eminent Physicists** 

**Search the Archive**

**Fascinating Documents**

**Annotated
Photo
Gallery**

**In
Her Own Words**

**Some Physics History**

**500+ Books and Articles**

**Project Staff**

**Field Editors**

**Project Support**

**Photo Credits**

**Copyright Notice**

#### Crystallography

|  |  |  |
| --- | --- | --- |
| Contributions  ---  Publications  ---  Honors  --- |
 Dorothy Crowfoot Hodgkin   1910-1994 | Jobs/Positions  ---  Education  ---  Additional Information  --- |

### Some Important Contributions

In 1934, with J. D. Bernal in Cambridge, photographed for the first
time single crystals of a protein - pepsin.

**First to determine the three-dimensional structure of a complex bio-organic molecule.**
She
determined the structure of cholesteryl iodide by x-ray diffraction in
1941-42 (published in 1945) in complete three-dimensional detail, at a
time when no one else was determining complex structures in three
dimensions because of the formidable calculations involved.

**Determined the structure of penicillin**  in 1944 (published in
1949), again in three-dimensional detail. Before her work there was
only fragmentary and conflicting evidence on the structure, from
chemical analysis, of this rather unstable molecule, which was of
immense importance as an antibiotic during and immediately after World
War II.

**Determined the structure of vitamin B-12** in 1956, using one of
the first high-speed digital computers. This was by far the most
complex molecule whose three-dimensional architecture had been
established, and some of its unusual structural features were quite
unanticipated.

**Determined the structure of insulin**  in 1969. This culminated a study pursued
over three decades. The details of the structure provided insight into the function of this vital hormone.

### Some Important Publications

**Speech on receiving the Nobel Prize in Chemistry:**  
"The X-ray Analysis of Complicated Molecules," *Science* 150:
979 (1965).

"The Crystal Structure of Cholesteryl Iodide," *Proceedings of the Royal Society* A184: 64 (1945), with C.H. Carlisle.

"X-ray Crystallographic Investigation of the Structure of Penicillin," in  *Chemistry of Penicillin*, Princeton University Press, Princeton, NJ 1949, with C. Bunn, B. Rogers-Low, and A. Turner-Jones.

"The Crystal Structure of the Hexacarboxylic Acid Derived from B-12 and the Molecular Structure of the Vitamin," *Nature* 176: 325 (1955), with J. Pickworth, J. Robertson, K. Trueblood, R. Prosen, and J. White.

"Structure of Vitamin B-12," *Nature* 173: 64 (1956), with J. Kamper, M. Mackay, J. Pickworth, K. Trueblood, and J. White.

"Structure of Rhombohedral 2-zinc Insulin Crystals," *Nature* 224: 491 (1969), with M. Adams, T. Blundell, E. Dodson, G. Dodson, M. Vijayan, E. Baker, M. Harding, B. Rimmer, and S. Sheat.

"Atomic Positions in Rhombohedral 2-zinc Insulin Crystals," *Nature* 231: 506 (1971), with T. Blundell, J. Cutfield, S. Cutfield, E. Dodson, G. Dodson, D. Mercola, and M. Vijayan.  


### Honors

**The Nobel Prize** in Chemistry, 1964 - "For her determinations by X-ray techniques of the structures of important biological substances."

Chancellor, Bristol University 1970 - 1988

Fellow of The Royal Society (London)

Order of Merit (U.K.) 1965

Fellow, Indian Academy of Sciences

Bakerian Lecturer, National Academy of Sciences (U.S.) 1972

**Some other prizes and medals:**

Royal Medal, The Royal Society of London 1956  
Copley Medal, The Royal Society of London 1976  
Longstaff Medal, British Association for the Advancement of Science 1978  
Mikhail Lomonosov Gold Medal, Soviet Academy of Sciences 1982  
Dimitrov Prize 1984  
Lenin Peace Prize 1987

**Honorary D.Sc.:** Universities of Bath, Brown, Cambridge,
Chicago, Delhi, East Anglia, Exeter, Ghana, Harvard, Hull, Kent, Leeds,
London, Manchester, Mount Sinai, Oxford, St. Andrews, Sussex, Warwick.

#### Other honorary degrees:

Dr. Medicine and Surgery (Modena); D.Univ. (Open, York, Zagreb);
LL.D. (Bristol);
D.L. (Dalhousie).

**Foreign Member:** Royal Netherlands Acad. of Science and Letters, American Acad. of Arts and Sciences, Bavarian Acad.,
Austrian Acad.,
Ghana Acad. of Sciences, Puerto Rico Acad. of Sciences,
Australian Acad. of Sciences,
Leopoldina Acad. of Sciences, Norwegian Acad. of Sciences,
Indian Acad. of Sciences, Royal Irish Acad. of Sciences,
National Acad. of Sciences (U.S.A.) .

**Honorary Member:** USSR Academy of Sciences, Royal Institution of Great Britain.

**Honorary Fellow:** Somerville College, Oxford; Linacre
College, Oxford; Girton College, Cambridge; Newnham College,
Cambridge.
; Bristol University (1988-1994).

### Jobs/Positions

1934-35 Research Fellow, Somerville College, Oxford   
1935-55 Official Fellow and Tutor, Somerville College, Oxford   
1946-56 Demonstrator and Lecturer, Oxford University  
1947-48 Rockefeller Fellowship   
1956-60 University Reader, Oxford University  
1960-77 Wolfson Research Professor, Oxford University  
1956-77 Professorial Fellow, Somerville College, Oxford   
1977-83 Fellow, Wolfson College, Oxford   
1977-94 Professor Emeritus, Oxford University

**President, International Union of Crystallography 1972-75.  
President, British Association for the Advancement of Science 1977-78.  
President, Pugwash Conference on Science and World Affairs 1975-88.**


### Education

B.A. Somerville College, Oxford University 1931  
D.Phil. Cambridge University 1937

### References consulted

[iss1970kl], [ssmbi1981mp], [qrb1994mp], [iww1994], [nsb1994lj], [cww1994mv], [1F N20], [1982 JCE], [N 1994mp]

### Additional Information/Comments

"I felt embarrassed when I was awarded the Nobel Prize before Dorothy,
whose great discoveries had been made with such fantastic skill and
chemical insight, and had preceded my own." ---- Max Perutz in *Structural Studies of Molecules of
Biological Interest*, G. Dodson, J.P.Glusker, D. Sayre, eds.

In 1934, just the first single crystals of a protein - pepsin -
were found and photographed, she suffered onset of
a severe case of rheumatoid arthritis that progressively
crippled her throughout the rest of her life.

During WWII, she was primarily occupied with the structure analysis
of penicillin, the first of the antibiotics, which played an
important role in the treatment of war wounded.

"She pursued her crystallographic studies, not for the sake of honors,
but because that was what she liked to do. There was a magic about her
person. She had no enemies, not even among those
whose scientific theories she demolished or whose political views
she opposed. Just as her X-ray cameras bared the intrinsic beauty
beneath the rough surface of things, so the warmth and gentleness
of her approach to people uncovered in everyone, even the most hardened
scientific crook, some hidden kernel of goodness." ----Max Perutz,
obituary in *The Independent*.

A remembrance of Max Perutz - "There are certain letters which I
dread to open," she once told me, " and when I saw one from
Buckingham Palace I left it sealed, fearing that they wanted to make me
Dame Dorothy." It would have made her feel like a femme formidable,
which she happily was not. She was relieved to find that the Queen
offered her the Order of Merit, a much greater honor. ----ibid.

She was only the second woman to receive the Order of Merit; the
first was Florence Nightingale. There are just 25 living persons in the
Order of Merit at any given time. In 1992, Dorothy Hodgkin was the
senior member and
Margaret Thatcher, the former Prime Minister (who had been a student of
Dorothy's in Somerville College, Oxford), was the most junior, i.e.
most
recent, member.

"In her life she was a constant source of inspiration and help to those
around her. In particular, through her work at Somerville she has left a legacy
of distinguished women scientists around the world, that includes Pauline
Harrison, Jenny Glusker, Marjorie Harding, Margaret Adams, Eleanor Dodson,
Judith Howard and Carol Huber, among others." ----Professor Louise Johnson,
University of Oxford.

She was an active participant in the  Pugwash
Conferences on Science and World Affairs founded in response to the
Russell-Einstein
Manifesto issued by Bertrand Russell and Albert Einstein, which called upon
scientists of all political persuasions to assemble to discuss the threat posed
to civilization by the advent of thermonuclear weapons.

She was President of Pugwash from 1975 - 1988.

She was married to Thomas Hodgkin, and had three children Luke, Elizabeth, and
Toby.

#### Further Reading

The biography by Georgina Ferry, *Dorothy Hodgkin: A Life*, Granta 1998.
  
The Times(London) Obituary

#### Links to other internet sites

Memorial Address by M. F. Perutz 

Dr. Dorothy Crowfoot Hodgkin:
Chemist, Crystallographer, Humanitarian  - a biography by Linda Cohen from The Nobel Prize Internet Archive.

The Papers of Dorothy Mary Crowfoot Hodgkin

---


#### Field Editor: Professor Kenneth Trueblood

  

 Copyright � CWP and Regents of the University of California 1997-98.

***To cite this citation:***  
" Hodgkin, Dorothy Crowfoot." *CWP*   
< http://www.physics.ucla.edu/~cwp>

**BACK TO THE TOP**


---


3/16/98 nb & 5/22/00 nb
