# Supplementary material for: Space groups and crystallographic symmetry: writing a multi-featured tutorial in a new style
Source: Acta Crystallogr E Crystallogr Commun. 2021 Jul 16;77(Pt 9):857–63. doi: 10.1107/S2056989021007039 (PMC8423017; doi:10.1107/S2056989021007039)
Supplement: Supplementary file 1 [file e-77-00857-sup2.zip › symandsg/Main/hwu.htm]

Chemistry Faculty @ Clemson :: Hwu

|  |  |
| --- | --- |
|  |  |
|  |  |
|  |  |
|  |  |
|  |  |
|  |  |
|  |  |
|  |  |
|  |  |
|  |  |
|  |  |

Chemistry
Faculty

|  |  |  |
| --- | --- | --- |
| Shiou-Jyh Hwu, Professor | | |
| Inorganic Chemistry | | |
|  | | |
| **Phone:** | (864) 656-5031 | |
| **Office:** | 477 Hunter Laboratories | |
| **E-mail:** | shwu@clemson.edu | |

|  |  |  |
| --- | --- | --- |
| Research | Research Groups | Recent Publications |

Dr.
Hwu received his B.S. (1978) in chemistry from Fu-Jen Catholic University
in Taipei, Taiwan and his Ph.D. (1985) in inorganic chemistry from
Iowa State University. His dissertation work focused on solid-state,
condensed-cluster chemistry under the guidance of Prof. John D.
Corbett. He conducted postdoctoral research (1985-88) in materials
chemistry and superconductivity at Northwestern University with
Prof. Kenneth R. Poeppelmeier. Dr. Hwu served on the faculty at
Rice University (1988-1995) before coming to Clemson in 1995.

Research  
  
One project deals with the synthesis of solids containing confined  
transition-metal-oxide frameworks. The primary objective is to survey
new solids that are of fundamental importance for experimental and
theoretical studies of spin interactions. Current research also
involves the study of solids as molecular magnets for device applications.  
  
The second project studies electrochemical synthesis of conducting
solids in nonaqueous media. The goal is to develop a highly efficient,
bench-top synthetic method that can facilitate a controlled synthesis,
with a possibility by design, of solid state materials. The scope
is to explore novel solids possessing unusual electronic properties,
including superconductivity.  
  
The third area of materials research is on the synthesis of a new
class of hybrid materials via salt inclusion. The resulting solids
exhibit a composite framework of mixed covalent and ionic lattices.
The current interest is to seek parameters that govern the formation
of nanometer-sized transition metal (TM) oxide lattices of desired
size, geometry and shape.  
  
back to top

Recent
Publications  
  
1. King, T.C., Y.K. Kuo, M.J. Skove, and S.J. Hwu, Vacancy ordering
transitions in one-dimensional lattice gas with Coulomb interactions.
*Physical Review B*, **2001**. 6304(4): p. art.
no.-045405.  
  
2. Huang, Q., S.J. Hwu, and X.H. Mo, High-temperature synthesis
of an open-framework compound, Na2Cs2Cu3(P2O7)(2)Cl-2(CU-4), by
molten-salt methods. *Angewandte Chemie-International Edition,*
**2001**. 40(9): p. 1690-1693.  
  
3. Huang, Q. and S.J. Hwu, High-temperature synthesis of two new
aluminophosphates, NaRb2Al2(PO4)(3) and NaCs2Al(PO4)(2), in molten
alkali metal chloride media. *Chemistry of Materials*, **2001**.
13(5): p. 1794-1799.  
  
4. Schujman, S.B., G.S. Nolas, R.A. Young, C. Lind, A.P. Wilkinson,
G.A. Slack, R. Patschke, M.G. Kanatzidis, M. Ulutagay, and S.J.
Hwu, Structural analysis of Sr8Ga16Ge30 clathrate compound. *Journal
of Applied Physics*, **2000**. 87(3): p. 1529-1533.  
  
back to top

Site last updated 10.25.2005  
Site maintained by Chemistry  
Site Created by
