# Supplementary material for: Space groups and crystallographic symmetry: writing a multi-featured tutorial in a new style
Source: Acta Crystallogr E Crystallogr Commun. 2021 Jul 16;77(Pt 9):857–63. doi: 10.1107/S2056989021007039 (PMC8423017; doi:10.1107/S2056989021007039)
Supplement: Supplementary file 1 [file e-77-00857-sup2.zip › symandsg/Main/ibasic_d.html]

Interactive guide to diffraction ..

|  |  |
| --- | --- |
| **Interactive Tutorial about Diffraction**  **Interactive examples: Ewald sphere** | |
| **Basic examples**  Single atom  Pair of atoms  Row of atoms  **Interactive examples**  1D crystal builder  Polygons of atoms  Different atoms  *Ewald sphere*  Units  Interference  Bragg's law  **Goto**  Contents | This example shall illustrate the different 'scans' in reciprocal space and allows you to see the path a THETA-scan and a H-scan follows. All scattering patterns so far have been along H,K or L in reciprocal space. On a laboratory diffractometer, usually THETA scans are carried out The construction you are about to see is known to crystallographers as **Ewald sphere**.   ---         ---   Here are a few questions to explore:   - Recall the scattering vector diagram presented in the   lecture when discussing the case of two electrons. The   scattering process is illustrated on the right. - When executing this example you will see a plot that   shows the 2D scattering intensity in the *hk* plane.   The blue circle shows the   path for the THETA scan which can be seen as    blue graph. The thick green   line shows the scan DISCUS presented, again shown   as green graph. Both result from   the same basic pattern ! Just hit RUN and see for yourself .. - Now start changing the distance and orientation (within the   xy-plane) of the electron pair and describe their influence. - Finally investigate the effect of the wavelength of the   X-rays. Typical values used in laboratory sources are   Mo=0.71 A, Cu=1.54 A and Ag=0.55 A. |
| � Th. Proffen and R.B. Neder, 2003 | |
