# Supplementary material for: Space groups and crystallographic symmetry: writing a multi-featured tutorial in a new style
Source: Acta Crystallogr E Crystallogr Commun. 2021 Jul 16;77(Pt 9):857–63. doi: 10.1107/S2056989021007039 (PMC8423017; doi:10.1107/S2056989021007039)
Supplement: Supplementary file 1 [file e-77-00857-sup2.zip › symandsg/Main/interest1.htm]

Louis Pasteur

A site created and hosted by
David V. Cohn, Ph.D.,Emeritus Professor of Biochemistry, University of
Louisville

Jenner  
Landmark
Publications on Smallpox Vaccination   
  
Lady Mary Montagu on  
 inoculation  in
Yr. 1717   
  
Note on James
Phipps  
  
Pasteur  
Controversy with
Koch  
  
Report:Pouilly-le-Fort

First rabies treatment  
  

The Rumford Medal

Koch  
Controversy with  
Pasteur

Lister

Semmelweis  
  
Puepural Fever   
  
" Discovery" in his words  
  
Notes

Definitions

References

Search

Site Map

***Please examine links on left for detailed
documentation of Pasteur's career***

Louis Pasteur

***His scientific approaches, intuition and breadth of
accomplishment mark Louis Pasteur as a giant among scientists. The essay that follows is
the keynote address by Prof. Cohn for the Centennial Celebration of the death of Pasteur
that was sponsored jointly in 1996 at the University of Louisville by the University, the
Pasteur Institute of Paris, and the Alliance Fran�aise de Louisville. It has been revised
for presentation on this Web site.***

                                               **The Life and
Times of Louis Pasteur**                     
               
  

---

                                         
**Index,  Go to**  
                                       
  Education and Growing
Up                      
Diseases of Silkworms  
                                                       
Crystallography                                     
Germ Theory of Disease  
                                                       
Alcoholic
Fermentation                           
Anthrax  
                                                       
Spontaneous
Generation                         
Rabies

---

We need no reminder that the foundations of our knowledge of health
and disease were constructed by scientific giants who worked decades, even centuries, ago.
It  is with tributes such as the one today to Louis Pasteur that we pay homage to
these great minds -- to acknowledge their achievements and our indebtedness to them which
we can never repay.   
  
With certainty, one hallmark of Pasteur's research was not only the importance of his
individual discoveries, but the overwhelming breadth of his accomplishment.  
Pasteur's long time collaborator, Emile Duclaux, wrote, "A mind ... of a scientific
man is a bird on the wing; we see it only when it alights or when it takes flight. ... We
may by watching closely keep it in view, and point out just where it touches the earth.
But why does it alight here and not there? Why has it taken this direction and not that in
its flight toward new discoveries?"   
  
Pasteur, himself, provided us with an answer: He believed that his research was
"enchained" to an inescapable, forward moving logic. As we review today
Pasteur's scientific discoveries we shall see the truth of this statement: how one
discovery, one concept, led almost "inescapably" to another.  
  
  
**Education and Growing Up**  
Pasteur was born in Dole in 1822 and grew up in the nearby town of
Arbois, the only son of a poorly
educated tanner, Jean Pasteur. Louis was not an outstanding student during his years of
elementary education, preferring fishing and drawing to other subjects (Fig.1) In fact,
young Louis drawings suggested that he could easily have become a superior portrait
Artist. His later drawings of friends done at college were so professional that Pasteur
was listed in at least two compendia of XIX C. artists.

**Fig. 1.** Portrait of
Pasteur's father by young Louis.

The Senior Pasteur, however, did not see his son ending up as an
Artist, and Louis, himself, was showing increasing interest in chemistry and other
scientific subjects. The highest wish Father Pasteur had for his son was that he complete
his education in the local schools and become a professor in the college at Arbois. 
However the headmaster of the college recognized that Louis could do much better and
convinced father and son that Louis should try for the Ecole Normale Superieure in Paris.
This most prestigious French University was founded specifically to train outstanding
students for University careers in science and letters. And it was here that Pasteur
entered and began his long journey of scientific discovery.  
  
 **Crystallography**  
It may surprise some to learn that Pasteur, the father of microbiology and immunology, was
a chemist who launched his memorable scientific career by studying the shapes of organic
crystals. Pasteur was 26 years old, working for his doctorate in chemistry in the
laboratory of Antoine Balard. Crystallography was just emerging as a branch of chemistry.
His project was to crystallize a number of different compounds. Happily he started working
with tartaric acid. Crystals of this organic acid are present in large amounts in the
sediments of fermenting wine. Often one also found in the sediments in the wine barrels
crystals of a second acid called paratartaric acid or "racemic acid".  A
few years earlier, the chemical compositions of these two acids, tartaric and
paratartaric, had been determined. They were identical. But in solution there was a
striking difference. Whereas tartaric acid rotated a beam of polarized light passing
through it to the right, paratartaric acid did not rotate the light (Fig. 2). This puzzled
the young Pasteur. How could this be?

**Fig. 2.** Paratartrate does not rotate polarized light while
tartrate does.

Pasteur refused to accept the notion that two compounds that had the same chemical
composition yet acted so differently in respect to rotation of light could be identical.
He was convinced that the internal structure of the two compounds must be different and
this difference would show itself in the crystal form. The experts in this field had
looked examined tartrate and paratartrate crystals but never saw a difference, perhaps
because, as Duclaux thought, they believed that no difference could exist. Pasteur
believed that there were differences and indeed found them!  
  
Upon intense examination beneath his microscope, he saw that every crystal of pure
tartaric acid looked like every other one. When he examined the paratartrate crystals, on
the other hand, he saw two types of crystals, nearly identical but not quite! One type was
the mirror image the other -- the way the right hand mirrors the left hand. This was the
difference he was looking for!  
  
  
  
  
Pasteur then performed one of the simplest and yet most elegant experiments in the annals
of chemistry. With a dissecting needle and his microscope, he separated the left and right
crystal shapes from each other to form two piles of crystals (Fig. 3). He then showed that
in solution one form rotated light to the left, the other to the right. This simple
experiment proved that the organic molecules with the same chemical composition can exist
in space in unique stereospecific forms. And with this work did Pasteur launch the new
science of stereochemistry.     
      
                                                    
  
**Fig 3**. Dextro-
and levorotary forms of tartrate. An equal mixture of the two forms cancels each other's
rotation.  Hence, the mixture does not rotate polarized light.

To Pasteur this discovery had a deeper meaning. He proposed that asymmetrical molecules
were indicative of living processes. In the broadest sense, he was correct. We know today
that all of the proteins of higher animals are made up of only those amino acids that
exist in the left-hand form. The mirror image right-hand amino acids are not used by human
or animal cells. Likewise, our cells burn only the right-handed form of sugar, not the
left-handed form that can be made in the test tube. It was the discovery of asymmetry of
organic molecules that provided Pasteur with the   
"inescapable forward moving logic" that enchained him as he began his studies on
alcoholic fermentation.  
  
                          ***(For more details on Pasteur's crystallographic research, click on Rumford Medal)***  
  
**Alcoholic Fermentation**   
Pasteur served on the faculty of science of Dijon for a brief period and then was
transferred to Strasbourg University where he continued his studies on molecular
asymmetry. In Strasbourg, Pasteur had the immense good fortune to meet and marry the
University Rector's daughter Marie Laurent, who was to be his devoted wife, mother and
scientific helpmate through the remainder of his life.  
  
In 1854 Pasteur was appointed Dean and professor of chemistry at the Faculty of Sciences
in Lille, France. Lille was an industrial town with a number of distilleries and
factories. The Minister of Public Instruction was not completely sold on "science for
science's sake". He reminded university faculty that (and here I quote the Minister's
words) "whilst keeping up with scientific theory, you should, in order to produce
useful and far reaching results, appropriate to yourselves the special applications
suitable to the real wants of the surrounding country."  
  
Pasteur, in contrast to other faculty, needed no prodding. He enjoyed taking his students
on tours of the factories and was quick to advise the managers that he was available to
help solve their problems. In the summer of 1856, M. Bigot, father of one of his students
in chemistry, called upon Pasteur to help him overcome difficulties he was having
manufacturing alcohol by fermentation of beetroot. Often, instead of alcohol, Bigot's
fermentations yielded lactic acid.  
  
To better appreciate the discoveries to follow, we should understand what was believed at
that time about alcoholic fermentation. Chemistry was emerging as a true science, freed
from the pseudoscience of the alchemist. The mysterious chemical processes of living
animals were slowly being unraveled in strictly chemical terms. Lavoisier had shown that
chemical combustion in living animals was quantitatively identical to that occurring in a
furnace. Lavoisier also showed that sugar, the starting product of fermentation, could be
broken down to alcohol, CO2 and H2O by simply dropping a sugar solution on heated
platinum. Woehler startled the scientific world by synthesizing the organic compound urea,
showing for the first time that organic compounds, believed up to then as capable of
synthesis only by living animals could be made in a test tube. And due, in no small part
to Pasteur's work on crystals, internal structure and analysis of complex organic
compounds was becoming routine.  
  
In this light, fermentation leading to production of wine, beer and vinegar was believed
to be a straightforward chemical breakdown of sugar to the desired molecules. The chemical
experts of the day proclaimed that the breakdown of sugar into alcohol during fermentation
of sugar to wine and beer was due to the presence of inherent unstabilizing vibrations.
One could transfer these unstabilizing vibrations from a vat of finished wine to new grape
pressings to start fermentation anew.  
  
Yeast cells were found in the fermenting vats of wine, and were recognized as being live
organisms, but they were believed simply to be either a product of fermentation or
catalytic agents that provided useful ingredients for fermentation to proceed. Those few
biologists who earlier concluded that yeast was the cause of, and not the product of,
fermentation were ridiculed by the scientific experts: The deep conviction of the
scientific establishment was that chemistry had come too far to allow a vitalistic life
force theory to challenge pure chemical explanations of molecular reaction. To attribute
such chemical changes to mysterious life forces would represent a major backward step in
science!.   
  
Unfortunately, the "scientific establishment" was not providing much help to the
brewers of wine, beer and vinegar. These manufacturers were plagued by serious economic
problems related to their fermentations. Yields of alcohol might suddenly fall off; wine
might unexpectedly grow ropy or sour or turn to vinegar; vinegar, when desired, might not
be formed and lactic acid might appear in its place; the quality and taste of beer might
unexpectedly change making quality control a nightmare! All too often the producers would
be forced to throw out the resultant batches, start anew, and sadly have no better luck!  
  
Into M. Bigot's factory, microscope in hand, came Pasteur. He quickly found three clues
that allowed him to solve the puzzle of alcoholic fermentation. First, when alcohol was
produced normally, the yeast cells were plump and budding. But when lactic acid would form
instead of alcohol, small rod like microbes were always mixed with the yeast cells.
Second, analysis of the batches of alcohol showed that amyl alcohol and other complex
organic compounds were being formed during the fermentation. This could not be explained
by the simple catalytic breakdown of sugar shown by Lavoisier. Some additional processes
must be involved. Third, and this may have been the critical clue to Pasteur, some of
these compounds rotated light, that is they were asymmetric. As we said earlier, Pasteur
suspected that only living cells produced asymmetrical compounds. He concluded and was
able to prove that living cells, the yeast, were responsible for forming alcohol from
sugar, and that contaminating microorganisms turned the fermentations sour!  
  
Over the next several years Pasteur identified and isolated the specific microorganisms
responsible for normal and abnormal fermentations in production of wine, beer, vinegar. He
showed that if he heated wine, beer, milk to moderately high temperatures for a few
minutes, he could kill living microorganism and thereby sterilize (pasteurize), the
batches and prevent their degradation. If pure cultures of microbes and yeasts were added
to sterile mashes uniform, predictable fermentations would follow.  
  
 **Spontaneous Generation**  
In the midst of the great excitement and controversy created by Pasteur's research on
fermentation, a debate was ongoing in the scientific world on the theory of
"spontaneous generation". The idea that beetles, eels, maggots and now microbes
could arise spontaneously' from putrefying matter was speculated on from Greek and Roman
times. And in the 1860's spontaneous generation was still a subject of debate in the
exalted French Academy of Sciences. Against the advice of his colleagues, who saw dabbling
in this field as thankless and unrewarding, Pasteur entered the fray. Based on his work on
fermentation it seemed obvious to him that the sources of yeasts and other microorganisms
that were found during fermentation and putrefaction entered from the outside, for
example, on the dust of the air. Pasteur conducted a series of ingenious experiments that
destroyed every argument supporting "spontaneous generation". He showed that the
skin of grapes towards the beginning of grape harvest was the source of the yeast. Drawing
grape juice from under the skin with sterile needles gave juice that would not ferment.
Covering the grape arbors with fine cloth or wrapping the grapes with cotton to keep off
contaminating dust, gave grapes that would not produce wine. In order to show that dust of
the air was the carrier of contamination, he allowed air collected at different altitudes,
from sea level to mountain tops, to enter sterilized vessels containing fermentable
solutions. The higher the altitude the less the dust in the air and the fewer flasks
showed growth.  
  
The experimental design that clinched the argument was the use of the swan-neck flask. In
this experiment, fermentable juice was placed in a flask and after sterilization the neck
was heated and drawn out as a thin tube taking a gentle downward then upward arc --
resembling the neck of a swan (Fig 4). The end of neck was then sealed. As long as it was
sealed, the contents remained unchanged. If the flask was opened by nipping off the
end of the neck, air entered but dust was trapped on the wet walls of the neck. Under this
condition, the fluid would remain forever sterile, showing that air alone could not
trigger growth of microorganisms. If, however, the flask was tipped to allow the sterile
liquid to touch the contaminated walls and this liquid was then returned to the broth,
growth of microorganisms immediately began.

**Fig 4.**  Swan
flask.  The long "swan-like" neck is open to air, but dust and air-borne
microbes cannot reach the liquid.  Some of Pasteur's preparations are at the Pasteur
Institute, Paris where they continue to remain sterile for more than 100 years.

In the words of Pasteur "Never will the doctrine of spontaneous generation recover
from the mortal blow of this simple experiment. No, there is now no circumstance known in
which it can be affirmed that microscopic beings came into the world without germs,
without parents similar to themselves."   
  
 **Diseases of Silkworms**  
As if Pasteur was not busy enough with his studies on fermentation and
spontaneous generation, he was asked by the Department of Agriculture to head a commission
to see what could be learned about a devastating disease of silkworms that was destroying
the French silk industry (Fig. 5). Even though Pasteur knew nothing of silkworms and had
no idea that they suffered from disease, his research on silkworms forged another link in
his "inevitable" chain of discovery.

**Fig. 5**.  Healthy silkworms

Now there were at least two
different types of silkworm diseases
that Pasteur came to grips with: Pebrine, in which black spots and corpuscles are
generally, but not always, present on the worm. In such cases the worms often die within
the cocoons.  In the second type of disease, flacherie, the worms exhibit no
corpuscles or spots but fail to spin cocoons. Pasteur suspected, but was not sure, that
pebrine corpuscles were associated with the failure of the worms. Nonetheless, by
examining the worms under the microscope he was able to identify those free of pebrine and
used only their eggs for breeding. Next he excluded from breeding eggs from worms with
flacherie whom he identified by their sluggish behavior in climbing leaves when about to
construct cocoons. He instructed the silkworm farmers on these methods of selection and
how to use the microscope to detect sickness in the worms. Soon the silk industry in
France, Italy and other European countries returned to health.

Pasteur considered these studies important landmarks in his
investigations on infection and infectious disease. As he expanded his research, he found
that healthy worms became infected when allowed to nest on leaves used by infected worms.
He also noted that the susceptibility of the worms varied widely, some worms dying shortly
after infection, some weeks later, some not at all. He determined that temperature,
humidity, ventilation, quality of the food, sanitation and adequate separation of the
broods of newly hatched worms each played a role in susceptibility to the disease. So here
from Pasteur's research we see the emergence of his future concepts of the influence of
environment on contagion.  
  
 **Germ Theory of Disease**   
The crowning achievements of Pasteur's career were development of the germ theory of
disease and the use of vaccines to prevent these diseases. Pasteur's studies on
contamination of wine and beer by airborne yeast clearly stimulated certain investigators
to recognize that these "diseases" were due to entry of foreign microorganisms.
Lister in England was so impressed by Pasteur's work that he began to systematically
sterilize his instruments, bandages and sprayed phenol solutions in his operatories thus
reducing infections following surgery to incredibly low numbers.  
  
By 1875 many physicians recognized that some diseases were accompanied by specific
microorganisms, but the body of medical opinion was unwilling to concede that important
diseases --cholera, diphtheria, scarlet fever, childbirth fever, syphilis, smallpox - could
ever be caused by these agents. To give you an idea of the magnitude of the problem,
according to Pasteur's biographer son-in-law Vallery-Radot between April 1 and May 10,
1856, in the Paris Maternity Hospital there were 64 fatalities due to childbirth fever out
of 347 confinements. The hospital was closed and the patients were transferred to a
different hospital. Sadly, the contagion followed these women and nearly all of them died!  
  
As Pasteur wandered through hospital wards he became increasingly aware that infection was
spread by physicians and hospital attendants from sick to healthy patients. Pasteur
impressed on his physician colleagues that avoidance of microbes meant avoidance of
infection. In a famous speech before the august Academy of Medicine in Paris he stated,
"This water, this sponge, this lint with which you wash or cover a wound, may deposit
germs which have the power of multiplying rapidly within the tissue....If I had the honor
of being a surgeon....not only would I use none but perfectly clean instruments, but I
would clean my hands with the greatest care...I would us only lint, bandages and sponges
previously exposed to a temperature of 1300 to 1500 degrees. Slowly, but surely, through
the preaching of Pasteur, Lister and other physicians antiseptic medicine and surgery
became the rule.  
  
 **Anthrax**  
At this time, anthrax, a fatal disease of sheep and cattle, was decimating the sheep
industry and the economy of France. Important strides in identifying the causative agent
of anthrax had been made by the time Pasteur entered the arena. The great German
physician/scientist Robert Koch, isolated the anthrax bacillus, previously identified by
the French physician Davain, from infected spleens and showed that under resting
conditions the bacillus formed long-lived spores.  
  
Definitive proof was still lacking that the cultured bacillus, itself, and not something
carried along in Koch's culture medium was responsible to giving injected animals anthrax.
Pasteur provided this proof. As described by Dubos, Pasteur placed one drop of blood from
a sheep dying of anthrax into 50 ml of sterile culture, grew up the bacterium, and then
repeated this process 100 times. This represented a huge dilution of the original culture
so that not a single molecule of the original culture remained in the final culture. Yet,
the last culture was as active as the first in producing anthrax. As only the bacillus,
itself, by growing up each time in the new culture, could escape dilution, it proved
beyond all doubt that the anthrax bacillus and nothing else could be responsible for the
disease. Thus was the germ theory of disease firmly established!  
  
But how did the disease spread? Why was one field deadly to sheep, another harmless? Here
Pasteur's studies on silkworm contagion provided the clue. During one of Pasteur's
excursions to a field where sheep were grazing he noted that the ground in one part of the
field was differently colored than the rest. There it was that the farmer had buried some
sheep dead of anthrax. The color of the soil was due to earth worm casts. He realized that
earth worms were feeding on the carcasses of the buried sheep and bringing the anthrax
spores to the surface where other sheep could graze on the contaminated soil. Although
containment of the animals on uncontaminated fields would help control the spread of
anthrax, more was needed.  
  
Interestingly, Pasteur's studies on chicken cholera going on at this time provided the
breakthrough that led to development of specific vaccines to fight disease. Cholera was a
serious problem for farmers. Chicken cholera would spread through a barnyard rapidly and
wipe out the entire flock in as little as 3 days. Spread could be by contaminated food or
animal excrements. Pasteur had identified the cholera bacillus and was growing it in pure
culture. When injected, chicken invariably died in 48 hours.  
  
Then luck intervened. During the heat of the summer, Pasteur returned to Paris leaving the
cholera cultures used for infection stored on the shelves of the Arbois laboratory. Upon
return, Pasteur's collaborators were disappointed to find that these stored cultures no
longer killed injected chickens, nor even made them sick. The group set to work to make
new cultures of the bacillus and tested these batches on new birds and those healthy
previously treated birds. The results were astonishing: The previously injected birds were
unaffected by the bacillus, while the new birds all died. When Pasteur saw these results
he immediately realized that in a sense he was repeating the studies of Jenner 80 years
earlier who had conferred on humans immunity to smallpox by vaccinating individuals with a
mild form of cowpox. Pasteur then reproducibly manufactured attenuated cultures of chicken
cholera vaccines by growing the cholera bacillus at 42 - 43 degrees C. at which
temperature the bacillus is non-infectious. These attenuated bacterial cultures could
routinely prevent cholera in the vaccinated chickens.  
  
If attenuated cholera bacillus could render chickens resistant to the disease, would not
an attenuated anthrax bacillus render sheep immune to anthrax? By various techniques
involving oxidation and aging, anthrax vaccines indeed prevented anthrax in laboratory
trials. Pasteur's reports on preventing sheep anthrax were so exciting to some and
unbelievable to many, that he was challenged by the well-known veterinarian Rossignol to
conduct a carefully controlled public test of his anthrax vaccine. This was to take place
at Pouilly le Fort, a farm in the town of Melun south of Paris (Fig. 6). Twenty-five sheep
were to be controls, the other twenty-five were to be vaccinated by Pasteur and then all
animals would receive a lethal dose of anthrax. All of the control sheep must die and the
vaccinated sheep must live. When Pasteur's colleagues learned that he had agreed to the
test they were concerned. The challenge was severe and there was no room for error. The
vaccines were still in the developmental stage. "What succeeded with 14 sheep in our
laboratory will succeed with 50 at Melun", said Pasteur.  But inwardly
he worried  as his results were not always reproducible. Failure at Pouilly
le Fort would be a disaster.

Fortunately, Pasteur's colleagues Chamberlain and Roux followed
up the results of a research physician Jean-Joseph-Henri Toussaint who reported
a year earlier that carbolic-acid/heated anthrax serum would immunize against
anthrax. These results were difficult to reproduce and discarded although, as it
turned out, Toussaint was on the right track.  This led Pasteur and his
assistants to substitute an anthrax vaccine prepared not dissimilar to that of
Toussaint and different that Pasteur had announced.

Fig. 6. Vaccinating
sheep at Pouilly-le-fort.

The publicity was intense. A reporter from the London Times sent
back daily dispatches. Newspapers in France followed the events with daily bulletins.
There were crowds of onlookers, farmers, engineers, veterinarians, physicians, scientists
and a carnival atmosphere. Would Pasteur's claims of vaccination hold up? Even Pasteur was
privately concerned that he had acted impetuously in accepting the challenge. Happily, the
trial was a complete success -- indeed, a triumph! Two days after final inoculation (May
5, 1882), every one of 25 control sheep was dead and every one of the 25 vaccinated sheep
was alive and healthy. The fame of Pasteur and these experiments spread throughout France,
Europe and beyond. It was, says Duclaux, "the anthrax vaccine that spread through the
public mind faith in the science of microbes". Within 10 years a total of 3.5 M sheep
and a half M cattle had been vaccinated with a mortality of less than 1%. The immediate
savings to the French economy were enormous, at least 7 M francs, estimated to be enough
to cover the reparations that France was required to pay to Prussia for the loss of the
Franco-Prussian War in 1871. **To read the original report by Pasteur,
Chamberland and Roux,
click here.** 
  
Supported by the successes with anthrax and fowl cholera diseases, Pasteur identified and
isolated over the next 2-3 years the microbes for many other diseases including swine
erysipelas, childbirth fever and pneumonia.  
  

***(Robert Koch sharply criticized Pasteur's s******cientific
methodology and conclusions in the area of Anthrax Vaccination leading  to a
bitter dialog between these two scientists.  To learn more about this
controversy******,
click here.)*  
  

Rabies**  
The final and certainly most famous success of Pasteur's research was the development of a
vaccine against rabies or hydrophobia as it is also known. The disease has always had a
hold on the public imagination and has been looked upon with horror. It evokes visions of
"raging victims, bound and howling, or asphyxiated between two mattresses"
(Duclaux). The treatments applied to victims were as horrible as the supposed symptoms:
this included cauterizing the bite wounds with a red-hot poker. Actually very few persons
die in any year from being bitten by a rabid dog or wolf. The symptoms of the disease are
variable: onset may take weeks to months to develop if they develop at all. Nonetheless,
Pasteur and his colleague Roux realized that conquest of rabies would be recognized as a
great achievement to the world of science and to the public at large.  
  
Pasteur and Roux initially attempted to transfer infection by injecting healthy dogs with
saliva from rabid animals. The results were variable and unpredictable. Later, recognizing
that the active agent was in the spinal cord and brain, and because they were unable to
detect a specific rabic microorganism, Pasteur and Roux applied extracts of rabid spinal
cord directly to the brain of dogs. With this technique they could reproducibly produce
rabies in the test animals in a few days.  
  
The goal was next to develop a vaccine that would provide protection to the subject before
the rabic agent moved from the bite site to the spinal cord to the brain. This was
achieved by injecting into test animals suspensions of spinal cord of rabid rabbits that
were attenuated in strength by air drying over a 12-day period in the now-famous Roux
Bottle (Fig. 7).  A strip of spinal cord was suspended from a hanger in the center of
the bottle containing a hole at the top of the bottle and one on the lower side. Air
entered from the bottom opening, passed over a drying agent and exited from the top. The
longer the cord was dried, the less potent was the tissue in producing rabies.

**Fig. 7.** Roux bottle. The
strip of spinal cord hangs from the top of the flask; the drying agent rests on the
bottom. Sterility is preserved by the cotton plugs that allow air but not microbes to
enter.

The treatment plan used to develop immunity to rabies was to
inject under skin of a dog the least potent preparation of minced spinal cord, followed
every day for the next 12 days with a stronger and stronger extract. At the end of this
time, the animal was completely resistant to bites of rabid dogs and failed to develop
rabies if the most potent extracts were applied directly to the brain.   Forty
dogs were successfully treated in this manner.  
  
Following confirmation of his reports in 1885 that he had made dogs refractory to rabies
by vaccination, Pasteur received wide acclaim and much favorable publicity. But why not
use the vaccine on humans? Frankly, Pasteur was terribly afraid of things going wrong and
he was particularly uneasy about being unable to isolate the rabic substance. And so he
continued to insist that many years of additional research were necessary before the
treatment could be tried on humans.

**Fig. 8**.   Pasteur
examining a spinal cord specimen in a Roux bottle.

But the press of events made him act sooner.  Two initial
trials in humans conducted with the help of his physician colleagues were indecisive: one
patient was discharged from the hospital by the administration after one injection and
what became of him was not known;  the second patient was a young girl suffering from
an advanced stage of rabies such that she died shortly after the procedure had commenced.
  A full trial of the anti-rabies vaccine was yet to be made.  On July 6, 1885,
9 year old Joseph Meister and his mother appeared at Pasteur's laboratory. Two days
earlier the young boy had been bitten repeatedly by a rabid dog. He was so badly mauled
that he could hardly walk. His mother appealed to Pasteur to treat her son as this youth
faced certain death.  Pasteur, with much trepidation, after consultation
with Drs. Grancher and Vulpian, two distinguished physicians of the Academy of
Medicine, agreed that the youth must be treated to save his life.  Despite
Pasteur's fears, Meister made a perfect recovery and remained in fine health for the
remainder of his life.  
  
A few months later another victim turned up. He was a young shepherd also bitten by a mad
dog. Following reports of his successful treatments, the wild acclaim for Pasteur knew no
bounds! Victims of dog and wolf bites from France, Russia, the United States poured into
his laboratory for treatment. The newspapers and public followed these treatments and
cures with intense interest. Pasteur became a hero and a legend. The Pasteur Institute
funded by public and governmental subscriptions was built in Paris initially to treat
victims of rabies who were coming to Pasteur's laboratory in increasing numbers. Later,
Pasteur Institutes were built, including 3 in the United States, to deal with human rabies
and other diseases.

***(To
read a
translation of the original paper by Pasteur presented before the French Academy
of Science in 1885 describing his treatment for rabies,
Click
here) .***     
  
Rabies was the last major research of the master scientist. His health was failing and a
paralysis of his left side from a serious stroke he suffered in his 46th year made his
working in the laboratory increasingly difficult. Pasteur died September 28,1895 after suffering
additional strokes. He was buried, a national hero, by the French Government. His funeral
was attended by thousands of people (Fig. 9).

**Fig. 9.**  Pasteur
funeral cortege

Shortly after his death the London Illustrated News published as
a tribute to the great scientist a full page portrait
of Pasteur on its front page (Fig. 10).

                                               
                                                                    **Fig. 10.**  Tribute to Louis Pasteur.

His remains, initially interred in the Cathedral of Notre Dame,
was transferred to a permanent crypt in the Pasteur Institute, Paris (Fig 11).

**Fig. 11.** The
marble and mosaic crypt in the lower level of the Pasteur Institute, Paris.

In a tragic footnote to history, Joseph Meister, the first person
publicly to receive the rabies vaccine, returned to the Pasteur Institute as an employee
where he served for many years as Gatekeeper. In 1940, 45 years after his treatment for
rabies that made medical history, he was ordered by the German occupiers of Paris to open
Pasteur's crypt. Rather than comply, Joseph Meister committed suicide!

(Copyright, 1996; rev. Jan. 2001; Mar 2001; Jun. 2001;
Oct. 2004)
