# Supplementary material for: Space groups and crystallographic symmetry: writing a multi-featured tutorial in a new style
Source: Acta Crystallogr E Crystallogr Commun. 2021 Jul 16;77(Pt 9):857–63. doi: 10.1107/S2056989021007039 (PMC8423017; doi:10.1107/S2056989021007039)
Supplement: Supplementary file 1 [file e-77-00857-sup2.zip › symandsg/Main/intro.html]

Introduction

|  |  |  |
| --- | --- | --- |
|  |  |  |

Overview

Identity

Proper Rotation

Inversion

Reflection

Improper Rotation

Overview

Examples

Exercise

Use of Point Groups

  

You are here: Symmetry & Point Groups � Introduction

  
  


# Introduction

**Why do chemists care about symmetry and point groups?**

Symmetry
helps us understand molecular structure, some chemical properties, and
characteristics of physical properties (spectroscopy).

This tutorial is set up to quickly guide you through learning to:

- "see" the symmetry of molecules,
- chracterize symmetry elements, and
- determine point groups.

**How to use the tutorial?**

**1. Java Applets**

- If you do not see the applets, please install Java applet from http://java.sun.com/products/plugin/
- Click
  your mouse in the applet area. First get familiar with the applets, how
  they work. Then rotate clockwise, counter-clockwise, or any other
  direction you want.
  - Rotate the molecule by holding down the left mouse button and dragging the mouse
  - Rotate about the axis perpendicular to the screen by holding down the control key while dragging as described above.
  - Scale the model by holding down the alt key while dragging.
  - Translate the model by holding down the shift key while dragging
- Keyboard Controls  
  **b** - Ball & Stick style  
  **l** - Line style  
  **s** - Space-filled style  
  **h** - toggle hydrogen atoms (if any)  
  **r**- reset orientation and drawing style

**2. Flash images**

- If you do not have Flash Player, please download it from Macromedia Flash Player Download Center.
- Most
  flash images are mouse overs. To compare different versions of the
  images, we provided different orientations of the image. Do a mouse
  over on the image when it is asked.

**3. Quiz**

- The
  quiz part of the tutorial is to help you gain expertise in symmetry
  elements and point groups. You can try as many times as you want.

|  |  |  |
| --- | --- | --- |
|  |  |  |

|  |  |
| --- | --- |
| Close Tutorial |  |
