# Supplementary material for: Space groups and crystallographic symmetry: writing a multi-featured tutorial in a new style
Source: Acta Crystallogr E Crystallogr Commun. 2021 Jul 16;77(Pt 9):857–63. doi: 10.1107/S2056989021007039 (PMC8423017; doi:10.1107/S2056989021007039)
Supplement: Supplementary file 1 [file e-77-00857-sup2.zip › symandsg/Main/iscrizione.htm]

Associazione Italiana Cristallografia

Associazione Italiana Cristallografia

Associazione Italiana Cristallografia

Associazione
Italiana Cristallografia

Associazione
Italiana Cristallografia

**Home**

**NEWS**

**Crystallography   
in Italy**

**Meeting and School**

**L'Associazione  
(in Italian)**

**Addresses of
AIC Members**

**Archive**

**Links**

|  |  |
| --- | --- |
|  | **Consiglio di Presidenza e Commissioni** |
|  | **Regolamento** |
|  | **Statuto** |
|  | **Iscrizione** |
|  | **Verbali** |
|  | **Premi AIC** |
|  | **Storia AIC** |

**ASSOC****IAZIONE ITALIANA DI CRISTALLOGRAFIA**

**dal REGOLAMENTO
- Art. 1**  
La quota ordinaria annuale di iscrizione ammonta a

- 15 Euro, per i soci con meno di 35 anni;   
- 25 Euro, per i soci che abbiano compiuto i 35 anni d' età
nell' anno in cui la quota si riferisce.   
  
Per organizzazioni pubbliche o private la quota annuale è di 100 Euro  
  
La quota individuale come socio ECA ammonta a 10 Euro, che possono
essere versate in aggiunta
alla quota per l'AIC

Iil versamento delle quote va effettuato tramite
bollettino sul c/c postale 10747277 - Associazione Italiana di
Cristallografia.  
  
**dallo STATUTO - Art. 3**   
Può essere iscritta nell'Associazione ogni persona
fisica che ne condivida le finalità e la cui domanda d'iscrizione
sia presentata da almeno due Soci.

Il modulo d'iscrizione può
essere scaricato cliccando su formato di file desiderato

|  |  |
| --- | --- |
| iscrizione.rtf | iscrizione.doc |

Webmaster   
**Rita Ollà**  
 ritaolla@unipr.it  
  
*last update*   
*17 Luglio 2006*
