# Supplementary material for: Space groups and crystallographic symmetry: writing a multi-featured tutorial in a new style
Source: Acta Crystallogr E Crystallogr Commun. 2021 Jul 16;77(Pt 9):857–63. doi: 10.1107/S2056989021007039 (PMC8423017; doi:10.1107/S2056989021007039)
Supplement: Supplementary file 1 [file e-77-00857-sup2.zip › symandsg/Main/jmr.htm]

J.M. Robertson

# John Monteath Robertson, C.B.E., F.R.S. (1900-1989)

*Gardiner Professor of Chemistry, Glasgow University, 1942-70*

---


John Monteath Robertson ("JM") was a pioneer in the field of X-ray
crystallography and the founder of organic crystallography. Among many
other achievements, he is noted for the development of heavy-atom and
isomorphous-replacement methods for solving the phase problem in
chemical crystallography, thereby laying the foundations for subsequent
determination of the structures of proteins and other biological
macromolecules by X-ray diffraction. In 1939 (Nature, **143**,
75) he predicted that the structure of insulin would be solved in this
way - a task finally accomplished by Dorothy Hodgkin and her Oxford
group in 1969.

Gardiner Professor 1942-70  
F.R.S. 1945  
Royal Society Council 1954-56  
Davy Medal 1960  
CBE 1962   
Longstaff Medal of the Chemical Society 1966  
Paracelsus Medal of the Swiss Chemical Society 1971  
Gregori Aminoff Medal 1962  
President of the Chemical Society 1962-64

---

Return to  Chemistry home page.
