# Supplementary material for: Space groups and crystallographic symmetry: writing a multi-featured tutorial in a new style
Source: Acta Crystallogr E Crystallogr Commun. 2021 Jul 16;77(Pt 9):857–63. doi: 10.1107/S2056989021007039 (PMC8423017; doi:10.1107/S2056989021007039)
Supplement: Supplementary file 1 [file e-77-00857-sup2.zip › symandsg/Main/Kaftory.htm]

Kaftory


## Chemical Crystallography: Structure and Reactivity in Organic Solids

|  |  |
| --- | --- |
|  | Menahem KAFTORY   **Professor**  Born in Israel 1943  M.Sc. 1971 and D.Sc. 1973 Technion  On Technion faculty since 1979  Technion's Dean of Students (1996-1999)  Head of the Center for Pre-Academic Education (2002-2005) Dean, Department of Chemistry (since January 1, 2006) |


Prof. Menahem KAFTORY  
Department of Chemistry  
Technion - Israel Institute of Technology  
Haifa 32000, Israel

|  |  |
| --- | --- |
|  | +972-4-8293761 |
|  | +972-4-8295703 |
|  | kaftory@techunix.technion.ac.il |
| WWW | http://www.technion.ac.il/technion/ chemistry/staff/kaftory/ |

:   RESEARCH INTERESTS
:   SELECTED PUBLICATIONS


**Department of Chemistry Home Page** ---
