# Supplementary material for: Space groups and crystallographic symmetry: writing a multi-featured tutorial in a new style
Source: Acta Crystallogr E Crystallogr Commun. 2021 Jul 16;77(Pt 9):857–63. doi: 10.1107/S2056989021007039 (PMC8423017; doi:10.1107/S2056989021007039)
Supplement: Supplementary file 1 [file e-77-00857-sup2.zip › symandsg/Main/lattices.htm]

Bravais lattices

# The fourteen Bravais lattices

There are fourteen distinct space groups that a Bravais lattice can have. Thus, from the point of view of symmetry,
there are fourteen different kinds of Bravais lattices.  
 Auguste Bravais (1811-1863) was the first to count the categories correctly.

## The seven crystal systems

I list below the seven crystal systems and the Bravais lattices belonging to each.

---

### *Cubic (3 lattices)*

The cubic system contains those Bravias lattices whose point group is just the symmetry group of a cube.
Three Bravais lattices with nonequivalent space groups all have the cubic point group. They
are the simple cube, body-centered cubic, and face-centered cubic.


### *Tetragonal (2 lattices)*

The simple tetragonal
is made by pulling on two opposite faces of the simple cubic and
stretching it into a rectangular prism with a square base, but a height
not equal to the sides of the square. By similarly stretching the
body-centered cubic one more Bravais lattice of the tetragonal system
is constructed, the centered tetragonal.

### *Orthorhombic (4 lattices)*

The simple orthorhombic is made by deforming the square bases of the tetragonal into rectangles, producing an object
with mutually perpendicular sides of three unequal lengths. The base orthorhombic is obtained by adding a lattice point on two opposite
sides of one object's face. The body-centered orthorhombic is obtained by adding one lattice point in the center of the object. And
the face-centered orthorhombic is obtrained by adding one lattice point in the center of each of the object's faces.

### *Monoclinic (2 lattices)*

The simple monoclinic is obtained by distorting the rectangular faces perpendicular to one of the orthorhombic axis into
general parallelograms. By similarly stretching the base-centered orthorhombic one produces the base-centered monoclinic.

### *Triclinic (1 lattice)*

The destruction of the cube is completed by moving
the parallelograms of the orthorhombic so that no axis is perpendicular
to the other two.
The simple triclinic produced has no restrictions except that pairs of opposite faces are parallel.

### *Trigonal (1 lattice)*

The simple trigonal (or rhombohedral) is obtained by stretching a cube along one of its axis.

### *Hexagonal (1 lattice)*

The hexagonal point group is the symmetry group of a prism with a regular hexagon as base. The simple hexagonal bravais
has the hexagonal point group and is the only bravais lattice in the hexagonal system.
