# Supplementary material for: Space groups and crystallographic symmetry: writing a multi-featured tutorial in a new style
Source: Acta Crystallogr E Crystallogr Commun. 2021 Jul 16;77(Pt 9):857–63. doi: 10.1107/S2056989021007039 (PMC8423017; doi:10.1107/S2056989021007039)
Supplement: Supplementary file 1 [file e-77-00857-sup2.zip › symandsg/Main/laue-bio.html]

Max von Laue - Biography

- Nobel Foundation
- Nobel Media
- Nobel Museum
- Nobel Peace Center
- Nobel Web
- SEARCH
- CONTACT US
- HOME


- NOBEL PRIZES
- ALFRED NOBEL
- PRIZE AWARDERS
- NOMINATION
- PRIZE ANNOUNCEMENTS
- AWARD CEREMONIES
- EDUCATIONAL GAMES

- By Year
- Nobel Prize in Physics
- Nobel Prize in Chemistry
- Nobel Prize in Medicine
- Nobel Prize in Literature
- Nobel Peace Prize
- Prize in Economics


## Max von Laue

The Nobel Prize in Physics 1914

## Biography

**Max Laue** was born on October 9,
1879 at Pfaffendorf, near Koblenz. He was the son of Julius von
Laue, an official in the German military administration, who was
raised to hereditary nobility in 1913 and who was often sent to
various towns, so that von Laue spent his youth in Brandenburg,
Altona, Posen, Berlin and Strassburg, going to school in the
three last-named cities. At the Protestant school at Strassburg
he came under the influence of Professor Goering, who introduced
him to the exact sciences.  
  
In 1898 he left school and for a year did his military service.
He then went to the University of Strassburg where he studied
mathematics, physics and chemistry; but soon he moved to the
University
of Göttingen, where he worked under Professor W. Voigt
and Professor W. Abraham, who greatly influenced him. After a
semester at the University of Munich he went, in 1902, to the
University of
Berlin to work under Professor Max Planck. Here he attended lectures by
O. Lummer on interference spectroscopy and heat radiation, the
influence of which was shown in von Laue's dissertation on
interference phenomena in plane-parallel plates.  
  
After obtaining his doctorate at Berlin in 1903, von Laue went
for two years to the University of Göttingen. In 1905 he was
offered the post of assistant to Max Planck at the Institute for
Theoretical Physics at Berlin. Here he worked on the application
of entropy to radiation fields and on the thermodynamic
significance of the coherence of light waves.  
  
In 1909 he went as Privatdozent to the University of Munich,
where he lectured on optics, thermodynamics and the theory of
relativity and in 1912 he became Professor of Physics at the
University of
Zurich. In 1914 he moved, as Professor of Physics, to
Frankfurt
on Main and from 1916 he was engaged in war work at the
University
of Würzburg on high vacuum tubes used for telephony and
wireless communication. In 1919 he was appointed Professor of
Physics at the University of Berlin, a post which he held until
1943. From 1934 onwards he acted as consultant to the
Physikalisch-Technische Reichsanstalt at
Berlin-Charlottenburg.  
  
In 1917, when the Institute for Physics was established at
Berlin-Dahlem with Einstein as
its Director, von Laue had charge, as Second Director, of most of
the administrative work of this Institute, which was in close
touch with German scientific research. Von Laue exerted, during
this period and also later, considerable influence on the
development of scientific research in Germany. When Berlin was
bombed, this Institute moved to Hechingen, in Württemberg
and von Laue accompanied it there. He remained at Hechingen from
1944 until 1945 and here, to distract his thoughts from the war,
he wrote a History of Physics, which went into four editions and
was translated into seven other languages. Here he welcomed the
arrival of the French troops and was taken by an Anglo-American
mission, together with nine other German scientists, to England
where he remained until 1946 During his confinement in England he
wrote a paper on the low absorption of X-rays during diffraction,
which he contributed in 1948, to the International Union of
Crystallographers at Harvard University. In 1946 he went to
Göttingen as Acting Director of the Max Planck Institute and
Titular Professor in the University there.  
  
In 1951 he was elected Director of the Fritz Haber
Institute for Physical Chemistry at Berlin-Dahlem and here he
did much work on X-ray optics in collaboration with Borrmann and
others.  
  
In 1958 he retired and in 1959 his 80th birthday was celebrated
in Berlin-Dahlem. He lived on, still actively at work, for
another six months.  
  
Apart from his earlier work already mentioned, von Laue's
scientific work extended over a wide field. Early in his career
he was greatly excited by Einstein's theory of relativity and
between 1907 and 1911 he published eight papers on the
application of this theory. In 1911 he published a book on the
restricted theory and in 1921 another on the general theory, both
books going into several editions  
  
His best known work, however, for which he received the Nobel
Prize for Physics for 1914, was his discovery of the diffraction
of X-rays on crystals. This discovery originated, as he related
in his Nobel Lecture, when he was discussing problems related to
the passage of waves of light through a periodic, crystalline
arrangement of particles. The idea then came to him that the much
shorter electromagnetic rays, which X-rays were supposed to be,
would cause in such a medium some kind of diffraction or
interference phenomena and that a crystal would provide such a
medium. Although his colleagues Sommerfeld, W. Wien and others,
with whom he discussed the idea on a skiing expedition, raised
objections to the idea, W. Friedrich, one of Sommerfeld's
assistants and P. Knipping tested it out experimentally and,
after some failures, succeeded in proving it to be correct. Von
Laue worked out the mathematical formulation of it and the
discovery was published in 1912. It established the fact that
X-rays are electromagnetic in nature and it opened the way to the
later work of Sir William and Sir
Lawrence Bragg. Subsequently von Laue made other
contributions to this subject.  
  
Also prominent in von Laue's work were his contributions to the
problems of superconductivity which he made when he was Professor
of Theoretical Physics at Berlin University. At this time Walther
Meissner was studying at the Physikalisch-Technische
Reichsanstalt in Berlin, the remarkable disappearance of ohmic
resistance shown by many metals at temperatures of the order of
that of liquid helium. An especially valuable contribution then
made by von Laue was his explanation, in 1932, of the fact that
the threshold of the applied magnetic field which destroys
superconductivity varies with the shape of the body because, when
the magnetic field is established after the state of
superconductivity has been established, the magnetic field is
deformed by the supercurrents induced at the surface of the metal
being used. This explanation was confirmed and it opened the way
to Meissner's subsequent discovery that a superconductor
eliminates the whole magnetic field in its interior and this
became the basic idea of F. and H. London's theory of
superconductivity. Von Laue published one paper in collaboration
with F. and H. London and between 1937 and 1947 he published a
total of 12 papers and a book on this subject.  
  
Among the many honours and distinctions which he was awarded were
the Ladenburg Medal, the Max-Planck Medal and the
Bimala-Churn-Law Gold Medal of the Indian Association at
Calcutta. He held Honorary Doctorates of the Universities of
Bonn, Stuttgart, Munich, Berlin, Manchester and Chicago, was a
member of the Russian Academy and the Academy of Sciences of
Berlin, the German Physical Society and Mathematical Society, the
Kant Society, the Academy of Sciences of Vienna, the American
Physical Society, the Société Française de
Physique and the Société Française de Mineralogie
et Crystallographie. He was also Honorary Senator of the
MaxPlanck Society and Honorary Member of the German Röntgen
Society, and Corresponding Member of the Academies of Sciences of
Göttingen, Munich, Turin, Stockholm, Rome (Papal), Madrid,
the Academia dei Lincei of Rome, and the Royal Society of London.
In 1948 he became Honorary President of the International Union
of Crystallographers, in 1952 he was made a Knight of the Order
Pour le Mérite, in 1953 he received the Grand Cross with
Star for Federal Services, and in 1957 he became an Officer of
the Legion of Honour of France.  
  
Much esteemed by his contemporaries for his character and sound
judgment, von Laue's opinions were often sought and during his
life he exerted great influence on the direction and development
of German scientific work. Among his characteristics were a deep
love and admiration of Prussia and a strong sense of justice and
fair play. When Hitler and the National Socialist Party were in
power, he defended, even at the risk of reprimand or personal
injury, scientific views, such as the theory of relativity, which
were not approved by the Party or by such strong adherents to it
as the physicist Lenard. When Einstein resigned from the Berlin
Academy and the Vice-President of this Academy stated that this
was no loss, von Laue was the only member of the Academy who
protested.  
  
Chief among his recreations were sailing, skiing, mountaineering
and motoring. Von Laue was not a rock-climber, but preferred to
tour the Alpine glaciers with his scientific friends. As a
motorist he was famous in Berlin, first on the motor bicycle on
which he went at high speed to his lectures; and later in a car.
He loved high speeds, but never, until the fatal collision that
ended his life, had any accident.  
  
In his later years he suffered from attacks of depression and a
feeling of being persecuted by scientists and by the military
authorities, whom he disliked intensely. Usually, however, he
successfully overcame these attacks and regained his sense of
humour and joy in life. He did not practise any art, but he took
an interest in many arts, especially in classical music; and he
read widely history and the philosophy of science. He thought of
the stars, the mountain peaks and the achievements of the human
with awe and humility and was at heart a deeply religious man. He
asked that his tombstone should bear the statement that he died
trusting firmly in the mercy of God.  
  
In 1910 von Laue married Magdalena Degen.  
  
On April 8, 1960, when he was driving alone to his laboratory, a
motor cyclist, who had only received his licence two days
previously, collided with von Laue's car. The motor cyclist was
instantly killed and von Laue's car overturned in the Berlin
speedway and he was taken from beneath it by the Fire Brigade.
Although he showed at first some signs of recovery from his
injuries, he died of them on April 24, at the age of 80.

From *Nobel Lectures, Physics 1901-1921*, Elsevier Publishing Company, Amsterdam, 1967


This autobiography/biography was first
published in the book series *Les
Prix Nobel*.
It was later edited and republished in *Nobel
Lectures*.
To cite this document, always state the source as shown above.

Copyright © The Nobel Foundation 1914

- Printer Friendly
- Comments & Questions
- Tell a Friend

#### The 1914 Prize in:

PhysicsChemistryMedicineLiteraturePeace

- Prev. year
- Next year

#### The Nobel Prize in Physics 1914

- Presentation

##### Max von Laue

- Biography
- Nobel Lecture
- Swedish Nobel Stamps

Only 33 out of 758 are women!

Who can nominate for a Nobel Prize?

"War leaves no victors, only victims."


The Official Web Site of the Nobel Foundation

Copyright © Nobel Web AB 2006
