# Supplementary material for: Space groups and crystallographic symmetry: writing a multi-featured tutorial in a new style
Source: Acta Crystallogr E Crystallogr Commun. 2021 Jul 16;77(Pt 9):857–63. doi: 10.1107/S2056989021007039 (PMC8423017; doi:10.1107/S2056989021007039)
Supplement: Supplementary file 1 [file e-77-00857-sup2.zip › symandsg/Main/Laves.htm]

Laves

# Fritz Laves (27.2.1906 – 12.8.1978)

Fritz
Laves was born in Hannover but spent most of his youth in Göttingen. From his
father who was a judge he gained a sense of justice that was to be evident in
his later actions. He tried to muster the support of the members of the Faculty
of Science at the University of Göttingen so that V. M. Goldschmidt could stay
on after 1933; after World War II he became a member of a commission at the
University of Marburg which decided on the re-employment of former University
professors; he participated in discussions concerning the reform of the E.T.H.
in Zürich.

F.
L. maintained a long and active interest in music and the arts. As a piano
student of 17 he used the confusion created by run-away inflation to pawn a
child's microscope three times in order to buy piano scores of all the sonatas
of Beethoven and Mozart. Later he liked to buy records with artists' individual
interpretations of piano sonatas and concerts. He was proud of being a
descendant of Georg Ludwig Friedrich Laves (1788 - 1864), the court architect
of the King of Hannover and England for five decades, who was responsible for
Hannover's opera house, the Wangenheim palace, changes in the "Leine
Schloß", as well as a city plan for the town itself. It was said that F.
L. inherited not only several paintings of the 18th and 19th century but also
his three dimensional vision which was highly praised by V. M. Goldschmidt. His
literary favorites ranged from Goethe, to Thomas Mann, Hermann Hesse, and
Hermann Kasack. He also inherited two pistols of Goethe's Werther from his
great grandmother, Charlotte Buff.

His
artistic interests were shared by his wife Melitta Druckenmüller trained as an
architect, she became involved in his frameworks and representations of crystal
structures. She is responsible for many of the figures in his publications.
Esteemed by colleagues, co-workers, and students, Melitta Laves stood by her
husband in critical situations, especially after his heart attack of 1972.
Because of her help and care, F. L. recovered quickly in their house on the
Italian Riviera and was able to continue his scientific writings to the end of
his life.

F.
L.'s scientific activity began as a twelve year old school boy who collected
rocks and minerals. He had his first contact with "Geheimrat"
Professor Mügge, Director of the Mineralogical Institute, who gave him
permission to see the museum as often as he wishes. The young boy was also told
that one of his specimens was a feldspar with the composition KAlSi_3_O_8_ which was to be a critical fact in his scientific thinking.

When
Fritz Laves finished the gymnasium in 1924 his father suggested that his son
study geology because he could not see any profession for a mineralogist other
than as a professor; and that itself seemed to be rather risky. F. L. began his
studies in Innsbruck, continued in Göttingen, and later went to Zürich. It was
in Zürich that P. Niggli's ideas in crystallography convinced him to go into
this field; at that time Brandenberger, Heesch, Ito, Nowacki, Parker in
crystallography and the mathematicians van der Waerden and J. J. Burkhardt were
creating a systematic nomenclature for crystal-chemical description. P. Niggli
in 1919 had already proposed the term lattice complex, but it was used in
another sense too, namely as a configuration of a lattice complex. F. L.
started to study the lattice complexes in respect to their properties of sphere
packings in a few space groups of the crystal classes C_4_ and S_4_
and convinced P. Niggli that it would take longer than one human life to
calculate the homogeneous sphere packings of all 230 space groups by hand. The
discussion resulted in a thesis about "Bauzusammenhänge" introducing
the terms "Insel, Kette, Netz, Gerüst". In 1978 F. L. made the
following retrospective remarks about this paper:

"Stimulated by ideas of P. Niggli
(1927), Laves (1930) tried to present a classification of crystal structures on
the basis of topological concepts and metrical relations between 'units', from
which the crystal structures may be considered to consist. For this purpose
some definitions had to be formulated for words or word combinations that were
already used in the literature, however undefined, that is varying in their
meaning. This holds especially for the German words 'Element, Einheit,
Zusammenhang, Verband'. For distinction the syllable 'Bau-' has mostly be
added. As an English translation the word combinations 'web element', 'web
unit', 'web assemblage' and 'web connection' may be proposed."

Actually,
the classification could have been done without the word "Element"
and "Zusammenhang" as these are only "head concepts" for
the different kinds of "Einheiten" and "Verbände",
specified by additional words. He also proposed the following translation in
1978:

Bau-Insel        web island,  
Bau-Kette       web chain,  
Bau-Netz        web net,  
Bau-Skelett     web skeleton.

In the second part of the thesis he applied the
letters *I*, *K*, *N*, *G* for homogeneous and *i, k, n,
g* for heterogeneous "Bauverbände" to structure elements of AB and
more complex compounds. For further divisions F. L. had to introduce the
coordination numbers and (normalized) distances in a complete de­scription. For
example:

In
his only paper with P. Niggli homogeneous system-symmorphic nets and frameworks down to orthorhombic
system are shown in respect to their coordination number, space filling, and
their dependence on parameters and axial ratios. The plane partitioning into
Wirkungsbereiche (Voronoi polyhedra or Wigner-Seitz cells) and coordination
numbers are studied in detail. The thinnest sphere packing is found when the
spheres of *Y\** are replaced by three; only 5.6% of the space are filled.
At the meeting on isomorphy in Göttingen F. L. presented a paper about
miscibility and typy, iso and heterotypy. Because of the importance of these
definitions for crystal chemical considerations and because of the war (the
issue of "Die Chemie" had only a few copies) the article with
additional remarks, Table of Contents and Bibliography is reprinted here. The
search for a unique definition of the coordination number was a matter of
discussion in several publications during his whole life. His coworkers Brunner
and Schwarzenbach (1971) use a maximum gap in a distance histogramm - i.e., a
registration of the distances of successive coordination shells to define the
characteristic coordination number.

This
work in theoretical crystallography (Bauverbände, sphere packings, space and
plane partitioning) provided the impetus for several review and survey articles
on different subjects such as silicates, AB_2_ compounds, metals and
intermetallic compounds and in general on inorganic compounds. Besides the
geometrical factors F. L. also discussed the influence of valence electrons,
ionic bonding, and anomalous volume contraction.

The
first two decades of his scientific life (1928 - 1948), were spent in Zürich
(doctorand and assistent, until 1930), Göttingen (as assistant and dozent until
1943), Halle (as Extraordinarius and Director of the Institute until 1945), and
Marburg (as Ordinarius and Director of the Institute until 1948), F. L. was
engaged mainly in two large fields of research, namely metals and intermetallic
compounds and order/disorder. As an assistant of V. M. Goldschmidt and inspired
by the school of Tamann (and his successor Masing) and in cooperation with
Wallbaum, Witte, Löhberg, and Ralfs the factors governing the structure of
intermetallic compounds were estimated.

The first paper about coordination numbers and valence
electron in intermetallic compounds, presented by V. M. Goldschmidt to the
"Gesellschaft der Wissenschaften zu Göttingen" in 1932, was a proof
of the Hume-Rothery rules for *beta*- and *gamma*-brass.
In the same year F. L. finished his habilitation thesis on the structure of Ga,
which showed dumbbells at the first coordination. Besides the isotypy between
Mg_3_Al_2_ (better Mg_17_Al_12_) and *alpha*-Mn
several representatives of the Laves-Friauf structure types of MgCu_2_,
MgZn_2_, and their relation to MgNi_2_ were shown. The
existence of these three types in the ternary system Mg-Cu-Al and in the
quasibinary section MgCu_2_-MgAl_2_ was de­termined by X-ray
analysis. The "Zintl" line in the periodic table separating
intermetallic compounds from salts and polyanionic salts has been checked by F.
L. with the occurrence of the NiAs-type. While V. M. Goldschmidt had shown the
factors controlling the structure of ionic substances (ratio of the compounds,
ratio of the radii, polarization) F. L. summarized the state of knowledge of
intermetallic compounds in several review articles describing the space symmetry
and connection principles and introducing the *Q*-factor*,* for example *A_m_B_n_*compounds

|  |  |  |
| --- | --- | --- |
|  | Q = (*d_A_* + *d_B_*) / 2*d_AB_* |  |
|  | *d_A_* (*d_B_*) | the shortest distance between *A*(*B*) atoms; |
|  | *d_AB_* | the shortest distance between *AB* atoms; |

to show
the deviation from heterogenity (i.e. NaCl, *Q =* 1.41, NiAs, *Q* =
1.19). Structures with large contraction as La in LaNi_2_ (MgCu_2_-type)
require a "suitable electron transfer".

Through
the influence of Paul Rosbaud, Laves was ordered to leave the army during World
War II and to work on metallurgical problems, especially to look for cubic
Mg-alloys for airplanes; also for answers to dissolution problems of Al-Cu and
Al-Mg.

In
the last one and a half decades of his life he studied Al, Ga, V, Nb, and Ta
alloys in respect to the superconductivity of the *beta*-W-Cr_3_Si-type,
in dependence on phase transition and pressure, and in respect to the change of
*T_c_*.

The
order-disorder problem in crystals is a leitmotif in F. L.'s whole scientific
work. Together with Nieuwenkamp and Witte he showed already in 1935 the difference
streaks in the reciprocal space for 2-dimensional order and 1-dimensional
disorder (especially Lagenfehlordnung) in crystals. With Jagodzinski the
solution of this problem was given, - solutions had been found for ZnS
(wurtzite/sphalerite), carborundum (SiC), graphite, maucherite (~Ni_3_As_2_).
C. Wagner and W. Schottky proposed in 1931 three types of disorder for mixed
phases, namely substitution (Substitution), interstitial occupation
(Zwischengitterbesetzung), and formation of voids (Lückenbildung). However
after the study of several mixed crystals and especially the silver halogenides
and chalcogenides, F. L. suggested a division into complete and incomplete
crystals, the latter with the subdivision of addition, subtraction, and
division crystals again subdivided by the term multiplication (i.e., different
coordination for the same atom). Consequently the decomposition and the
recrystallization of glasses (with the composition NaO.3TiO_2_.8SiO_2_,
Na_2_O-TiO_2_-SiO_2_, and Li_2_-CeO_2_
(CeO_15_)-SiO_2_] was investigated by electron microscope and
electron diffraction. In several periods F. L. tried to answer the question "what is a
crystal" ending up in the statement: "Ich hoffe, . . . gezeigt zu
haben, daß es schwierig bzw. unmöglich ist, die Frage 'Was ist ein Kristall mit wenigen Worten zu
beantworten, sobald man den Boden der mathematischen Definition 'Idealkristall'
verläßt."

A
complete change in the direction of his scientific efforts occurred when he
went to the University of Chicago in 1948 as a "paperclip specialist of
the Navy" to investigate order-disorder in silicates, a field he had begun
to research for a thesis in Marburg. He became so involved working with his own
hands using the microscope and the Precession camera for X-rays that he forgot
to finish the last papers about structures of intermetallic compounds [Mg_2_Ga(In,Tl)
and Mg_5_Ga_2_(Tl_2_)].

His
activities on feldspar continued during the following three decades. New
methods like IR, NMR, ESR and electron microscopy in different variations
[PhEEM, SEM, TEM(s) and with polarization] allowed expanded knowledge of a most
important rock forming mineral. The first investigations dealt with K-feldspar
(sanidine, orthoclase, mikrocline, adularia) and Na-feldspar (high-low),
showing their optical and structural behavior and different types of twinning,
the exsolution in moonstone, and phase relations of the alkaline feldspars.
Julian R. Goldsmith, colleague and friend, cooperated over a decade with F. L.
in the feldspars, their long/short-range order, their use as geological
thermometer, the intermediate types of K-feldspar, the order phenomena in
partially replaced Al/Si by Ga/Ge in anorthite, the effect of temperature and
composition on the Al/Si distribution, the coexistence of two plagioclases in
the oligoclase region. After structure determination of anorthite and celsian
by Helen Megaw et al. a controversial discussion with F. L. and J. R. Goldsmith
took place concerning the definition of displacive transformation, domains and
boundaries in between, their influence of order/disorder problems of Al/Si in
the framework, the importance of Ca for low temperature structure and the
usefulness of doubling the *c_0_* constant (14 Å), also in the
alkalifeldspar region. The possibility of an intermediate state for orthoclase,
between sanidine and microcline, was also discussed.

In
1954 F. L. accepted the Chair of Mineralogy at the ETH in Zürich as successor
to P. Niggli, where he remained till his emeritation inspite of an offer as
director of the Fritz Haber Institut of the Max Planck Society, Berlin. With
the support of colleagues in chemistry, H. H. Günthard, and the physicists E.
Brun and F. Waldner, he introduced the IR-spectroscopy and the methods of
paramagnetic nuclei and electron resonance into the fields of feldspars and
other compounds. With the aid of St. Hafner he confirmed the order/disorder
statements and the intermediate stages of orthoclase etc. by IR and NMR. A
comparison of the results received by these two methods on the feldspar problem
are given.

His
other researches in feldspars include the following. The temperature dependence
of domains and the X-ray intensities of anorthite were in­vestigated in detail.
The schiller and submicroscopic lamellae in labradorite, peristerite and
moonstone were studied with an electromicroscope (also in transmission and as
photoemission). An important part of the efforts on feldspars was in
application to geological problems and on lunar samples. After the numerous
papers on plagioclases and a careful study of J. V. Smith's book
"Feldspars", Vol I. and II., F. L. gave his paper "Gedanken zum
stabilen Phasendiagramm der Plagioklase", at the joint meeting of the
Österreichische Mineralogische Gesellschaft and the Schweizer
Mineralogisch-Petrographische Gesellschaft in Salzburg, September 1977. He
showed a more simplified diagram with one exsolution curve and four maxima as a
summation summary of thirty years effort in this field.

F.
L. also conducted work on quartz. Unpublished results of electrolysis in quartz
with Au- and Ag-anodes in 1939 were the starting point twenty years later of a
study of the mechanism at work during electrolysis, the diffusion of cations
(Au ^+^, Ag ^+^, Cu^+^, Li ^+^, H^+^)
in connection with elastical oscillation, changes of refractive indices,
structural and defect channels in the structure, and color centers. IR
absorption bands, especially in the region ~ 3000 cm^-1^ are due to
OH-vibrations and permit an explanation of the behavior of H in its dependence
on temperature, thermoluminiscence, lamellae, and geological environment.
Traces of Na in quartz were estimated by neutron activation analysis and by
diffussion experiments without a D.C. field followed. A further method - the
electron paramagnetic re­sonance - was introduced to observe defects in quartz
crystals with color centers obtained by electrolysis. A1PO_4_ in the
low-quartz modifi­cation has been investigated in respect to the electrical
quadrupol exchange of Al^27^. The structure of the subcell of Mg[Al_2_Si_3_O_10_]
has been found to be similar to the high-quartz modification with the position
of Mg in structural channels parallel *c_0_*.
The polytypy and polytropy of tridymite has been discussed in respect to its
superperiods in *c_0_* and in respect to the disappearance of
reflections far above (180°-230°) the transition tempera­ture of 170°.

IR
absorption spectra and nuclear magnetic resonance of Al^27^ were
applied to order/disorder problems in Al-spinels and A1_2_O_3_;
NMR of Na^23^, Al^27^, and Si^29^ to cation order in
nepheline and Cr in spinels. Starting in 1960 with the publication of an
inverse trirutile type Cr_2_WO_4_ numerous representatives of
oxides with TiO_2_-types were synthesized and X-ray examined, as well
as representatives of the spinel, perovskite, columbite, *alpha*-Mn_2_O_3_
type. The structural relations between alpha-PbO_2_, wolframite (FeWO_4_),
columbite (FeNb_2_O_6_) and brookite (TiO_2_)
were shown in detail. New phases of TiO*_x_* 1< *x*<
2 were be found. Especially the tantalite (ATaO_4_) and niobate (ANbO_4_)
and their binary mixed crystals belonged to the *alpha*-PbO_2_,
wolframite, AlNbO_4_, pyrochlore, and BiTaO_4_ types. Single
crystals of the oxides Nb_2_O_5_ and Ta_2_O_5_
have been grown and their different polytypes have been investigated showing
the different possibility of octahedral connection with the composition A_2_O_5_.
The crystal chemistry of tellurium oxides and their complexes as Te^4+^
and Te^6+^ has been reported within the new structure type Mg_3_TeO_6_.
Average and superstructure of *beta*-eucryptite (LiAlSiO_4_)
has been determined by X-ray.

In
addition to his continuous interest in feldspars, he intended to write a small
book on "twinning and ferroelasticity" and another one comparing the
symbolism on "Bauverbände" with that created by C. Hermann (1960) based on the nomenclature for lattice complexes.

Finally
it should be mentioned that F. L. worked also in the field of organic
chemistry, namely about urea and thiourea adducts, the structures of
nicotinacidamid, squalene, pigment substances and biantraquinone.

In a talk given in 1958 reviewing
the development of crystallography during the last 50 years (an anniversary of
the "Deutsche Mineralogische Gesellschaft") F. L. stated: "Die
Laue'sche Entdeckung - in Deutschland geboren - ermöglichte eine
Kristallstrukturforschung. Deren Bedeutung für alle Zweige der
Naturwissenschaften und Technik ist uns allen geläufig. Praktisch betrieben
wurde sie aber, und wird sie auch heute noch, vorwiegend im Ausland",
(This has changed in a few fields in the last two decades). Two
years later in an obituary of Max von Laue he reviewed the history of
crystallography: M. v. Laue's traditional hypothesis about lattices in crystals
which was supported by Leonhard Sohncke till 1897 and continued by Paul Groth;
the discussion by P. P. Ewald, a doctorand of Sommerfeld, who thought about the
reaction of X-rays when passing lattices occupied by atoms; and the willingness
of Friedrich and Knipping to do the proposed experiments.

F.
L. succeeded in establishing in Zürich cooperation with colleagues in other
fields to study the physics and chemistry of minerals and to gain a deeper
understanding of order/disorder problems, twinning, and geological
significance. In this respect he was an excellent teacher to students and post­doctoral
fellows. Twenty of his students and assistants later became professors at
universities and continue his research interests.

F. L. was
chairman of the "Deutsche Mineralogische Gesellschaft" (DMG)
1956-1958, member of the Executive Committee of IUCr (1960-1966) and its
vice-president, member of the Akademie der Naturforscher "Leopoldina" Halle,
corresponding member of the "Akademie der Naturwissenschaften und
Literatur in Mainz" and of the "Bayerische Akademie der
Wissenschaften in München". He received the doctor honoris causa from the
University of Bochum, the Roebling medal of the Mineralogical Society of
America, and the Abraham-Gottlieb-Werner medal of the DMG. In 1971 he became a
honorary member of the DMG as well as of the "Österreichische
Mineralogische Gesellschaft".

His affiliation with the Zeitschrift
für Kristallographie is long-standing. When he was 27 years old he prepared the
main register for volumes 51 through 75 (particularly of authors). With vol.
106 he became Editor of the Journal. Fritz Laves considered mineralogy as an
important part of natural science based on crystallography, physics, and
chemistry; in this sense he acted as an editor of this Journal for which he has
won the thanks of the editors and the staff of the Akademische
Verlagsgesellschaft. The Journal's name in thus reflects his philosophy.
Publications in the field of crystallography, intermetallic compounds,
feldspars, quartz and applied research methods will be indebted to the basic
research and results of Fritz Laves for many years to come.

*Nachruf
von Erwin Hellner in Z. Krist. **151**, 1980 (ohne die umfangreiche
Bibliographie)*

  

|  |  |  |
| --- | --- | --- |
| *Mathematische Kristallographie in Marburg* | *Elke Koch* | *Werner Fischer* |

*Stand: Mai 2001*
