# Supplementary material for: Space groups and crystallographic symmetry: writing a multi-featured tutorial in a new style
Source: Acta Crystallogr E Crystallogr Commun. 2021 Jul 16;77(Pt 9):857–63. doi: 10.1107/S2056989021007039 (PMC8423017; doi:10.1107/S2056989021007039)
Supplement: Supplementary file 1 [file e-77-00857-sup2.zip › symandsg/Main/lonsdale.htm]

CWP at physics.UCLA.edu//Kathleen Lonsdale


|  |  |
| --- | --- |
| **Welcome to CWP at UCLA** |  |

**86 Eminent Physicists** 

**Search the Archive**

**Fascinating Documents**

**Annotated
Photo
Gallery**

**In
Her Own Words**

**Some Physics History**

**500+ Books and Articles**

**Project Staff**

**Field Editors**

**Project Support**

**Photo Credits**

**Copyright Notice**

#### Crystallography

|

|  |

|  |  |  |
| --- | --- | --- |
| Contributions  ---  Publications  ---  Honors  --- |
 Kathleen Yardley Lonsdale    1903-1971 | Jobs/Positions  ---  Education  ---  Additional Information  --- |

 **"Her experimental determination of the structure of the benzene ring by
x-ray diffraction, which showed that all the ring C-C bonds were of the
same length and all the internal C-C-C bond angles were 120 degrees, had an
enormous impact on organic chemistry."**

-- Professor K. N. Trueblood, UCLA.

### Some Important Contributions

**Confirmed experimentally the ring structure of benzene** , conjectured
by Kekul� in 1865. She not only demonstrated the ring structure but
showed that the molecule is
planar and hexagonal
- and gave its precise dimensions.

**"Kathleen Lonsdale had a profound influence on the development of X-ray
crystallography and related fields in chemistry and physics. Very few have
made so many important advances in so many different directions."**  

-- J. M. Robertson,
[dsb1981jr]


Contributions to X-ray crystallography:

"As a physicist and mathematician by training, Kathleen Lonsdale's first
major contribution was a  **profound and systematic study of the theory of
space groups** , methods for their determination, and the possibilities of
molecular symmetry that are involved.
This work was published with W. T. Astbury in 1924. ... A later continuation
and extension of this work consisted of the structure factor tables published
in 1936 ..., and the many volumes of the International Tables for X-Ray
Crystallography published from 1952 onwards. These volumes, of which Kathleen
Lonsdale was principal editor, are in constant use today and form an
essential tool for crystal structure determination.
" -- J. M. Robertson, ibid.

Work on physics of crystals:

"She made an intensive study of the magnetic anisotropy of crystals, and
**by measuring the diamagnetic susceptibilities in and perpendicular to the planes
of a number of aromatic molecules, she was able to show that, while
sigma electronic orbits were of atomic dimensions, the pi orbits were of
molecular dimensions.** The far reaching importance of this result was that
it established the reality of the concept of molecular orbitals."
-- ibid.

Demonstrated that diffuse X-ray reflection by single crystals is
directly related to the elastic constants of the crystal and can be
used to determine molecular orientation.

Development of divergent-beam X-ray
photography of crystals:

"She was responsible for the development of divergent-beam X-ray photography of
crystals, a technique which gives information about the texture and
perfection of the crystal, and also can be used to make precise measurements
of lattice constants or wavelength. By this means she was able to measure
the C-C distance in individual diamonds to seven significant figures. She
had a special interest in the structure of diamonds and made many other
important contributions to their study."

-- J. M. Robertson, [dsb1981jr]

### Some Important Publications

 ***Papers*** 

"The Structure  of the Benzene Ring in Hexamethylbenzene," *Proceedings of the Royal Society* 123A: 494 (1929).

"An X-Ray Analysis of the Structure of Hexachlorobenzene, Using the Fourier Method," *Proceedings of the Royal Society* 133A: 536 (1931).

"Tabulated Data for the Examination of the 230 Space-groups by Homogeneous X-Rays," *Philosophical Transactions of the Royal Society* 224A: 221 (1924) with W. T. Astbury.

"Magnetic Anisotropy and Electron Structure of Aromatic Molecules," *Proceedings of the Royal Society* 159A: 149 (1937).

"An Experimental Study of Diffuse X-Ray Reflection by Single Crystals," *Proceedings of the Royal Society* 179A: 8 (1941).

"Diamonds, Natural and Artificial," *Nature* 153: 669 (1944).

"Divergent Beam X-ray Photography of Crystals," *Philosophical Transactions of the Royal Society* 240A: 219 (1947).

***Texts***

*Simplified Structure Factor and Electron Density Formulae for the 230 Space Groups of Mathematical Crystallography*, G. Bell & Sons, London, 1936.

*Crystals and X-Rays*, G. Bell & Sons, London, 1948.

*International Tables for X-Ray Crystallography*; vol. I (with N.F.M. Henry), vol. II (with J. Kasper), vol. III (with C.H. MacGillavry and G.D.
Rieck; Kynoch Press, Birmingham; 1952, 1959, 1962.

### Honors (partial list)

Fellow of The Royal Society (London) 1945

*The Society was founded in 1660. Kathleen Lonsdale and
microbiologist Marjory Stephenson were the first women Fellows; elected in 1945.*

Dame Commander of the Order of the British Empire 1956

Davy Medal of The Royal Society (London) 1957

General Secretary, British Association for the Advancement of Science 1959-64

Rare meteoric diamond named Lonsdaleite in her honor.

National University of Ireland, Maynooth, Co. Kildare established the Lonsdale Prize in Chemistry.
*[Lonsdale was born in Newbridge, Co. Kildare.]*

Honorary Doctorates:   
 Awarded by Universities of Wales, Leicester, Manchester,
Lancaster, Leeds, Dundee, Oxford, and Bath.

### Jobs/Positions

1922-23 Research Assistant to William H. Bragg, University College, London
  
1923-27 Research Assistant to William H. Bragg, The Royal Institution, London
  
1927-30 Amy Lady Tate Scholar and part-time demonstratorship, Leeds University
  
(Between 1929 and 1934, Lonsdale gave birth to three children, and continued her research at home.)  
1934 The Royal Institution  
1935-37 Leverhulme Research Fellow, The Royal Institution, London  
1944-46, Dewar Fellow, The Royal Institution, London  
1946-49 Reader in Crystallography, University College, London.(
Founded Crystallography Group.)   
1947 Special Fellow of the United States Federal Health Service  
1949-68 Professor of Chemistry and Head of the Department of Crystallography, University College, London  
1968-71 Professor Emeritus, University College, London

### Education

B.S. Bedford College for Women 1922  
M.S. University College, London 1924  
D.Sc. University College, London 1936

### References

[bmfrs1975dh], [dsb1981jr], [iss1970kl]


### Additional Information/Comments

Lonsdale generously gave scientific assistance and encouragement to
colleagues and coworkers. For example, she
encouraged and assisted Nobel Laureate
Dorothy Crowfoot Hodgkin  in the work on the structure of penicillin,
one of the discoveries which led to her Nobel Prize. Professor Hodgkin wrote about Dame Lonsdale [bmfrs1975dh]:

"..there are many of her interventions in scientific research
that are unpublished, in the memories of all of us who knew and worked
with her. One such, I should record, involved my own research. It was
Kathleen Lonsdale who encouraged Sir Henry Dale to send to the Squibb
Research Institute for sodium benzylpenicillin for my researches. And
the first test of the X-ray microscope developed by W. L. Bragg and
Charles Bunn was actually made at the Royal Institution by Kathleen
Lonsdale herself to assist structure factor calculations for the
penicillin crystals--a pilot operation which was later successfully
extended in the actual X-ray analysis that defined the penicillin
structure."

On the difficult task of balancing a career and family, Dame Lonsdale wrote:

"Sir Lawrence Bragg once described the life of a university
professor as similar to that of a queen bee, nurtured, tended and cared
for because she has only one function in life. Nothing could be farther
from the life lived by the average professional woman." [iss1970kl].

A major priority for Lonsdale was
encouraging young people to enter science. She helped found the Young
Scientists' section of the British Association for the Advancement of
Science, and made the following note to herself: "Never refuse an
opportunity to speak at schools." [bmfrs1975dh]

On women in science, Dame Lonsdale wrote:

"Any country that wants to make full use of all its potential
scientists and technologists could do so, but it must not expect to get
the women quite so simply as it gets the men. It seems to me that
marriage and motherhood are at least as socially important as military
service. Government regulations are framed to ensure (in the United
Kingdom) that a man returning to work from military service is not
penalized by his absence. Is it Utopian, then, to suggest that any
country that really wants married women to return to a scientific
career when her children no longer need her physical presence should
make special arrangements to encourage her to do so?"[iss1970kl]

As a form of protest against World War II, Dame Lonsdale didn't
register for civil defense duties and refused to pay the �2 fine. As a
result, she was sentenced to one month in Hollaway Prison for Women.
Lonsdale's first-hand experience made her a vocal advocate of prison
reform. She was Member of Board of Visitors, Aylesbury Prison for Women
and Borstal Institution for Girls; and Deputy Chairman of the Board of
Visitors, Bullwood Hall, Borstal Institution for Girls.

She was a Quaker and an active spokesperson for world peace supporting the
Pugwash Movement and serving as Vice-President of the Atomic Scientists
Association and President of the Women's International League for Peace and
Freedom.

She travelled extensively lecturing and attending meetings in over 25 countries.

Married Thomas Lonsdale in 1927 and they had three children - Jane, Nancy and Stephen.

**A list of firsts:**

First woman in 1945, along with microbiologist Marjory Stephenson, to be elected
Fellow of The Royal Society (London).  
First female professor, University College, London.   
First woman president of the International Union of Crystallography (1966).  
First woman president of the British Association for the Advancement of Science (1967).

#### Field Editor: Professor K. N. Trueblood

 Copyright � CWP and Regents of the University of California 1996 - 2002.

***To cite this citation:***  
" Lonsdale, Kathleen." *CWP*   
< http://www.physics.ucla.edu/~cwp>

**BACK TO THE TOP**


---
