# Supplementary material for: Space groups and crystallographic symmetry: writing a multi-featured tutorial in a new style
Source: Acta Crystallogr E Crystallogr Commun. 2021 Jul 16;77(Pt 9):857–63. doi: 10.1107/S2056989021007039 (PMC8423017; doi:10.1107/S2056989021007039)
Supplement: Supplementary file 1 [file e-77-00857-sup2.zip › symandsg/Main/mauguin.pdf]

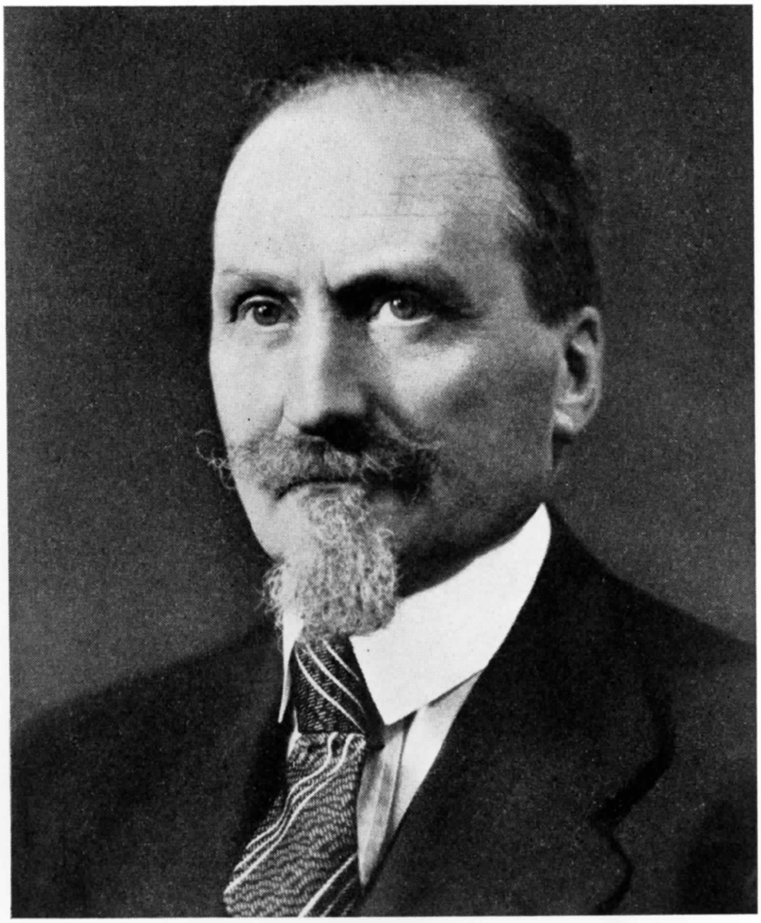

CHARLES MAUGUIN
1878—1958

Charles Mauguin est mort d'un cancer le 25 avril dernier à l'Institut Gustave Roussy où il était entré un mois plus tôt. Il aurait eu, en septembre prochain, quatre-vingts ans. Jusqu'à ses tout derniers jours, il a conservé son entière lucidité et un intérêt passionné pour la science.

Depuis la création des *Acta Crystallographica*, il faisait partie de son 'Advisory Board'. Ch. Mauguin est le fondateur de l'école française de cristallographie moderne. Par ses travaux, son enseignement, il a su attirer vers la cristallographie de nombreux élèves.

Il avait commencé sa carrière scientifique au laboratoire de chimie organique de l'Ecole Normale Supérieure et sa thèse de doctorat, soutenue en 1910, se rapportait à la transformation des amides en uréides. C'est Pierre Curie qui, dans son enseignement, lui donna la goût de la cristallographie. Il devint, en 1910, assistant de Wallerant au laboratoire de minéralogie de la Sorbonne où il revint, après un court séjour aux Facultés des Sciences de Bordeaux et de Nancy, à la fin de la première guerre mondiale, d'abord comme maître de conférences, puis comme Professeur titulaire de la chaire quand Wallerant prit sa retraite en 1933.

Dès 1910, Mauguin attirait sur lui l'attention du monde scientifique par ses travaux sur les cristaux liquides. Les conclusions du physicien allemand Otto Lehmann concernant la biréfringence de ces substances étaient fortement controversées. Mauguin, en orientant le liquide par un champ magnétique, ou par les parois de lames de verre ou par des lames de mica, obtint

des préparations parfaitement transparentes, fit des mesures de biréfringence, expliqua les phénomènes observés. Ses résultats et ses techniques ont conservé tout leur intérêt et sont encore à la base des travaux qui se poursuivent sur ces substances si intéressantes des points de vue physico-chimique et biologique.

Mauguin avait tout de suite compris l'importance, pour la cristallographie et la minéralogie, des découvertes de Laue et de Bragg; dans cette période de pionniers qui s'étend de 1920 à 1930, il établit les structures atomiques du cinabre, du calomel, du graphite; il clarifie, en utilisant les données de l'analyse chimique et des rayons X, la chimie jusqu'alors incompréhensible des micas et des chlorites. Il fournit, à l'occasion de la publication, en 1935, des premières Tables Internationales pour la détermination des structures des cristaux, le nouveau symbolisme des groupes spatiaux qui a rendu le nom de Mauguin familier aux cristallographes du monde entier.

Esprit encyclopédique, très bon naturaliste, il s'intéressait aussi aux questions les plus théoriques où il pouvait faire apporter une contribution originale: sa dernière publication aux Comptes Rendus de l'Académie des Sciences, en 1952, se rapportait à l'Astronautique et à la Relativité et s'intitulait 'A l'assaut de l'Espace-temps'.

Ces dernières années, la chimie dans ses rapports avec la biologie, en particulier le problème de la genèse de la vie, occupait toutes ses pensées.

Charles Mauguin croyait passionnément à la science; son plus cher désir était de la voir toujours utilisée pour le bonheur de l'humanité.

J. WYART
