# Supplementary material for: Space groups and crystallographic symmetry: writing a multi-featured tutorial in a new style
Source: Acta Crystallogr E Crystallogr Commun. 2021 Jul 16;77(Pt 9):857–63. doi: 10.1107/S2056989021007039 (PMC8423017; doi:10.1107/S2056989021007039)
Supplement: Supplementary file 1 [file e-77-00857-sup2.zip › symandsg/Main/Megaw,_Helen851234567.html]

CWP at physics.UCLA.edu // Megaw


|  |  |
| --- | --- |
| **Welcome to CWP at UCLA** |  |

**86 Eminent Physicists** 

**Search the Archive**

**Fascinating Documents**

**Annotated
Photo
Gallery**

**In
Her Own Words**

**Some Physics History**

**500+ Books and Articles**

**Project Staff**

**Field Editors**

**Project Support**

**Photo Credits**

**Copyright Notice**

#### Crystallography

|  |  |  |
| --- | --- | --- |
| Contributions  ---  Publications  ---  Honors  --- |
 Helen D. Megaw   1907- | Jobs/Positions  ---  Education  ---  Additional Information  --- |

### Some Important Contributions

Comparison of the structures of ordinary and `heavy' ice.

With J.D. Bernal, published an early, and now classic, study of hydrogen bonding in metal hydroxides.

First determination of the crystal structure of barium titanate, BaTiO3 -
one of the most important ferroelectrics used widely in industry for its dielectric properties.

Discovered complexities in feldspar structures, and distinguished between unit cell and lattice disorders.

Impact on ferroelectricity theory through her research on titanates and perovskites.

"Helen's impact on ferroelectricity was profound, especially in the
early days. She brought to the subject a visual aspect embodied in the
crystal structures of ferroelectrics, thus showing how this important
effect arose. Her book, *Ferroelectricity in Crystals*, the first book on the
subject, was for a long time just about the only book on the subject
and became almost a "bible" for those working in ferroelectricity."
---- Professor A. Michael Glazer, University of Oxford.

### Some Important Publications

"Cell Dimensions of Ordinary and `Heavy' Ice," *Nature* 134: 900 (1934).

"The Function of Hydrogen in Intermolecular Forces," *Proceedings of the Royal Society of London* A151: 384 (1935), with J.D. Bernal.

"Crystal Structure of Barium Titanate," *Nature* 155: 484 (1945).

"Origin of Ferroelectricity in Barium Titanate and Other Perovskite-type Crystals," *Acta Crystallographica* 5: 739 (1952).

"Notation for Felspar Structures," *Acta Crystallographica* 9: 56 (1956).

"Order and Disorder. I. Theory of Stacking Faults and Diffraction Maxima," *Proceedings of the Royal Society of London* A259: 59 (1959).

"Order and Disorder. II. Theory of Diffraction Effects in the Intermediate Plagioclase Feldspars," *Proceedings of the Royal Society of London* A259: 184 (1959).

"Structures and Transitions in Perovskites," *J. de Physique* 33 (Proceedings of Conference at Dijon): C2-1 (1972).

"Studies of the Lattice Parameters and Domains in the Phase Transitions of NaNbO_3_," *Acta Crystallographica* A29: 489 (1973), with A.M. Glazer.

"The Architecture of Felspars," and "Tilts and Tetrahedra in Felspars," in MacKenzie & Zussman (eds.), *Proceedings of the NATO Advanced Study Institute on Felspars*. Manchester: Manchester University Press, 1974.

"Geometrical and Structural Relations in the Rhombodedral Perovkites," *Acta Crystallographica* A31: 161 (1975).

**Books:**

*Ferroelectricity in Crystals*. Methuen, London 1957.

*Crystallographic Book List*. International Union of Crystallography Commission, Utrecht 1965 (and 2 supplements (1966) (1972)).

*Crystal Structures: A Working Approach*. W.B. Saunders Co.,
Philadelphia 1973.

**Of historical interest:**

"The Domain of Crystallography," in J. Lima de Faria (ed.), *Historical Atlas of Crystallography*.
Kluwer Academic Publishers, Dordrecht 1990. "This is a survey
explaining the relationship of Crystallography to Physics and to
Chemistry--not of great interest in itself, but perhaps useful to
physicists looking back at historical developments." --- Helen D. Megaw

### Honors

Honorary Doctor of Science, University of Cambridge 1967

Honorary Doctor of Science, Queen's University, Belfast, Northern Ireland 2000
  ***Citation***

Fellow, Mineralogical Society of America

Fellow, Institute of Physics

Roebling Medal, Mineralogical Society of America 1989 [Megaw was the first woman to receive this medal.]

**The name Helen D. Megaw Island was given to an island in the Antartica, located at about 66.9� S, 67.7� W.** 
This honor was bestowed when the Glaciological Society was naming
features in that neighborhood after scientists who had done some
original work on ice. [See *Ice* 9: 10-11, 16 (1962).]

### Jobs/Positions

1934-35 Post-doctoral research with Prof. H. Mark, University of Vienna  
1935-36 Post-doctoral research with Prof. F. Simon, Clarendon Laboratory, Oxford University  
1936-43 Assistant Mistress at (1) Bedford High School and (2) Bradford Girls' Grammar School  
1943-45 Crystallographic scientist, Materials Research Laboratory, Philips Lamps Ltd., Mitcham  
1945-46 Assistant Director of Research in Crystallography, Birkbeck College, London  
1946-49 Assistant for Experimental Research in Crystallography,
Cavendish Laboratory, Cambridge  
1946-72 Fellow, Lecturer, and Director of Studies in Physical Science, Girton College, Cambridge  
1949-59 Assistant Director of Research in Crystallography, Cavendish Laboratory, Cambridge  
1959-72 Lecturer, Cambridge University  
1968-present Life Fellow, Girton College, Cambridge

Councilor, Mineralogical Society of Great Britain and Ireland 1971-74.

### Education

Queen's University, Belfast 1925-26  
B.S. Girton College, Cambridge 1930  
Ph.D. (mineralogy and petrology) University of Cambridge 1934  


### Additional Information/Comments

"Helen was a formidable person, as well as being very nice and
scrupulously honest as a scientist. She was talented in some rather
interesting ways. She was the only person I have met who could imagine
a crystal structure in her mind and turn it around, and then draw it
out for you on a piece of paper from any direction you cared to
choose!"

--- Professor Michael Glazer, Clarendon Laboratory, University of Oxford.

"Along with 
Kathleen Lonsdale  and
 Dorothy Hodgkin ,
Helen Megaw is one of the grand old school of British women crystallographers who serve
as role models for many of us - men and women alike."   
--- Professor Robert Newnham, Pennsylvania State University [hm1990rn]

"Much has been said about the difficulties of women in science, but I
would like
to say explicitly that I at least was never or rarely aware of
discrimination.
Perhaps I was particularly lucky, in that everyone who advised me on my
education and guided my career assumed that women should be given the
same opportunities as men. First and foremost I am thankful to my
parents
for this, and then to those far-sighted women of earlier generations
who
founded Girton College as a college for women, within Cambridge
University and
an integral part of it."   
---Helen Megaw, on accepting the Roebling Medal of the Mineralogical Society of
America for 1989. [am1990hm]

British Crystallographic Association
'Crystallography News'
December 2000 notice

  

#### Edited by:

Helen Megaw & Professor A. Michael Glazer, University of Oxford
  

#### Field Editor: Professor A. Michael Glazer, University of Oxford Copyright � CWP and Regents of the University of California 1997 ***To cite this citation:*** " Megaw, Helen." *CWP* < http://www.physics.ucla.edu/~cwp>

**BACK TO THE TOP**


---


Last revised 4/2/01 nb
