# Supplementary material for: Space groups and crystallographic symmetry: writing a multi-featured tutorial in a new style
Source: Acta Crystallogr E Crystallogr Commun. 2021 Jul 16;77(Pt 9):857–63. doi: 10.1107/S2056989021007039 (PMC8423017; doi:10.1107/S2056989021007039)
Supplement: Supplementary file 1 [file e-77-00857-sup2.zip › symandsg/Main/Membership.htm]

Membership

# ACA MEMBERSHIP

Membership
in the ACA is open to any person who is actively interested in the
purposes of the Association and whose application is approved by ACA
Council or its designee, upon payment to the Treasurer of such dues are
specified in the Rules. All members are entitled to voting privileges.
Student members are very welcome and their contributions to the life
and vigor of the Association have always been important. The 2006
annual dues are $24 for students, $36 for post docs, $90 for regular
members, and $39 for retired members. The benefits of membership are
the same in all categories. These include: voting privileges, the ACA
Newsletter that is published quarterly, Program and Abstract Book of
all annual meetings, complimentary subscription to the **ICUrNewsletter** also
published quarterly, reduced prices of the Transactions of the American
Crystallographic Association, and for the International Tables for
X-Ray Crystallography, Structure Reports, Journal of Applied
Crystallography, and Acta Crystallographica when purchased for the
member’s personal use only. Because the ACA is a member society of the
American Institute of Physics (AIP) members also receive *Physics Today*, a monthly publication of AIP.

The
total membership of the ACA is about 2,200.  Meetings are held
annually. There are 12 Special Interest Groups concerned with
Biological Macromolecules, Fiber Diffraction, General Interest,
Industrial, Materials Science, Neutron Scattering, Powder Diffraction,
Service Crystallography, Small Angle Scattering, Small Molecules,
Synchrotron Radiation and Young Scientists. ACA Members may join as
many of these that interest them. Each Special Interest Group is
responsible for organizing sessions at Annual Meetings at least every
other year.

## **Corporate Membership Benefits and Information**

The Association’s mailing address is:  ACA, P.O. Box 96 Ellicott Station, Buffalo, NY 14205-0096.

Telephone: (716) 898-8690, Fax: (716) 898-8695, aca@hwi.buffalo.edu,

## Membership Links

- Member Directory

- Update Address

- Membership Application & Dues Form

- Membership Benefits

- Corporate Membership

- Current Corporate Members

- 2005 Officers and Appointments

- 2006 ACA Annual Meeting

|  |  |  |  |
| --- | --- | --- | --- |
|  |  |  |  |
|  | | |  |
|  |  |  |  |
|  |  |  |  |

Current

Current

Past

Past

Communications

Communications

Continuing Education

Continuing Education

Data, Standards & Computing

Data, Standards & Computing

Past Committee Members

Past Committee Members

Duties

Duties

Biological Macromolecules

Biological Macromolecules

Fiber Diffraction

Fiber Diffraction

General Interest

General Interest

Industrial

Industrial

Materials Science

Materials Science

Neutron Scattering

Neutron Scattering

Powder Diffraction

Powder Diffraction

Service Crystallography

Service Crystallography

Small Angle Scattering

Small Angle Scattering

Small Molecule

Small Molecule

Synchrotron Radiation

Synchrotron Radiation

Young Scientists

Young Scientists

How to Form a SIG

How to Form a SIG

2006 SIG Officers

2006 SIG Officers

Canadian Division

Canadian Division

2005 SIG Reports

2005 SIG Reports

Available Publications

Available Publications

Order Form

Order Form

International Union of Crystallography

International Union of Crystallography

American Institute of Physics

American Institute of Physics

U.S. National Committee

U.S. National Committee

Crystallographic Resources

Crystallographic Resources

American Association on Crystal Growth

American Association on Crystal Growth

ACA Summer Courses

ACA Summer Courses

Ongoing Workshops & Schools

Ongoing Workshops & Schools

Other

Other

Education Web Sites

Education Web Sites

AIP Education & Student Services

AIP Education & Student Services

Career Opportunities

Career Opportunities

What is Crystallography

What is Crystallography


|  |  |  |  |  |  |  |  |  |  |  |  |  |  |  |  |  |  |  |  |  |  |  |  |
| --- | --- | --- | --- | --- | --- | --- | --- | --- | --- | --- | --- | --- | --- | --- | --- | --- | --- | --- | --- | --- | --- | --- | --- |
|  |  |  |  |  |  |  |  |  |  |  |  |  |  |  |  |  |  |  |  |  |  |  |  |
|  | | | | | | | | | | | | | | | | | | | | | | |  |
|  | | | | | | | | | | | | |  |  |  |  |  |  |  |  |  |  |  |
|  |  |  |  |  |  |  |  |  |  |  |  |  |  |  |  |  |  |  |  |  |  |  |  |
|  |  |  |  |  |  |  |  |  |  |  |  |  |  |  |  |  |  |  |  |  |  |  |  |
|  |  | | | | | | | | | | |  |  |  |  |  |  |  |  |  |  |  |  |
|  |  | | | | | | | | | | |  |  |  |  |  |  |  |  |  |  |  |  |
|  |  | | | |  | |  | | | | |  |  |  |  |  |  |  |  |  |  |  |  |
|  |  |  |  |  |  | |  |  |  |  |  |  |  |  |  |  |  |  |  |  |  |  |  |
|  |  | |  | | | | | | | |  |  |  |  |  |  |  |  |  |  |  |  |  |
|  |  |  |  | | | | | | | |  |  |  |  |  |  |  |  |  |  |  |  |  |
|  |  |  |  | | |  | | |  | |  |  |  |  |  |  |  |  |  |  |  |  |  |
|  |  |  |  |  |  |  | | |  |  |  |  |  |  |  |  |  |  |  |  |  |  |  |
|  |  |  | | | | | | | | | |  |  |  |  |  |  |  |  |  |  |  |  |
|  |  |  | | | | | | | | | |  |  |  |  |  |  |  |  |  |  |  |  |
|  |  |  | |  | | | | | |  | |  |  |  |  |  |  |  |  |  |  |  |  |
|  |  |  |  |  | | | | | |  |  |  |  |  |  |  |  |  |  |  |  |  |  |
|  |  |  |  |  |  | | |  | |  |  |  |  |  |  |  |  |  |  |  |  |  |  |
|  |  |  |  |  |  | | |  |  |  |  |  |  |  |  |  |  |  |  |  |  |  |  |
