# Supplementary material for: Space groups and crystallographic symmetry: writing a multi-featured tutorial in a new style
Source: Acta Crystallogr E Crystallogr Commun. 2021 Jul 16;77(Pt 9):857–63. doi: 10.1107/S2056989021007039 (PMC8423017; doi:10.1107/S2056989021007039)
Supplement: Supplementary file 1 [file e-77-00857-sup2.zip › symandsg/Main/mem-ship.htm]

Society of Crystallographers in Australia and New Zealand (SCANZ) - Membership

[Back to SCANZ Homepage |
What's New |
Contact Information |
SCANZ Conferences and Events]

---

#### (formerly: Society of Crystallographers in Australia (SCA))

## Membership

The **SCANZ** homepage is
located at **http://www.sca.asn.au**

---

To join the SCANZ, two current members of the Society must
sponsor the application. Please write to the Secretary if you do
not know any sponsors.

Cost of Membership depends on your catagory and how
good you are at sending the cheques in on time:

```
Regular  ($25)    Student ($7)   Corporate ($130)
```

- These amounts are reduced to $20, $5 and $100, respectively,
  if paid by April 1. Membership is on a calendar year basis.
  Applications received after June 30 will apply for the following year unless otherwise
  requested.
- Therefore, to join the Society the appropriate
  fees are $20, $5 or $100, respectively.

Fill in our **membership application** form and mail
to the SCANZ treasurer with a cheque for the correct amount payable to
SCANZ.

**Click Here!** to view, then print out the
SCANZ Membership application form.

## Secretary:

G.B. Jameson (Massey University, New Zealand),

**Tel:** +64 (6) 350-4431  
**Fax:** +64 (6) 350-5682  
**E-mail:**
**G.B.Jameson@massey.ac.nz**  
**Postal Address:** Institute of Fundamental Sciences, Massey University,
Palmerston North, New Zealand

## Treasurer:

B.Kobe (University of Queensland, Qld),

**Tel:** (07) 3365-2132  
**Fax:** (07) 3365-4699  
**E-mail:**
**b.kobe@uq.edu.au**  
**Postal Address:** School of Molecular and Microbial Sciences,
University of Queensland, Brisbane, Australia.  
  

---

**Go Direct!**  

[Back to SCANZ Homepage]
  
[About the SCANZ |
SCANZ Executive |
What's New |
Contact Information]
  
[Sketches of Laboratories |
SCANZ Newsletters]
  
[Membership |
SCANZ Conferences and Events |
Constitution |
Educational Material]
  
[SCANZ/IUCr Related Websites |
Other Web Sites |
Australasian Labs |
Jobs]


---

Please feel free to send any queries, comments or suggestions to:
**bws@crystal.uwa.edu.au**
