# Supplementary material for: Space groups and crystallographic symmetry: writing a multi-featured tutorial in a new style
Source: Acta Crystallogr E Crystallogr Commun. 2021 Jul 16;77(Pt 9):857–63. doi: 10.1107/S2056989021007039 (PMC8423017; doi:10.1107/S2056989021007039)
Supplement: Supplementary file 1 [file e-77-00857-sup2.zip › symandsg/Main/miller.html]

Chris Miller, Brandeis Univesity Biochemistry faculty member

Brandeis Search

**Home**
> Faculty

|  |  |  |
| --- | --- | --- |
|  | **Christopher Miller, Ph.D.** Professor of Biochemistry  Investigator, Howard Hughes Medical Institute  Structure and Mechanism in Ion Channel Proteins   Ph.D., University of Pennsylvania | |
|  | contact information   (781) 736-2340 | |

> Our work here is aimed at understanding the molecular
> and structural underpinnings of the generation of cellular
> electricity. All such phenomena - from the nerve action
> potential, to sensory transduction, to control of processes
> as varied as muscle contraction, hormone secretion, or blood
> volume homeostasis - are ultimately mediated by a single
> class of membrane proteins: the ion channels. We seek to
> understand the molecular mechanisms by which ion channel
> proteins open and close to switch the flows of ions across
> cellular membranes, and by which the open pore is able to
> choose so exquisitely which ions are able to permeate.
>
> The laboratory focuses on a broad class of ion channels
> - the CLC-type Cl- channels. These proteins are interesting
> because so little is known about them, despite the fact
> that high-resolution x-ray crystal structures are now known
> for some. The relationship between structure and functional
> behavior is an area of active investigation here, using
> bacterial CLC homologues and a combination of x-ray crystallography
> and electrophysiological analysis.
>
> We recently discovered, to our shock and awe, that a bacterial
> homologue of this Cl- channel family is not itself an ion
> channel, but rather functions as an ion "pump," stoichiometrically
> exchanging Cl- on one side of the membrane for H+ on the
> other. Using a combination of electrophysiology, membrane
> reconstitution, and x-ray crystallography, we are endeavoring
> to understand how these ransport proteins work and also
> to comprehend the wider mechanistic implications of this
> co-habitation within the same molecular family of such fundamentally
> different ion-transport mechanisms.

> **Selected Publications**
>
> Kobertz, W.R., Williams, C., and Miller, C. 2000. Hanging gondola
> structure of the T1 domain in a Shaker K+ channel. **Biochemistry**
> 39:10347-10352. [abstract
> in PubMed]
>
> Mindell, J.A., Maduke, M., Miller, C., and Grigorieff, N. 2001.
> Projection structure of a ClC-type Cl- channel at 6.5 � resolution.
> **Nature** 409:219-223. [abstract
> in PubMed]
>
> LeMasurier, M., Heginbotham, L., and Miller, C. 2001. KcsA: It's
> a potassium channel. **J. Gen. Physiol.** 118:303-314. [abstract]
>
> Iyer, R., Iverson, T.M., Accardi, A., and Miller, C. 2002. A
> biological role for prokaryotic ClC Cl- channels. **Nature**
> 419:715-718. [abstract]
>
> Nimigean, C.M., Chappie, J.S., and Miller, C. 2003. Electrostatic
> tuning of ion conductance in K+ channels. **Biochemistry**
> 42:9263-9268. [abstract]
>
> Accardi, A. and Miller, C. 2004. Secondary active transport mediated
> by a prokaryotic homologue of CLC Cl- channels. **Nature**
> 427:803-807. [abstract]
>
> Nimigean, C.M., Shane, T., and Miller, C. 2004. A cyclic nucleotide
> modulated prokaryotic K+ channel. **J Gen Physiol.** 124:203-210.
> [abstract]
>
> Posson, D.J., Ge, P. Miller, C. Bezanilla, F., and Selvin, P.R.
> 2005. Small vertical movement of a K+ channel voltage sensor measured
> with luminescence energy transfer. **Nature** 436:848-851.
> [abstract]
>
> Miller, C. 2006. CLC chloride channels viewed through a transporter
> lens. **Nature** 440:484-489.
>
> View Complete Publication List on PubMed:
> Chris
> Miller
>
> ---
>
> Last reviewed: March 22, 2006. E-mail comments
> or questions to the webmaster.

Top
of Page | Life
Science Faculty | Home
