# Supplementary material for: Space groups and crystallographic symmetry: writing a multi-featured tutorial in a new style
Source: Acta Crystallogr E Crystallogr Commun. 2021 Jul 16;77(Pt 9):857–63. doi: 10.1107/S2056989021007039 (PMC8423017; doi:10.1107/S2056989021007039)
Supplement: Supplementary file 1 [file e-77-00857-sup2.zip › symandsg/Main/monoc-gif.html]

Draft


# Monoclinic lattices

#### a_1_ ≠ a_2_ ≠ a_3_

#### α = γ = 90^o^ ≠ β


|  |  |
| --- | --- |
| Simple monoclinic    Click on the picture to repeat animation   Go back | Base centered monoclinic    Click to see animation |
