# Supplementary material for: Space groups and crystallographic symmetry: writing a multi-featured tutorial in a new style
Source: Acta Crystallogr E Crystallogr Commun. 2021 Jul 16;77(Pt 9):857–63. doi: 10.1107/S2056989021007039 (PMC8423017; doi:10.1107/S2056989021007039)
Supplement: Supplementary file 1 [file e-77-00857-sup2.zip › symandsg/Main/node2.html]

The Patterson Function

  
 **Next:** Introduction
 **Up:** chapter6
 **Previous:** Contents
   **Contents**
  
  


# **The Patterson Function**

  

---


**Subsections**

- Introduction
- The Patterson function
  - *Periodicity of the Patterson function*
  - *The heavy atom method*
  - *Overlap of interatomic vectors*
- Classification of Patterson vectors
- Symmetry of the Patterson function
- Harker peaks
- Overview of Advanced Applications
  - *Superposition methods*
  - *Rotation functions*
  - *Translation functions*
  - *Molecular replacement*
- **Exercises**

  

---


Uri Shmueli
2003-09-03
