# Supplementary material for: Space groups and crystallographic symmetry: writing a multi-featured tutorial in a new style
Source: Acta Crystallogr E Crystallogr Commun. 2021 Jul 16;77(Pt 9):857–63. doi: 10.1107/S2056989021007039 (PMC8423017; doi:10.1107/S2056989021007039)
Supplement: Supplementary file 1 [file e-77-00857-sup2.zip › symandsg/Main/node3.html]

Space Group Operations

 


---

**Next:** Introductory Examples of Space Group Patterns
**Up:** Space Group Patterns
 **Previous:** Two-Dimensional Periodic Patterns

# Space Group Operations

Space groups comprise two types of symmetry operations:

(a) purely translational operations expressed by the Bravais lattice (denoted
by a capital letter in the space group symbol), and

(b) operations of point symmetry elements, glide planes and/or screw axes, as
listed in the following table:

|  |  |  |
| --- | --- | --- |
| --- | | |
| Symmetry element | Point symmetry elements | Symmetry elements involving translations |
| --- | | |
| of the first kind | *rotation axes* | *screw axes* |
| (proper) | 1 2 3 4 6 | 2_1_ 3_1_ 3_2_ 4_1_ 4_2_ 4_3_ etc |
|  | | |
| of the second kind | *rotary inversion* | *glide planes* |
| (improper) | *axes* | *a b c n d* |
|  |  |  |
| --- | | |

  

These symmetry operations can be depicted as shown in Figs. 2 and 3 (if
necessary, see also Fig. 4 for explanation of the diagrams). Symmetry
operations of the second kind, unlike those of the first kind (or proper
operations), transform a right-handed into a left-handed object and vice versa.
Two enantiomeric objects can be related only by a symmetry operation of the
second kind (or an improper operation). This distinction of the two kinds of
symmetry elements is also of considerable practical importance, as it will soon
be evident, in recognizing the space group of a given pattern.

**Figure 2:**
Symmetry operations of the first kind (examples).

  

**Figure 3:**
Symmetry operations of the second kind.

  

**Figure 4:**
Scheme used in space group patterns (worksheets (i) to (xv)).

  

Some point symmetry operations (which leave at least one point in space
unaffected) are shown on the left hand side of Figs. 2 and 3. Examples of
operations containing translational components (which are incompatible with a
finite array of objects) are presented on the right hand side in these figures.

More details on crystallographic symmetry elements can be readily obtained from
most textbooks on crystallography.^4^ For information on symbols and notation
it is best to consult the *International Tables* .^5^

---


   
 **Next:** Introductory Examples of Space Group Patterns
**Up:** Space Group Patterns
 **Previous:** Two-Dimensional Periodic Patterns


**Copyright © 1984, 1998 International Union of
Crystallography**

 IUCr
Webmaster
