# Supplementary material for: Space groups and crystallographic symmetry: writing a multi-featured tutorial in a new style
Source: Acta Crystallogr E Crystallogr Commun. 2021 Jul 16;77(Pt 9):857–63. doi: 10.1107/S2056989021007039 (PMC8423017; doi:10.1107/S2056989021007039)
Supplement: Supplementary file 1 [file e-77-00857-sup2.zip › symandsg/Main/nvk.htm]

NVK Nederlandse Vereniging voor Kristallografie

|  |  |  |  |  |
| --- | --- | --- | --- | --- |
|  | | | | |
| **informatie** | **werkgroepen** | **vacatures** | **links** |  |

# Nederlandse Vereniging voor Kristallografie

(bijgewerkt op 27 juni 2006)

---

## Recent

- Nieuwsbrief 2006/02 (23 juni 2006)
- Poederprijsvraag 2006 (23 juni 2006)
- Vacature Technical Support Engineer - X-ray diffraction facilities TU Delft (22 juni 2006)

---

## Informatie over de vereniging

|  |  |  |
| --- | --- | --- |
| - Agenda en activiteiten    (bijgewerkt op 23 juni 2006) - Voorgaande jaren - Nieuwsbrieven | - Doelstelling - Bestuur - Lidmaatschap - Mailing list | - KNCV-prijs voor de Kristallografie |

---

## Werkgroepen

- Poederdiffractie
  (27 maart 2006)
- Eiwitkristallografie
  (17 juni 2005)
- Onderwijs in de kristallografie
  (14 oktober 2005)

---

## Vacatures

- Technical Support Engineer - X-ray diffraction facilities TU Delft (22 juni 2006)
- Projectleider Avantium (12 december 2005)

---

## Verwijzingen

- IUCR: International Union of Crystallography
  - 7th Ewald Prize to Professor P. Coppens
- ECA: European Crystallographic Association
- KNCV: Koninklijke Nederlandse Chemische Vereniging
- NVV: Nederlandse Natuurkundige Vereniging
- Lijst met links voor Kristallografie
  (laatst gecontroleerd op 3 januari 2006)

---
