# Supplementary material for: Space groups and crystallographic symmetry: writing a multi-featured tutorial in a new style
Source: Acta Crystallogr E Crystallogr Commun. 2021 Jul 16;77(Pt 9):857–63. doi: 10.1107/S2056989021007039 (PMC8423017; doi:10.1107/S2056989021007039)
Supplement: Supplementary file 1 [file e-77-00857-sup2.zip › symandsg/Main/oprian.html]

Daniel Oprian, Brandeis University Biochemistry faculty member

Brandeis Search

**Home**
> Faculty

|  |  |  |
| --- | --- | --- |
|  | **Daniel Oprian, Ph.D.** Louis and Bessie Rosenfield Professor of Biochemistry   Ph.D., University of Michigan | |
|  | contact information   (781) 736-2322 | |

> Our laboratory is interested in the structure and function
> of G protein-coupled receptors with particular focus on
> the subgroup of receptors known as visual pigments. The
> pigments are major components of rod and cone photoreceptor
> cells and form the basis of phototransduction in the vertebrate
> retina. As is typical of G protein-coupled receptors, the
> visual pigments are integral membrane proteins composed
> of seven transmembrane helical segments. However, what is
> atypical is that each pigment is bound covalently to a small
> molecule ligand, 11-cis-retinal, which is a chromophore
> for the absorption of light.

> Spectral Tuning: There are four visual pigments in
> the human retina: rhodopsin, the pigment of rod photoreceptor
> cells, and the blue, green and red color vision pigments
> of cone photoreceptor cells. These four pigments have
> absorption spectra which span (actually define) the
> visible region of the electromagnetic spectrum. And
> yet, the small molecule chromophore responsible for
> absorption of light is the same in each pigment. One
> of the goals of our research is to understand the
> underlying mechanism by which the spectrum of each
> pigment is “tuned” by interactions of
> the retinal chromophore with amino acid side chains
> in the active site of the proteins.

> Mechanism of Retinal Disease: A large number of mutations
> in rhodopsin are known to cause inherited diseases
> of the retina. We are interested in a small group
> of these which result in constitutive activation of
> rhodopsin - that is, these mutations cause the protein
> to activate the G protein transducin in the absence
> of light. The activating mutations are known to cause
> two different diseases: a devastating degenerative
> disease of the retina known as retinitis pigmentosa
> and, in comparison, a relatively benign disease known
> as congenital stationary night blindness. Our goal
> is to elucidate the underlying molecular mechanisms
> responsible for pathophysiology in these diseases.
> To do this, we employ a broad array of techniques
> ranging from classical biochemistry and mutagenesis
> for in vitro characterization of the mutant proteins
> to the development of transgenic animals combined
> with single-cell electrophysiology to test models
> of disease under in vivo conditions.

|  |  |
| --- | --- |
| Structure of the Active State: Rhodopsin is the only G protein-coupled receptor for which a structure has been determined by x-ray crystallography. While the structure of rhodopsin has had an enormous impact on our understanding of the protein it is clear that we are at the very beginning of these structural studies and major questions remain. In particular, we still do not have a structure for the active conformation of the protein – the published rhodopsin structure is for the inactive- or dark-state. Our laboratory is currently undertaking a major effort to determine the x-ray crystal structure of the active state of rhodopsin. In addition, we are undertaking efforts to determine the crystal structure of other G protein-coupled receptors. |  |

**Selected Recent Publications:**

> Kono M, Crouch RK, Oprian DD. (2005) A dark and constitutively
> active mutant of the tiger salamander UV pigment. *Biochemistry*.
> **44**:799-804. [abstract]
>
> Kim JM, Altenbach C, Kono M, Oprian DD, Hubbell WL, Khorana HG.
> ( 2004) Structural origins of constitutive activation in rhodopsin:
> Role of the K296/E113 salt bridge. *Proc Natl Acad Sci U S A.*
> **101**(34):12508-13. [abstract]
>
> Das J, Crouch RK, Ma JX, Oprian DD, Kono M. (2004) Role of the
> 9-methyl group of retinal in cone visual pigments. *Biochemistry*.
> **43**(18):5532-8. [abstract]
>
> Jin S, Cornwall MC, Oprian DD. Opsin activation as a cause
> of congenital night blindness. (2003) *Nat Neurosci.* **6**:731-5. [abstract]
>
> Jin S, McKee TD, Oprian DD. (2003) An improved rhodopsin/EGFP
> fusion protein for use in the generation of transgenic Xenopus
> laevis. *FEBS Lett.* **542**(1-3):142-6. [abstract]
>
> Oprian DD. (2003) Phototaxis, chemotaxis and the missing
> link. (2003) *Trends Biochem Sci.* **28**:167-9.
> [abstract]
>
> Gross AK, Rao VR, Oprian DD. (2003) Characterization of
> rhodopsin congenital night blindness mutant T94I. *Biochemistry*. **42**:2009-15. [abstract]
>
> Gross AK, Xie G, Oprian DD. (2003) Slow binding of retinal
> to rhodopsin mutants G90D and T94D. *Biochemistry*. **42:**2002-8. [abstract]
>
> Xie G, Gross AK, Oprian DD. (2003) An opsin mutant with increased
> thermal stability. *Biochemistry*. **42**:1995-2001. [abstract]
>
> View Complete Publication List on PubMed:
>
> Daniel Oprian
>
> ---
>
> Last update: August 25, 2005. E-mail comments
> or questions to the webmaster.
>
> Top
> of Page
> | Life Science
> Faculty | Home
