# Supplementary material for: Space groups and crystallographic symmetry: writing a multi-featured tutorial in a new style
Source: Acta Crystallogr E Crystallogr Commun. 2021 Jul 16;77(Pt 9):857–63. doi: 10.1107/S2056989021007039 (PMC8423017; doi:10.1107/S2056989021007039)
Supplement: Supplementary file 1 [file e-77-00857-sup2.zip › symandsg/Main/Patterson.html]

A.L. Patterson Award

|  |  |  |  |  |  |  |  |  |  |  |
| --- | --- | --- | --- | --- | --- | --- | --- | --- | --- | --- |
|  | | | | | | | | | |  |
|  | | | | | | | | | |  |
|  | | | | | A.L. Patterson Award | | | | |  |
|  |  |  |  |  |  | | Upcoming Awards:  2005   - Presentation of Awards at Annual Meeting   Upcoming Awards:  2008   - September 1, 2006      Call for Patterson nominations - April 1, 2007 Closing date for submission of nominations - May 2008    Presentation of Awards at Annual Meeting | | |  |
|  |  |  |  |  |  |  |  |  |  |  |
|  |  | |  |  |  |  |  |  |  |  |
|  |  |  |  |  |  |  |  |  |  |  |
|  | Awards & Prizes Quick Links   - Upcoming Awards & Deadlines  - Nomination Guidelines  - Awards LIst  - Poster Prizes  - Schedule  - Previous Winners | | |  |  |  |  |  |  |  |
|  |  |  |  |  | To recognize and encourage outstanding research in the structure of matter by diffraction methods, including significant contributions to the methodology of structure determination and/or innovative application of diffraction methods and/or elucidation of biological, chemical, geological or physical phenomena using new structural information. A monetary award of $1,500 and up to $1,500 travel expenses to accept award at Annual Meeting, and a certificate are awarded every three years. Established in 1980.  **Recipient of 2002 Award** **Douglas Dorset,** *Correlations, Convolutions and the Validity of Electron Crystallography*  **Past Recipients** | | | | |  |
|  |  |  |  |  |  |  |  |  |  |  |
|  |  |  |  |  | Other Awards: | | | | |  |
|  |  |  |  |  | - Warren Award | - Buerger Award | | - Etter Award | - Fankuchen Award |  |
|  |  |  |  |  | - Patterson Award | - Wood Award | | - Trueblood Award | - Supper Award |  |
|  |  |  |  |  |  |  |  |  |  |  |
|  | |  |  |  | - Service Awards | | | | |  |
|  | | | | | | | | | |  |
