# Supplementary material for: Space groups and crystallographic symmetry: writing a multi-featured tutorial in a new style
Source: Acta Crystallogr E Crystallogr Commun. 2021 Jul 16;77(Pt 9):857–63. doi: 10.1107/S2056989021007039 (PMC8423017; doi:10.1107/S2056989021007039)
Supplement: Supplementary file 1 [file e-77-00857-sup2.zip › symandsg/Main/patterson.pdf]

Obituary

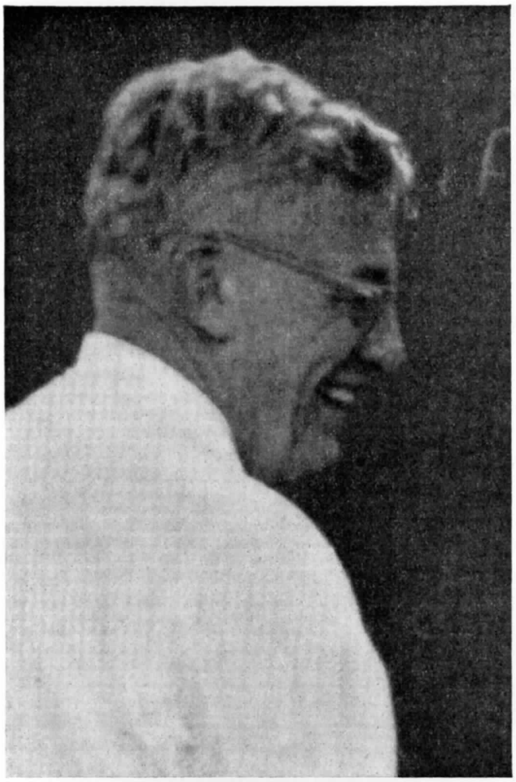

Arthur Lindo Patterson 1902–1966

On November 6, 1966, the community of crystallographers and of scholars in allied fields lost one of its most widely and dearly beloved members in the death of Arthur Lindo Patterson. Only three days earlier he had put in a normal morning's work at his laboratory in the Institute for Cancer Research, Philadelphia. On his way to lunch he began suddenly to suffer from a severe headache and soon lost consciousness, which he never recovered. Death resulted from a massive cerebral hemorrhage.

Lindo Patterson was born July 23, 1902, in Nelson (near Auckland), New Zealand. His family emigrated to Montreal, Canada, where he spent his boyhood. He attended Tonbridge School in England, and then returned to Montreal to attend McGill University. He majored in physics, receiving a bachelor's degree in 1923 and a master's in 1924. His master's thesis was on the production of hard X-rays by interaction of radium β rays with solids.

His introduction to X-ray diffraction studies of crystals came during a two-year stay in W. H. Bragg's laboratory at the Royal Institution in London. There he determined the unit cells and space groups of various phenylaliphatic acids. (The state of the art had not yet advanced to the degree that deriving the atomic arrangements in such complex molecules was a practical possibility.) He then spent a year with Hermann Mark at the Kaiser-Wilhelm Institute in Berlin, working, under a Canadian National Research Council Fellowship, on the application of X-ray diffraction methods to determine accurately the 'particle size' of cellulose. His work led to some of the first important contributions to the theory of particle-size line broadening. It was in connection with this work that he began to develop a deep interest in the nature of Fourier transformation which was later to become, in his words, an 'obsession with the notion that something had to be learned about structural analysis from Fourier theory'. While in Berlin he developed a friendly acquaintanceship with Max von Laue, with whom he had frequent discussions on many areas of mutual interest.

In 1927 he returned to McGill, finishing his work for the Ph.D. degree in 1928 and staying on for an additional year as a lecturer. (Some of the early work in low-temperature X-ray crystallography took place, in winter, outside Patterson's laboratory window in Montreal.) After two years with R. W. G. Wyckoff's

group at the Rockefeller Institute in New York, he accepted a position at the Johnson Foundation for Medical Physics in Philadelphia to apply X-ray diffraction to biological materials.

In 1933 Patterson went to the Massachusetts Institute of Technology, where he was to stay for three years as an unpaid guest in B. E. Warren's laboratory. There he cultivated the acquaintance of the celebrated mathematician Norbert Wiener, and in many conversations with him learned much about Fourier theory, including especially the properties of the convolution or *Faltung* and of its Fourier transform. Meanwhile, 'Warren with Gingrich and others had perfected the techniques used by Debye and Menke in the study of the X-ray scattering from liquids. These were of course based on the original suggestions of Zernike and Prins. Warren and Gingrich had already had the idea that these methods applied to powders would give the radial distribution in a crystal. While trying to learn about their work I noticed that the mathematical form of the theory given by Debye and Menke would be identical with that of the *Faltung* if the integrations over random orientation were left out and the randomness of choice of origin was left in. What was immediately apparent was that the crystal contained atoms and that the *Faltung* of a set of atoms was very special in that it would consist of a set of atom-like peaks whose centers were specified by the distances between the atoms in the crystal'. Thus was born, in 1934, the *Patterson function*. Viewed from this point in time, the Patterson function (which its author always referred to as the 'F^2 series') represents perhaps the most important single development in crystal-structure analysis since the discovery of X-ray diffraction itself. However, the full implementation of the principle had to wait many years for development of practical means of summing the Fourier series in two and three dimensions; to this, Patterson, together with George Tunell, made an important early contribution in the development of the Patterson–Tunell summation strips.

In 1935 Lindo Patterson married Elizabeth Knight of New York City. The following year he joined the faculty at Bryn Mawr College, a women's college near Philadelphia, where he combined work in X-ray diffraction analysis with teaching and the training of research students. While at Bryn Mawr he published two important papers on particle-size line broadening, representing work begun at M.I.T. In addition he became concerned about the problem of uniqueness of the deconvolution of the Patterson function and was able to show that under some conditions several different atomic arrangements – 'homometric structures' – could exist that would give the same Patterson function and therefore the same intensities in reciprocal space.

In 1949 he left Bryn Mawr to start an X-ray structure analysis group at the Institute for Cancer Research in Philadelphia. Here he was able to realize a long-held ambition to apply X-ray analysis to substances of biological interest; some of the more important work is represented by a series of papers on the crystal structures of citric acid and of various citrate salts. He drew to his laboratory a lively group of young scientists, several of whom in due course have left to found their own laboratories elsewhere. They have taken with them, besides their research experience, the inspiration, both scientific and personal, that rubbed off so easily from Lindo Patterson.

Patterson served the crystallographic community as a member of the Executive Committee of the International Union of Crystallography from 1948 to 1954, and on a subcommittee concerned with revising its Statutes and Bylaws. He also served for many years on the U.S.A. National Committee for Crystallography, of which he was Chairman from 1948 to 1950. Any list of his formal contributions to crystallography should also include an extremely valuable chapter in Volume II (1959) of *International Tables for X-ray Crystallography*, summarizing the basic mathematics underlying crystallographic theory.

Lindo Patterson's high standing among crystallographers, particularly in America, goes far beyond his published scientific work. It derives to a very great extent from his character and personality, for he possessed the rare combination of a keen mind, a lively humor, and a gentle disposition. Large segments of two generations of crystallographers – numerically out of all proportion to the number he trained in any formal sense – were inspired by Lindo Patterson and proud of even a casual acquaintanceship with him. Many of us remember, in particular, the lift we got from Patterson's friendly word of appreciation and encouragement after one of our first attempts at giving a paper at a meeting of the American Crystallographic Association. We will no longer see him at these meetings, but we are truly grateful for the privilege of having known Lindo Patterson.

The Institute for Cancer Research has established the A. L. Patterson Memorial Fund in his honor. It is hoped that this fund will grow so that a Fellowship can be awarded in his name.

R. E. MARSH
D. P. SHOEMAKER
