# Supplementary material for: Space groups and crystallographic symmetry: writing a multi-featured tutorial in a new style
Source: Acta Crystallogr E Crystallogr Commun. 2021 Jul 16;77(Pt 9):857–63. doi: 10.1107/S2056989021007039 (PMC8423017; doi:10.1107/S2056989021007039)
Supplement: Supplementary file 1 [file e-77-00857-sup2.zip › symandsg/Main/rontgen-bio.html]

Wilhelm Conrad Röntgen - Biography

- Nobel Foundation
- Nobel Media
- Nobel Museum
- Nobel Peace Center
- Nobel Web
- SEARCH
- CONTACT US
- HOME


- NOBEL PRIZES
- ALFRED NOBEL
- PRIZE AWARDERS
- NOMINATION
- PRIZE ANNOUNCEMENTS
- AWARD CEREMONIES
- EDUCATIONAL GAMES

- By Year
- Nobel Prize in Physics
- Nobel Prize in Chemistry
- Nobel Prize in Medicine
- Nobel Prize in Literature
- Nobel Peace Prize
- Prize in Economics


## Wilhelm Conrad Röntgen

The Nobel Prize in Physics 1901

## Biography

**Wilhelm
Conrad Röntgen** was born on March 27, 1845, at Lennep in
the Lower Rhine Province of Germany, as the only child of a
merchant in, and manufacturer of, cloth. His mother was Charlotte
Constanze Frowein of Amsterdam, a member of an old Lennep family
which had settled in Amsterdam.  
  
When he was three years old, his family moved to Apeldoorn in The
Netherlands, where he went to the Institute of Martinus Herman
van Doorn, a boarding school. He did not show any special
aptitude, but showed a love of nature and was fond of roaming in
the open country and forests. He was especially apt at making
mechanical contrivances, a characteristic which remained with him
also in later life. In 1862 he entered a technical school at
Utrecht, where he was however unfairly expelled, accused of
having produced a caricature of one of the teachers, which was in
fact done by someone else.  
  
He then entered the University of Utrecht in 1865 to study physics. Not
having attained the credentials required for a regular student,
and hearing that he could enter the Polytechnic at Zurich by
passing its examination, he passed this and began studies there
as a student of mechanical engineering. He attended the lectures
given by Clausius and also worked in the laboratory of Kundt.
Both Kundt and Clausius exerted great influence on his
development. In 1869 he graduated Ph.D. at the University of Zurich,
was appointed assistant to Kundt and went with him to
Würzburg in the same year, and three years later to
Strasbourg.  
  
In 1874 he qualified as Lecturer at Strasbourg University and in
1875 he was appointed Professor in the Academy of Agriculture at
Hohenheim in Wurtemberg. In 1876 he returned to Strasbourg as
Professor of Physics, but three years later he accepted the
invitation to the Chair of Physics in the University of
Giessen.  
  
After having declined invitations to similar positions in the
Universities of
Jena (1886) and Utrecht (1888), he accepted it from the
University
of Würzburg (1888), where he succeeded Kohlrausch und
found among his colleagues Helmholtz and Lorenz. In 1899 he
declined an offer to the Chair of Physics in the University of
Leipzig, but in 1900 he accepted it in the University of
Munich, by special request of the Bavarian government, as
successor of E. Lommel. Here he remained for the rest of his
life, although he was offered, but declined, the Presidency of
the Physikalisch-Technische Reichsanstalt at Berlin and the Chair
of Physics of the Berlin Academy.  
  
Röntgen's first work was published in 1870, dealing with the
specific heats of gases, followed a few years later by a paper on
the thermal conductivity of crystals. Among other problems he
studied were the electrical and other characteristics of quartz;
the influence of pressure on the refractive indices of various
fluids; the modification of the planes of polarised light by
electromagnetic influences; the variations in the functions of
the temperature and the compressibility of water and other
fluids; the phenomena accompanying the spreading of oil drops on
water.  
  
Röntgen's name, however, is chiefly associated with his
discovery of the rays that he called X-rays. In 1895 he was
studying the phenomena accompanying the passage of an electric
current through a gas of extremely low pressure. Previous work in
this field had already been carried out by J. Plucker
(1801-1868), J. W. Hittorf (1824-1914), C. F. Varley (1828-1883),
E. Goldstein (1850-1931), Sir William Crookes (1832-1919), H.
Hertz (1857-1894) and Ph. von
Lenard (1862-1947), and by the work of these scientists the
properties of cathode rays - the name given by Goldstein to the
electric current established in highly rarefied gases by the very
high tension electricity generated by Ruhmkorff's induction
coil-had become well known. Röntgen's work on cathode rays
led him, however, to the discovery of a new and different kind of
rays.  
  
On the evening of November 8, 1895, he found that, if the
discharge tube is enclosed in a sealed, thick black carton to
exclude all light, and if he worked in a dark room, a paper plate
covered on one side with barium platinocyanide placed in the path
of the rays became fluorescent even when it was as far as two
metres from the discharge tube. During subsequent experiments he
found that objects of different thicknesses interposed in the
path of the rays showed variable transparency to them when
recorded on a photographic plate. When he immobilised for some
moments the hand of his wife in the path of the rays over a
photographic plate, he observed after development of the plate an
image of his wife's hand which showed the shadows thrown by the
bones of her hand and that of a ring she was wearing, surrounded
by the penumbra of the flesh, which was more permeable to the
rays and therefore threw a fainter shadow. This was the first
"röntgenogram" ever taken. In further experiments,
Röntgen showed that the new rays are produced by the impact
of cathode rays on a material object. Because their nature was
then unknown, he gave them the name X-rays. Later, Max von Laue and his pupils showed that
they are of the same electromagnetic nature as light, but differ
from it only in the higher frequency of their vibration.  
  
Numerous honours were showered upon him. In several cities,
streets were named after him, and a complete list of Prizes,
Medals, honorary doctorates, honorary and corresponding
memberships of learned societies in Germany as well as abroad,
and other honours would fill a whole page of this book. In spite
of all this, Röntgen retained the characteristic of a
strikingly modest and reticent man. Throughout his life he
retained his love of nature and outdoor occupations. Many
vacations were spent at his summer home at Weilheim, at the foot
of the Bavarian Alps, where he entertained his friends and went
on many expeditions into the mountains. He was a great
mountaineer and more than once got into dangerous situations.
Amiable and courteous by nature, he was always understanding the
views and difficulties of others. He was always shy of having an
assistant, and preferred to work alone. Much of the apparatus he
used was built by himself with great ingenuity and experimental
skill.  
  
Röntgen married Anna Bertha Ludwig of Zürich, whom he
had met in the café run by her father. She was a niece of
the poet Otto Ludwig. They married in 1872 in Apeldoorn, The
Netherlands. They had no children, but in 1887 adopted Josephine
Bertha Ludwig, then aged 6, daughter of Mrs. Röntgen's only
brother. Four years after his wife, Röntgen died at Munich
on February 10, 1923, from carcinoma of the intestine.

From *Nobel Lectures, Physics 1901-1921*, Elsevier Publishing Company, Amsterdam, 1967


This autobiography/biography was first
published in the book series *Les
Prix Nobel*.
It was later edited and republished in *Nobel
Lectures*.
To cite this document, always state the source as shown above.

Copyright © The Nobel Foundation 1901

- Printer Friendly
- Comments & Questions
- Tell a Friend

#### The 1901 Prize in:

PhysicsChemistryMedicineLiteraturePeace

- Next year

#### The Nobel Prize in Physics 1901

- Presentation Speech

##### Wilhelm Conrad Röntgen

- Biography
- Nobel Lecture
- Swedish Nobel Stamps
- Educational
- Other Resources

Only 33 out of 758 are women!

Who can nominate for a Nobel Prize?

"War leaves no victors, only victims."


The Official Web Site of the Nobel Foundation

Copyright © Nobel Web AB 2006
