# Supplementary material for: Space groups and crystallographic symmetry: writing a multi-featured tutorial in a new style
Source: Acta Crystallogr E Crystallogr Commun. 2021 Jul 16;77(Pt 9):857–63. doi: 10.1107/S2056989021007039 (PMC8423017; doi:10.1107/S2056989021007039)
Supplement: Supplementary file 1 [file e-77-00857-sup2.zip › symandsg/Main/spcgrp_tut.htm]

Introduction to Space Groups

# Introduction to Space Groups

---

### Introduction

In the previous chapter we have seen that a combination of
symmetry operations on the structure and subsequent stacking into
a lattice will build up a crystal. Let us investigate this a
little further.

### Point group operations

The *point group symmetry operations* applied to a
motif can be deduced from the external symmetry of a (perfect)
crystal. Normals to the crystal faces intersect in a single *point*
in the centre of the crystal. Examples for point group symmetry
operations are Rotation Axes, Mirror Planes, and Inversion
Axes.Two important restrictions apply to the point group symmetry
operations :

1. The symmetry operations must be compatible with infinite
   translational repeats in a lattice
2. A symmetry operation cannot induce a higher symmetry than
   the motif already has.

**Rotation Axes** : Condition (1) limits *n*-fold
rotation axes to *n* = 1, 2, 3, 4,
6. You cannot create a space filling pattern with 5- fold or
8-fold rotations, but a single molecule may well have 5-fold
symmetry (check).  **Exercise** : If the above is true, how comes that one of
the platonic bodies, the pentagon dodecahedron, exhibits pentagonal
faces and/but has cubic symmetry?

**Inversion Axes** : Same as above, except that
after the 360/*n* rotation the object is projected through
a inversion center on the axis. The axes are denoted with a bar
above, I use minus signs here : -1, -2, etc....   
Here is a fun **exercise** that will help you
understand the second limitation : draw a simple chiral molecule, and then apply a -2 operation to it. What happened?

**Mirror Plane** : pretty obvious operation,
denoted *m*. After the exercise above, you should already
have found that neither inversion axes nor a mirror plane are
acceptable symmetry operations in case of a protein molecule.

A combination of the point group symmetry operations leads to *32 point groups*. As they can be
deduced from the macroscopic crystal symmetry, they are also
referred to as *the 32 Crystal Classes*. For the same
reason, the symmetry elements that give rise to the Crystal
Classes are sometimes referred to as *external* symmetry
elements.

### Crystal Systems and Bravais Lattices

Let us now consider in which way we can translate our cell
contents in 3 dimensions to obtain a crystal. The translations
which are allowed create **14
Bravais lattices** which belong to **7 crystal
systems****.** Each system has a
primitive cell, and some allow face or body centering, as well as
the rhombohedral centering in the trigonal system (the
rhombohedral lattice can be derived from a cube by pulling along
it's space diagonal). The translational elements also allow for 2
new types of symmetry operation :

**Screw Axes :** There are n-1 n-fold screw axes
N_z_ , z =1..n-1, n=2,3,4,6. They are combined symmetry
elements, resulting from a rotation around 360/n degrees,
followed by a translation by z translations of 1/n \* x, where x
is a lattice vector parallel to the screw axis. For a 2-fold screw axis (2_1_), there is
only one translation by 1/2\*x, for 4 folds there are 3
possibilities 4_1_, 4_2_, 4_3_ , with
translation of 1/4, 2/4, and 3/4 along the parallel lattice
vector. Screw axes are very common in protein structures.

**Glide Planes :** For the same reasons mentioned
above, glide planes, a combination of a mirror plane and a
translation operation parallel to it, cannot appear in protein
structures and are sometimes ignored in macromolecular
crystallography textbooks.They are very common in inorganic
structures (diamond for example, has, you guessed it, a diamond
glide *d*). Generally, a glide is constructed by a mirror
operation followed by a translation along a/2, b/2, c/2 (*a,b,c*),
a face diagonal (a+b)/2, (a+c)/2, (b+c)/2 (in all cases denoted *n*)
or, in case of *d* along (a+b)/4, (a+c)/4 or (b+c)/4.

**Note** : Glides and screws are not point group
operations, because they involve translations. They only work
with (and are compatible with) additional translations in a
lattice. As an example, a 4_3_ axis yields an identical
orientation of the molecule only after 4 repeated applications -
3 unit cells away (4\*3/4 = 3). Clearly, this is not a point group
operation. The same holds for glides, and we'll see that *all
operations involving translations* (Bravais centerings,
glides and screws) yield *observable extinctions* (systematically absent reflections) in the
diffraction pattern. We will discuss this shortly. One also
cannot distinguish a rotation axis from a corresponding screw
axis or a glide plane from a mirror plane by just looking at the
crystal faces. Thus these symmetry elements involving
translations are also referred to as *internal symmetry
elements*, in contrast to the *externally observable point
group* operations (point groups or
Crystal Classes). The only difference between 2
and 2_1_ is that every other
molecule would stick out of a lattice plane (crystal face)
perpendicular to the axis by 1/2 of the unit cell in case of the
screw axis. Currently none of the AF microscopes are good enough
to see this.

### Finally, Space Groups

The combination of all available symmetry operations (point
groups plus glides and screws) with the Bravais translations
leads to exactly 230 combinations, the 230 *Space Groups*.
The International Tables list those by symbol and number,
together with symmetry operators, origins, reflection conditions,
and space group projection diagrams. In the next
chapter we will go into more detail of space groups and use
an interactive program to decode some of the space group symbols.


---

**Back to X-ray Tutorial Index**  
**This World Wide Web site conceived and maintained
by Bernhard Rupp**  **Last revised
April 06, 1999 11:05**

---
