# Supplementary material for: Space groups and crystallographic symmetry: writing a multi-featured tutorial in a new style
Source: Acta Crystallogr E Crystallogr Commun. 2021 Jul 16;77(Pt 9):857–63. doi: 10.1107/S2056989021007039 (PMC8423017; doi:10.1107/S2056989021007039)
Supplement: Supplementary file 1 [file e-77-00857-sup2.zip › symandsg/Main/spcgrps.htm]

# Space Groups

---

### Introduction

In the previous chapter we have shown that the combination
of all available symmetry operations (point group operations
plus glides and screws) with the Bravais translations leads to
exactly 230 combinations, the 230 *Space Groups*. The International Tables (IT)
list those by symbol and number, together with symmetry operators, origins, reflection
conditions, and space group projection diagrams. We will go into more detail of space
groups now and we will use an interactive web program to learn more about the space
groups.

### Space group symbols

The commonly used space group symbols are the short Hermann-Mauguin symbols, consisting
of a Bravais symbol followed by the symmetry operators generating the equivalent positions
(equipoints). I have listed all 230 space groups and
the symbols we use to enter them into the decoding program. Note that the combination of
the given symmetry operations yields additional symmetry elements not listed separately
(see the space group symmetry diagrams in the IT). For example, Fm3m is a cubic space
group, and the 2-folds and 4-folds present are not listed in the symbol. Only the so
called full symbol (F 4/m -3 2/m) shows these additional elements.

At this point it is worthwhile to recall that protein structures are restricted to
non-mirror and non-inversion symmetry operations. This effectively reduces the number of
possible groups to the 65 chiral space groups. The by far most
common space groups for proteins are P 21 21 21 and P 21. An entropic model indicates that
these space groups are the least restrictive to packing (rigid body degrees of freedom)
and thus they can be created in a larger number of ways (Wukovitz and Yates, Nature
Structural Biology **2** (12), 1062-1067, 1995).

### How space groups work on a motif

In order to account for the whole unit cell content, we need to apply the symmetry and
Bravais operations to the asymmetric unit, in our case the protein (or something such as a
dimer, perhaps with a non-crystallographic symmetry relating the 2 monomers). The easiest
way to see this at work is to use the following program to decode the space group and to
read through the output. An explanation for the
output is available.

**Enter a space group or space group number  and hit enter**

### Exercises :

- Try a centrosymmetric space group. See what happens to the number of equivalent
  positions created.
- Decode a space group with Bravais centering. What does it add to the equipoints created?
- What is the maximum number of equivalent positions in a space group? (hint : try a
  higher symmetric space group such as F d 3 m).
- Based on that number (and neglecting that such a space group is not chiral) would you
  expect a protein with such a unit cell?
- **FINAL QUIZ** : go back to the introduction and look at the RGFP crystal. What is the spacegroup and its
  symmetry operator(s)? Click here to submit your answer.
  If your answer is right, you will be listed in the Space Group
  Hall of Fame. If not, I'll reply and explain. In case you worry, I will not list wrong answers.

Real life example for
protein unit cell (gif images of BPTI, PDB
entry 1BPI

By now you should have a feel for space group symmetry and how to interpret a symbol.
It is now time to revisit the diffraction experiment in
detail and to dwell on the relations between the space group
symmetry and the diffraction data. You may save a lot of time on data collection and
will have less problems later in the structure determination by understanding these
concepts.


---

**Back to X-ray Tutorial Index**  
**This World Wide Web site conceived and maintained
by Bernhard Rupp**  **Last revised
April 04, 2000 10:17**

---
