# Supplementary material for: Space groups and crystallographic symmetry: writing a multi-featured tutorial in a new style
Source: Acta Crystallogr E Crystallogr Commun. 2021 Jul 16;77(Pt 9):857–63. doi: 10.1107/S2056989021007039 (PMC8423017; doi:10.1107/S2056989021007039)
Supplement: Supplementary file 1 [file e-77-00857-sup2.zip › symandsg/Main/swift.html]

Georgetown University - Department of Chemistry

Faculty

Faculty

Staff

Staff

Graduate Students & Postdocs

Graduate Students & Postdocs

Overview

Overview

Courses

Courses

Chemistry Club

Chemistry Club

Research

Research

Awards

Awards

Summer REU Program

Summer REU Program

Procedures & Application

Procedures & Application

Visiting the Department

Visiting the Department

Admissions

Admissions

Financial Information

Financial Information

Degree Program & Areas of Study

Degree Program & Areas of Study

Frequently Asked Questions

Frequently Asked Questions

Admissions Contact Information

Admissions Contact Information

Resources

Resources

Phase I Examination Information

Phase I Examination Information

Housing Information

Housing Information

Prospective

Prospective

Current

Current

Research Facilities

Research Facilities

Stockroom

Stockroom

Library

Library

Safety

Safety

Tutoring

Tutoring

Career Planning

Career Planning

Department News

Department News

Seminar Series

Seminar Series

*Hoya Chimica* Newsletter

*Hoya Chimica* Newsletter

Maps & Directions

Maps & Directions

Georgetown University

Georgetown University

Washington, D.C.

Washington, D.C.

Giving

Giving

Alumni Whereabouts

Alumni Whereabouts

Alumni Records

Alumni Records


|  |  |
| --- | --- |
|  |  |

**Georgetown University  
Department of Chemistry**  
Box 571227  
Washington DC 20057  
Phone: (202)687-6073  
Fax: (202)687-6209

|  |  |
| --- | --- |
| Jennifer A. Swift  Associate Professor  Department of Chemistry    Georgetown University   37th and O Streets NW   Washington, DC 20057-1227 **Office:**  647 Reiss Science  **Phone:** 202-687-5567  **Fax:** 202-687-6209   **E-mail:** | |
| Lab web site | http://georgetown.edu/faculty/jas2 |
| Education /  Background | B.A. *magna cum laude*, 1991 Bowdoin College  Ph. D. 1997 Yale University  Postdoctoral associate, 1997-1999 Dept of Chemical Engineering & Materials Science, University of Minnesota,  *NSF CAREER Award (2001); CNDLS Teaching, Learning & Technology Fellow (2003-2004); Camille Dreyfus Teacher-Scholar Award (2004); American Crystallography Association Margaret C. Etter Award (2005);  *College Dean’s Award for Excellence in Teaching (2005)**  [cv] |
| Teaching | Organic Chemistry Lecture I, Organic Chemistry Lab I & II, Solid State Organic Chemistry |
| Research Interests | Research in the Swift lab is directed toward gaining a more detailed understanding of the mechanisms by which molecular crystals nucleate and grow. Most of the projects in this research area are quite interdisciplinary - meaning students will be expected to gain experience in at least a few different areas of chemistry including x-ray crystallography, atomic force microscopy (AFM), organic synthesis, and computational modeling. 1.  Crystal Growth in Human Disease/Biomineralization  **Cholesterol** is a major constituent of human gallstones and arterial plaques.  Using *in situ* AFM, the real-time crystal growth of this important material can be elucidated.  Unprecedented details on the crystal's surface structure, growth rates and growth mechanisms are obtained in model bile solutions and in a variety of conventional organic solvents.  The nucleating (or anti-nucleating) influence of assorted impurities is also a subject of interest.  **Uric Acid** is a known component of human kidney stones and gouty deposits.  Fundamental AFM studies provide insight into how solution parameters (e.g. pH, supersaturation, impurities) affect crystal growth kinetics and/or mechanisms.  Complementary experiments using microspectrometry are used to elucidate recognition events between the crystal surface and designer impurities.  2.  Crystal Growth With 2D- and 3D- Templates  **Crystal Growth in Gels** has been used for over a century as a method for growing metals and inorganic crystals, but the details of nucleation and growth within these media are not well understood on the molecular-level.  Our fundamental studies seek to understand how intermolecular interactions between the gel and crystal can be exploited to access unusual crystal morphologies, affect chiral separations and/or template the growth of new polymorphic phases.  **Self Assembled Monolayers** can be very effective templates for the growth of a variety of moleuclar crystals.  The rational design and testing of a series of SAMs with controlled functionality and lattice spacing, provides a means to construct a combinatorial library of 2D templates for molecular crystal growth studies. |
| In the News | Crystal Clear Persuasion   The Department of Chemistry's Jennifer Swift is launching a war on crystals that cause gallstones or kidney stones, hoping to slow down their growth and understand what makes them tick. June 1, 2005 Chemistry Professor Honored with Achievement Award  Professor Jennifer Swift has received the 2005 Margaret C. Etter Early Career Award. The annual award recognizes scientists who have made outstanding achievements and shown great potential at early stages of their careers. January 12, 2005  Georgetown Recognizes Outstanding Faculty  Georgetown University awarded professors Norman J. Finkel, Nicoletta Pireddu and Jennifer Swift the College Dean Awards for Excellence in Teaching at the 2005-2006 College Faculty Convocation on November 14, 2005. |
| Recent Publications | Rupa Hiremath, Joseph A. Basile, Stephen W. Varney, and Jennifer A. Swift*,* “Controlling Polymorphism of Molecular Crystals with Self-Assembled Monolayer Templates,” *J. Am. Chem. Soc*., 127, 18321-18327 (2005).  Richard S. Abendan and Jennifer A. Swift, “ Dissolution on Cholesterol Monohydrate Single Crystal Surfaces Monitored by In Situ Atomic Force Microscopy ,” *Crystal Growth & Design,* 5(6), 2146-2153 (2005).  Ryan E. Sours, Amanuel Z. Zellelow and Jennifer A. Swift, “An *in situ* Atomic Force Microscopy Study of Uric Acid Crystal Growth,” *J. Phys. Chem B,* 109, 9989-9995 (2005)*.*  Rupa Hiremath, Stephen W. Varney and Jennifer A. Swift, “Oriented Crystal Growth of 4-Iodo-4’-Nitrobiphenyl on Polar Self-Assembled Monolayer Templates: a Case for “Chemical Epitaxy”,” *Chem. Mater.,*  16, 24, 4948- 4954 (2004). M. Crina Frincu, Sean D. Fleming, Andrew L. Rohl and Jennifer A. Swift, “The Epitaxial Growth of Cholesterol Crystals from Model Bile Solutions on Calcite Substrates,” *J. Am. Chem. Soc.,* 126, 25, 7915 – 7924 (2004). [complete list] |

   page last updated:
April 19, 2006

people | undergraduate | graduate | resources | news & events | alumni | home

� Copyright 2006 Georgetown University - Department
of Chemistry. All Rights Reserved

Site design: Academic Web Pages
