# Supplementary material for: Space groups and crystallographic symmetry: writing a multi-featured tutorial in a new style
Source: Acta Crystallogr E Crystallogr Commun. 2021 Jul 16;77(Pt 9):857–63. doi: 10.1107/S2056989021007039 (PMC8423017; doi:10.1107/S2056989021007039)
Supplement: Supplementary file 1 [file e-77-00857-sup2.zip › symandsg/Main/swift_files/arrows.htm]

Georgetown University: File not found


File not found

The web page you selected could not be found, or the server has been instructed not to let you have the page.

To search for the missing page or find a related page, please use one of these options:

A. Search the Georgetown web

Help
| Advanced
| A-Z index

B. Browse the alphabetical site index

Recommended for common topics.

C. Search for a person

Search the online directory of Georgetown University students, faculty, and staff.

D. Search for an event

Search the university event calendar.
