# Supplementary material for: Space groups and crystallographic symmetry: writing a multi-featured tutorial in a new style
Source: Acta Crystallogr E Crystallogr Commun. 2021 Jul 16;77(Pt 9):857–63. doi: 10.1107/S2056989021007039 (PMC8423017; doi:10.1107/S2056989021007039)
Supplement: Supplementary file 1 [file e-77-00857-sup2.zip › symandsg/Main/tric-gif.html]

Draft


# Triclinic lattice

#### a_1_ ≠ a_2_ ≠ a_3_

#### α ≠ β ≠ γ


## Simple triclinic

 


#### Click on the picture to repeat animation

 Go back
