# Supplementary material for: Space groups and crystallographic symmetry: writing a multi-featured tutorial in a new style
Source: Acta Crystallogr E Crystallogr Commun. 2021 Jul 16;77(Pt 9):857–63. doi: 10.1107/S2056989021007039 (PMC8423017; doi:10.1107/S2056989021007039)
Supplement: Supplementary file 1 [file e-77-00857-sup2.zip › symandsg/Main/trueblood.htm]

(IUCr) Crystallographers Kenneth Nyitray Trueblood, (1920-1998) - Obituary


---

# Kenneth Nyitray Trueblood, (1920-1998) - Obituary

*Acta Cryst.* (1998). B**54**, 505-506.


Kenneth N. Trueblood, a pioneer in the development of the use of
computers for the determination and refinement of crystal structures
and in the analysis of molecular motion in the solid state, died of a
melanoma at home in Los Angeles on 7 May 1998. He was a much respected
scientist and teacher. He leaves a wife, Jean, a graduate of the School
of Social Work at Columbia University.

Born in Dobbs Ferry, New York, USA, on 24 April 1920, the youngest
of three boys, Ken obtained an AB from Harvard in 1941 and a PhD in
chemistry from Caltech in 1947. After 2 years as a postdoc at Caltech,
where he was inspired by the work of Linus Pauling, Ken went to UCLA as
an instructor in the Chemistry Department. Within a year he had been
made an Assistant Professor, becoming a full Professor in 1960. He
received a Fulbright Award to study at Oxford University between 1956
and 1957, and he used this occasion to work on vitamin B12
with Dorothy Hodgkin. He was an exchange visitor at the Institute of
Elemento-Organic Compounds in Moscow in 1965, where he got to know
Kitaigorodsky, and a Visiting Professor at the University of Ibadan,
Nigeria, 1964-1965. He also worked with Jack Dunitz when he was on
sabbatical at the Swiss Federal Institute of Technology in Zurich,
1976-1977. Ken retired from UCLA in 1989 as Professor Emeritus, but
continued with research projects. A small meeting held at that time in
his honor demonstrated the affection that his former students and his
colleagues had for him. Ken served the crystallographic community as
President of the American Crystallographic Association in 1961, member
of the US National Committee for Crystallography and its Vice Chair in
1963-1965. He also served with distinction in several administrative
positions at UCLA - Chair of the Chemistry Department 1965-1970 and
1990-1991 (called back after his retirement), Dean of the UCLA College
of Letters and Science from 1971 to 1974, and Chair of the UCLA
Academic Senate 1983-1984. In addition to science he loved sports,
especially the New York Yankees, and attended as many UCLA games as
possible with his wife, Jeanie.

Among the earliest papers from the US on crystallographic computer programs was a gem from Ken's laboratory [Acta Cryst. (1953), **6**,
427] on three-dimensional summations on a digital computer. This came
at a time when few crystallographers were working with
three-dimensional data because the calculations involved were
unbelievably tedious and time-consuming. The US National Bureau of
Standards Western Automatic Computer had been installed at UCLA in the
early 1950's and Ken set about using it for crystallographic work. The
development of suitable programs for calculating electron-density maps
and, later, for refining structures by Ken and his students Stan Mayer,
Dick Prosen, F. Kruse and Bob Sparks led to his collaboration in 1954
with Dorothy Hodgkin on the crystal structure of vitamin B12.
This was the largest unknown chemical structure determined at that time
and its solution contributed to the Nobel Prize won by Dorothy. In a
collaboration carried out by normal mail between Oxford, England, and
California, USA, Ken calculated electron-density maps phased on
successively larger and larger portions of the molecule. Ken's part in
this story was essential to the determination of the chemical formula
of the vitamin [Nature, (1955), **176**, 325]. He then
continued with the refinement of the structure. Crystallographic
computer programs were, at that time, among the most complex written
and, because of the amount of memory they used and the amount of time
they took, they usually were run at night. The programmer also had to
be the computer operator and needed to know how to test and replace
tubes, diodes and other portions of the massive machine in addition to
rectifying problems in the program. Later Ken and Robert Long developed
some of the very first programs for use in direct methods of structure
determination.

Ken continued with crystallographic studies of strained polycyclic
molecules and host-guest complexes, and also, in collaboration with
Verner Schomaker and Jack Dunitz, worked on thermal motion and disorder
in the crystalline state and its adequate description (rigid-body
analysis, force constants and rotation barriers) [J. Phys. Chem. (1988), **92**,
856]. When he was told that his time was limited because of his
illness, his major task (which he completed) was to finish a paper with
Verner Schomaker (who died in 1997). Uniquely, this final paper
immediately follows this obituary. Crystal structures determined by him
include those of hydroxy­proline and some derivatives of nucleic acid
bases, which led to some observations on hydrogen bonding between base
pairs with Jerry Donohue. He also studied the structures of some
strained organic molecules including hexahelicene, for which he
established the absolute configuration, and some cyclophanes with Emily
Maverick, Joel Bernstein, Hakon Hope and Carolyn Knobler. Later he
studied host-guest complexes with Don Cram, Emily Maverick and Carolyn
Knobler.

Ken excelled as a teacher. He was a member of the IUCr Commission on
Crystallographic Teaching and obtained the first UCLA Distinguished
Teaching Award in 1961, an Award for Excellence in Teaching from the
Manufacturing Chemists Association in 1978, a Letters and Science
Faculty Award from UCLA in 1982 and the Fankuchen Memorial Award of the
American Crystallographic Association in 1995. He taught in many ACA-
and IUCr-sponsored schools and always provided the students with clear
and relevant presentations. His significant contributions to
crystallographic teaching were remembered with respect at the opening
of the 1998 Erice Crystallography School. In addition, he coauthored Crystal Structure Analysis: A Primer and Chem One,
and both texts have influenced several generations of students. At a
memorial session for him at the ACA meeting in Washington, July 1998,
some of his former students described how he knew the names of each
individual member of his classes (no matter how large), demanded high
standards from them in experimental work and writing, and was always
available for them to talk to. Ken could be gentle but firm, but it was
always clear that he wanted to encourage students to do their best and
develop their interests and understanding of chemistry. A modest man,
he had a fine sense of humor and an infectious chuckle. This
enthusiastic pioneer of modern crystallography will be sadly missed.

*Jenny P. Glusker
  
The Institute for Cancer Research,
  
The Fox Chase Cancer Center,
  
7701 Burholme Avenue,
  
Philadelphia, PA 19111, USA*

Index to Crystallographers

---

**Copyright © 1998 International Union of Crystallography**


IUCr Webmaster
