# Supplementary material for: Space groups and crystallographic symmetry: writing a multi-featured tutorial in a new style
Source: Acta Crystallogr E Crystallogr Commun. 2021 Jul 16;77(Pt 9):857–63. doi: 10.1107/S2056989021007039 (PMC8423017; doi:10.1107/S2056989021007039)
Supplement: Supplementary file 1 [file e-77-00857-sup2.zip › symandsg/Main/volume_A.htm]

International Tables for Crystallography, Volume A: Space Group... - Crystal...Journals, Books & Online Media | Springer

|  |  |
| --- | --- |
| Please select Africa Asia Australia / Oceania Europe France Germany Italy North America South America Switzerland United Kingdom |  |

|  |  |  |  |
| --- | --- | --- | --- |
|  |  | | |
|  | All Author/Editor Title ISBN/ISSN Series |  |  |
|  |  |  |  |

|  |  |  |  |
| --- | --- | --- | --- |
| Journals | Textbooks | Series | Landolt-Börnstein |

Select your subdisciplineCharacterization & Evaluation of MaterialsNanotechnologyOptical & Electronic MaterialsPolymer ScienceSpecial types of MaterialsStructural Materials & BiomaterialsSurfaces, Interfaces, Thin Films, Corrosion, Coatings


Select your
subdiscipline
Characterization & Evaluation of
Materials
Nanotechnology
Optical & Electronic
Materials
Polymer Science
Special types of Materials
Structural Materials &
Biomaterials
Surfaces, Interfaces, Thin Films,
Corrosion, Coatings

Select a disciplineArchitecture & DesignAstronomyBiomedical SciencesBusiness & ManagementChemistryComputer ScienceEconomicsEducationEngineeringEnvironmental SciencesGeographyGeosciencesHumanitiesLawLife SciencesLinguisticsMaterialsMathematicsMedicinePhilosophyPhysicsPsychologyPublic HealthSocial SciencesStatistics


Select a
discipline
Architecture & Design
Astronomy
Biomedical Sciences
Business & Management
Chemistry
Computer Science
Economics
Education
Engineering
Environmental Sciences
Geography
Geosciences
Humanities
Law
Life Sciences
Linguistics
Materials
Mathematics
Medicine
Philosophy
Physics
Psychology
Public Health
Social Sciences
Statistics

> 


Home
/
Materials

|  |  |  |  |  |
| --- | --- | --- | --- | --- |
|  | International Tables for Crystallography, Volume A: Space Group Symmetry  Series: International Tables for Crystallography , Vol. A   Hahn, Theo (Ed.)   5th revised ed. 2002. Corr. 2nd printing, 2005, 938 p., Hardcover  ISBN: 0-7923-6590-9     This item usually ships in 2-3 days.  $255.00      About this book  \|  Table of contents      About this book  Volume A treats crystallographic symmetry in direct or physical space.  The first five parts of the volume contain introductory material: lists of symbols and terms; a guide to the use of the space-group tables; the determination of space groups; synoptic tables of space-group symbols; and unit-cell (coordinate) transformations. These are followed by the plane-group and space-group tables.  The rest of the volume is at a much higher theoretical level than Parts 1 to 5; it has many features of an advanced textbook of crystallography. Parts 8 to 15 deal with the following aspects of symmetry theory: the mathematical approach to space groups; crystal lattices; point groups and crystal classes; symbols for symmetry operations; symbols for space groups; isomorphic subgroups of space groups; lattice complexes; and normalizers of space groups.  Volume A is designed not only for professional crystallographers, but also for chemists, physicists, mineralogists, biologists and material scientists who employ crystallographic methods and who are concerned with the structure and the properties of crystalline materials.  The fifth edition of Volume A has been reviewed by P. Paufler [Acta Cryst. (2004). A60, 641-642]. The first edition was reviewed by K. M. Stadnicka, B. J. Oleksyn and K. Z. Sokalski [Acta Cryst. (1987). A43, 156-159].  International Tables for personal use can be purchased at a discount. Contact Customer Service for further information and to place an order.  Written for:  Academia, researchers and industry involved with the use and determination of crystal structures |  | Print version  Recommend to others   Additional information  Reviews   All books by this editor  Hahn, Theo     Related subjects  Chemistry  Geoscience  Geosciences  Materials  Physics |  |

Help

|

Login

|

Contact

|

Shopping cart

|

About us

|

Terms & conditions

|

Now on Sale

  

Privacy statement

|

© Springer. Part of Springer Science+Business Media

|

Sitemap
