# Supplementary material for: Space groups and crystallographic symmetry: writing a multi-featured tutorial in a new style
Source: Acta Crystallogr E Crystallogr Commun. 2021 Jul 16;77(Pt 9):857–63. doi: 10.1107/S2056989021007039 (PMC8423017; doi:10.1107/S2056989021007039)
Supplement: Supplementary file 1 [file e-77-00857-sup2.zip › symandsg/Main/wardpubs.html]

Publications

|  |  |
| --- | --- |
| ***M. D. Ward Group***  **Chemical Engineering & Materials Science  University of Minnesota - Twin Cities** | ***Crystal Growth and Design*** |

---


**Publications** 

(You may need a proper IP or a user ID to read pdf
files in some journals.)

158. "Hydrogen-bonded host frameworks with tunable cavities: structural
characterization and inclusion-based separations of molecular isomers", M. J.
Horner, S. Grabowski, K. Sandstrom, K. T. Holman, M. Bader, M. D. Ward, W.-S.
Kim, *ACA Transactions* **2004**, *39*, 130.

157. "Dynamic behavior of particle agglomeration of europium oxalate during
reaction crystallization in semi-batch reactor",
W.-S. Kim, W.-S. Kim, K.-S. Kim, J.-S. Kim, T. Jung, and M. D. Ward, 
*Chem. Eng. Commun.* **2006**, *193*, 370.

156. "Design of Crystalline
Molecular Networks with Charge-Assisted Hydrogen Bonds", M. D. Ward, 
*Chem. Commun.* **2005**, 5838.

155. "Directing the Assembly
of Molecular Crystals", M. D. Ward, 
*MRS Bulletin* **2005**, *30*, 705.

154. "Chiral Discrimination in Low-Density Hydrogen-Bonded Frameworks", R.
Custelcean, and M. D. Ward, *Cryst. Growth Des.* **2005**, *5*,
2277.

153. "Crystal Surface Adhesion
Explains the Pathological Activity of Calcium Oxalate Hydrates in Kidney
Stone Formation", X. Sheng, M. D. Ward and J.
A. Wesson, 
*J. Am. Soc. Nephrol* **2005**, *16*, 1904.

152. "Snapshots of Crystal Growth", M. D. Ward, 
*Science* (Perspectives) **2005**, *308*, 1566.

151. "Lyotropic Phases Reinforced
by Hydrogen Bonding", S. M. Martin, and M. D. Ward, 
*Langmuir* **2005**, *21*, 5324.

150. "Structural Transference and
Regulation of Bimodal Assembly of Charge-Assisted Hydrogen-Bonded Networks", S.-O. Lee, D. M. Shacklady,
M. J. Horner, S. Ferlay, M. W. Hosseini, and M. D. Ward, 
*Cryst. Growth Des.* **2005**, *5*, 995.

149. "Thermotropic Properties
of Organic Nanocrystals Embedded in Ultrasmall Crystallization Chambers",
J.-M. Ha, M. A. Hillmyer, and M. D. Ward,
*J. Phys. Chem. B*, **2005**, *109*, 1392.

148. "Adhesion at calcium oxalate crystal surfaces and the effect of urinary
constituents",
X. Sheng, T. Jung, J. A. Wessen, and M. D. Ward, *Proc. Natl. Acad. Sci. USA*, **2005**, *102*, 267. 

147. "Structure and order in soft matter: symmetry transcending length scale",
M. D. Ward, and M. J. Horner, *Cryst. Eng.
Comm.*, **2004**, *6*, 401. 

146. "Probing crystallization of calcium oxalate monohydrate and the role of
macromolecule additives with in situ atomic force microscopy",
T. Jung, X. Sheng, C. K. Choi, W.-S. Kim, J. A. Wesson, and M. D. Ward,
*Langmuir*, **2004**, *20*, 8587.

145. "Rheology and morphology of smectic liquid crystal/polymer blends",
J. Yonezawa, S. M. Martin, C. W. Macosko, and M. D. Ward,
*Macromolecules* **2004**, *37*, 6424.

144. "Structure and rheology of hydrogen bond reinforced liquid crystals",
S. M. Martin, J. Yonezawa, M. J. Horner, C. W. Macosko, and M. D. Ward,
*Chem. Mater.*,
**2004**, *16*, 3045.

143. "Organic thin-film transistors based on tolyl-substituted oligothiophenes",
S. Mohapatra, B. T. Holmes, C. R. Newman, C. F. Prendergast, C. D. Frisbie,
and M. D. Ward,
 *Adv. Func. Mater.*, **2004**,  *14*,
605.

142. "Structural Characterization of a Pentacene Monolayer on an Amorphous SiO2
Substrate with Grazing Incidence X-ray Diffraction",
S. E. Fritz, S. M. Martin, C. D. Frisbie, M. D. Ward, and M. F. Toney,
 *J. Am. Chem. Soc.*, **2004**,  *126*,
4084.

141. "Polymorph Selectivity under Nanoscopic Confinement",
J.-M. Ha, J. H. Wolf, M. A. Hillmyer, and M. D. Ward,
 *J. Am. Chem. Soc.*, **2004**,  *126*,
3382.

140. "Crystal Agglomeration of Europium Oxalate in Reaction Crystallization Using
Double-Jet Semi-Batch Reactor",
W.-S. Kim, W.-S. Kim, K.-S. Kim, J.-S. Kim, and M. D. Ward,  *Mater. Res. Bull.*, **2004**,  *39*,
283.

139. "Two-Dimensional Host-Guest Inclusion Crystals at the Air-Water Interface", 
D. J. Plaut, S. M.
Martin, K. Kjaer, M. J. Weygand , M. Lahav, L. Leiserowitz, I. Weissbuch and M.
D. Ward, *J. Am. Chem. Soc.*, **2003**, *125*, 15922-15934.

138. "Molecular Fuel Tanks",
M. D. Ward, *Science*
(Perspectives), **2003**, *300*, 1104.

137. "Study of the (001) Cleavage Planes of Guanidinium Methanesulfonate Single Crystals by AFM and He Diffraction",
G. Bracco, Y. Hu, J. Acker, and M. D. Ward, *Appl. Surf. Sci.*
, **2003**, *212-213*, 151.

136. "Atomic Diffraction Study of the Interaction of Helium Atoms with the Surface
of an Organic Single Crystal: the (001) Cleavage Planes of Guanidinium Methanesulfonate",
G. Bracco, J. Acker, M. D. Ward, and G.
Scoles, *J. Chem. Phys*., **2003**, *118*, 8405. [Selected for the
May 5,
2003
issue of the *Virtual Journal of Nanoscale
Science and Technology*; http://www.vjnano.org]

135. "Tricyanovinyl-Substituted
Oligothiophenes",
M. M. Bader, R. Custelcean, M. D. Ward, *Chem. Mater*. **2003**; *15*, 616.

134. "Adhesion between Molecules and Calcium Oxalate Crystals: Critical
Interactions in Kidney Stone Formation",
X. Sheng, M. D. Ward, and J. A. Wesson, *J. Am. Chem. Soc*., **2003,** *125*, 2854.
[Highlighted in *Nature*, **2003**, *422*, 275].

133. "Helium Diffraction Study of Polar and Non-Polar (001) Surfaces of a Layered
Hydrogen-bonded Crystal",
G. Bracco, J. Acker, M. D. Ward, and G. Scoles, *Langmuir*, **2002**, *18*, 5551.

132. "Hydrogen-bonded Helices in Crystals with Prescribed Organization",
R. Custelcean and M. D. Ward, *Angew. Chem*. *Int. Ed.*
**2002**, *41*, 1724.

131. "Direct
Visualization of Calcium Oxalate Monohydrate (COM) Crystallization and
Dissolution with Atomic Force Microscopy (AFM) and the Role of Polymeric
Additives", Shouwu Guo,
Michael D. Ward, and Jeffrey A. Wesson, *Langmuir* **2002**, *18*, 4284.

130. "Probing
Vibrational Dynamics of Hydrogen-bonded Inclusion Compounds with Inelastic
Neutron Scattering and *ab initio* Calculations",
A. M. Pivovar, M. D. Ward, C. M. Brown, and D. A. Neumann, *J. Phys. Chem*. *B*, **2002**, *106*, 4916.

129. "Engineering Space Group Symmetry and Polar Order in Molecular Host
Frameworks",
K. T. Holman, A. M. Pivovar, M. D. Ward, *Science*
**2001**, *294*, 1907.

128. "Lamellae-Nanotube
Isomerism in Hydrogen-Bonded Host Frameworks", 
M. J. Horner, K. T. Holman, M. D. Ward, *Angew. Chem. Int. Ed.* **2001**, *40*, 4045.

127. "Shape Selective Separation of Molecular Isomers with Tunable Hydrogen-bonded
Host Frameworks",
A. M. Pivovar, K. T. Holman and Michael D. Ward, *Chem. Mater.* **2001**, *13*, 3018.

126. "Vibrational
Mode Analysis of Isomorphous Hydrogen-Bonded Guanidinium Sulfonates with
Inelastic Neutron Scattering and Density Functional Theory",
A. M. Pivovar, M.
D. Ward, T. Yildirim, D. A. Neumann, *J.
Chem. Phys*., **2001**, *115*, 1909.

125. "Electrochemical
Growth of Epitaxial Organic Films",
M. D. Ward,
Encyclopedia of Electrochemistry, Volume 10, “Modified Electrodes”

124. "Selective
Nucleation and Discovery of Organic Polymorphs through Epitaxy with Organic
Single Crystal Substrates",
C. Mitchell, L. Yu, and M. D. Ward, *J. Am. Chem. Soc*, **2001**, *123*, 10830.

123. "The Generality of Architectural Isomerism in Designer Inclusion Frameworks",
K. T, Holman, S. M. Martin, D. P. Parker and M. D. Ward, *J. Am. Chem. Soc*. **2001**,
*123*, 4421.

122. "Epitaxy
and Molecular Organization on Solid Substrates",
D. E. Hooks, T. Fritz and M. D. Ward, *Adv. Mater.* **2001**, *13*, 227.

121. "Metric Engineering of Soft Molecular Host Frameworks",
K. T. Holman, A. M. Pivovar, J. A. Swift and M. D. Ward,
invited article, *Acc. Chem. Res*., **2001**, *34*, 107.

120. "Bulk Crystals to Surfaces: Combining X-ray Diffraction and Atomic Force
Microscopy to Probe the Structure and Formation of Crystal Interfaces",
M. D. Ward, *Chem. Rev.,* **2001**, *101*, 1697.

119. "Plastic Sandwiches `a la carte", 
M. D. Ward,
*Nature*
(News and Views), **2000**, *405*, 293.

118. "Building Molecular Frameworks with Tailored Pore Structures", 
D. J. Plaut, K. T. Holman,
A. M. Pivovar and M. D. Ward, *J. Phys. Org. Chem.* Special Issue “Materials for the 21st Century,”
**2000**, *13*, 858.

117. "Crystal Engineering with Soft and Topologically Adaptable Molecular Host
Frameworks",
K. T. Holman and M. D. Ward,  *Stimulating
Concepts in Chemistry*, Vogtle, Stoddart, and Shibasaki, Eds., Wiley-VCH,
Weinheim, **2000**, p221.

116. "Metric Engineering of Crystalline Inclusion Compounds by Structural Mimicry", 
K. T. Holman and M. D. Ward, *Angew. Chem. Int.
Ed. Engl*., **2000**, *39*, 1653.

115. "Low-density hydrogen-bonded networks in crystals and at the air-water
interface",
D. J. Plaut, K. M. Lund, and M. D. Ward, *Chem.
Comm*., **2000**, 769.

114. "Cooperative Polar Ordering in Topologically Constrained Crystalline
Inclusion Compounds",
J. A. Swift and M. D. Ward, *Chem.
Mater.* **2000**, *12*, 1501.

113. "Control of Hierarchical Order in Crystalline Composites of Diblock Copolymers and a Molecular Chromophore",
C. C. Evans, F. S. Bates, and M. D. Ward, *Chem.
Mater.* **2000**, *12*, 236.

112. "Structural Studies of a Crystalline Insulin Analog Complex with Protamine by
Atomic Force Microscopy",
C. M Yip, M. L. Brader, B. H. Frank, M. R. DeFelippis and M. D. Ward, *Biophys. J.*, **2000**, *78*, 466.

111. "Electrochemical Quartz Crystal Microbalance",
M. D. Ward, *Encyclopedia
of Analytical Chemistry*, R. A. Meyers, Ed., John Wiley & Sons,
Chichester, 9930 – 9958, **2000**.

110. "Self-Assembly of Crystalline Lattices",
M. D. Ward and A. M. Pivovar,  *The Encyclopedia of Materials Science and Technology, 2nd edition,* **1999**.

109. "Organic Host-Guest Molecular Assemblies",
M. D. Ward and A. M. Pivovar, *Current Opinion in* *Solid
state and Materials Science*, **1999**,
*4*, 581.

108. "Automated Electrochemical Analysis with Combinatorial Electrode Arrays",
M. G. Sullivan, H. Utomo, P. J. Fagan and M. D. Ward, *Anal.
Chem*., **1999**; *71*, 4369.

107. "The Physicochemical Origins of Coincident Epitaxy in Molecular Overlayers:
Lattice Modeling vs. Potential Energy Calculations",
J. A. Last, D. E. Hooks, A. C. Hillier and M. D. Ward, *J. Phys. Chem. B*,
**1999,** *103*, 6723.

106. "Steric
Control of Architectural Isomerism in Hydrogen Bonded Crystalline Clathrates",
C. C. Evans, L. Sukarto and M. D. Ward, *J.
Am. Chem. Soc*., **1999**, *121*, 320.

105. "Surfactant Enhanced Spreading",
T. Stoebe, R. M. Hill, M. D. Ward, L. E. Scriven and H. T. Davis,
"Silicone Surfactants," *Surfactant Science* Series, *Vol.
86*, R. M. Hill, Ed.; Marcel Dekker, New York, **1999**.

104. "Chemistry on the Edge: A Microscopic Analysis of the Intercalation,
Exfoliation, Edge Functionalization, and Monolayer Surfacing Tiling
Reactions of a-Zirconium Phosphate",
D. M. Kaschak, S. A. Johnson, D. E. Hooks, H.-N. Kim, M. D. Ward, T.
E. Mallouk,
*J. Am. Chem. Soc.* **1998**, *120*, 10887.

103. "Cooperative Host-Guest Recognition in Crystalline Clathrates: Steric Guest
Ordering by Molecular Gears",
J. A. Swift, A. M. Reynolds and M. D. Ward, *Chem. Mater*., **1998**, *10*, 4159.

102. "Nanoconfined
Electrochemical Nucleation of Crystalline Molecular Monolayers on Graphite
Substrates",
D. E. Hooks, C. M. Yip, and M. D. Ward, *J. Phys. Chem.
B.* **1998**,
*102*, 9958.

101. "Molecular Scaffolds",
M. D. Ward, *Chem*. *Brit*., **1998**, *34*, 52.

100. "Structural and Morphological Characterization of Ultralente Insulin Crystals by Atomic
Force Microscopy:Evidence of Hydrophobically Driven Assembly",
C. M. Yip, M. R. DeFelippis, B. H. Frank, M. L. Brader and M. D. Ward, *Biophys. J*., **1998**, *75*, 1172.

99. "Template Induced Architectural Isomerism in Porous Molecular Lattices:
Engineering of Crystalline Clathrates",
J. A. Swift, A.M. Pivovar, and A. M. Reynolds, *J. Am. Chem. Soc.*, **1998**, *120*, 5887.

98. "Direct Force Measurements of Insulin Monomer-Monomer Interaction Forces",
C. M. Yip, C. C. Yip and M. D. Ward,  *Biochem.* **1998**, *37*, 5439.

97. "Atomic Force Microscopy of Crystalline Insulins: The Influence of Sequence
Variation on Crystallization and Interfacial Structure",
C. M. Yip, M. L. Brader, M. R.
DeFelippis and M. D. Ward, *Biophys. J.* **1998**, *74*, 2199.

96. "Interweaving of Two-Dimensional Hydrogen-Bonded Networks Directed by
Chloride Ions",
V. A. Russell and M.D. Ward, *New J. Chem.*,
Special Issue on Supramolecular Chemistry, **1998**,
*22*, 149.

95. "Epitaxially-Driven
Assembly of Crystalline Molecular Films on Ordered Substrates",
J. A. Last, A. C. Hillier, D. E. Hooks, J. B. Maxson, and M. D. Ward, *Chem. Mater.*, **1998**, *10*, 422.

94. "Structure
and Physical Properties of Hexacyanomanganate(IV), [MnIV(CN)_6_]^2-^",
W. E. Buschmann, C. Vazquez, R. S. McLean, M. D. Ward, N. L. Jones, J. S.
Miller, *J. Chem. Soc. Chem. Commun.* **1997**, 409. 

93. "Organoporous
Hosts with Adjustable Molecular Environments",
J. A. Swift, V. A. Russell and M. D. Ward, *Adv.
Mater.* **1997**,
*9*, 15.

92. "Superspreading
of Aqueous Films Containing Trisiloxane Surfactant on Mineral Oil",
T. Stoebe, Z. Lin, R. M. Hill, M. D. Ward and H. Ted Davis,  *Langmuir* **1997**, *13*, 7282.

91. "Enhanced
Spreading of Aqueous Films Containing Ionic Surfactants on Solid Substrates",
T. Stoebe, R. M. Hill, M. D. Ward, and H. Ted Davis, *Langmuir,* 
**1997**, *13*, 7276.

90. "Enhanced
Spreading of Aqueous Ethoxylated Alcohol Surfactant Systems on Solid Substrates",
T. Stoebe, Z. Lin, R. M. Hill, M. D. Ward, and H. Ted Davis,  *Langmuir* **1997**, *13*, 7270.

89. "Two-Dimensional
Hydrogen-Bonded Assemblies: The Influence of Steric Effects and Competitive
Hydrogen Bonding on the Structures of Guanidinium Arenesulfonate Networks",
V. A. Russell and M.D. Ward, *J. Mater.
Chem.* "Special Issue on
Molecular Assemblies and Nanostructures," **1997**, *7*, 1123.

88. "Nanoporous
Molecular Sandwiches: Pillared Two-Dimensional Hydrogen-Bonded Networks with
Adjustable Porosity",
V. A. Russell, C. Evans, W. Li and M. D. Ward, *Science*,
**1997**, *276*, 575.

87. "Nanoscale
Visualization of Crystal Habit Modification by Atomic Force Microscopy",
G. Mao,
L. Lobo, R. Scaringe and M. D. Ward, *Chem. Mater.* **1997**,  *9*, 773.

86. "Organic Crystal Surfaces: Structure, Properties and Reactivity",
M. D. Ward, *Current
Opinion in Colloid and Interfacial Science*, **1997**, *2*, 51.

85. "Nucleation
of Molecular Crystals at Guanidinium Alkylsulfonate Langmuir Monolayers",
L. M. Frostman and M. D. Ward, *Langmuir*, **1997**, *13*, 330.

84. "Epitaxial
Interactions between Organic Overlayers and Ordered Substrates",
A. C. Hillier and M. D. Ward, *Phys. Rev. B*, **1996**, 54, 14037.

83. "Probing Solvent Dynamics in Concentrated Polymer Films with a High Frequency
Shear Mode Quartz Resonator",
A. Katz and M . D. Ward, *J. Appl. Phys.*, **1996**,
*80*, 4153.

82. "Electrochemical
Annealing of Domains in Epitaxial Molecular Films",
J. A. Last and M. D. Ward,  *Adv. Mater.*, **1996**, *8*, 730.

81. "Atomic Force Microscopy of Insulin Single Crystals: Direct Visualization of
Molecules and Crystal Growth",
C. M. Yip and M. D. Ward, *Biophys.
J.*  **1996**, *71*, 1071.

80. "Molecular Crystals with Dimensionally Controlled Hydrogen-Bonded
Nanostructures",
V. A. Russell and M. D. Ward,  *Chem. Mater.*, **1996**, *8*, 1654.

79. "Determination of Contact Angles and Surface Tensions with the Quartz Crystal
Microbalance",
Z. Lin and M. D. Ward, *Anal.
Chem.* **1996**, *68*,
1285.

78. "Surfactant Enhanced Spreading",
T. Stoebe, Zuxuan Lin, Randal M. Hill, H. Ted Davis, and M. D. Ward, *Langmuir*, **1996**, *12*, 337.

77. "Improved Accuracy in Dynamic Quartz Crystal Microbalance Measurements of
Surfactant Enhanced Spreading",
Z. Lin, T. Stoebe, R. M. Hill, H. T. Davis, and Michael D. Ward, *Langmuir*, **1996**, *12*, 345.

76. "Scanning Tunneling and Atomic Force Microscopy of Electrochemical Interfaces
(Invited Book Chapter)",
M. D. Ward and H. S. White, *Modern Techniques in
Electroanalysis*, Petr Vanysek, Ed.; Volume 139, Wiley (New York), **1996**,
p 107.

75. "Solid-State
Structure of a Layered Hydrogen-Bonded Salt: Guanidinium
5-Benzoyl-4-Hydroxy-2-Methoxybenzenesulfonate Methanol Solvate",
V. A. Russell and M. D. Ward, *Acta Cryst.,* **1996**, B52, 209-214.

74. "Structural
and Physical Properties of Delocalized Mixed-Valent [Cp\*M(pentalene)M’Cp\*]n+ and
[Cp\*M(indacene)M’Cp\*]n+ (M,M’ = Fe, Co, Ni; n = 0, 1, 2) Complexes",
J. M. Manriquez, M. D. Ward, W. M. Reiff, J. C. Calabrese, N. L. Jones, P. J. Carroll,
E. E. Bunel, J. S. Miller,  *J. Am. Chem. Soc.* **1995**, *117*,
6182.

73. "Selective
Nucleation of an Organic Polymorph by Ledge-Directed Epitaxy on a Molecular
Crystal Substrate",
S. Bonafede and M. D. Ward, *J. Am. Chem. Soc.*, **1995**,
*117*, 7853.

72. "Solubilization
Control by Redox-Switching of Polysoaps",
P. Anton, A. Laschewsky, and M. D. Ward, *Polymer Bull.* **1995**, *34*, 331.

71. "V_2_O_5_ Xerogel
Films as Intercalation Hosts for Lithium I. Insertion Stoichiometry, Site
Concentration and Specific Energy",
H.-K. Park, W. H. Smyrl, and M. D. Ward,  *J. Electrochem. Soc.*, 142, **1995**, 1068.

70. "Molecular
Nanoclusters as Precursors to Conductive Thin Films and Crystals",
A. C. Hillier, J. Hossick Schott, and M. D. Ward, *Adv.
Mater.* (Research News),  **1995**, *7*, 409.

69. "The Role of Longitudinal Waves in Quartz Crystal Microbalance Applications
in Liquids",
Z. Lin and M. D. Ward, *Anal. Chem.,* **1995**, *67*, 685.

68. "Molecular
Engineering of Crystals by Electrostatic Templating",
P. J. Fagan and M. D. Ward,
*Perspectives in Supramolecular Chemistry,* G. R. Desiraju, Ed., John Wiley,
New York, **1995**.

67. "Self-Assembly
of Low-Dimensional Molecular Nanoclusters on Au(111) Surfaces",
J. Hossick Schott and M. D. Ward, *Langmuir*, **1995**, *11*, 177.

66. "Principles and Applications of the Electrochemical Quartz Crystal
Microbalance",
M. D. Ward,
*Monographs in Electroanalytical Chemistry and Electrochemistry: Physical
Electrochemistry, Principles, Methods, and Applications,* I. Rubinstein, Ed.,
**1995**, Marcel Dekker, New York.

65. "Nanoscale
Imaging of Molecular Adsorption",
H. Cai, A. C. Hillier, K. Franklin, C. C. Nunn, and M. D. Ward, *Science*, **1994**, *266*, 1551.

64. "Electrocrystallization
of an Ordered Organic Monolayer: Selective Epitaxial Growth of 
b-(ET)2I3
on Graphite",
A. C. Hillier, J. B. Maxson and M. D. Ward, *Chem. Mater.*  **1994**, *6*, 2222.

63. "Determination
of Wetting Velocities of Surfactant Superspreaders with the Quartz Crystal
Microbalance",
Z. Lin, R. M. Hill, H. T. Davis, and M. D. Ward, *Langmuir*,
**1994**, *10*, 4060.

62. "Guanidinium
para-Substituted Benzenesulfonates: Competitive Hydrogen Bonding in Layered
Structures and the Design of Nonlinear Optical Materials",
V. A. Russell, M. C. Etter and M. D. Ward, *Chem.
Mater.* **1994**, *6*,
1206.

61. "Scanning Tunneling Microscopy of Layered Molecular Conductors",
M. A. Dvorak and M. D. Ward, *Chem.
Mater.* **1994**, *6*, 1386.

60. "Layered
Materials by Molecular Design: Structural Enforcement by Hydrogen Bonding in
Guanidinium Alkyl- and Aryl Sulfonates",
V. A. Russell, M. C. Etter and M. D. Ward, *J. Am. Chem. Soc*., **1994**, *116*, 1941.

59. "Snapshots
of Crystal Growth: Nanoclusters of Organic Conductors on Au(111) Surfaces",
J. Hossick Schott and M. D. Ward, *J. Am. Chem. Soc*., **1994**, *116*,
6806.

58. "Atomic Force Microscopy of the Electrochemical Nucleation and Growth of
Molecular Crystals",
A. Hillier and M. D. Ward, *Science*, **1994**, *268*,
1261.

57. "Directing
Polymorph Selectivity During Nucleation of Anthranilic Acid on Molecular
Substrates",
P. W. Carter and M. D. Ward, *J. Am. Chem. Soc.*, **1994**,
*116*, 769.

56. "Nucleation
and Growth of Molecular Crystals on Self-Assembled Monolayers",
L. M. Frostman and M. D. Ward, *Langmuir*, **1994**, *10*, 576.

55. "Self-Assembled
Monolayers with Charge-Transfer Groups: 16-Mercaptohexadecyl Tetrathiafulvalene
Carboxylate on Gold",
C. M. Yip and M. D. Ward, *Langmuir*, **1994**,  *10*, 549.

54. "Nanoscale
Surface Topography and Growth of Molecular Crystals: The Role of Anisotropic
Intermolecular Bonding",
A. Hillier, P. W. Carter and M. D. Ward, *J. Am.
Chem. Soc.*, **1994**, *116*, 944.

53. "Molecular Single Crystal Interfaces: Topographical Structure and Crystal
Growth",
P. W. Carter, A. C. Hillier and M. D. Ward, *Mol. Crystal. Liq. Cryst.*
(Proceedings of ICCOSS XI), **1994**, *242*, 53.

52. "Depletion Layer Effects on the Response of the Electrochemical Quartz
Crystal Microbalance",
W.-W. Lee, H. S. White and M. D. Ward, *Anal. Chem.*, **1993**,
*65*, 3232.

51. "Topographically Directed Nucleation of Organic Crystals on Molecular Single
Crystal Substrates",
P. W. Carter and M. D. Ward, *J. Am. Chem. Soc.*, **1993**, *115*,
11521.

50. "Synthesis
and Properties of Optically Active Helical Metallocene Oligomers",
T. J. Katz, A. Sudhakar, M. F. Teasley, A. M. Gilbert, W. E. Geiger, M. P. Robben, M. Wuensch
and M. D. Ward, *J. Am. Chem Soc.* **1993**, *115*, 3182.

49. "Operation
of an Ultrasensitive 30 MHz Quartz Crystal Microbalance in Liquids",
Z. Lin, C. M. Yip, I. S. Joseph and M. D. Ward, *Anal. Chem*. **1993**,
*65*, 1546.

48. "Scanning Tunneling Microscopy Studies of Organic Conductors",
S. Li, H. S. White and M. D. Ward, *Synthetic Metals*, **1993**, *55-57*, 426.

47. "Real-time Measurement of Anchorage-Dependent Cell Adhesion Using a Quartz
Crystal Microbalance",
D. Gryte, M. D. Ward and W.-S. Hu, *Biotechnol. Prog.*  **1993**, *9*, 105.

46. "Piezoelectric
pH Sensors: AT-cut Quartz Resonators with Amphoteric Polymer Films",
J. Wang, M. D. Ward, R. C. Ebersole and R. A. Foss, *Anal. Chem*. **1993**,
*65*, 2553.

45. "Self-assembled
Thiol Monolayers with Carboxylic Acid Functionality: Measuring pH-Dependent
Phase Transitions with the Quartz Crystal Microbalance",
J. Wang, L. M. Frostman and M. D. Ward, *J. Phys. Chem.* **1992**, *96*, 5224.

44. "Scanning
Tunneling Microscopy of the Organic Semiconductor [(h^5^-C_5_Me_5_)_2_Ru(h^6^,h^6^-[22]
(1,4)-cyclophane)][TCNQ]_4_", S. Li, H. S. White and M. D. Ward, *Chem. Mater.* **1992**, *4*,
1082.

43. "Scanning
Tunneling Microscopy of the Organic Conductor (TMTSF)_2_X
(X = ClO_4_^-^,
ReO_4_^-^)",
S. Li, H. S. White and M. D. Ward, *J. Phys. Chem.* **1992**, *96*,
9014.

42. "Scanning electrochemical mass sensitivity mapping of the quartz crystal
microbalance in liquid media",
A. C. Hillier and M. D. Ward, *Anal. Chem.* **1992**, 64,
2539.

41. "Measurement of Interfacial Processes at Electrode Surfaces with the
Electrochemical Quartz Crystal Microbalance",
D. A. Buttry and M. D. Ward, *Chem. Rev.*
(invited review), **1992**, *92*. 1355.

40. "Piezoelectric Cell Growth Sensor",
R. C. Ebersole, R. P. Foss and M. D. Ward, *Bio/. Technology*,
**1991**, *9*, 450.

39. "Radial Sensitivity of the Quartz Crystal Microbalance in Liquid Media",
M. D. Ward and E. J. Delawski, *Anal. Chem.*, **1991**, *63*, 886.

38. "Redox-Active
Polymers by Group Transfer Polymerization: Physical, Electrochemical and
Electronic Properties",
A. Laschewsky and M. D. Ward, *Polymer*, **32**,
146, **1991**.

37. "Oxidation
Chemistry of d^0^ Transition
Metal Complexes",
M. J. Burk, W. A. Tumas, M. D. Ward and D. R. Wheeler, *J.
Am. Chem. Soc.*, **112**, 6133, **1990**.

36. "Building Molecular Crystals",
P. J. Fagan and M. D. Ward, *Scientific American*, July
**1992**.

35. "In-situ Interfacial Mass Measurements with Piezoelectric Transducers:
Fundamentals and Applications",
M. D. Ward and D. A. Buttry, *Science*, **249**, 1000,  **1990**.

34. "Low
Dimensional Organometallic Charge Transfer Complexes. X-Ray Structure and
Magnetic Properties of 
a-
and 
b-[Cr(C_6_Me_3_H_3_)_2_^+^][TCNQ-]",
D. O'Hare, M. D. Ward and J. S. Miller, *Chem. Mater*. **2**,
758, **1990**.

33. "Electrochemical
and Quartz Crystal Microbalance Evidence for Mediation and Direct
Electrochemical Reactions of Small Molecules at
Tetrathiafulvalene-tetracyanoquinodimethane (TTF-TCNQ) Electrodes",
M. S. Freund, A. Brajter-Toth and M. D. Ward, *J. Electroanal. Chem.*, **289**, 127,
**1990**.

32. "Spontaneously
Formed Functionally Active Avidin Monolayers on Metal Surfaces: A Strategy for
Immobilizing Biological Reagents and Design of Piezoelectric Biosensors", R. C. Ebersole,
J. R. Moran and M. D. Ward, *J. Am. Chem. Soc* , **112**,
3239, **1990**.

31. "Molecular
Control of Self-Assembled Monolayer Films of Imidazole-2 Thiones: Adsorption and
Reactivity",
A. J. Arduengo, J. R. Moran and M. D. Ward, *J. Am. Chem. Soc.*,
**112**, 6153, **1990**.

30. "Single-Crystal
EPR Study of Triplet Excitons in [Fe(mesitylene)_2_^2+^][C_3_(C(CN)_2_)_3_^-^]_2_", J. R. Morton, K. F. Preston, Y. Le Page, A. J. Williams and M. D. Ward, *J.
Chem. Phys.*, **93**, 2222, **1990**.

29. "Stuctural
and Magnetic Characterization of Electron Transfer Salts of
Tris(dicyanomethylene)cyclopropanide, C_3_[C(CN)_2_]_3_-,
and Fe(C_5_Me_5_)^2+^ (1:1;
Two Phases) and Fe(C_6_H_3_Me_3_)_2_^2+^ (1:2),
Evidence for 1-D Antiferromagnetic Behavior in Segregated [Fe(C_5_Me_5_)^2+^]Chains",
J. S. Miller, M. D. Ward, J. H. Zhang and W. M. Reiff, *Inorg. Chem.,*  **29**, 4063, **1990**.

28. "Hexaazaoctadecahydrocoronene,
HOC. Structural and Physical Properties of [HOC]_n_ (n
= 0, 1+, 2+, 3+, 4+)",
J. S. Miller, D. A. Dixon, J. C. Calabrese, C. Vazquez, P. J. Krusic, M. D.
Ward and R. L. Harlow, *J. Am. Chem. Soc.*, **112**,
381, **1990**.

27. "Magnetic
Coupling Relationships for Bis(Metallocene)-Benzene Complexes",
J. M. Manriquez, M. D. Ward, J. C. Calabrese, P. J. Fagan, A. J. Epstein and
J. S. Miller,  *Mol. Cryst. Liq. Cryst.* **176**, 527, **1989**.

26. "^57^Fe
Mossbauer Spectroscopy Studies of New Examples of Fully Averaged Mixed Valence
Diiron and Iron/Ruthenium Compounds",
W. M. Reiff, J. M. Manriquez, M. D. Ward and J. S. Miller,  *Mol. Cryst. Liq. Cryst.* **176**, 423, **1989**.

25. "Probing Electrocrystallization of Charge Transfer Salts with the Quartz
Crystal Microbalance", *J. Electroanal. Chem.* , **273**, 79, **1989**.

24. "Single
Crystal EPR Spectra of Triplet Excitons in [(h5-C_5_Me_5_)Ru(h6,
h6-[22](1,4)-cyclophane)Ru(h5-C_5_Me_5_)][TCNQ]_4_", J. R. Morton, K. F. Preston, M. D. Ward and P. J. Fagan, *J. Chem. Phys.*,
**90**, 2148, **1989**.

23. "Molecular
Engineering of Solid State Materials:Organometallic Building Blocks",
P. J. Fagan, M. D. Ward, and J. C. Calabrese, *J. Am. Chem. Soc*, **111**,
1698, **1989**.

22. "Electrostatic
Structural Enforcement in Low Dimensional Solids: Synthesis, Structure and
Electronic Properties of Polycationic Ruthenium Complexes with Polycyanoanions",
M. D. Ward, P. J. Fagan, J. C. Calabrese and D. C. Johnson, *J. Am. Chem.
Soc*, **111**, 1719, **1989**.

21. "Organometallic
Donor-Acceptor Complexes with Non-Planar Donors: The Zig-Zag Linear Chain
Complex [(h6-C_6_Me_6_)_2_M][iso-C_4_(CN)_6_]
(M=Fe, Ru)",
M. D. Ward and J. C. Calabrese, *Organometallics*, **8**,
593, **1989**.

20. "Electrocrystallization
of Low Dimensional Solids: Directed Selectivity and Investigation of Crystal
Growth with the Quartz Crystal Microbalance",
M. D. Ward, *Synthetic Metals*,
**27**, B211-B218, **1988**.

19. "Electrochemical Aspects of Low-Dimensional Molecular Solids",
A. J. Bard, Ed., *Electroanal. Chem.* 161,**1988**.

18. "Amplified
Mass Immunosorbent Assay with a Quartz Crystal Microbalance",
R. C. Ebersole and M. D. Ward, *J. Am. Chem. Soc*, **110**, 8623, **1988**.

17. "Synthesis
and Unusual Properties of a Helically Twisted Metallated Rubrene Derivative,
P. J. Fagan, M. D. Ward, J. V. Caspar, J. C. Calabrese and P. J. Krusic, *J.
Am. Chem. Soc.*, **110**, 2981, **1988**.

16. "Ion
Exchange of Ferro(ferricyanide) into Polyvinylferrocene Films",
M. D. Ward, *J. Electrochem.
Soc.* **135**, 2747, **1988**.

15. "Investigation
of Open Circuit Reactions of Polymer Films Using the Quartz Crystal
Microbalance. Reactions of Polyvinylferrocene Films",
M. D. Ward, *J. Phys.
Chem,* **92**, 2049, **1988**.

14. "Electrocrystallization,
Structural and Physical Properties of Charge Transfer Complexes Derived >From [(h6-C_6_Me_6_)_2_M]^2+^ (M=Fe,Ru)
and TCNQ (TCNQ = tetracyanoquinodimethane)",
M. D. Ward and D. C. Johnson,  *Inorg. Chem.* **26**, 4213, **1987**.

13. "Methane
Photoactivation on Copper Molybdate. An Experimental and Theoretical Study",
M. D. Ward, J. F. Brazdil, S. P. Mehandru and A. B. Anderson, *J. Phys. Chem.*,
**91**, 6515, **1987**.

12. "Investigation
of Electron Transfer Processes at Open Circuit Polyvinylferrocene Films Using
Rotating Ring-Disk Electrodes",
M. D. Ward, *J. Electroanal. Interfac. Chem.*,
**236**, 139, **1987**.

11. "Characaterization
of the Charge Transfer Reaction Between Decamethylferrocene and
7,7,8,8-Tetracyano-p-quinodimethane (1:1). The
57Fe Mossbauer Spectra and
Structures of the Paramagnetic, Dimeric and Metamagnetic One-Dimensional Salts
and the Molecular and Electronic Structures of [TCNQ]_n_ (n =0, 1-, 2-)",
J. S. Miller, J. H. Zhang, W. M. Reiff, D. A. Dixon, L. D. Preston, A. H. Reis, Jr.,
E. Gebert, M. Extine, J. Troup, A. J. Epstein and M. D. Ward, *J. Phys. Chem.*,
**91**, 4344, **1987**.

10. "Linear
Chain Organometallic Donor-Acceptor Complexes and One- Dimensional Alloys.
Synthesis and Structure of [(h
6-C6Me3H3)2M][C6(CN)6]
(M=Fe, Ru)",
M. D. Ward, *Organometallics*, **6**,754, **1987**.

9.
"Electrocrystallization
of Poorly Conducting Charge-Transfer Complexes",
M. D. Ward, *Inorg. Chem.*,
**25**, 4444, **1986**.

8.
"Photocatalytic
Alcohol Dehydrogenation Using Ammonium Heptamolybdate",
M. D. Ward, J. F. Brazdil,
and R. K. Grasselli, *J. Phys. Chem.* **88**, 4210, **1984**.

7.
"Electrochemical
Investigation of the Energetics of Particulate Titanium Dioxide Photocatalysts.
The Methyl Viologen-Acetate System",
M. D. Ward, J. R. White, and A. J. Bard, *J.
Am. Chem. Soc.*, **105**, 27, **1983**.

6.
"Photocurrent
Enhancement via Trapping of Photogenerated Electrons of TiO2 Particles",
M. D. Ward and A. J. Bard, *J. Phys. Chem.*, **86**, 3599, **1982**.

5.
"Electrophilic
Chemistry of Oxide-Supported Rhodium Complexes. Activation of H-H and Alkane C-H
Bonds",
M. D. Ward and J. Schwartz, *Organometallics*, **1**, 1030,  **1982**.

4. "Silica-Bound
Rhodium Hydride Catalysts for Arene Hydogenation",
M. D. Ward and J. Schwartz,  *J. Am. Chem. Soc*., **103**, 5253, **1981.** 

3. "Catalytically Active Metal Oxide-Supported Rhodium Hydride Complexes",
M. D. Ward and J. Schwartz, *J. Mol. Catal.*, **11**, 397, **1981**.

2.
"Synthesis
and Partial Characterization of (Silica)rhodium Dihydrides: A New Catalyst for
Olefin Hydrogenation",
M. D. Ward, T. V. Harris, and J. Schwartz, *J. Chem. Soc.
Chem. Comm.*, 357, **1980**.

1. "Silica-Supported
Zirconium Hydrides as Isomerization or Hydrogenation Catalysts",
J. Schwartz and M. D. Ward, *J. Mol. Catal.*, **8**, 465, **1980**.

---

|  |  |  |
| --- | --- | --- |
|  |  | **Main Page** |
