# Supplementary material for: Space groups and crystallographic symmetry: writing a multi-featured tutorial in a new style
Source: Acta Crystallogr E Crystallogr Commun. 2021 Jul 16;77(Pt 9):857–63. doi: 10.1107/S2056989021007039 (PMC8423017; doi:10.1107/S2056989021007039)
Supplement: Supplementary file 1 [file e-77-00857-sup2.zip › symandsg/Main/warren.htm]

Bertram Eugene Warren (1902-1991) - Obituary


---

# Bertram Eugene Warren (1902-1991) - Obituary

*J. Appl. Cryst.* (1996). **29**, 309-310.

**Bertram Eugene Warren  
1902-1991**

Bertram Eugene Warren was born in 1902
and died on June 27, 1991, one day short of his 89th
birthday. He was one of the brilliant long-lived X-ray
crystallographers, like Ewald, Laue, Bragg, Debye and Pauling. His
research and teaching were characterized by exceptional clarity and
devotion to two guiding principles: make as few assumptions as possible
and reduce theory to a state where measurements can be made. He taught
his course to students over a 40 year period and attracted many capable
students to his laboratory. There were students from France, Germany,
Norway, Australia, Finland and, of course, from the USA, as well as
other countries.

Warren was born in Waltham and, as one could tell from his Boston
accent, he was a real New Englander. He studied at MIT over the periods
1919-1925 and 1927-1929. In 1926, he was at the Technische
Hochschule Stuttgart studying under Glocker and Ewald. In 1927 W. L.
Bragg was at MIT and Warren was assigned to assist him, and this
interaction grew into a silicate structure paper. Subsequently, Bragg
became his dissertation supervisor, with the topic being metasilicate
structures. For the year 1929-1930, Warren was at Bragg's home
institution (University of Manchester) working with Bragg and
R. W. James. In 1930,
Warren returned as Assistant Professor of Physics to MIT, where he spent
the rest of his academic life.

Warren continued his early interest in silicate structures for a
brief period, but soon moved away from pure crystallography with some
important exceptions. In 1933 he confirmed Zachariasen's hypothesis
concerning the structure of glass and continued with this interest until
1942. In a classical Warren approach, he returned to the study of
glasses with his last graduate student, R. L. Mozzi, in 1966 after
experimental and other techniques had been greatly improved.

In the period 1933-1936, A. L. Patterson was a guest in
Warren's laboratory and was enamored of utilizing Fourier series in the
determination of crystal structures. He was exposed to the method of
Warren and Gingrich in applying Fourier integral analysis to liquids and
powder patterns. It may be that it was Warren who provided Patterson
with the final key to the
|*F*|^2^
problem but Warren never claimed any credit. Warren's scientific
generosity is also seen in his calling short-range order coefficients
Cowley coefficients, while Cowley called them Warren coefficients.

Warren's main efforts were not so much in expanding and improving
methods of structure determination, although his contributions here were
significant, but in developing a newer application of diffraction: the
investigation of nonperiodic and nearly periodic structures through
quantitative measurement of X-ray intensities. His greatest
contributions to science lie in his creation of mathematical and
experimental techniques for deriving information on the structure of
matter from X-ray scattering not necessarily restricted to Bragg
reflections. The glass studies were only the beginning of these. Warren
himself has said, in this connection, `As with humans, it is the
deviations from regularity that are more interesting'.

During this period of the thirties there were structure
determinations by Warren and coworkers of simple substances that had not
previously been done because of various difficulties; these included
rhombic sulfur, black phosphorus, crystalline bromine, uranium and
carbon black. Again, Warren returned to the study of carbon black in
1954 and 1965 with the improved methods available.

Warren had the idea that short-range order would be liquid-like and
that it would produce a modulated intensity from which order parameters
could be obtained by Fourier methods. In the 1950's, this was realized
in a number of studies by Warren's students. Temperature diffuse
scattering (TDS) also became of interest; these studies were important
in solid-state physics since TDS results from thermal vibration. Also in
the 1950's, Warren developed mathematical methods by which Fourier
analyses of line profiles were able to give crystallite sizes, strain
distributions and stacking-fault distributions.

Thus the early interest in deviations from regularity had been
continued by Warren into his most recent publications (1976, 1978),
which were concerned with temperature effects on powder patterns and
with powder-pattern intensity distributions from very small crystals. He
did not remain static in his interests, one subject leading him into the
next. Then, when techniques and corrections were improved, he was right
back into the old game and making order of magnitude improvements. This
always produced excitement around the laboratory and this was expecially
obvious to the visitor.

If Warren had not been a great teacher, it is doubtful that there
would have been anything like the Warren School that did come about.
Warren's teaching was on a par with his research; there were beautifully
organized lectures, slides, models and examples from his own research.
He began with the fundamentals and developed the solution to the problem
within the problem with inexorable logic. There was never any fuzziness;
only after the argument had been carried as far as possible were
assumptions inserted. The working relations were then developed so that
theory could be related to the results of experiments. When we worked on
problems for Warren, we were experimental and theoretical
physicists.

Warren corrected every homework problem, every laboratory report,
and the lengthy home examinations with meticulous care. His door was
open six days a week. Students (and ex-students) could enter, ask
questions, discuss results and learn physics. Often, graduate students
who were working for other professors expressed envy of those who were
in Warren's group. His help to those who were not members of his own
dissertation group was remarkably generous and effective.

If Warren had not had the character he had, the `Warren School of
X-ray Diffraction' probably would not have been as effective. While he
set a high standard for himself and demanded the same from his students,
he established a rapport with them which was uncommon. He was enormously
proud of his `boys'. Elna Warren, his wife, wrote that `to him, his
graduate students were his life'. He often invited his students to go
fishing with him at his camp in Maine and often had lobster cook-outs on
Crane's Beach. A scene that sticks in my mind is that of Warren striding
down a street leading a pack of ten or twelve of his students and
ex-students to dinner while at the Pittsburgh Diffraction Conference in
the fifties. In those days, we were a major part of the
Conference.

Warren was a man without false pride. He would, at conferences at
remote locations, just as well fraternize with the gatekeeper as with
the eminent scientists. It did not upset him that MIT put four retired
physics professors, including himself, in one room. The fact that a
physics secretary did not know who he was soon after he had retired did
not bother him. In 1979, after having written his last paper, he stated
that order-disorder in crystals was perhaps not the most important
thing in the world.

In 1984, when he was up at his camp in Maine, he measured the lake
acidity daily and, in a blistering statement, condemned Reagan for
inaction on acid rain. He applied his skill to the Indian method of
starting fires by rubbing sticks together and achieved the remarkable
result of producing fire in under one minute. He was a licensed Maine
guide but, during one camping trip, a missed communication led to a
newspaper headline of `MIT Prof Lost in Woods'.

B. E. Warren was a stout friend and as loyal to his students as
they were to him. He would write references and say to the person, `I
lied as much as I could'. He was married to his wife Elna for 56 years.
His life was an example to us and, even if we are not up to his
standard, we are better human beings for having known him.

LEONARD
MULDAWER

Index to Crystallographers

---

Updated 12th April 1997

**Copyright © 1996 International Union of Crystallography**

IUCr Webmaster
