# Supplementary material for: Space groups and crystallographic symmetry: writing a multi-featured tutorial in a new style
Source: Acta Crystallogr E Crystallogr Commun. 2021 Jul 16;77(Pt 9):857–63. doi: 10.1107/S2056989021007039 (PMC8423017; doi:10.1107/S2056989021007039)
Supplement: Supplementary file 1 [file e-77-00857-sup2.zip › symandsg/Main/wh-bragg-bio.html]

William Bragg - Biography

- Nobel Foundation
- Nobel Media
- Nobel Museum
- Nobel Peace Center
- Nobel Web
- SEARCH
- CONTACT US
- HOME


- NOBEL PRIZES
- ALFRED NOBEL
- PRIZE AWARDERS
- NOMINATION
- PRIZE ANNOUNCEMENTS
- AWARD CEREMONIES
- EDUCATIONAL GAMES

- By Year
- Nobel Prize in Physics
- Nobel Prize in Chemistry
- Nobel Prize in Medicine
- Nobel Prize in Literature
- Nobel Peace Prize
- Prize in Economics


## William Bragg

The Nobel Prize in Physics 1915

## Biography

**William Henry Bragg** was
born at Westward, Cumberland, on July 2, 1862. He was educated at
Market Harborough Grammar School and afterwards at King William's
College, Isle of Man. Elected a minor scholar of Trinity College,
Cambridge, in
1881, he studied mathematics under the well-known teacher, Dr. E.
J. Routh. He was Third Wrangler in the Mathematical Tripos, Part
I, in June 1884, and was placed in the first class in Part II in
the following January. He studied physics in the Cavendish
Laboratory during part of 1885, and at the end of that year
was elected to the Professorship of Mathematics and Physics in
the University of Adelaide, South Australia.
Subsequently he became successively Cavendish Professor of
Physics at Leeds (1909-1915), Quain Professor of Physics at
University College
London (1915-1925), and Fullerian Professor of Chemistry in
the Royal
Institution.  
  
His research interests embraced a great many topics and he was an
adept at picking up a subject, almost casually, making an
important contribution, then dropping it again. However, the work
of Bragg and his son Lawrence in 1913-1914 founded a new branch
of science of the greatest importance and significance, the
analysis of crystal structure by means of X-rays. If the
fundamental discovery of the wave aspect of X-rays, as evidenced
by their diffraction in crystals, was due to von Laue and his collaborators, it is
equally true that the use of X-rays as an instrument for the
systematic revelation of the way in which crystals are built was
entirely due to the Braggs. This was recognized by the award of
the Nobel Prize jointly to father and
son in 1915.  
  
During the First World War, Bragg was put in charge of research
on the detection and measurement of underwater sounds in
connection with the location of submarines. It was probably in
acknowledgement of his work, as well as of his scientific
eminence, that Bragg was made a C.B.E. in 1917 and was knighted
in 1920. The Order of Merit followed in 1931. After having been a
Fellow since 1907, he was elected President of the Royal Society in
1935.  
  
He was an honorary doctor of some sixteen universities, and a
member of the leading foreign societies. Many other medals and
awards were bestowed upon him among which may be mentioned the
Rumford Medal in 1916 and the Copley Medal (its premier award) in
1930.  
  
He was the author of many books, including *Studies in
Radioactivity; X-Rays and Crystal Structure; The World of Sound;
Concerning the Nature of Things; Old Trades and New Knowledge; An
Introduction to Crystal Analysis,* and *The Universe of
Light.* His favorite recreation was golf.  
  
In 1889 he married Gwendoline Todd, daughter of Sir Charles Todd,
F.R.S., Postmaster General and Government Astronomer of South
Australia. Their son William Lawrence
Bragg shared the Nobel Prize with his father.  
  
After a life of astonishing productiveness, Sir William Bragg
died on March 10, 1942.

From *Nobel Lectures, Physics 1901-1921*, Elsevier Publishing Company, Amsterdam, 1967


This autobiography/biography was first
published in the book series *Les
Prix Nobel*.
It was later edited and republished in *Nobel
Lectures*.
To cite this document, always state the source as shown above.

Copyright � The Nobel Foundation 1915

- Printer Friendly
- Comments & Questions
- Tell a Friend

#### The 1915 Prize in:

PhysicsChemistryMedicineLiteraturePeace

- Prev. year
- Next year

#### The Nobel Prize in Physics 1915

- Presentation

##### William Bragg

- Biography
- Nobel Lecture
- Swedish Nobel Stamps

##### Lawrence Bragg

- Biography
- Nobel Lecture
- Swedish Nobel Stamps
- Other Resources

Only 33 out of 758 are women!

Who can nominate for a Nobel Prize?

"War leaves no victors, only victims."


The Official Web Site of the Nobel Foundation

Copyright � Nobel Web AB 2006
