# Supplementary material for: Space groups and crystallographic symmetry: writing a multi-featured tutorial in a new style
Source: Acta Crystallogr E Crystallogr Commun. 2021 Jul 16;77(Pt 9):857–63. doi: 10.1107/S2056989021007039 (PMC8423017; doi:10.1107/S2056989021007039)
Supplement: Supplementary file 1 [file e-77-00857-sup2.zip › symandsg/Main/wl-bragg-bio.html]

Lawrence Bragg - Biography

- Nobel Foundation
- Nobel Media
- Nobel Museum
- Nobel Peace Center
- Nobel Web
- SEARCH
- CONTACT US
- HOME


- NOBEL PRIZES
- ALFRED NOBEL
- PRIZE AWARDERS
- NOMINATION
- PRIZE ANNOUNCEMENTS
- AWARD CEREMONIES
- EDUCATIONAL GAMES

- By Year
- Nobel Prize in Physics
- Nobel Prize in Chemistry
- Nobel Prize in Medicine
- Nobel Prize in Literature
- Nobel Peace Prize
- Prize in Economics


## Lawrence Bragg

The Nobel Prize in Physics 1915

## Biography

**William Lawrence Bragg**,
son of William Henry Bragg, was born in Adelaide, South
Australia, on March 31, 1890. He received his early education at
St. Peter's College in his birthplace, proceeding to Adelaide
University to take his degree in mathematics with first-class
honours in 1908. He came to England with his father in 1909 and
entered Trinity College, Cambridge, as an Allen Scholar, taking
first-class honours in the Natural Science Tripos in 1912. In the
autumn of this year he commenced his examination of the von Laue phenomenon and published his
first paper on the subject in the *Proceedings of the Cambridge
Philosophical Society* in November.  
  
In 1914 he was appointed as Fellow and Lecturer in Natural
Sciences at Trinity College and the same year he was awarded the
Barnard Medal. From 1912 to 1914 he had been working with his
father, and the results of their work were published in an
abridged form in *X-rays and Crystal Structure* (1915). It
was this work which earned them jointly the Nobel Prize for Physics in 1915, and from this
year to 1919, W. L. Bragg served as Technical Advisor on Sound
Ranging to the Map Section, G.H.Q., France, receiving the O.B.E.
and the M.C. in 1918. He was appointed Langworthy Professor of
Physics at Manchester University in 1919, and held this post
till 1937.  
  
W. Lawrence Bragg, who had been elected Fellow of the Royal Society in
1921, was Director of the National Physical Laboratory in 1937-1938 and
Cavendish Professor of Experimental Physics, Cambridge, from 1938
to 1953. He was Chairman of the Frequency Advisory Committee from
1958 to 1960.  
  
Knighted in 1941, Sir Lawrence holds the degree of M.A.
(Cambridge), Honorary D.Sc. (Dublin, Leeds, Manchester,
Lisbon, Paris,
Brussels, Liege, and Durham), honorary Ph.D. (Cologne), and honorary LL.D. (St.Andrews).
He has many honorary fellowships and is an honorary or foreign
member of American, French, Swedish, Chinese, Dutch, and Belgian Scientific
Academies, besides being Membre d'Honneur de la Soci�t�
Fran�aise de Min�ralogies et Cristallographie.  
  
He was awarded the Hughes Medal of the Royal Society in 1931; the
Royal Medal of the same Society in 1946, and the Roebling Medal
of the Mineral Society of America in 1948.  
  
Together with his father, he has published various scientific
papers on crystal structure after their joint publication of
1915: *The Crystalline State* (1934), *Electricity*
(1936), and *Atomic Structure of Minerals* (1937).  
  
Sir Lawrence's chief interests at the present time are the
application of X-ray analysis to the structure of protein
molecules, which are being investigated in the Davy Faraday
Laboratory of the Royal Institution, in continuation of similar work
at the Cavendish Laboratory, Cambridge. This collaboration
has succeeded in determining for the first time the structure of
the highly complex molecules of living matter.  
  
Having been awarded the Nobel Prize at the very early age of 25,
W. Lawrence Bragg was the youngest-ever laureate. The very rare
opportunity of celebrating a golden jubilee as a Nobel Laureate
was given special attention during the December ceremonies at
Stockholm in 1965, when Sir Lawrence, at the invitation of the
Nobel Foundation, delivered a
lecture - the first Nobel Guest Lecture - in retrospect, on
developments in his field of interest during the last fifty
years.  
  
In 1921 he married Alice Grace Jenny (*n�e* Hopkinson)
of Cambridge, and they have two sons (the elder of whom became
chief scientist with Rolls Royce, while the younger entered a
Cambridge instrument-making firm), and two daughters (the elder
of whom married an official of the Foreign Office, while the
second married the son of the Master of Corpus Christi
College, Cambridge).

From *Nobel Lectures, Physics 1901-1921*, Elsevier Publishing Company, Amsterdam, 1967


This autobiography/biography was first
published in the book series *Les
Prix Nobel*.
It was later edited and republished in *Nobel
Lectures*.
To cite this document, always state the source as shown above.

*Lawrence Bragg died on July 1, 1971.*

Copyright � The Nobel Foundation 1915

- Printer Friendly
- Comments & Questions
- Tell a Friend

#### The 1915 Prize in:

PhysicsChemistryMedicineLiteraturePeace

- Prev. year
- Next year

#### The Nobel Prize in Physics 1915

- Presentation

##### William Bragg

- Biography
- Nobel Lecture
- Swedish Nobel Stamps

##### Lawrence Bragg

- Biography
- Nobel Lecture
- Swedish Nobel Stamps
- Other Resources

Only 33 out of 758 are women!

Who can nominate for a Nobel Prize?

"War leaves no victors, only victims."


The Official Web Site of the Nobel Foundation

Copyright � Nobel Web AB 2006
