# Supplementary material for: Space groups and crystallographic symmetry: writing a multi-featured tutorial in a new style
Source: Acta Crystallogr E Crystallogr Commun. 2021 Jul 16;77(Pt 9):857–63. doi: 10.1107/S2056989021007039 (PMC8423017; doi:10.1107/S2056989021007039)
Supplement: Supplementary file 1 [file e-77-00857-sup2.zip › symandsg/Main/wyckoff.htm]

(IUCr) Crystallographers Ralph W. G. Wyckoff (1897-1994)


---

# Ralph W. G. Wyckoff (1897-1994) - Obituary

*Acta Cryst.* (1995). A**51**, 649-650.


  

**Ralph W. G. Wyckoff  
1897-1994**

On 3 November 1994, Ralph Walter Graystone Wyckoff died at the age
of 97. Wyckoff was a scientist with many talents and was extremely
productive. He published more than 400 papers; the first appeared when
he was 19 and the last one approximately 70 years later. During that
long and active period, he was employed in no less than eleven
laboratories of very different types. His strongest side was to apply
new techniques or use new instruments, when they became available, in an
intelligent way. It was not basic discoveries but the recognition of
what had become possible at a certain moment that characterized his
scientific career. In this way, he became famous in two areas of
structural research: X-ray diffraction and electron microscopy.

Ralph was born on 9 August 1897 in Geneva in New York State as the
son of the judge Abram Ralph Wyckoff and Ethel Agnes (n�e
Catchpole) Wyckoff. His father stemmed from Dutch immigrants who arrived
in America in 1634. His mother was born in England and went to the USA
around 1880. He spent his youth and went to school in Geneva. Although
left-handed by nature, the tough discipline at school forced him to
write with his right hand. This made him able to use both hands easily,
which was an advantage in his later experimental work. His scientific
career started at Hobart College in his home town. There he took all
possible chemistry classes, became an assistant and wrote his first
publication in 1916, which appeared in the *Journal of
the American Chemical Society*.

With his BS degree from Hobart College,
Ralph moved, at 19 years old, to Cornell University in Ithaca. It had
always been his dream to become an astronomer. Unfortunately, the war
had depleted the Astronomy Department of its faculty and this prevented
him from realising his dream. He therefore decided to read chemistry as
an alternative way to study matter in the universe! At Cornell, Wyckoff
started to work on X-ray diffraction. This choice was strongly
influenced by the Japanese crystallographer S. Nishikawa, who spent a
couple of years in the Physics Department of Cornell. With Nishikawa as
supervisor, he determined the structures of NaNO_3_ and
CsICl_2_, the subjects of his thesis, which he defended in 1919
at the age of 21. The linear and symmetric structure of the
[Cl-I-Cl]^-^ ion was
a surprising result. It pointed to an unknown type of bond, which held
the interest of structural chemists for many years.

After his graduation from Cornell, a period
of fruitful structural research followed in the Geophysical Laboratory
in Washington (DC). The emphasis was on minerals with high symmetry.
Like Nishikawa, Wyckoff collected X-ray diffraction data from
multi-wavelength Laue patterns and powder patterns. The classification
of compounds into structure types according to Schoenflies' space-group
theory played an important role in the structure determinations. The one
or two parameters not determined by symmetry were roughly estimated from
the relative intensities of a small number of reflections. This was in
striking contrast to the procedure used by British crystallographers.
They followed the Braggs, used monochromatic radiation and regarded
quantitative intensity measurements as essential. However, they tended
to underestimate the value of a systematic structural theory. This
controversy disappeared automatically when the study of more complicated
structures required both accurate intensities and knowledge of the space
groups.

After some delay due to this controversy, Wyckoff published his
book *The Analytical Expression of the Results of the
Theory of Space Groups* in 1922. It contained tables
with the positional coordinates, both general and special, permitted by
the symmetry elements. This book formed the prelude to the description
of the 230 space groups in *International Tables for
X-ray Crystallography*, the first edition of which
appeared in 1935. In 1924, Wyckoff prepared another book,
*The Structure of Crystals*, a
compilation of X-ray structural work known at the time. For many years,
he continued the description of all known crystal structures with the
exception of non-stoichiometric intermetallic compounds. The last
edition of his popular series *Crystal
Structures* appeared between 1963 (part I) and 1971
(part VI-2).

In 1927, Wyckoff moved to the Rockefeller Institute for Medical
Research intending to extend his studies to organic compounds. However,
this decision led very soon and unintentionally to the end of his own
experimental X-ray work. At the Rockefeller he started to construct an
X-ray spectrometer provided with an ionization counter. He used the
instrument to determine the structure of urea and also collected data
for the amino acid glycine with the ultimate goal of studying proteins.
Unfortunately, it soon appeared that the Rockefeller Institute was no
longer interested in X-ray crystallography. Wyckoff's co-worker R. B.
Corey left for the California Institute for Technology in Pasadena.
Wyckoff himself stayed on for a while and used his instruments to
measure the effect of X-rays on living cells. In those New York years,
his private life also changed. After a first marriage, which did not
last very long, he remarried and in this happy marriage three daughters
were born.

An activity that he undertook in a quite different field was the
construction of an ultracentrifuge of improved design for the isolation
and purification of proteins and viruses. After being dismissed from the
Rockefeller Institute in 1937, he worked in a few industrial
laboratories. The industrial interest was in a virus causing Western
Equine Encephalomyelitis, which killed thousands of horses in the
western United States. The purification of this virus led to the
production of a successful vaccine against the illness. Subsequently,
with the advent of the Second World War, he produced first a vaccine
against epidemic typhus fever and then dried blood plasma for in-field
use by the armed forces. After terminating his relations with industry,
Wyckoff was unemployed for several months. Although he tried very hard,
his applications did not bring him a job until, in 1943, he was offered
the chance to work part-time at the University of Michigan and for the
rest of the time at the Michigan State Department of Health. He was not
completely happy in Ann Arbor until he found an unused electron
microscope in the basement of the Bacteriology Laboratory and succeeded
in taking beautiful pictures of the influenza virus. They were
especially appealing because of the metal shadowing technique, which
provided a three-dimensional image. This success provided him with a
position at the National Institutes of Health in Bethesda. There, he
spent happy and fruitful years (1946 to 1952) studying viruses and
macromolecules. He was asked to show his impressive pictures at a
meeting of European crystallographers in London (UK). Here, as a
prominent American crystallographer, he became engaged in the foundation
of what in 1948 would become the International Union of Crystallography,
of which he was Vice-president and President from 1951 to 1957. While in
London, he met many European scientists, which led to several life-long
friendships. In the post-war period, Wyckoff was mainly engaged in
electron microscopy and in organizational activities in crystallography.
He travelled a great deal and felt at home in Europe. He therefore
accepted without hesitation an offer to be scientific attach� at
the American embassy in London for a period of two years. Even there he
continued his electron microscopy research on a modest scale with an
instrument provided by the Philips Company.

His return to Bethesda was a great disappointment. He deeply
disliked the bureaucracy in the enormously expanded National Institutes
of Health and in 1960 he decided to leave before being retired. He
moved to the University of Arizona in Tucson to become a Professor in
physics and microbiology. In Arizona he focused his interest on fossils,
which he studied with the electron microscope and by chemical analysis.
When the rules of the University forced him to retire, he was already
over 80 years old. He continued to work for a while with a copper mining
company, but this also came to an end when he was 88. He spent the
remaining years of his life in California.

Ralph Wyckoff had enormous energy. He did not obey official
working hours; one could often find him at his microscope at 6 o'clock
in the morning or at 2 o'clock at night. However, he was by no means an
unworldly scientist. He had a deep interest in politics and in cultural
matters, and loved classical music. He enjoyed travelling, especially in
Europe. Many honours and honorary positions were bestowed on him and he
was a member of several prominent scientific institutions.

We thank Eelco H. Wiebenga for personal information and
Mike H. Dacombe for improving the language.

JAN DRENTH
  
AAFJE LOOYENGA-VOS

Index to Crystallographers

---

Updated 2nd May 1997

**Copyright � 1995 International Union of Crystallography**

IUCr Webmaster
